# An exploratory study of the correlates of weight-based mistreatment at work

**DOI:** 10.1371/journal.pone.0342144

**Published:** 2026-08-26

**Authors:** Grace Lemmon, Jaclyn M. Jensen, Goran Kuljanin, Mounica Reddy

**Affiliations:** 1 Department of Management and Entrepreneurship, DePaul University, Chicago, Illinois, United States of America; 2 Department of Psychology, DePaul University, Chicago, Illinois, United States of America; University of Beira Interior: Universidade da Beira Interior, PORTUGAL

## Abstract

Weight-based mistreatment (WBM), which reflects mistreatment through hostility, threats, underhanded comments, physical violence, or microaggressions that explicitly targets a person’s larger body size, occurs frequently in the workplace. To begin to uncover why it occurs, this research documents correlates of WBM by investigating stigmatization of weight through four conceptual frames: stereotyping, the justification-suppression model of prejudice, objectification theory, and occupational contexts. Across a sample of 1,008 self-identified larger-bodied respondents, 383 paired coworkers, and data from O*NET about occupational features of the larger-bodied respondents’ jobs, we conducted a cross-sectional quantitative exploratory study. Results suggest that larger-bodied employees in workplace climates that promote thinness (e.g., where talk emphasizing that coworkers should strive to be thinner is common) or who fail to regulate or condemn negative body talk tend to report more WBM, as do employees in more generally unsupportive or aggressive interpersonal professional climates. Furthermore, employees’ self-views contribute to differences between rates of WBM: larger employees who report states of self-evaluated vulnerability – that is, believing that they do not measure up to peers or feeling generally unworthy – also report more WBM. Taken together, results suggest that correlates of WBM are multi-factorial, germinating from weight stigmatizing perceptions that originate within the self, from coworkers, and various attributes of workplace climates.

## Introduction

Weight-based mistreatment, defined as hostility, threats, underhanded comments, physical violence, or microaggressions that explicitly target a person’s larger body size, is a significant issue in today’s society [1], In the workplace alone, 75% of larger-bodied employees report experiencing some form of WBM within the past six months perpetrated by colleagues, bosses, direct reports, and customers [1]. Serious and harmful outcomes of weight-based mistreatment (WBM) include performance decrements, self-isolation, and retaliatory acts at work, as well as self-reported disordered health behavior and mental health crises [1,2]. As such, addressing the problem of WBM requires examination of correlates that potentially sanction or amplify it, such as relational contexts, organizational cultures, and power dynamics [3–6]. Anti-fat stigmatization is undergirded by the belief that fatness is objectively wrong, that larger-bodied people cannot perform their roles effectively, that fatness is both an urgent and entirely controllable problem, and/or that larger-bodied people deserve mistreatment because of their personal “failing” to manage their weight. We argue that workplace features that echo or endorse the central tenants of anti-fat stigmatization co-occur with WBM.

We use four frames to explore these workplace features: Frame 1 leverages *stereotyping*, which is stigmatization through the application of unfavorable stereotypes related to higher body weight [7]. Frame 2 uses the *justification-suppression model of prejudice* to examine a broader array of target and work environment features that potentially enhance or minimize rates of stigmatization of weight depending on how salient those features make body weight, disrespect, or adverse power dynamics [8]. Frame 3 looks at how objectification of the body by oneself and others manifest as attitudes that enhance the vulnerability of a larger-bodied person to predation [9,10]. Finally, Frame 4 (*occupational contexts*) looks at if job features that call attention to the stigmatized attribute of weight relate to WBM incidence [11]. To examine correlates of WBM through these four frames, we use three sources of data: self-reports from mistreated and non-mistreated larger-bodied workers, matched co-worker data, and occupational data linked to each larger-bodied worker’s occupation.

This research on correlates of WBM matters for several reasons. First, WBM stigmatizes larger-bodied people, bringing intrapersonal pain: many larger-bodied people report a host of psychological and physical maladies when encountering mistreatment due to their weight [12–15]. Second, the experience of abuse and/or ostracism impedes formation of collaborative relationships with colleagues and the organization, which harms work effectiveness [1,16–18]. Third, weight stigmatization also directly influences organizational costs, such as increasing rates of absenteeism and withdrawal (1,e.g., [19]). Finally, while many organizations increasingly espouse values of inclusivity [20], to let WBM occur at such significant rates can undercut this commitment to organizational promises of inclusion and belongingness [3]. Understanding its correlates provides key information about how to reduce it.

### Research questions

Perpetrators of mistreatment often target those who are socially vulnerable and focus on that vulnerability during the mistreatment encounter [21–23]. In the case of WBM, the target’s vulnerability is their body size. Here we argue that WBM tends to happen alongside intra- and interpersonal dynamics and contexts that enhance weight-based stigmatization of larger body sizes. Stigmatization occurs when a person is devalued, denigrated, or otherwise inappropriately or unfairly considered because of a single attribute considered unacceptable to society [24,25]. Across much of the global population, body weight is strongly stigmatized in modern culture [26–28]. For example, the stigma is so strong that among 4,000 “normal” weight people, 46% stated they would trade one year of life over being identified as “obese,” and 30% said they would prefer divorce to “obesity” [29]. (Please note: to not medicalize body weight, we use quotes around weight classifications and note that the categories reflect the study author’s framings to participants [30]) Furthermore, stigmatization of weight is not improving over time, even with social movements urging inclusivity [31–34]. In U.S. samples, greater unconscious bias against larger-bodied people exists compared to those who are gay, Muslim, disabled, of darker skin tone, or older in age [32,35]. Finally, the discrimination levied against those stigmatized on the basis of weight in employment practices has measurable and long-term effects on the target’s economic (e.g., pay, selection, promotion) and mental (e.g., depression, anxiety) well-being [36]. We next discuss four frames for understanding correlates of WBM.

### Frame 1: Stereotyping

We stigmatize those in larger bodies as both cold and incompetent and feel punitive toward those who display such characteristics [7,37,38]. Specifically, a larger-bodied person is stereotyped as lacking warmth because they are presumed to prioritize their own desires over others’ needs; this indicates greediness and indulgence at the expense of others, such as assumption that they take more food or rest than others; [39] They are also presumed to lack competence because their bodies are assumed to be intrinsically ill and thus cannot effectively meet productivity goals [39,40]. Furthermore, underlaying both assumptions is the belief that larger-bodied people lack self-regulation skills because weight is controllable [7,38]. Furthermore, these assumptions are held by *all* individuals regardless of their own body size; in other words, larger-bodied people also judge themselves and fellow larger-bodied people through these stereotypical frames [41]. As such, we examine larger-bodied employees’ warmth and competence perceptions of themselves, how they think coworkers view them, and how coworkers report viewing them as correlates of WBM. As a research question (RQ), we ask:

***RQ1*** *: What differences exist in perceptions of competence and warmth between larger-bodied workers who experienced WBM and those who did not? Overall, for those who experienced WBM, to what extent do perceptions of competence and warmth account for the rate of WBM and which perceptions matter most?*

### Frame 2: Justification-suppression model of prejudice

Stigmatization of people in larger body sizes may be enhanced or minimized by features of the target and their environment that signal the acceptability of weight stigmatization. The justification-suppression model of prejudice explains this phenomenon: bias toward stigmatized characteristics is swift and often unconscious. Effortful processing of additional information either reinforces prejudice or diminishes it, producing a predictable degree of discrimination [8]. By way of example, research finds that larger-bodied people are thought of as less deserving of help, attention, or general interpersonal consideration because of intrinsic stigmatization of their body weight [42–44]. This effect is magnified when a larger person exhibits a stereotype-consistent behavior, like drinking a milkshake or not dressing professionally), which serves as signal that initial stereotypes were justified. However, this effect is muted when a larger-bodied person exhibits a signal counter to a stereotype, such as when they talk of exercise or eat a meal colloquially understood to be “healthy.”

In the present study, we explore signals that may prompt justification or suppression of stigmatization of body size vis-à-vis WBM. We first examine attributes of the *interpersonal climate* at work. This climate serves as a broad signaling about allowance or acceptability of aggressive and unbecoming behavior between employees, with greater amounts of disrespect and poorer relations enabling perpetrators to justify mistreatment of larger-bodied targets under the pretext that disregarding the well-being of colleagues and attacking their vulnerable attribute is the norm [45,46]. Similarly, we anticipate an association between aggressive interpersonal dynamics at work and WBM. We specifically examine perceptions of the quality of relationship between an organization and its employees, between coworkers, and between bosses and direct reports, as well as a more direct assessment of day-to-day interpersonal conflict among peers. Finally, we also examine inclusivity norms for integration of employee voice around distribution of resources and decision-making [47]. Such patterns of relations reflect prioritization of inclusion/acceptance, respect, and helping versus exclusion, disrespect, and obstructionism.

***RQ2*** *: What differences exist in perceptions of interpersonal climate between larger-bodied workers who experienced WBM and those who did not? Overall, for those who experienced WBM, to what extent do perceptions of interpersonal climate account for the rate of WBM and which perceptions matter most?*

The workplace climate can also specifically reflect how frequently body weight is talked about. For example, at work, is it common to talk about body size, to talk about wanting to be a particular ideal body size, to talk through exercise routines and fitness goals, or to talk through various means to achieve certain body sizes? Such climates that reflect desires and intentions around *weight control* provide signals about the desirability of thinner bodies, and thus the unacceptability of being larger, invoking stigmatization of larger people that could provide justification for perpetrators to engage in WBM. We thus ask:

***RQ3*** *: What differences exist in climates related to weight control between larger-bodied workers who experienced WBM and those who did not? Overall, for those who experienced WBM, to what extent do climates related to weight account for the rate of WBM and which matter most?*

The workplace climate as it specifically pertains to weight may also be influenced by *human resource (HR) policies* that highlight weight. HR communications about health can consist of seemingly neutral messaging about weight, insofar as discussion revolves around mental health or getting enough rest. But they can also talk more explicitly about body size (e.g., a weight loss challenge), creating a climate wherein weight is a salient topic of communication, or even where negative views – that is, stigmatizations of weight – are normalized if the content of the HR policies highlight the importance of being a “healthy weight.” Furthermore, policies equivocating wellness and health have the unfortunate impact of reinforcing weight stigma by suggesting (or affirming existing beliefs) that weight and health are synonymous and that maintenance of a certain body size is a matter of personal choice and responsibility, providing pretext for derision of those failing to meet body weight ideals [48].

***RQ4*** *: What differences exist in HR offerings and communications between larger-bodied workers who experienced WBM and those who did not? Overall, for those who experienced WBM, to what extent do HR offerings and communications account for the rate of WBM and which matter most?*

Turning our attention to target attributes, we consider if non-weight physical attributes of the target indicative of social status compounds stigmatization of weight because perpetrators, again, target the most vulnerable [21]. Specifically, atrtibutes that make larger-bodied people more socially vulnerable, such as being less educated, being larger among larger-bodied peers, or one of a few women, may relate to instances of WBM because these larger-bodied people possess no status indicators to suppress stereotypes about their weight [49,50]. Relatedly, in contexts that prioritize appearance, a larger-bodied person may be mistreated more frequently because their non-ideal body size is more obvious as their stigmatized characteristic (weight) is primed [51]. Finally, through the lens of intersectionality, certain concurrent identities – here being a minority race *and* larger-bodied or being female *and* being larger-bodied – present particular vulnerabilities because stigmatized or historically subjugated identities reduce the social power of the target [52–54]. Recent research has argued, for example, that intersectionality considerations matter when examining weight-based stigmatization as certain groups contend with greater social pressure to conform to body size standards [55]. This pressure has material effects, as evidenced by the fact that women perceive rates of economic discrimination due to weight at 16 times the rate as men [56,57].

***RQ5*** *: What differences exist in variables related to non-weight-based physical attributes that indicate social status between larger-bodied workers who experienced WBM and those who did not? Overall, for those who experienced WBM, to what extent do these variables related to social status account for the rate of WBM and which matter most?*

Finally, we consider active strategies a larger-bodied person can enact to suppress perpetrator’s stigmatization of their weight. Shih et al. [58] introduced *identity management strategies* that do not require a larger-bodied person to necessarily change their size to achieve self-directed acceptance. Instead, these identity management strategies – specifically identity switching, which means focusing on other valued identities one possess and identity redefinition, which is focusing on re-orienting what it means to be larger-bodied – enable coping with an identity-based stigma one cannot readily shed [58]. When a larger person enacts these identity management strategies, they project strength and confidence to outsiders, potentially warding off attack from perpetrators who choose to target points of vulnerability; here the larger-bodied person’s confidence suppresses the perpetrator’s instinctual stigmatization of their vulnerability because that vulnerability is offset by target confidence [21,23]. We thus ask:

***RQ6*** *: What differences exist in identity management strategies between larger-bodied workers who experienced WBM and those who did not? Overall, for those who experienced WBM, to what extent do identity management strategies account for the rate of WBM and which matter most?*

### Frame 3: Objectification theory

Now that we have discussed attribute- and climate-based correlates of WBM, we can look more directly at factors that fuel stigmatization of body size. Objectification theory outlines why stigmatization toward body weight occurs: culturally, many of us accept that there is some ideal physical form (for all genders) (e.g., [59,60]); because of this acceptance, we normalize judgment of our bodies, specifically judgment of our bodies by other people and self-judgment of our own body weight; and we feel distress when we fail to measure up to a body ideal, often manifesting as depression and anxiety [10]. These feelings of distress merit a brief discussion because they are not, as one may initially assume, due to the fault of the target. The relentless body ideal pressure, combined with fear of *and* enacted social censure for non-conformity, depletes a person’s mental state [61,62]. Given this, a larger-bodied individuals may self-castigate in fear of the social repercussions of stigmatization rather than being despondent over not achieving objective assessment of an ideal body type [13].

Unfortunately, this self-castigation and its negative impact on self-esteem, quality of life, and negative affect, may make a larger-bodied person vulnerable to WBM as perpetrators select victims who appear weaker as there is less risk of the target fighting back effectively or retaliation [21,63–67]. As such, negative self-appraisals stemming from self-objectification leave larger-bodied people more vulnerable to WBM. As such, we question if targets who display indicators of *objectification of the self* may also report more mistreatment directed toward their body size. Such indicators include: feeling guilty because they sense they do not belong, shame in feeling like a social outsider, depression, poorly evaluating their own worth, believing their body is unworthy especially because it is larger and feeling self-conscious about it, lacking trust in their ability to care for their body, or dissatisfaction with their body,. We do not imply in the least that targets are responsible for the mistreatment due to their vulnerability, only that these distressed states reflect the lived reality of fearing the repercussions of existing while larger in size, that is, anticipated stigma [68].

***RQ7*** *: What differences exist in self-objectification variables between those larger-bodied workers who experienced WBM and those who did not? Overall, for those who experienced WBM, to what extent do self-objectification variables account for the rate of WBM and which matter most?*

Beyond the first theme of self-judgment, objectification theory suggests a second theme: that we articulate the content that underlies the body weight judgments. Specifically, the workplace climate created through *objectification of weight by others* reflects the form and intensity of stigmatization within those judgments. Objectification of the weight of others can be evaluated through investigating if a workplace typically conveys a positive, neutral, or denigrating posture toward fatness, as well as how strongly being thinner is internalized as an ideal, as both can impact the perceived legitimacy of judging those in larger bodies (e.g., [69]). Furthermore, “fat talk” – discussions about other bodies, as well as one’s own body, in a way that denigrates larger sizes – portrays larger-bodied people as objectively and inherently wrong and collectively perpetuates an attitude that valorizes smaller bodies [70–72]. We anticipate with stronger norms of stigmatization of body weight, more judgments enacted via WMB occur [9].

***RQ8*** *: What differences exist in others objectifying their weight between larger-bodied workers who experienced WBM and those who did not? Overall, for those who experienced WBM, to what extent does objectification of weight account for the rate of WBM and which attitudes matter most?*

### Frame 4: Task, social, and physical occupational contexts

In our final frame, we explore if WBM occurs alongside certain occupational contexts that make body size (or the stereotypes body size invokes) more salient, and thus riper for stigmatization. Objective and subjective boundaries shaped by elements that identify an occupation, such as interpersonal factors, physical demands, norms, regulations, and values, operationalize occupational context [73]. Because of the breadth of occupational context, researchers often utilize three occupational sub-contexts: task, social, and physical [74]. We know that as perceived fit with one’s job suffers because of weight stigmatizing assumptions, mistreatment can follow [75]. Our main argument is an occupation’s role requirements may create contexts in which snap judgments, including acting upon stigmatization through WBM, commonly occur. The *task occupational context* refers to the objective task environment, including structural and informational boundaries that affect work roles, such as autonomy and availability of information [11,76]. In contexts where the task environment constrains rather than facilitates work (e.g., low job autonomy), experiences of stress increase [77]. Social categorization processes (such as stereotyping) and reductions in social niceties may follow as a person tries to conserve mental resources, both of which relate to WBM likelihood.

The *physical occupational context* refers to elements of the material environment such as light, noise, temperature, and hazardous work conditions [11,78]. Because “obesity” has extensively been classified and considered a disease, larger-bodied people may be perceived as less equipped to deal with the physical conditions of their work [7,79]. Perceived incongruence between physical demands of work (e.g., the occupational context of a job that requires intense physical labor or perpetual motion) and an individual’s status as a healthy, able-bodied worker facilitates a larger-bodied person’s standing as a stigmatized outgroup member, which may relate to incidents of WBM.

Finally, the *social occupational context* comprises interpersonal relationships, interactions, and social contingencies that affect role performance [76]. Interdependence is a feature of the social occupational context that encompasses how role performance depends on social relationships. Highly interdependent work contexts involve cooperation to drive goal attainment whereby workers help each other to facilitate the completion of others’ work [76]. Larger-bodied people are less likely to be rated desirable coworkers [80], stereotyped as less agreeable [81], and depicted as greedy or selfish [7], making them less likely to be viewed as valuable social partners and deserving of punishment [82]. Given the foregoing, we anticipate:

***RQ9*** *: What differences exist in occupational context variables between those who experienced WBM and those who did not? Overall, for those who experienced WBM, to what extent do occupational context variables account for the rate of WBM, and which context variables matter most?*

## Materials and methods

### Procedure

We received approval from DePaul University’s Internal Review Board to collect data according to the method described below (GL030618BUS). From September 5-October 1, 2018, we posted a call on Amazon’s Mechanical Turk for self-defined “overweight” full-time workers employed in the U.S. to [1] respond to a survey about their workplace experiences, and [2] be willing to send a separate survey about workplace experiences to a coworker with whom they worked at least 20 hours per week in the same location. We did not provide a medical definition of “overweight” but instead let respondents self-select into the study if they considered themselves eligible. In terms of consent, after reviewing the study’s informed consent document, both focal respondent and co-workers clicked on>> within their survey if they wished to take it.

An initial screening questionnaire assessed the degree to which respondents had experienced WBM at work, including never, within the last 6 months, or prior to the last 6 months. To ascertain if they experienced WBM, we explicitly asked: *Have you ever been disrespected at work because of your work?* Subsequent questions probed the nature of this disrespect. Respondents in the first two groups (never and within the last 6 months), which we refer to now as “focal respondents,” were then directed to the full study survey. Individuals who experienced WBM prior to the last 6 months were excluded from further surveys due to concerns about recall accuracy.

Focal respondents passed stringent quality checks, including a selfie from the neck down with three fingers held over their stomach (an image that was not easily searchable online), sensical responses to open-ended questions about their job duties, and proper responses to attentional checks. They were compensated: $4 for the focal respondent’s initial survey; $5 bonus payment to them if their coworker responded; a $5 big box store gift card to the focal respondent’s coworker if they responded.

### Sample

Our survey call was answered by 2,618 respondents. Of those, 1,186 reported WBM prior to six months ago, and so we did not allow them to complete our survey due to concerns about recall of the context surrounding the event (e.g., recall of its correlates), although we acknowledge that the trauma of the event likely lingered beyond six months. In the remaining focal respondent sample, 1,008 passed quality checks, and we received complete and acceptable (i.e., appropriate amount of time spent; answered attentional quality check questions correctly; sensical responses to open-ended questions; uploaded selfie in mandated position) surveys from 383 matched co-workers (matched dyad rate = 38%). Of the focal respondents who passed quality checks, 758 reported WBM within the past six months (WBM rate of 758 / 1,008 = 75.2%) while the remaining individuals (n = 250) indicated they never experienced WBM. The focal respondents were mostly female (59.1% of sample), mostly white (77.0%; other racial categories include: black [9.1%], Hispanic [7.8%], Asian [4.4%]), were an average of 33.6 years old (SD = 8.84, range = 18–76 years old), worked with their current employer for 5.16 years, the modal education was a bachelor’s degree (36.5%; other educational categories include: some college [23.6%], associate degree [16.6%], high school diploma [11.0%]). Across the sample, workers all worked at least 20 hours per week among co-workers, and workers were employed across a diverse range of occupations (243 distinct occupations), ranging from healthcare to sales to legal. Coworkers were 56.8% female, mostly white (69.5%), were an average of 34.5 years old (SD = 9.23, range = 19–68), had worked at their current company for an average of 5.41 years, and reported working with the focal respondent an average of 3.62 years.

### Measures

Respondents who indicated in the initial screening survey that they had experienced WBM in the past six months (*n* = 758) completed items assessing nine different types of WBM (e.g., benevolent, covert, overt, three types of incivilities, three types of microaggressions) committed by five different perpetrators (e.g., bosses, coworkers, subordinates, customers, other) on Likert response ratings (1 = never, 2 = sometimes, 3 = often) [1]. See sample items in S1 Table. To keep the reporting of our analyses reasonable throughout this manuscript, we computed a single composite WBM score across all these ratings, which consisted of 140 items in total. Reliability estimates are provided in S2 Table. We chose a composite score because prior research has demonstrated high levels of interrelation among WBM types [1], and our theoretical arguments linking correlates to WBM does not differentiate correlates by WBM type nor WBM source. That said, for the interested reader, we provided a granular analysis of the nine research questions for the nine types of WBM and five types of WBM sources in the supplemental materials of the manuscript (corresponding to S3-S16 Tables). In all cases, we used the nine sets of correlational measures to predict WBM scores for the focal respondents who experienced WBM in the past six months. Notably, the results for the composite WBM score largely reflect the high-level pattern of the more granular results.

For correlational variables, we drew from existing measures whenever possible. Table 1 lists the variables collected based on the four frames of stigmatization, alongside their definition, a brief theoretical explanation for why they may correlate with WBM, and example item and response scale. Most correlational measures that were rated by focal respondents or their coworkers used Likert response scales. For correlational measures with three or more items, we provide Cronbach’s alpha (i.e., α) and omega-total (i.e., ω_t_; see Revelle and Zinbarg [83] for more information) as reliability estimates. For the correlational measures that we created for this study, we asked four expert raters to evaluate the extent to which purported items measure the construct of interest on a five-point Likert scale, and we report the expert rater agreement (i.e., r_wg_ for single item measures or r_wg(j)_ for multi-item measures; see James and colleagues [84] for more information) for these measures. For the occupational context frame, we asked focal respondents to provide their occupation title, and we matched the title to a corresponding occupation found on the Occupational Information Network (O*NET), a database containing myriad occupation-specific, validated descriptors of a little over 1,000 jobs in the U.S. Among the data provided on O*NET are ratings of each occupation’s task, social, and physical context (rated on a 1–5 scale). The physical context consisted of 30 ratings (e.g., exposed to hazardous conditions, cramped workspace), the task context consisted of 11 ratings (e.g., duration of typical work week, freedom to make decisions), and the social context consisted of 14 ratings (e.g., level of competition, work with group or team). For each occupation, we averaged the O*NET provided ratings for each context (i.e., physical, task, and social). We provide the correlations among all variables in Table 1 in S17 Table.

**Table 1:** Correlates of WBM.

Variable^a^	Definition	Theoretical Explanation	Measurement • Source • Example Item • Response Scale^b^ • Reliabilities and/or Expert Agreement^c^
Correlates Derived From Theories on Stereotypes			
Competence (F, C)	Having diligence and conscientiousness	Stereotypes about larger-bodied people being less competent indicates they are capable of self-regulation and thus more deserving of WBM	• Single item based on Cuddy et al. [37] • *Would you describe [yourself/others/the person who sent you the survey] as competent, confident, independent, competitive, and intelligent?* • 1-5 SD/SA • r_wg_ = 0.83
Warmth (F, C)	Being easy-going and easy to engage with	Stereotypes about larger-bodied people being less warm indicates they are selfish and thus more deserving of WBM	• Single item based on Cuddy et al. [37] • *Would you describe [yourself/others/the person who sent you the survey] as tolerant, warm, good-natured, and sincere?* • 1-5 SD/SA • r_wg_ = 1.00
Correlates Derived From the Justification-Suppression Model of Prejudice			
*Interpersonal Climate*			
Civility climate (C)	Environment that censures disrespectful, rude, or inappropriate behavior	Work climates that convey disrespect, low concern for others, lack of support, and/or aggressive or mean-spirited interactions convey that WBM of peers is acceptable and thus justifiable	• Complete 4-item scale [45] • *Rude behavior is not accepted by my coworkers*. • 1-5 SD/SA • ⍺ = 0.87; ω_t_ = 0.91
Inclusivity climate: three dimensions of equitable employment practices, integration of differences, inclusion in decision-making (C)	Environment that acknowledges, respects, and values diverse people and perspectives		• Top loading 15-items from across the measure’s three dimensions: equitable employment practices, integration of differences, and inclusion of decision-making [47] • *The performance review process is fair in my work unit., My work unit has a culture in which employees appreciate the differences that bring people to the workplace., In my work unit, employee input is actively sought*. • 1-5 SD/SA • ⍺ = 0.90, 0.89, 0.92; ω_t_ = 0.93, 0.92, 0.94
Interpersonal conflict (C)	Environment in which aggressiveness with others over conflicts is common		• Complete 4-item scale [85] • *How often do you get into arguments with others at work?* • 1-5 N/VO • ⍺ = 0.87; ω_t_ = 0.89
Leader-member exchange (C)	Extent to which someone feels cared for and valued by their boss		• 3 items from affect and 2 items from contribution dimensions were only considered as they reflect, specifically, the emotional climate between a boss and direct report [86] • *My supervisor is a lot of fun to work with.* • 1-5 SD/SA • ⍺ = 0.90; ω_t_ = 0.95
Perceived organizational support (C)	Extent to which someone feels cared for by their organization		• Top three loading items used [87] • *My department or work unit really cares about my well-being.* • 1-5 SD/SA • ⍺ = 0.85; ω_t_ = 0.84
*Weight Control Climate*			
Climate for exercise (C)	Environment in which exercise is commonly discussed	Work climates with more pervasive negative talk about the weight of self and others reinforce judgment that justifies WBM	• Complete 7-item scale [88] • *In my work unit, there are plenty of opportunities to talk about exercise and physical activities.* • 1-5 SD-SA • ⍺ = 0.87; ω_t_ = 0.92
Pressure for thinness (C)	Environment in which it is common to pressure others to be thin		• 4 top loading items representing thin ideal and pressure to be thin from peers [89] • *My coworkers pressure each other to look in better shape.* • 1-5 N/A • ⍺ = 0.91; ω_t_ = 0.93
Tolerance for weight-based discussion (C)	Environment that accepts negative talk about being heavier		• 5 items developed • *Consider managers at work, including your own. How often do they tolerate the below behavior? Talk about not liking larger-bodied people much.* • 1-5 DNT/TC • r_wg(j)_ = 0.99; ⍺ = 0.93; ω_t_ = 0.95
Weight maintenance: organizational support, diet norms, social support, belittling higher weights (C)	Environment in which maintaining a healthy body weight is discussed		• Complete 12-item scale used across four dimensions (evaluated separately) of organizational support, diet norms, social support, belittling higher weight [90] • *My coworkers often encourage healthy food choices.* • 1-5 SD-SA • ⍺ = 0.90, 0.65, 0.82, 0.75; ω_t_ = 0.89, 0.64, 0.79, 0.75
*Human Resources Policies*			
HR messaging related to exercise, weight-loss, and health (C)	*See item*	Weight-focused content and directives from HR normalize negative talk about body weight, providing pretext for WBM	• 3 items developed • *To what extent does Human Resources have programs about [exercise; weight loss; maintaining health, evaluated separately] that are communicated to us often?* • 1-4 N/M • ⍺ = 0.97; ω_t_ = 0.96
HR programming related to exercise, weight-loss, and health (C)	*See item*		• 3 items developed • *To what extent does Human Resources have formal programs that focus on [increasing exercise; weight loss; maintaining health, evaluated separately]?* • 1-4 N/M • ⍺ = 0.94; ω_t_ = 0.93
*Social Status*			
Accent (F)	*See item*	Additional “out-group” indicators that indicate lower social status or social power of the target make it easier to justify WBM.	• Single item developed • *I have an accent that is different from my work colleagues.* • 1-5 SD-SA
BMI (F)	*See item*		• Calculated as a ratio of height to weight [(weight in pounds * 703) / (height in inches)2]
Education relative to others at work (F)	*See item*		• Single item developed • *In the organization where the bullying took place, what is the general level of education of your co-workers as compared to you?* •1-7 MH/ML
Ethnic name (F)	*See item*		• Single item developed • *I have a name that has a strong ethnic connotation.* • 1-5 SD-SA
Gender relative to others at work (F)	*See item*		• Single item developed • *10% increments comparing number of males to females (100% = all female)*
Managerial status (F)	*See item*		• Single item developed • *Are you a manager?*
Visible disability (F)	*See item*		• Single item developed • *I have a disability that is visible (e.g., hearing aid).* • 1-5 SD-SA
Weight relative to others at work (F)	*See item*		• Single item developed • *In the organization where the bullying took place, how would you rate your weight in comparison to your average co-worker?* • 1-7 MH/ML
*Identity Management Strategies*			
Identity redefinition – reassociation (F)	Re-frame attributes of stigmatized feature (e.g., “I’m not fat, I’m curvy”) [58]		• 3 items developed [58] • *I consider my larger body to be powerful*. • 1-5 SD/SA • r_wg(j)_ = 1.00; ⍺ = 0.82; ω_t_ = 0.82
Identity redefinition – regenerate (F)	Tout positive views of the stigmatized characteristic (e.g., “fat is beautiful”) [58]		• 3 items developed; *I affirm to myself the beauty of all body types.* • 1-5 SD/SA [58] • r_wg(j)_ = 0.94; ⍺ = 0.63; ω_t_ = 0.63
Identity switching – deemphasize (F)	Conceal or otherwise do not discuss a stigmatized identity you possess (e.g., do not talk about what you like eat at work) [58]	Helps target divert attention from their size, suppressing weight-based stigma that primes WBM	• 3 items developed [58] • *I deflect attention from my physical appearance*. • 1-5 SD/SA • r_wg(j)_ = 0.81; ⍺ = 0.62; ω_t_ = 0.66
Identity switching – emphasize (F)	Talk-up a non-stigmatized identity you possess (e.g., emphasize your role as mother; baseball coach) [58]		• 3 items developed [58] • *Often, I focus on other positive aspects of who I am as a person, for example: friend; mentor; mother; brother; volunteer*. • 1-5 SD/SA • r_wg(j)_ = 1.00; ⍺ = 0.92; ω_t_ = 0.91
Correlates Derived From Objectification Theory			
*Objectification of Weight by the Self*			
Anxiety (F)	Feeling emotionally unsettled	When the target self-objectifies and believes they do not measure up and/or they are unacceptable just as they are, this weakened state leaves them vulnerable to WBM	• Top four loading items used [91] • *I feel nervous or shaky inside.* • 1-5 SD/SA • ⍺ = 0.92; ω_t_ = 0.94
Body dissatisfaction (actual minus ideal) (F)	Satisfaction with appearance relative to norms [92]		• Assessed using two pictures of a spectrum of body sizes, labeled as one (smallest) through nine (largest); respondents indicated which best represents their current size, and which represents their ideal size [93]
Body trust (F)	Ability to recognize/listen to body hunger and satiety cues [94,95]		• Top three loading items used on the Reliance on Internal Hunger/Satiety Cues of the Intuitive Eating Scale [96] • *I trust my body to tell me when to eat.* • 1-5 SD/SA ⍺ = 0.92; ω_t_ = 0.91
Core self-evaluation (F)	Feeling of esteem, confidence, emotional stability, and efficacy		• Complete 12-item scale used [97] • *I am filled with doubts about my competence.* • 1-5 SD/SA • ⍺ = 0.88; ω_t_ = 0.91
Depression (F)	Feeling a low mood about oneself		• Top three loading items used [91] • *I feel worthless*. • 1-5 SD/SA • ⍺ = 0.92; ω_t_ = 0.94
Guilt (F)	Feeling wrong due to behaviors enacted		• Top three loading items used [98] • *I feel delinquent.* • 1-5 SD/SA • ⍺ = 0.86; ω_t_ = 0.84
Objectified body consciousness (F)	Feeling personally liable for your size (e.g., [99])		• Top three loading items used [100] • *I think a person’s weight is mostly determined by the genes they were born with.* • 1-5 SD/SA • ⍺ = 0.74; ω_t_ = 0.75
Shame (F)	Feeling wrong fundamentally as a person		• Top three loading items of the inadequacy sub-dimension [101] • *I think that people look down on me.* • 1-5 SD/SA • ⍺ = 0.81; ω_t_ = 0.79
Weight-based bias internalization (F)	Internalized belief that larger-bodied people deserve marginalization		• Complete 11-item scale used [41] • *I feel anxious about being overweight because of what people might think of me.* • 1-5 SD/SA • ⍺ = 0.90; ω_t_ = 0.92
*Objectification of Weight by Others*			
Anti-fat attitudes (C)	Environment that believes being larger sized is inherently wrong	Objectification of the target intensifies when there are stronger beliefs about body size norms; the judgment intrinsic to objectification cues WBM	• 3 top-loading items across the measure’s three dimensions (dislike, fear of fat, and willpower) of the anti-fat attitudes scale [38] • *At work, people tend to think that people who are overweight are not trustworthy.* • 1-5 N/A • ⍺ = 0.89, 0.83, 0.90; ω_t_ = 0.88, 0.81, 0.89
Negative talk about the weight of oneself (C)	Environment in which negative talk about one’s own weight is common		• Complete three-item scale; referent shift to work context [102] • *How often do people at work talk negatively about their own bodies?* • 1-5 N/A • r_wg(j)_ = 0.95; ⍺ = 0.90; ω_t_ = 0.89
Negative talk about the weight of others (C)	Environment in which negative talk about other’s weight is common		• Complete three-item scale; referent shift to work context and about others [102] • *How often do people at work talk negatively about other people’s bodies?* • 1-5 N/A • r_wg(j)_ = 0.95; ⍺ = 0.95; ω_t_ = 0.94
Thin ideal (C)	Environment that idolizes being thin		• Top loading item from each of these dimensions of the thin ideal scale: information, pressures, and internationalization-general [103] • *My coworkers compare their appearance to the appearance of TV and movie stars.* • 1-5 N/A • ⍺ = 0.90; ω_t_ = 0.89
Correlates Derived From Task, Social, and Physical Occupational Contexts			
Task (O)	Importance of task considerations of an occupation	Higher ratings of occupational attributes typically associating success with being slimmer prime condemnation of larger-bodied people	• 14 ratings; *Rate the duration of the typical work week.* • 1-5 scale; anchors dependent on item
Social (O)	Importance of social considerations of an occupation		• 11 ratings; *Rate the importance of work with a group or team.* • 1-5 scale; anchors dependent on item
Physical (O)	Importance of physical considerations of an occupation		• 30 ratings; *Rate the frequency of repetitive motions.* • 1-5 scale; anchors dependent on item

a F = rated by focal respondent; C = rated by focal respondent’s co-worker; O = O*NET ratings.

b Likert response scales: 1–5 SD/SA:1 = strongly disagree to 5 = strongly agree; 1–3 N/A: 1 = Never, 2 = Often, and 3 = Always; 1–5 NAA/AGD:1 = Not at all to 5 = A great deal; 1–7 MH/ML: 1 = much higher to 7 = much lower; 1–4 N/M:1 = none to 4 = many; 1–5 DNT-TC: 1 = do not tolerate to 5 = tolerates completely; 1–5 N/A:1 = never to 5 = always; 1–5 N/VO: 1 = never to 5 = very often.

c r_wg_ = single item expert agreement; r_wg(j)_ = multi-item expert agreement; α = Cronbach’s alpha; ω_t_ = omega-total.

### Reflexivity statement

Before describing results and their interpretation, we offer a reflexivity statement so that the reader better understands how the research team’s backgrounds may have influenced the design of the study and evaluation of its results. This study’s authors have differing relationships with their body weight and different experiences with how they have been treated socially as it relates to their body size. Two study authors are of “normal body weight” and have not experienced significant weight deviations in their life, nor have they experienced denigration of their appearance based on body weight. One study author experienced moderate weight fluctuations, and when at a greater weight, experienced some notable shaming about their body size but reports coping swiftly and effectively with that commentary. The remaining author has lived significant portions of their life in four body mass index classifications, with observers likely labeling them close to a Western “thin ideal” at times, and at other times, extraordinarily deviating from it. They report significantly different treatment personally and professionally across these weight categories, including bullying and ostracization at higher weights and adulation and privilege at lower weights. We report this author’s influence using the GRIPP2 short form [104]; this author’s experiences influenced the *study’s overall aim* insofar as the author was one of the study’s initial progenitors, the *methods* insofar as the author described methods most likely to sensitively ask about body-focused mistreatment, *interpretation of results* insofar as the author was able to contextualize for the research team why some associations might be expected (e.g., that even people at higher body weights can often negatively judge toward *others* in higher body weights – that is, internalized weight bias [105,106]), *discussion and conclusions* in that the author was able to communicate more clearly about patterns in the data because they *a priori* understood affinities between contextual correlates of antifat bias (e.g., why seemingly harmless talk about dieting actually can create a context for WBM), and finally, *critical perspectiv*e in that the author was able to frame, in particular, practical recommendations that would not make the author feel singled-out for their body size. That said, such involvement was counterbalanced by the remaining authors who were able to sensitively review design and framing choices to ensure that theory and data spoke for the lived experiences of many larger-bodied people not just one author’s experience. All authors hold a Ph.D. in work psychology or a related field.

## Results

### Analytical approach

During this study’s inception, we sought to create a comprehensive list of constructs we thought would predict WBM at work through careful review of many literatures that speak to body weight stigmatization. Because our study design precludes causal testing, we consider these variables as correlates. To understand the relations of these correlates to WBM, we take an exploratory, descriptive analytical approach as, in our case, prior to collecting data, we did not precisely define our population of larger-bodied individuals (e.g., all larger-bodied people in a country; all larger-bodied people with Amazon Mechanical Turk accounts; etc.) nor did we randomly sample from any possibly defined population. Experimental and survey research in the social sciences commonly follows a similar convenience sample data collection procedure. In such cases, researchers cannot generalize their results to populations. Instead, researchers can only report findings for their specific sample (e.g., describing the calculated relationships between variables for a specific sample of entities measured at a specific time). Importantly, in such cases, analytics that by “default” produce inferential probabilities, like the regression models we use, does not imply that researchers should “automatically” make utility assessments based on those inferential probabilities. For research that cannot generalize its results beyond a specific sample such as ours, those inferential probabilities hold questionable value. Instead, researchers should focus on using calculated relationships (e.g., like the regression coefficients and dominance weights we compute) to provide a description of the relationships between predictors and outcomes for a specific sample at a specific time. We do report frequentist probabilities or confidence intervals to match traditional reporting standards in social science research. Yet, rather than applying a “generalizability” mindset for this study by referencing reported frequentist probabilities, we encourage readers to apply a “descriptive” mindset when reviewing the results from the analyses applied to our specific sample; assessing the generalizability of our results requires additional research on new samples. To simplify reporting, we report results based on listwise deletion of cases, which resulted in minimal loss of data for seven research questions (i.e., RQ 1–3, 6–9 result in less than 3% data loss) and moderate loss of data for two research questions (i.e., RQ 4–5 result in about 16–23% data loss). The general pattern of results did not change meaningfully for the two research questions with moderate loss of data when imputing missing data using multivariate imputation by chained equations [107].

### Discrete results

We present the results for each of the nine research questions. For each research question, we conducted three analyses. The first analysis shows Cohen’s standardized mean difference on each correlational measure for our larger-bodied respondents who experienced WBM in the past six months versus those who never experienced WBM. These standardized mean differences provide a zero-order bivariate relationship between experiencing WBM and the correlational measures. The second analysis shows the results from a logistic regression model and subsequent dominance analysis of predicting the binary outcome of either experiencing or not experiencing WBM from the correlational measures. The logistic regression provides an estimate of the overall effect size of the correlational measures on predicting the binary outcome via Tjur’s R^2^ (total discrimination) metric (i.e., absolute difference between the mean of the probabilities for predicting membership into each of the two groups) (see Tjur [108] for more details). The dominance analysis decomposes Tjur’s R^2^ metric into an R^2^ value for each individual correlational measure to evaluate the strength of each correlational measure in predicting the binary outcome in the presence of multicollinearity among correlational measures. Dominance analysis works by estimating all possible subsets of the complete regression model, which ultimately results in *2*^*p*^ regression models with *p* equal to the number of predictors (see Azen and Budescu [109], Budescu [110], and Budescu and Azen [111] for more information on dominance analysis). The third analysis shows the results from an ordinary least-squares (OLS) regression model and subsequent dominance analysis of predicting overall WBM rate for those who experienced WBM in the past six months from the correlational measures. Like logistic regression, OLS regression provides a well-known estimate of the overall size of the correlational measures on predicting the overall rate of WBM via the ordinary R^2^ metric. The dominance analysis decomposes the ordinary R^2^ metric into an R^2^ value for each individual correlational measure to evaluate the strength of each correlational measure in predicting the outcome. Again, we emphasize that our results apply to just this sample due to our data collection methodology, and we use the specified regression models to describe just this sample.

**Fig 1:**
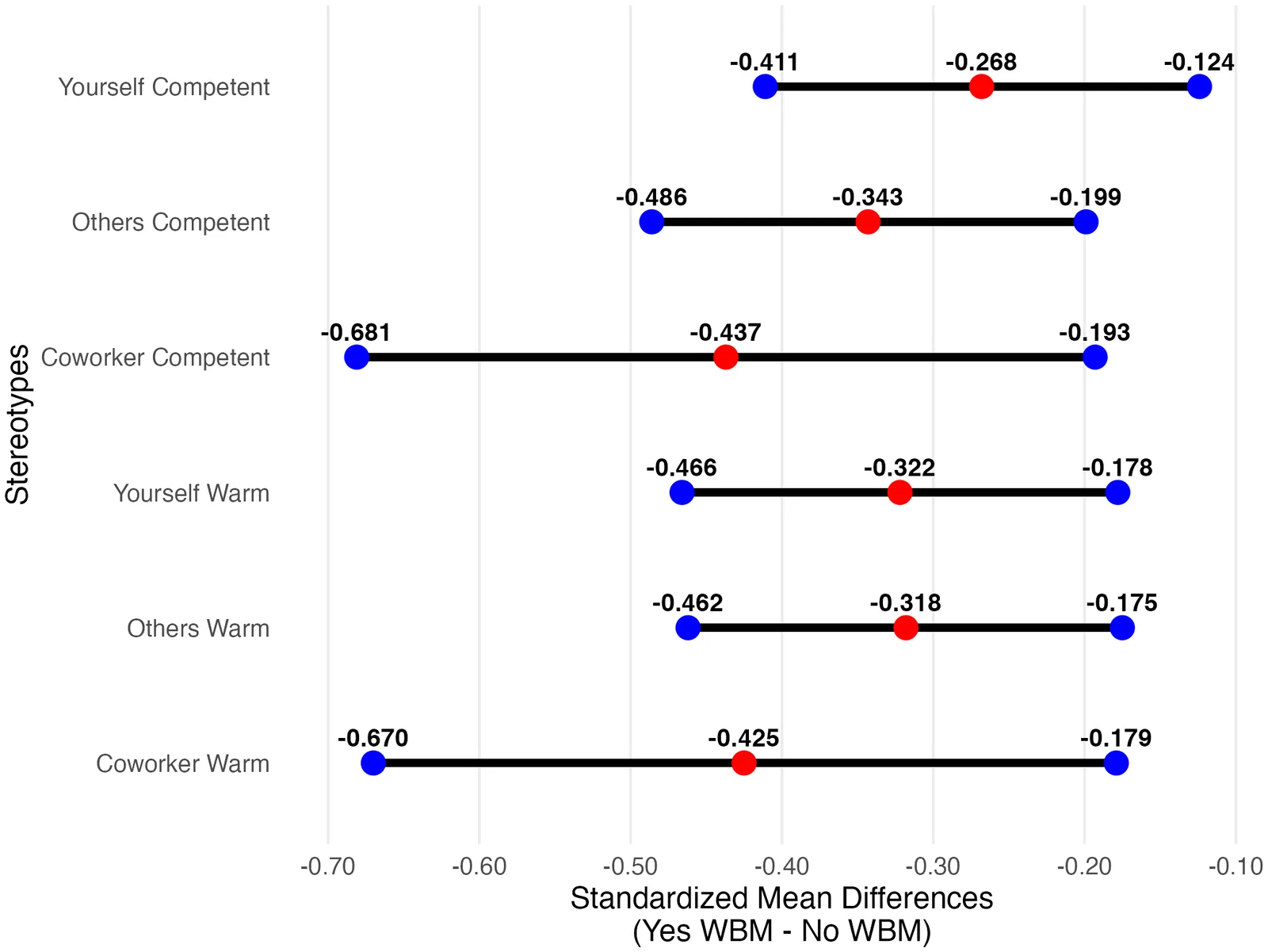
Standardized mean differences with 95% confidence intervals on stereotypes for “Yes” Minus “No” WBM (RQ1).

The first research question asked how perceptions of the stereotyped attributes of competence and warmth, as rated by focal employees and coworkers, relate to WBM. In Fig 1, the standardized mean differences indicate lower ratings of competence and warmth for those focal employees who experienced WBM in the past six months as opposed to those focal employees who did not experience WBM. In Tables 2 and 3, these six measures of stereotypes account for 5.2% of the total discrimination in experiencing WBM and 8.9% of the total variance in the composite WBM rate. In terms of which measures influence WBM most strongly, the dominance analysis suggests ratings of warmth accounts for most of the fitted differences between larger-bodied respondents who did and did not experience WBM (general dominance [GD] = 3.0%; relative general dominance [RGD] = 56.3%) whereas ratings of competence account for most of the fitted variance in the composite WBM rate among just those respondents who reported mistreatment (GD = 6.6%; RGD = 75.3%). Furthermore, the ratings of competence and warmth provided by coworkers account for most of the fitted differences between larger-bodied respondents who did versus did not experience WBM at work (GD = 4.2%; RGD = 80.1%) and fitted variance in the composite WBM rate (GD = 5.8%; RGD = 65.5%). To summarize the key findings for RQ1: those individuals who experienced WBM received lower self, other, and coworker ratings of competence and warmth compared to those individuals who did not experience WBM; how coworkers view an individual’s warmth most strongly relates to whether those individuals experienced WBM; how coworkers view the competence of larger-bodied individuals most strongly associates with the rate of experiencing WBM.

**Table 2:** Logistic regression and dominance analysis for stereotypes on binary WBM (RQ1).

	GD	RGD	B	SE	t	p
Intercept			5.348	1.186	4.508	< 0.001
Yourself Competent	0.1%	1.6%	−0.076	0.152	−0.501	0.617
Others Competent	0.2%	4.0%	0.092	0.197	0.465	0.642
Coworker Competent	2.0%	38.1%	−0.444	0.232	−1.917	0.055
Yourself Warm	0.4%	7.3%	0.287	0.178	1.609	0.108
Others Warm	0.4%	7.0%	−0.274	0.224	−1.222	0.222
Coworker Warm	2.2%	42.0%	−0.485	0.253	−1.913	0.056
Total R^2^	5.2%					
Sample Size	376					
Listwise Deletion	7					

GD = General Dominance; RGD = Relative General Dominance; B = Unstandardized Regression Coefficient; SE = Standard Error; t = t-test value; p = frequentist probability value. General dominance percentages sum to the total R² percentage; relative general dominance percentages sum to 100%.

**Table 3:** Ordinary least-squares regression and dominance analysis for stereotypes on composite WBM rate (RQ1).

	GD	RGD	B	SE	t	p
Intercept			1.816	0.107	16.931	< 0.001
Yourself Competent	1.3%	15.1%	−0.025	0.018	−1.375	0.170
Others Competent	1.4%	16.2%	−0.018	0.022	−0.805	0.422
Coworker Competent	3.9%	44.0%	−0.053	0.023	−2.334	0.020
Yourself Warm	0.1%	1.5%	0.012	0.023	0.524	0.601
Others Warm	0.1%	1.7%	0.005	0.026	0.181	0.857
Coworker Warm	1.9%	21.5%	−0.031	0.025	−1.224	0.222
Total R^2^	8.9%					
Sample Size	293					
Listwise Deletion	6					

Same notes as Table 2.

**Fig 2:**
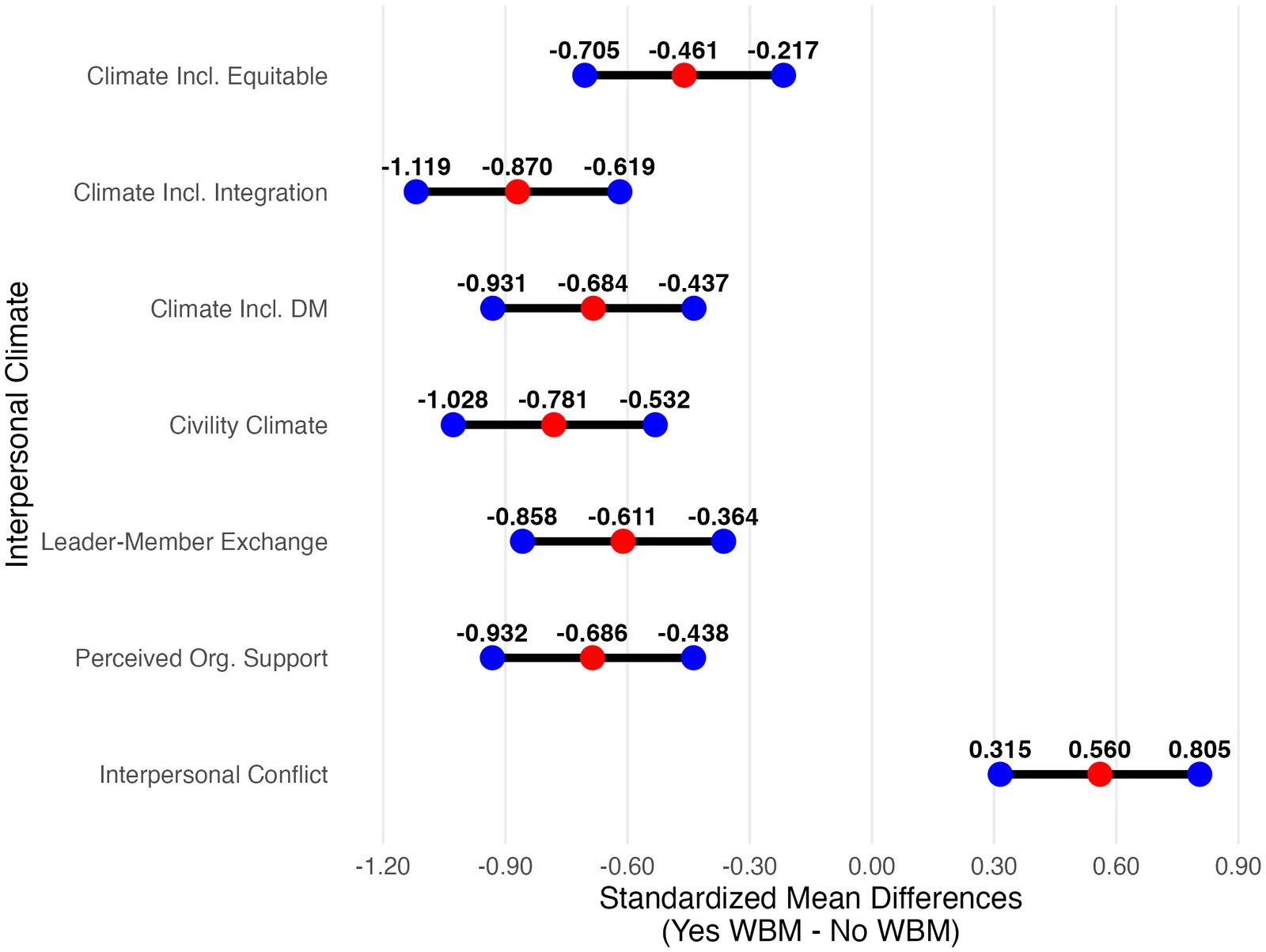
Standardized mean differences with 95% confidence intervals on interpersonal climate for “Yes” Minus “No” WBM (RQ2). Incl. = Inclusivity; DM = Decision-Making.

The second research question asked if interpersonal climates associate with WBM. In Fig 2, the standardized mean differences indicate lower ratings of various kinds of climate inclusivity and civility, leader relations, and perceived organizational support while higher ratings of interpersonal conflict for those focal employees who experienced WBM in the past six months as opposed to those focal employees who did not experience WBM. In Tables 4 and 5, these seven measures of interpersonal climate account for 17.1% of the total discrimination in experiencing WBM and 19.1% of the total variance in the composite WBM rate. Notably, the dominance analysis suggests a general climate for civility and a specific climate of integration account for more than half of the fitted discrimination in experiencing WBM (GD = 9.4%; RGD = 54.8%) whereas interpersonal conflict on its own accounts for more than half of the fitted variance in the composite WBM rate (GD = 10.2%; RGD = 53.1%). To summarize the key findings for RQ2: those individuals who experienced WBM suffered from moderately worse interpersonal climates compared to those individuals who did not experience WBM; the extent to which individuals experienced an integrated and civil climate related to whether individuals experienced WBM; the extent to which larger-bodied individuals experienced interpersonal conflict (e.g., rudeness, argumentativeness) most strongly associates with the rate of experiencing WBM.

**Table 4:** Logistic regression and dominance analysis for interpersonal climate on binary WBM (RQ2).

	GD	RGD	B	SE	t	p
Intercept			5.434	1.171	4.639	< 0.001
Climate Incl. Equitable	1.4%	8.4%	0.599	0.230	2.605	0.009
Climate Incl. Integration	5.6%	32.9%	−1.211	0.323	−3.744	< 0.001
Climate Incl. DM	1.9%	10.9%	−0.057	0.246	−0.233	0.816
Civility Climate	3.8%	21.9%	−0.377	0.203	−1.859	0.063
Leader-Member Exchange	1.1%	6.7%	−0.083	0.209	−0.398	0.691
Perceived Org. Support	1.8%	10.3%	−0.059	0.221	−0.266	0.790
Interpersonal Conflict	1.5%	8.9%	0.194	0.234	0.829	0.407
Total R^2^	17.1%					
Sample Size	382					
Listwise Deletion	1					

Same notes as Table 2; Incl. = Inclusivity; DM = Decision-Making.

**Table 5:** Ordinary least-squares regression and dominance analysis for interpersonal climate on composite WBM rate (RQ2).

	GD	RGD	B	SE	t	p
Intercept			1.317	0.102	12.895	< 0.001
Climate Incl. Equitable	1.0%	5.3%	−0.010	0.023	−0.453	0.651
Climate Incl. Integration	2.1%	11.2%	−0.051	0.028	−1.792	0.074
Climate Incl. DM	0.9%	4.5%	0.027	0.024	1.144	0.254
Civility Climate	1.9%	9.9%	−0.009	0.022	−0.405	0.686
Leader-Member Exchange	1.8%	9.2%	−0.022	0.021	−1.048	0.295
Perceived Org. Support	1.3%	6.8%	0.006	0.023	0.270	0.787
Interpersonal Conflict	10.2%	53.1%	0.119	0.022	5.419	< 0.001
Total R^2^	19.1%					
Sample Size	299					
Listwise Deletion	0					

Same notes as Table 4.

**Fig 3:**
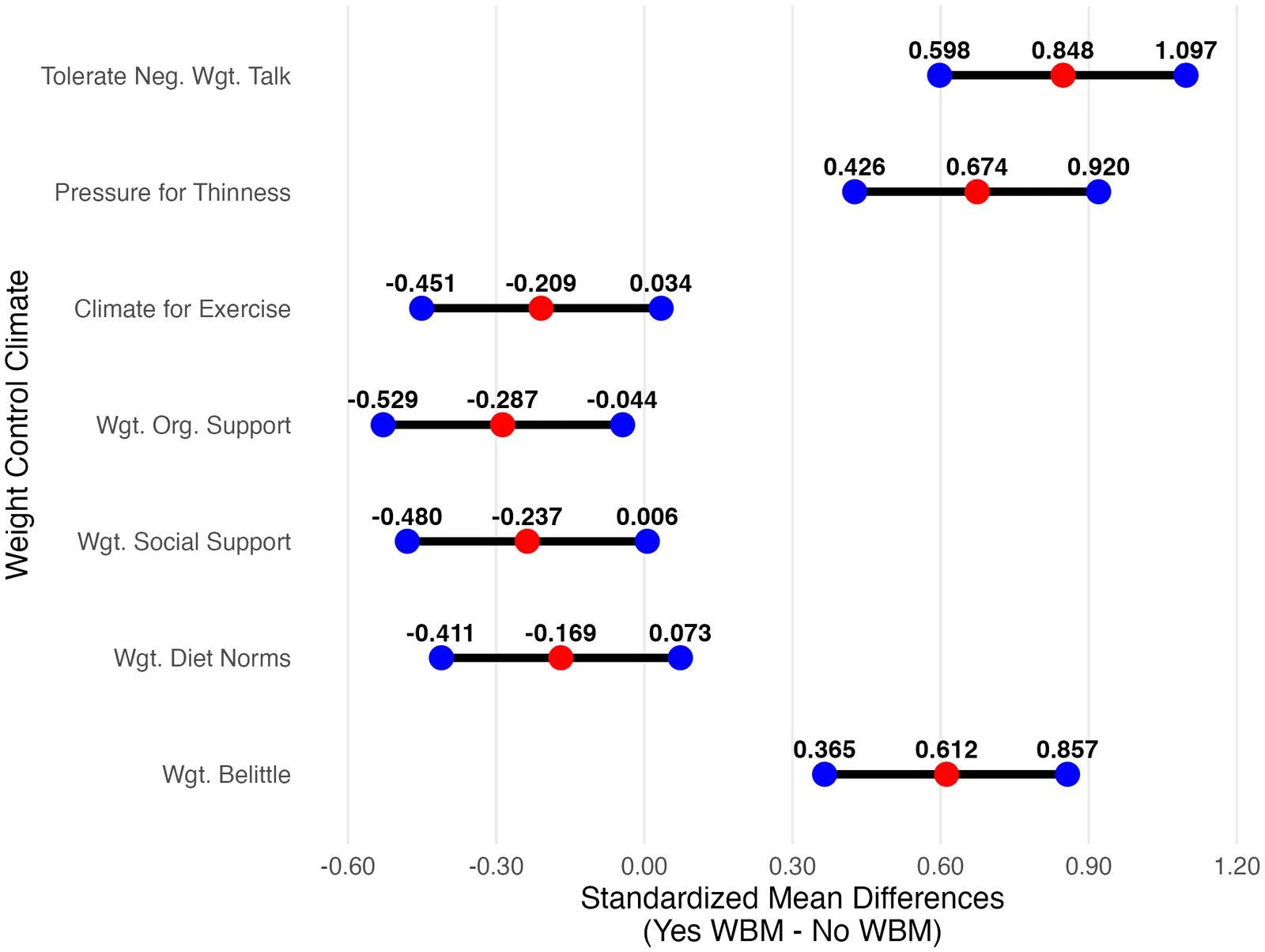
Standardized mean differences with 95% confidence intervals on weight control climate for “Yes” Minus “No” WBM (RQ3). Neg. = Negative; Wgt. = Weight; Org. = Organizational.

The third research question asked how the climate related to weight control associates with WBM. In Fig 3, the standardized mean differences indicate moderately higher ratings of tolerating negative talk about weight, pressure for thinness, and belittling weight loss attempts for those focal employees who experienced WBM in the past six months as opposed to those focal employees who did not experience WBM. In Tables 6 and 7, these seven measures of weight control climate account for 19.6% of the total discrimination in experiencing WBM and 16.2% of the total variance in the composite WBM rate. The dominance analysis suggests tolerance for negative talk about weight, pressure for thinness, and belittling weight loss attempts account for most of the fitted discrimination in experiencing WBM (GD = 16.9%; RGD = 86.0%) and most of the fitted variance in the composite WBM rate (GD = 14.5%; RGD = 89.6%). To summarize the key findings for RQ3: the extent to which individuals experienced cultures that tolerate negative talk about weight, pressure individuals to become thin, and belittle weight loss attempts related to the probability of individuals experiencing WBM and the rate of experiencing WBM to moderate degree.

**Table 6:** Logistic regression and dominance analysis for weight control climate on binary WBM (RQ3).

	GD	RGD	B	SE	t	p
Intercept			−0.365	0.615	−0.594	0.553
Tolerate Neg. Wgt. Talk	7.1%	36.2%	0.466	0.160	2.906	0.004
Pressure for Thinness	6.9%	35.2%	0.818	0.204	4.003	< 0.001
Climate for Exercise	0.6%	3.1%	−0.248	0.259	−0.959	0.338
Wgt. Org. Support	0.7%	3.4%	−0.070	0.198	−0.353	0.724
Wgt. Social Support	1.3%	6.5%	−0.483	0.232	−2.084	0.037
Wgt. Diet Norms	0.2%	1.1%	0.140	0.219	0.640	0.522
Wgt. Belittle	2.9%	14.6%	0.261	0.152	1.719	0.086
Total R^2^	19.6%					
Sample Size	383					
Listwise Deletion	0					

Same notes as Table 2; Neg. = Negative; Wgt. = Weight; Org. = Organizational.

**Table 7:** Ordinary least-squares regression and dominance analysis for weight control climate on composite WBM rate (RQ3).

	GD	RGD	B	SE	t	p
Intercept			0.940	0.074	12.670	< 0.001
Tolerate Neg. Wgt. Talk	8.6%	52.9%	0.070	0.016	4.352	< 0.001
Pressure for Thinness	3.0%	18.8%	0.032	0.019	1.649	0.100
Climate for Exercise	0.2%	1.5%	0.010	0.029	0.352	0.725
Wgt. Org. Support	0.1%	0.9%	0.007	0.024	0.318	0.751
Wgt. Social Support	0.4%	2.7%	−0.042	0.024	−1.746	0.082
Wgt. Diet Norms	0.9%	5.4%	0.046	0.025	1.866	0.063
Wgt. Belittle	2.9%	17.9%	0.037	0.017	2.136	0.033
Total R^2^	16.2%					
Sample Size	299					
Listwise Deletion	0					

Same notes as Table 6.

**Fig 4:**
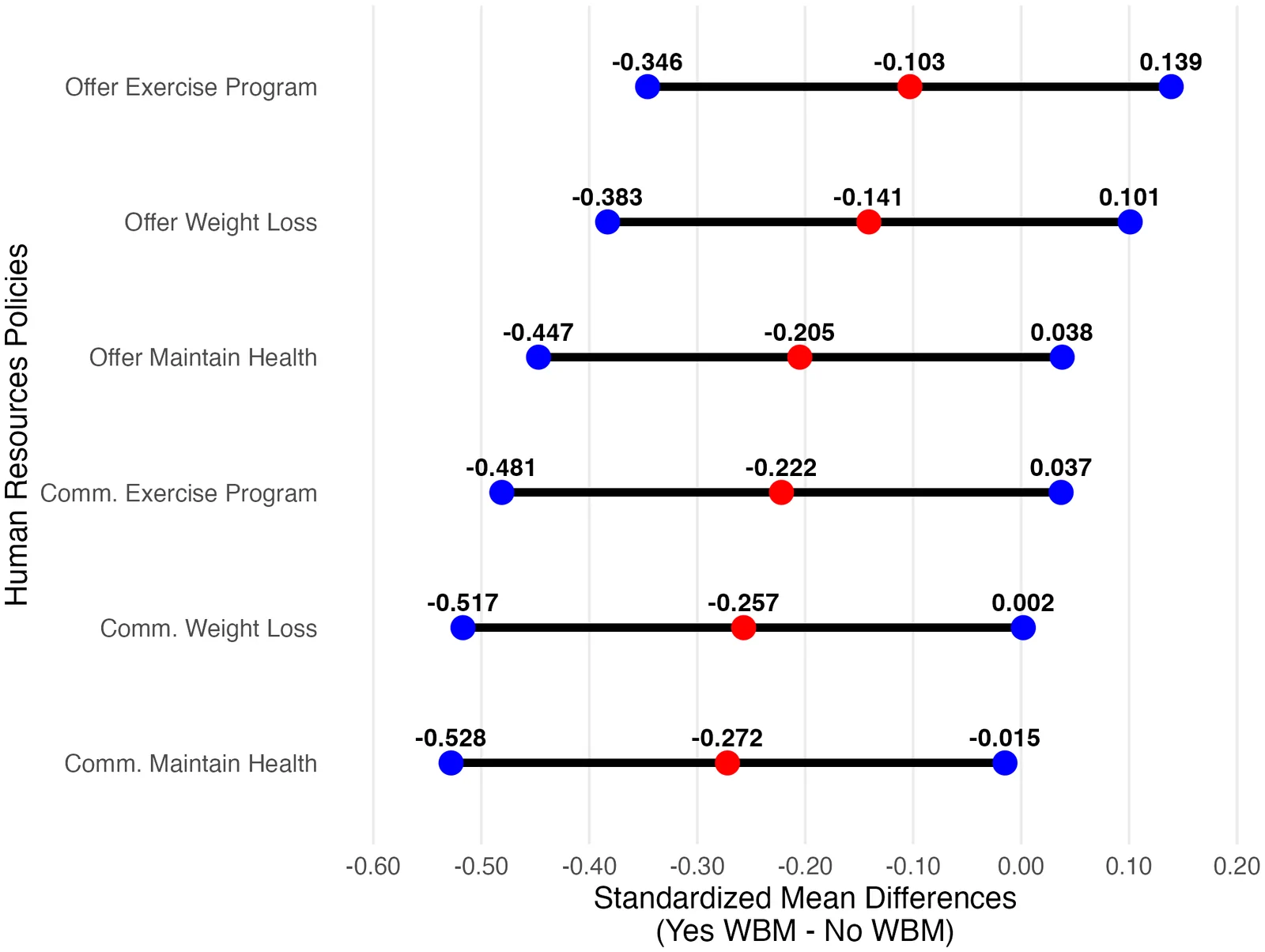
Standardized mean differences with 95% confidence intervals on human resources policies for “Yes” Minus “No” WBM (RQ4). Comm. = Communicate.

The fourth research question asked how human resources policies that invoke health associate with WBM. In Fig 4, the standardized mean differences indicate very small lower ratings of formal programs and communications from the human resources department with respect to exercise, weight loss, and maintaining health for those focal employees who experienced WBM in the past six months as opposed to those focal employees who did not experience WBM. In Tables 8 and 9, these six measures of human resources policies account for 2.2% of the total discrimination in experiencing WBM and 2.2% of the total variance in the composite WBM rate. Hence, as a summary of RQ4, these small total R^2^ values indicate the overall very small association of human resources policies on the probability of experiencing WBM and the rate of experiencing WBM.

**Table 8:** Logistic regression and dominance analysis for human resources polices on binary WBM (RQ4).

	GD	RGD	B	SE	t	p
Intercept			1.627	0.325	5.008	< 0.001
Offer Exercise Program	0.4%	19.5%	0.508	0.393	1.293	0.196
Offer Weight Loss	0.1%	6.5%	−0.054	0.385	−0.141	0.888
Offer Maintain Health	0.5%	22.3%	−0.340	0.322	−1.056	0.291
Comm. Exercise Program	0.4%	17.5%	−0.138	0.266	−0.521	0.602
Comm. Weight Loss	0.3%	14.7%	−0.061	0.275	−0.222	0.824
Comm. Maintain Health	0.4%	19.5%	−0.039	0.222	−0.175	0.861
Total R^2^	2.2%					
Sample Size	322					
Listwise Deletion	61					

Same notes as Table 2; Comm. = Communicate.

**Table 9:** Ordinary least-squares regression and dominance analysis for human resources polices on composite WBM rate (RQ4).

	GD	RGD	B	SE	t	p
Intercept			1.399	0.045	30.748	< 0.001
Offer Exercise Program	0.1%	4.2%	−0.019	0.057	−0.335	0.738
Offer Weight Loss	0.3%	15.5%	0.060	0.057	1.050	0.295
Offer Maintain Health	0.5%	25.0%	−0.051	0.051	−1.010	0.313
Comm. Exercise Program	0.1%	6.0%	0.012	0.038	0.310	0.757
Comm. Weight Loss	0.4%	16.2%	0.037	0.038	0.988	0.324
Comm. Maintain Health	0.7%	33.0%	−0.045	0.031	−1.433	0.153
Total R^2^	2.2%					
Sample Size	249					
Listwise Deletion	50					

Same notes as Table 8.

**Fig 5:**
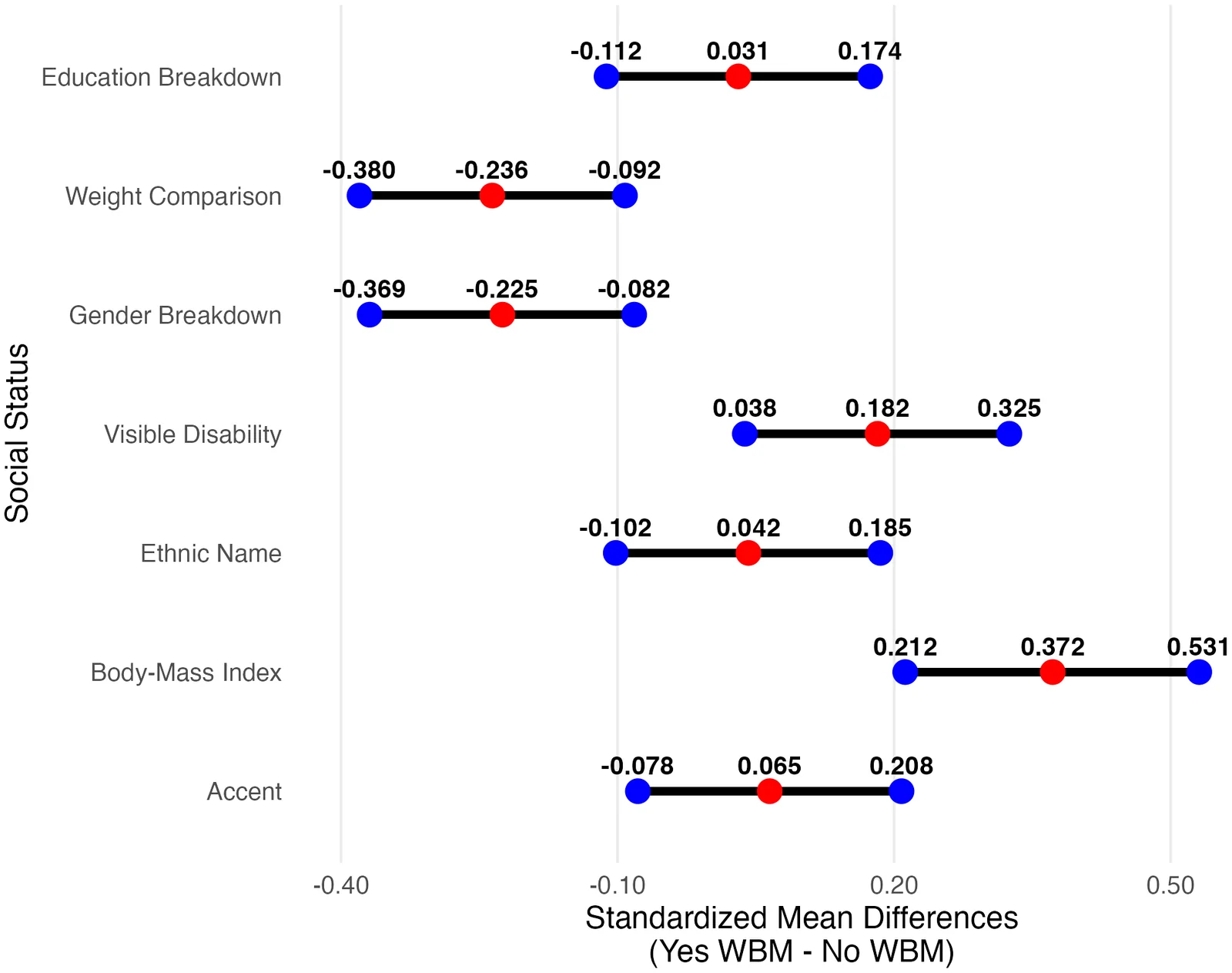
Standardized mean differences with 95% confidence intervals on social status for “Yes” Minus “No” WBM (RQ5).

The fifth research question asked how social status associates with WBM. In Fig 5, the standardized mean differences indicate very small differences with respect to more male composed workplaces, higher weight relative to coworkers, higher rate of possessing a visible disability, and higher body-mass index for those focal employees who experienced WBM in the past six months as opposed to those focal employees who did not experience WBM. In Tables 10 and 11, we include the seven measures of social status from Fig 5 plus binary indicators for manager, gender, and race. Together, these variables account for 6.6% of the total discrimination in experiencing WBM and 17.6% of the total variance in the composite WBM rate. The dominance analysis suggests that the body-mass index, holding a managerial position, and comparative weight account for most of the fitted discrimination in experiencing WBM (GD = 5.0%; RGD = 76.0%) whereas a visible disability, an ethnic name, and an accent account for most of the fitted variance in the composite WBM rate (GD = 12.6%; RGD = 71.3%). To summarize the key findings for RQ5: immediately discernable indicators of social status or social power (i.e., disability, ethnic name, accent, non-white) associate more strongly with a higher rate of experiencing WBM compared to indicators of social status that require some calculation (i.e., relative education).

**Table 10:** Logistic regression and dominance analysis for social status on binary WBM (RQ5).

	GD	RGD	B	SE	t	p
Intercept			−0.183	0.687	−0.266	0.790
Education Breakdown	0.2%	2.5%	0.052	0.072	0.714	0.475
Weight Comparison	1.2%	18.6%	−0.174	0.070	−2.474	0.013
Gender Breakdown	0.4%	6.2%	−0.059	0.035	−1.692	0.091
Manager	1.3%	19.5%	0.562	0.187	3.007	0.003
Visible Disability	0.3%	5.3%	0.169	0.113	1.498	0.134
Ethnic Name	0.0%	0.7%	−0.022	0.092	−0.239	0.811
Body-Mass Index	2.5%	37.9%	0.053	0.013	4.059	< 0.001
Accent	0.0%	0.3%	0.015	0.090	0.168	0.866
Gender	0.1%	1.5%	−0.079	0.178	−0.445	0.656
Race	0.5%	7.6%	−0.473	0.219	−2.158	0.031
Total R^2^	6.6%					
Sample Size	796					
Listwise Deletion	212					

Same notes as Table 2. Gender: Male = 0 and Female = 1; Race: Non-White = 0 and White = 1; Manager: No = 0 and Yes = 1; Gender Breakdown = higher values indicate higher perceived percentage of women in the workplace; Weight Comparison = higher values indicate lower perceived weight of employee relative to coworkers; Education Breakdown = higher values indicate higher perceived education of employee relative to coworkers.

**Table 11:** Ordinary least-squares regression and dominance analysis for social status on composite WBM rate (RQ5).

	GD	RGD	B	SE	t	p
Intercept			1.217	0.094	12.966	< 0.001
Education Breakdown	1.4%	7.7%	−0.029	0.010	−2.873	0.004
Weight Comparison	1.0%	5.5%	−0.029	0.010	−2.781	0.006
Gender Breakdown	0.2%	1.2%	0.007	0.005	1.377	0.169
Manager	1.9%	10.6%	0.086	0.026	3.348	0.001
Visible Disability	4.9%	27.9%	0.062	0.014	4.334	< 0.001
Ethnic Name	3.7%	20.8%	0.040	0.013	3.082	0.002
Body-Mass Index	0.2%	1.4%	0.002	0.002	1.320	0.187
Accent	4.0%	22.6%	0.043	0.013	3.320	0.001
Gender	0.3%	2.0%	0.036	0.025	1.411	0.159
Race	0.1%	0.4%	0.013	0.029	0.433	0.665
Total R^2^	17.6%					
Sample Size	593					
Listwise Deletion	165					

Same notes as Table 10.

**Fig 6:**
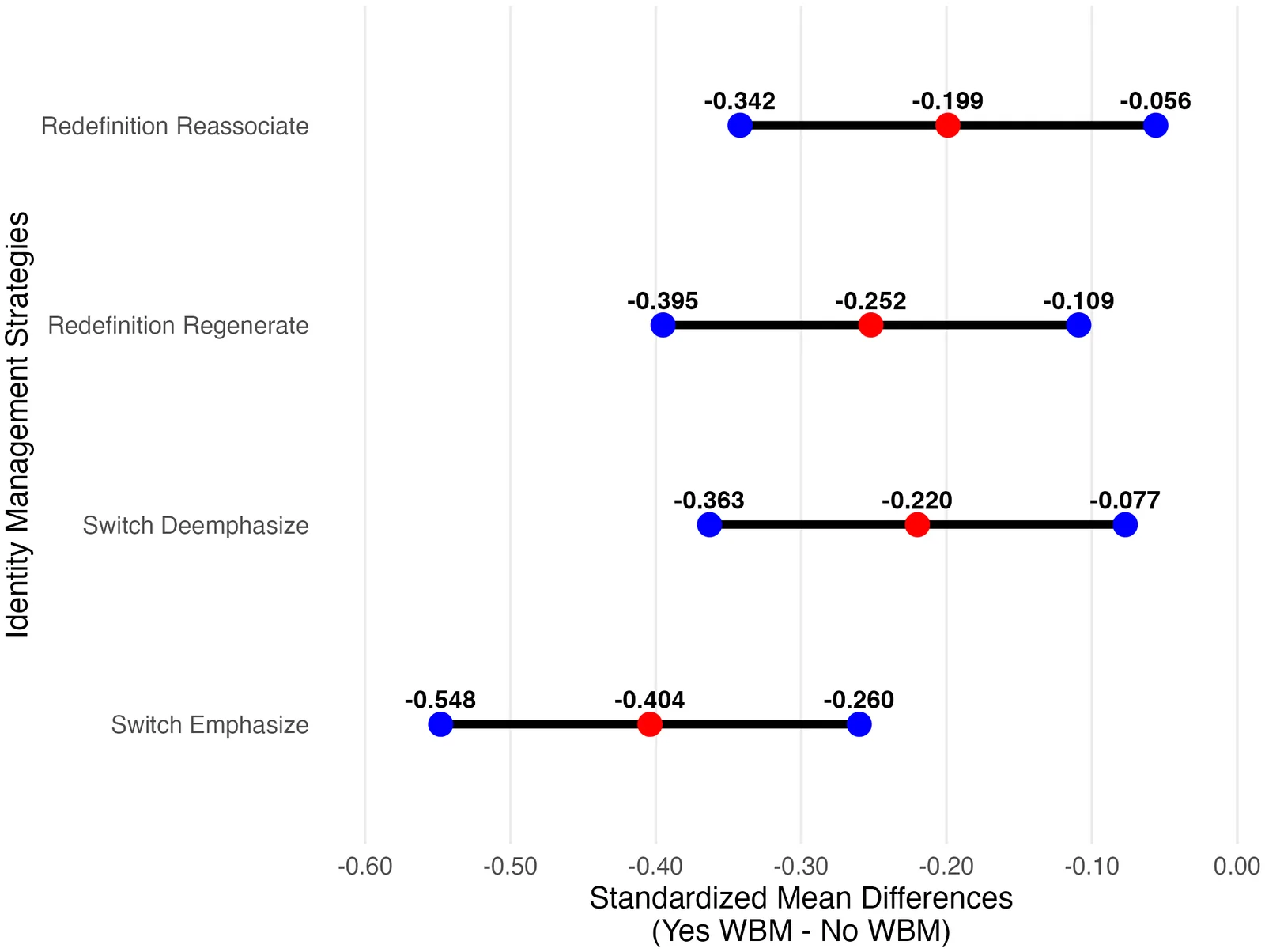
Standardized mean differences with 95% confidence intervals on identity management strategies for “Yes” Minus “No” WBM (RQ6).

The sixth research question asked how identity management strategies associate with WBM. In Fig 6, the standardized mean differences indicate small to medium lower tendency of emphasizing other characteristics for those focal employees who experienced WBM in the past six months as opposed to those focal employees who did not experience WBM. In Tables 12 and 13, these four strategies to reorganize one’s identity relative to weight account for 3.3% of the total discrimination in experiencing WBM and 3.0% of the total variance in the composite WBM rate. Hence, as a summary of RQ6, these small total R^2^ values indicate the overall small association of identity management strategies on the probability of individuals experiencing WBM and the rate of experiencing WBM.

**Table 12:** Logistic regression and dominance analysis for identity management strategies on binary WBM (RQ6).

	GD	RGD	B	SE	t	p
Intercept			2.519	0.343	7.335	< 0.001
Redefinition Reassociate	0.2%	7.3%	−0.044	0.083	−0.535	0.593
Redefinition Regenerate	0.4%	12.2%	−0.076	0.103	−0.740	0.459
Switch Deemphasize	0.4%	11.4%	0.172	0.119	1.447	0.148
Switch Emphasize	2.3%	69.0%	−0.461	0.104	−4.435	< 0.001
Total R^2^	3.3%					
Sample Size	1008					
Listwise Deletion	0					

Same notes as Table 2.

**Table 13:** Ordinary least-squares regression and dominance analysis for identity management strategies on composite WBM rate (RQ6).

	GD	RGD	B	SE	t	p
Intercept			1.389	0.047	29.624	< 0.001
Redefinition Reassociate	0.9%	31.6%	0.028	0.013	2.102	0.036
Redefinition Regenerate	0.9%	30.0%	0.036	0.017	2.185	0.029
Switch Deemphasize	0.1%	4.9%	0.018	0.019	0.947	0.344
Switch Emphasize	1.0%	33.5%	−0.053	0.016	−3.339	0.001
Total R^2^	3.0%					
Sample Size	758					
Listwise Deletion	0					

Same notes as Table 2.

**Fig 7:**
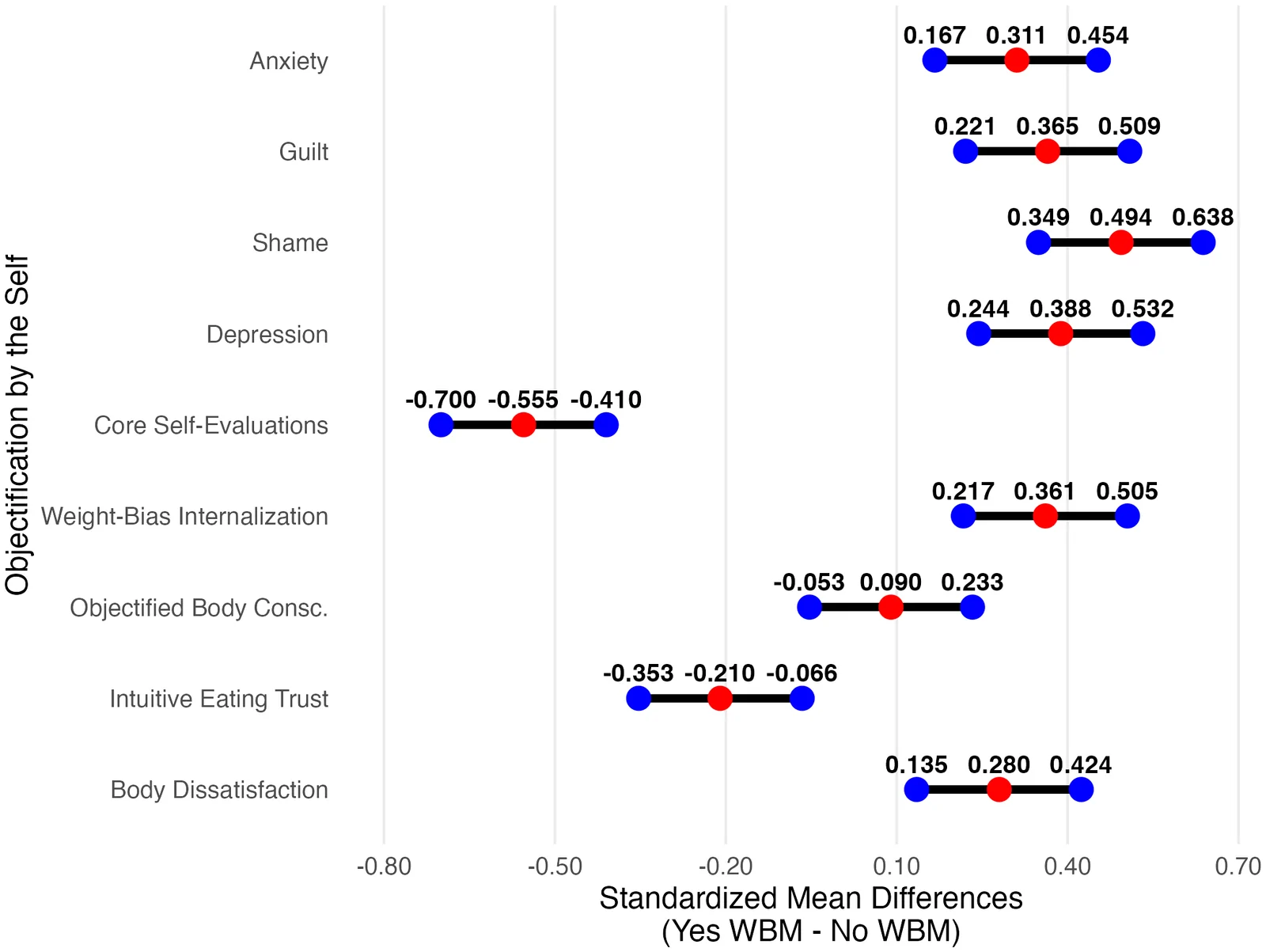
Standardized mean differences with 95% Confidence intervals on objectification of weight by the self for “Yes” Minus “No” WBM (RQ7). Consc. = Consciousness.

The seventh research question asked how objectification that the target levies on themselves associates with WBM. In Fig 7, the standardized mean differences indicate small to medium higher ratings of anxiety, guilt, shame, depression, weight-bias internalization, and body dissatisfaction and moderately lower ratings of core self-evaluations for those focal employees who experienced WBM in the past six months as opposed to those focal employees who did not experience WBM. In Tables 14 and 15, these nine measures of self-objectification account for 6.6% of the total discrimination in experiencing WBM and 18.4% of the total variance in the composite WBM rate. The dominance analysis suggests core self-evaluations and shame account for most of the fitted discrimination in experiencing WBM (GD = 3.8%; RGD = 57.8%) whereas anxiety and guilt account for a little less than half of the fitted variance in the composite WBM rate (GD = 8.0%; RGD = 45.6%). To summarize the key findings for RQ7: the extent to which individuals felt worse about themselves increased the probability of individuals experiencing WBM and the rate of experiencing WBM to a moderate degree.

**Table 14:** Logistic regression and dominance analysis for objectification of weight by the self on binary WBM (RQ7).

	GD	RGD	B	SE	t	p
Intercept			3.313	1.063	3.117	0.002
Anxiety	0.3%	5.0%	−0.051	0.100	−0.506	0.613
Guilt	0.6%	8.3%	0.125	0.099	1.263	0.207
Shame	1.4%	21.1%	0.242	0.118	2.050	0.040
Depression	0.6%	8.5%	−0.113	0.127	−0.894	0.371
Core Self-Evaluations	2.4%	36.7%	−0.728	0.184	−3.948	< 0.001
Weight-Bias Internalization	0.5%	7.5%	−0.196	0.140	−1.405	0.160
Objectified Body Consc.	0.1%	1.5%	−0.101	0.087	−1.160	0.246
Intuitive Eating Trust	0.1%	2.0%	−0.002	0.073	−0.033	0.974
Body Dissatisfaction	0.6%	9.4%	0.139	0.057	2.456	0.014
Total R^2^	6.6%					
Sample Size	983					
Listwise Deletion	25					

Same notes as Table 2. Consc. = Consciousness.

**Table 15:** Ordinary least-squares regression and dominance analysis for objectification of weight by the self on composite WBM rate (RQ7).

	GD	RGD	B	SE	t	p
Intercept			1.424	0.152	9.362	< 0.001
Anxiety	3.6%	19.5%	0.042	0.014	3.082	0.002
Guilt	4.4%	24.1%	0.056	0.013	4.190	< 0.001
Shame	1.2%	6.3%	0.023	0.018	1.290	0.198
Depression	1.7%	9.1%	−0.010	0.018	−0.577	0.564
Core Self-Evaluations	2.0%	11.0%	−0.057	0.026	−2.182	0.029
Weight-Bias Internalization	0.8%	4.1%	−0.049	0.021	−2.286	0.023
Objectified Body Consc.	2.1%	11.6%	0.036	0.012	2.972	0.003
Intuitive Eating Trust	0.5%	2.6%	0.012	0.011	1.124	0.261
Body Dissatisfaction	2.2%	11.8%	−0.031	0.008	−3.883	< 0.001
Total R^2^	18.4%					
Sample Size	736					
Listwise Deletion	22					

Same notes as Table 14.

**Fig 8:**
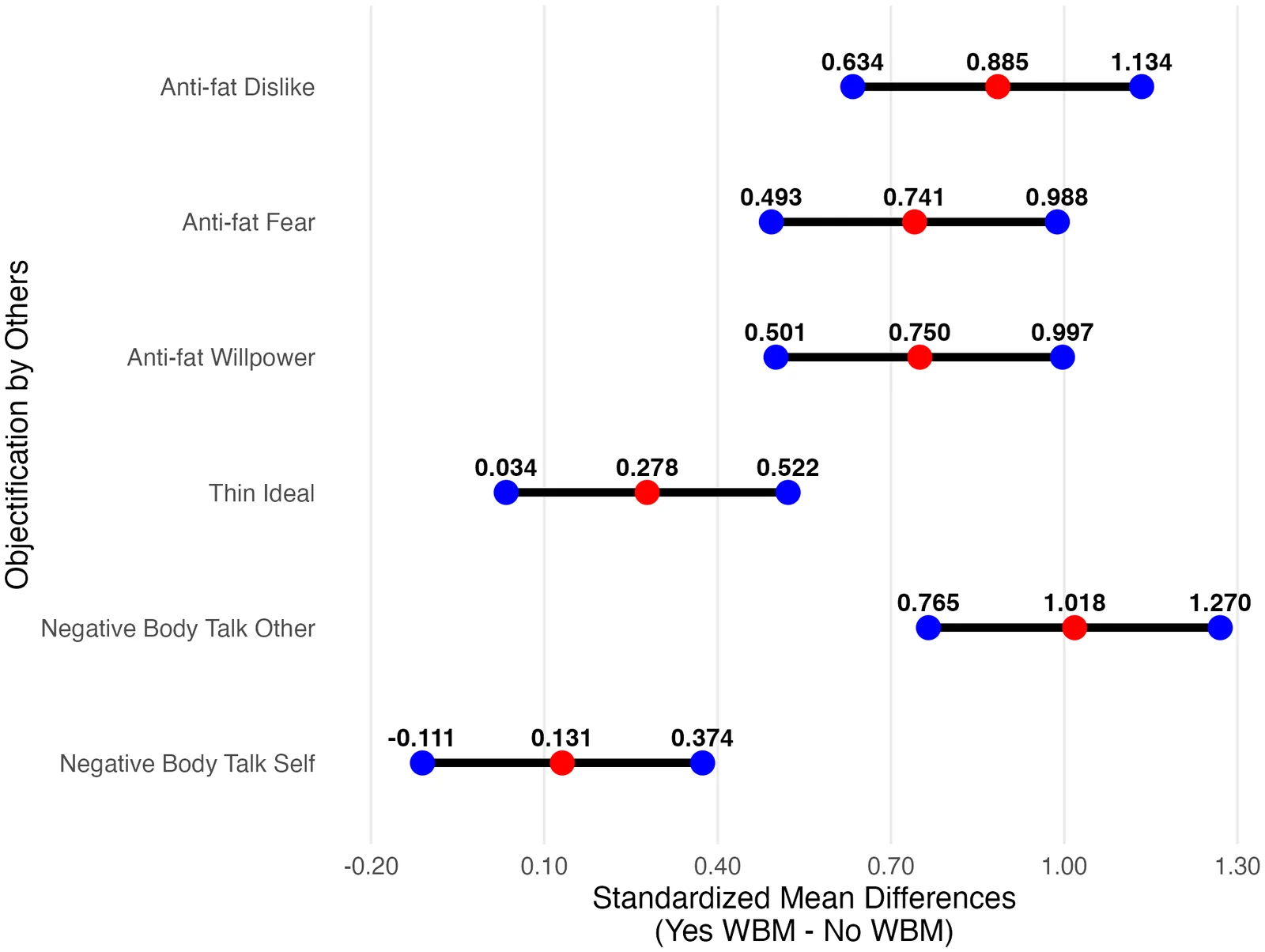
Standardized mean differences with 95% confidence intervals on objectification of weight by others for “Yes” Minus “No” WBM (RQ8).

The eighth research question asked objectification of weight by others associated with rates of WBM. In Fig 8, the standardized mean differences indicate large higher ratings of dislike of larger-bodied individuals, fear of gaining weight, larger-bodied individuals lacking willpower, and negative talk about the bodies of others for those focal employees who experienced WBM in the past six months as opposed to those focal employees who did not experience WBM. In Tables 16 and 17, these six evaluations of objectification of weight by others account for 20.1% of the total discrimination in experiencing WBM and 14.5% of the total variance in the composite WBM rate. The dominance analysis suggests negative talk about the bodies of others and anti-fat dislike account for more than two-thirds of the fitted discrimination in experiencing WBM (GD = 14.3%; RGD = 71.1%) whereas dislike of larger-bodied individuals, fear of gaining weight, and negative talk about the bodies of others account for most of the fitted variance in the composite WBM rate (GD = 11.6%; RGD = 79.7%). To summarize the key findings for RQ8: the extent to which individuals experienced anti-fat attitudes and negative body talk increased the probability of individuals experiencing WBM and the rate of experiencing WBM to a large degree.

**Table 16:** Logistic regression and dominance analysis for objectification of weight by others on binary WBM (RQ8).

	GD	RGD	B	SE	t	p
Intercept			−0.889	0.551	−1.614	0.106
Anti-fat Dislike	4.4%	22.1%	0.375	0.246	1.523	0.128
Anti-fat Fear	2.3%	11.5%	0.022	0.276	0.079	0.937
Anti-fat Willpower	2.4%	12.0%	0.205	0.187	1.095	0.274
Thin Ideal	0.4%	2.1%	−0.256	0.186	−1.377	0.169
Negative Body Talk Other	9.9%	49.0%	0.970	0.228	4.262	< 0.001
Negative Body Talk Self	0.7%	3.3%	−0.397	0.208	−1.915	0.056
Total R^2^	20.1%					
Sample Size	382					
Listwise Deletion	1					

Same notes as Table 2.

**Table 17:** Ordinary least-squares regression and dominance analysis for objectification of weight by others on composite WBM rate (RQ8).

	GD	RGD	B	SE	t	p
Intercept			1.028	0.073	14.157	< 0.001
Anti-fat Dislike	4.9%	34.0%	0.063	0.026	2.419	0.016
Anti-fat Fear	3.4%	23.2%	0.039	0.031	1.235	0.218
Anti-fat Willpower	1.2%	8.5%	−0.041	0.022	−1.824	0.069
Thin Ideal	1.2%	8.2%	0.014	0.019	0.743	0.458
Negative Body Talk Other	3.3%	22.5%	0.041	0.025	1.615	0.107
Negative Body Talk Self	0.5%	3.7%	0.013	0.022	0.574	0.567
Total R^2^	14.5%					
Sample Size	299					
Listwise Deletion	0					

Same notes as Table 2.

**Fig 9:**
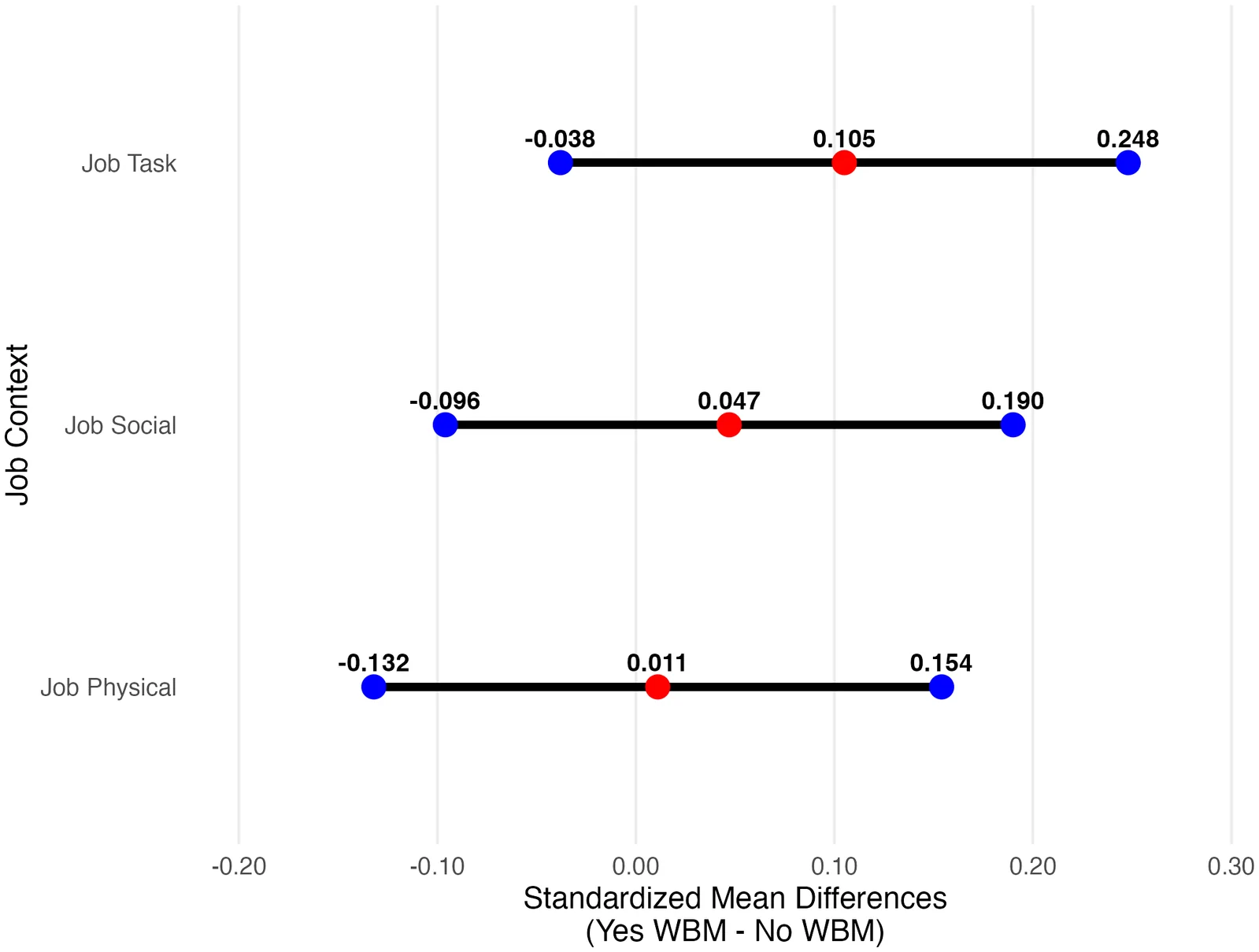
Standardized mean differences with 95% confidence intervals on job context for “Yes” Minus “No” WBM (RQ9).

The ninth research question asked how job context associates with WBM. In Fig 9, the standardized mean differences indicate negligible differences for those focal employees who experienced WBM in the past six months as opposed to those focal employees who did not experience WBM. In Tables 18 and 19, these three job context measures account for 0.2% of the total discrimination in experiencing WBM and 0.8% of the total variance in the composite WBM rate. Hence, as a summary of RQ9, these small total R^2^ values indicate the negligible association of job context on the probability of individuals experiencing WBM and the rate of experiencing WBM.

**Table 18:** Logistic Regression and dominance analysis for job context on binary WBM (RQ9).

	GD	RGD	B	SE	t	p
Intercept			−0.369	1.127	−0.327	0.743
Job Task	0.2%	88.4%	0.417	0.321	1.297	0.195
Job Social	0.0%	10.8%	0.040	0.241	0.166	0.868
Job Physical	0.0%	0.8%	−0.023	0.193	−0.121	0.904
Total R^2^	0.2%					
Sample Size	1007					
Listwise Deletion	1					

Same notes as Table 2.

**Table 19:** Ordinary least-squares regression and dominance analysis for job context on composite WBM rate (RQ9).

	GD	RGD	B	SE	t	p
Intercept			1.545	0.188	8.236	< 0.001
Job Task	0.5%	61.5%	−0.115	0.053	−2.163	0.031
Job Social	0.3%	37.6%	0.072	0.040	1.803	0.072
Job Physical	0.0%	0.9%	0.010	0.032	0.325	0.745
Total R^2^	0.8%					
Sample Size	757					
Listwise Deletion	1					

Same notes as Table 2.

### Supplemental analyses

We conducted additional *post-hoc* analyses on occupational context because the work of finds that context can both drive associations we see in the workplace, but also, interact with existing workplace features. To the latter possibility, we evaluated if occupational contexts moderated the relationship between focal correlates of WBM and the composite WBM rate. We report the results in S18 Table, but we summarily conclude that occupation minimally moderates any of the proposed relationships between focal correlates of WBM and the composite WBM rate.

## Discussion

This research’s main questions can be summarized as: *What employee and organizational features generally correlate with WBM?* To address this question, we drew on multi-disciplinary research and theory to articulate and evaluate four frames of weight stigmatization. Each suggest distinct constructs that may correlate with WBM. First, through the frame of *stereotyping,* automatic devaluation of larger bodied colleagues as it relates to core interpersonal traits (i.e., warmth; competence) may create a sense that WBM is somehow deserved and thus relate to its likelihood of co-occurrence [38]. Second, using the *justification-suppression model of prejudice* frame, we evaluated whether workplace climates or target features that affirm and/or compound stigmatization of larger-bodied people are related to the likelihood of larger-bodied workers experiencing WBM. Certain climates may tacitly signal the acceptability of mistreatment or make more salient or legitimate larger body size as a source of derision [21,63,77,112]. Furthermore, larger-bodied employees often experience *objectification* [9], which is judgment of a person based on if they measure up to a specific attribute that typically germinates from social consensus (and pressure) about ideal physical representation. We suggested stigmatizing judgments about body weight held by either the target or perpetrator makes targets particularly vulnerable to predation. Finally, occupational attributes can work as gate-keeping mechanisms when representations of what an ideal occupational occupant looks like bias people against unideal incumbents [73]. Here we investigated Johns’ [11] *task, physical,* and *social job contexts* to see if incongruities between occupational requirements and stereotypical attributes assigned to larger-bodied people co-occurs with WBM (e.g., [113]). As a broad theoretical contribution, we extend understanding of WBM by underscoring the range and diversity of variables that generally correlate with this form of mistreatment. The results of our exploratory, descriptive methods for analyzing these frames sought to transparently report the totality of our data collection effort, highlighting varied avenues for understanding WBM’s correlates. These are discussed next.

### “Othering” increases WBM

In our sample, coworker ratings of focal employees’ warmth and competence – in comparison to self-reported ratings – more strongly differentiated which larger-bodied respondents did and did not experience WBM, as well as WBM’s strength. In this way, when focal respondents were “othered” by coworkers through biased warmth and competency perceptions, they were more likely to be mistreated due to their size. This directly extends research on the “The Big Two” attributes of warmth and competence by demonstrating that among the three distinct points of view about these traits – evaluation of the self, evaluation of how you think others evaluate you, how another actually reports evaluating you – that last evaluation seemingly matters most, particularly when it relates to competency [114], when evaluating the association of these traits with WBM. Further evidence of the relationship between “othering” and WBM emerged when looking at the demographic context. These differences include possessing a visible disability, an accent, and an ethnic name. These findings extend recent work on stigma that asks researchers to evaluate compounding effects of possessing multiple stigmatized features [53]; here we offer preliminary evidence that the combinatory effect of being larger *and* possessing a noticeable indicator conveying lower social status was associated with more frequent reporting of WBM.

### Workplace climates that do and do not attenuate WBM

Formal HR programs and communications about dieting, exercise, and health did little to differentiate receipt or strength of WBM. While HR communication of health and wellness certainly needs to be decoupled [48], it does not seem to relate to WBM. More striking and practically important in our sample are the relationships between workplace climates that seemingly promote implicit and explicit weight stigmatizing messaging through daily workplace chatter, such as when negative talk about body size is common among peers, where thinness is described by coworkers as an important goal, and/or where abject pressure is placed on fellow colleagues to be slimmer. In this way, climates that promote being thinner through allowing conversation that directly pressures fellow employees to reduce their body size suggest the atmospheric impact of weight stigmatizing attitudes influence the rate of WBM. There are two theoretical implications of this. First, emergent stigmatization of body weight among peers as an attitude seemingly trickles down to impact individual perpetrator behavioral manifestations of stigmatization vis-à-vis WBM, indicating a potential cross-level relationship [53]. The second theoretical implication of this finding extends objectification theory as it pertains to body weight: results suggest that it is not only direct judgments of the target that signify objectification that potentially promotes WBM, but broader objectification through casual conversation also seem to relate to occurrence of WBM. Furthermore, myriad research studies demonstrating the association between weight and poorer economic outcomes suggest that the outcomes of objectification go beyond poorer affective states, as previously hypothesized (e.g., [115]). Finally, our results suggest that climates that generally are aggressive, uncivil, and unsupportive may create conditions ripe for WBM. As a main contribution, then, the affective tone of typical coworker interactions seems to justify stigmatizing attitudes toward fatness more readily than how HR frames health-related messaging.

Yet the news about climate was not solely bad: a culture of inclusion differentiated between the larger-bodied people who did and did not experience WBM, offering insight into a potential mechanism of weight-focused prejudice suppression. Two aspects of Nishii’s [47] climate of inclusion related negatively to WBM: equitable employment practices, which, in this context, could indicate that there is non-discrimination toward larger-bodied people in resource allocation decisions, an integration of differences, which, with respect to weight, means that those of all sizes are appreciated and valued. The general mistreatment literature documents well how organizational policies and sanctions against mistreatment prevent extensive predation [116]. And yet, the viability of this novel form of climate, which rather than being based on what should *not* be done, and instead, focuses on what should be done, points the WBM literature to examine climates synonymous with respect, dignity, and fairness for all as a means to provide some protection against WBM [117].

### Other- and self-objectification increases vulnerability to WBM

We found evidence that WBM occurred more commonly when targets seemingly objectified themselves via feeling dissatisfied with their bodies, feeling shame about themselves, and assessing their overall competency as poor. These states each suggest target vulnerability to predation [23,118]. This highlights the role that objectification theory plays in WBM in several ways. First, as previously discussed, workplace climates with stronger weight stigmatizing sentiment also tended to include targets who reported more WBM. Through the lens of the objectification process, this means the stronger the general discourse about judging body size, the more likely that judgment may be to manifest vis-à-vis WBM. Here it may be that both the strength of “ideal body size” attitude and comfort of judging someone who does not meet that ideal is more normalized. Second, objectification theory suggests that self-objectification, in which a person deems themself unacceptable because of their size and suffers confidence depletion as a result [10,119], creates a negative self-conception that – in the case of our sample – could leave larger-bodied workers susceptible to mistreatment. This suggests a direct theoretical implication: that variance of objectification of others may be compounded by a target’s self-objectification, meaning the two processes could amplify each other; future research is needed to further untangle this effect. Finally, self-objectification is a particularly interesting lens through which to view mistreatment because it leads us to consider attributions the target may make for the mistreatment. For example, we found that targets of WBM who internalized messages around fat being inappropriate, bad, or unacceptable reported more WBM. Here, perhaps attitudes of deservedness of WBM make the target vulnerable to attack [120]. Unfortunately, identity management strategies, such as re-evaluating what it means to be larger, while associated with less likelihood of experiencing WBM as a larger-bodied worker, provided little, if any, suppressive impact on the rate of mistreatment.

### Occupational associations with WBM

Put succinctly, whether rooted in social aspects of the job, physical requirements of the job, or features of an occupational task, occupational attributes had little association with degree of WBM. On one hand, this suggests that the penalty that could be levied on larger-bodied workers when they work in counter-type jobs (e.g., a larger person working a physically demanding job) does not exist. On the other hand, this suggests that occupational context provides no protection from WBM: irrespective of associations between job attributes and body size, WBM occurs. Effectively researching context means articulating what is rare, interesting, or special about an environment and how that unique context tenders an effect [121]. We expand this perspective to specify that the opposite is also true: when context fails to explain a phenomenon, that, too, is distinctive because it could mean that an even larger context is muting relations. Given the collection of other findings in this study, we suggest that the larger cultural context of stigmatization of body weight is perhaps so strong that occupational context does little to conceal its impact.

### Practical implications

Several practical implications emerge from this study’s findings. First, mistreated workers in our sample were marginally reported to be perceived as less warm and competent than peers by their coworkers, affirming core stereotypes often applied to those in larger bodies [114]. New research on combating weight stigma highlights the importance of communicating a larger body size’s complex etiology [122] – and our finding suggests that such information be supplemented by explaining not just that a person’s weight derives from multi-factorial roots, but also that it does not indicate these core attributes about a person. There are two distinct implications from this: first, that HR training on addressing weight stigma specifically include counternarratives about stereotypes we levy on larger-bodied colleagues. Also, researchers could invest time in developing and evaluating evidence-based content of such training, for example experimenting with different entry points for such conversation (e.g., within other diversity, equity, and inclusion training *or* separate; part of a broader discussion about professional appearance and demeanor at work *or* part of HR materials related to health and well-being that explicitly tackle myths around weight and wellness, an effective tactic [123,124]) or appraising various means of presenting stereotypes (e.g., just focusing on one stereotyped group at a time *or* including weight stereotypes as part of larger anti-stigma training efforts).

Another practical implication stems from a pattern of findings across two of the frames described in this study: when workplace conversation centers body weight, particularly in a negative light, we found that WBM occurs more often. For example, the more employees feel their coworkers believe that ideal bodies are smaller, that denigrating one’s own and other’s weights is normal, and that attitudes toward fatness (that is, that fatness is wrong and that larger people should be blamed for their size), the more likely WBM is to occur. This provides a clear indication that formal training, rules, or other consequential forms of employee management around how weight is talked about at work could be fruitful. For example, at work HR training talks about impermissible topics of conversation that would otherwise make a workplace feel unsafe and abusive, such as not making racist, sexist, homophobic, or related remarks, and often outline direct consequences for violation (e.g., termination). Organizations who frame talk about weight not just as taboo, but as a fundamental part of making the workplace a respectful *and* that attach consequences for violation could also experience a reduction in WBM.

A final implication of our work relates to legal concerns when WBM occurs.

Certain jurisdictions outlaw weight-based discrimination, and consequently, make a hostile work environment related to weight an occurrence serious not just for its psychological outcomes, but also its fiscal and reputational outcomes. Size diversity advocates have pressured lawmakers to expand such protections in recent years [125,126], particularly since this group (larger workers) historically possess little legal protection [127]. The present research presents clear evidence that climate preoccupations with idealized shape, size, and weight relate strongly to rates of WBM, and, as such, indicate or even contribute to a hostile work environment. A path for supporting protections for larger workers relates to conflict management, equity, and inclusion: larger-bodied people in work environments rife with unfairness and interpersonal ill-will report significantly more WBM. Furthermore, environments where anti-fat attitudes are stronger tend to also have larger-bodied workers who report WBM, indicating that monitoring and addressing (as described in the previous implication) how workers discuss body weight may prove crucial to creating environments that lack hostility toward larger people. We do note that shifting attention toward equity and inclusion will not be easy given strong cultural attitudes of fear and shame around fatness [32], but research suggests some degree of changeability of reactions to stigmatized characteristics [128].

### Strengths and limitations

We collected data from three different sources to capture multiple perspectives on the focal respondent’s current work context. Coworker data provided a peripheral view of organizational climate impacting the focal respondent, and O*NET data provided objective occupational information to better understand the focal respondent’s work context. We also transparently provided results to all correlation-related variables collected, rather than simply modeling only those that we discovered, post-hoc, differentiated mistreated versus non-mistreated larger-bodied workers. Finally, answering calls for a more multi-disciplinary approach to understanding WBM [9], we drew from multiple literatures that suggest distinctive weight-stigmatizing processes in order to best represent why WBM may occur.

In terms of weaknesses, we sought breadth over depth, meaning that while we now know that, for example, organizational climates that contain a lot of body-focused talk create a situation where WBM is more likely, we do not know which aspects of that talk are influential. Indeed, entire studies have been devoted to parsing fat talk [129], yet we focused here on basic evaluation of frequency or prevalence of our correlations. Relatedly, our manuscript does not explicate nor measure the process of WBM. While we outlined and measured constructs that predate WBM, we do not yet know the finer details of how, for instance, self-directed objectification creates weakness, or how a predator perceives and acts on that weakness.

Our research design contained several limitations starting with, first, a meta-limitation: we did not pre-register our study. Plainly stated, this omission was born of unfamiliarity with the importance of such a step at the time of the study’s inception. To course correct, we took an exploratory, descriptive approach to analyzing our data such that we present *all* correlates of WBM that we conceptualized at the time of the study’s design rather than a subset that tells a more focused yet less realistic story (i.e., we did not engage in HARKing, or hypothesizing after results were known). Furthermore, we included in this manuscript variables we initially conceived of and measured that were not pre-established measures (e.g., those on HR messaging). We hope that our exploratory, descriptive approach partially allays concern about transparency.

That said, more specific limitations also bear mentioning. First, there is potential for a homophily bias when the focal respondent sent their survey to a coworker, such as the coworker having the same body size and this impacting their perspective on, for example, aspects of organizational culture related to weight (i.e., antifat attitudes) because of their particular attunement to threats in the environment given their own body size. Research where random selection of a coworker is deployed when inquiring about aspects of workplace culture and the focal employee would lessen the potential amount of homophily bias.

Second, critically, our design precludes claims of causality and generalizability. We leveraged various literatures that intended to answer, through different lenses, why WBM occurs, which implies that the constructs evaluated herein could be conceived of as antecedents. Yet we cannot make such direct claims because our study’s design does not allow for causality testing. Because of its importance to advancing the conversation of WBM at work, we first suggest that researchers design studies that establish more causal relationships between correlates of WBM and its emergence. We generated research questions here to guide initial areas of inquiry, but studies that focus on hypothesis confirmation of causal relations are needed to untangle the potentially recursive or inverse relations proposed in the present study. Similarly, we cannot generalize results from our convenience sample to a population; evaluating generalizability would require a very planned data collection procedure. Finally, we excluded from analysis those who experienced WBM prior to the past six months. Recent research affirms that experiences with weight-based cruelty can linger long after the incident, and as such the strength of our effects may, in fact, be greater when considering all those who recalled such an incident [130].

### Future research

Researchers should explore weight-related workplace messaging. The present study suggests we ask: are there ways to frame health behaviors at work without potentially triggering WBM? Should discussions of weight at work be forbidden or regulated, and how does this create friction with free speech rights? Complicating these questions is research suggesting that cultures which promote a positive atmosphere can create the most deleterious effects when mistreatment occurs because the mistreatment is so jarring – it is not supposed to happen, so victims believe they *really* deserve it [131].

Another area for future research is intersectionality. A key question to explore is whether the conditions that fuel WBM are detrimental to groups managing multiple identities that historically receive prejudicial treatment (e.g., to be larger-bodied *and* black or to be old *and* larger-bodied). Unfortunately, we know that intersectional identities do more than layer disfavor, but instead, holders of multiple maligned identities report compounding effects [132]. Research that disentangles how stigmatization of various groups intertwine would be fruitful because any resulting training would be more likely to address the entire expanse of stigma. For example, the stereotype of incompetence affects both larger-bodied people and older people. Training to address the former and not the latter could leave employees with residual stereotypes that undo progress toward combating weight stigma.

## Conclusions

WBM occurs at an alarming rate. To understand and address the problem, our research aimed to document correlations of WBM by investigating stigmatization of a larger person’s body size through the lens of four frames: stereotyping, justification-suppression model of prejudice, objectification theory, and occupational contexts. Results suggest that larger-bodied employees report more WBM when their workplace climates promote thinness, such as normalizing the encouragement of “weight loss talk” or by failing to regulate or condemn negative body talk. Larger-bodied employees also report more WBM when they work in more plainly unsupportive or aggressive interpersonal climates. Furthermore, employees’ self-views contribute to differences between rates of WBM: larger-bodied employees who feel guilt, shame, and related aspects of self-consciousness report more mistreatment. Taken together, the origins of WBM are multi-factorial, germinating from the self, from peers, and from culture at large.

## Supporting information

S1 Table Forms of weight-based mistreatment. The question stem for each item read: “In the prior six months, to what extent did the following individuals engage in these behaviors towards you specifically because of your weight?” Participants provided responses to each item using a 3-point Likert response scale with 1 = never, 2 = sometimes, and 3 = often. Furthermore, participants responded to each item with respect to the following perpetrators: boss, coworker, subordinate, client/customer, and other. We used the top three loading items from each construct to lessen respondent fatigue.
(DOCX)

S2 Table Reliability calculations for the collapsed WBM measures. ω_h_ = omega-hierarchical; ω_t_ = omega-total; α = Cronbach’s alpha. We collapsed WBM by type (aggregated across sources) and source (aggregated across types) for ease of analysis and interpretation. See Revelle and Zinbarg (1) and Revelle (2) for more information about the omega coefficients and their advantages over Cronbach’s alpha.
(DOCX)

S3 Table Ordinary least-squares regression and dominance analysis for benevolent WBM. Incl. = Inclusion; Org. = Organizational; Neg. = Negative; Wgt. = Weight; Comm. = Communications; Imp. = Importance; Phys. = Physical; Consc. = Consciousness; GD = General Dominance; RGD = Relative General Dominance; B = Unstandardized Regression Coefficient; SE = Standard Error; t = t-test value; p = frequentist probability value. General dominance percentages sum to the total R² percentage; relative general dominance percentages sum to 100%. See Azen and Budescu (1) for more information on dominance analysis.
(DOCX)

S4 Table Ordinary least-squares regression and dominance analysis for covert WBM. Same notes as S3 Table.
(DOCX)

S5 Table Ordinary least-squares regression and dominance analysis for personal incivility WBM. Same notes as S3 Table.
(DOCX)

S6 Table Ordinary least-squares regression and dominance analysis for physical incivility WBM. Same notes as S3 Table.
(DOCX)

S7 Table Ordinary least-squares regression and dominance analysis for work incivility WBM. Same notes as S3 Table.
(DOCX)

S8 Table Ordinary least-squares regression and dominance analysis for micro-assault WBM. Same notes as S3 Table.
(DOCX)

S9 Table Ordinary least-squares regression and dominance analysis for micro-insult WBM. Same notes as S3 Table.
(DOCX)

S10 Table Ordinary least-squares regression and dominance analysis for micro-invalidation WBM. Same notes as S3 Table.
(DOCX)

S11 Table Ordinary least-squares regression and dominance analysis for overt WBM. Same notes as S3 Table.
(DOCX)

S12 Table Ordinary least-squares regression and dominance analysis for WBM by boss. Same notes as S3 Table.
(DOCX)

S13 Table Ordinary Least-Squares Regression and Dominance Analysis for WBM by Coworker. Same notes as S3 Table.
(DOCX)

S14 Table Ordinary least-squares regression and dominance analysis for wbm by customer. Same notes as S3 Table.
(DOCX)

S15 Table Ordinary least-squares regression and dominance analysis for WBM by subordinate. Same notes as S3 Table.
(DOCX)

S16 Table Ordinary least-squares regression and dominance analysis for WBM by other. Same notes as S3 Table.
(DOCX)

S17 Table Correlations between all antecedent correlates. Incl. = Inclusion; Org. = Organizational; Neg. = Negative; Wgt. = Weight; Comm. = Communications; Imp. = Importance; Phys. = Physical; Consc. = Consciousness; Gender: Male = 0 and Female = 1; Race: Non-White = 0 and White = 1; r = Pearson product-moment correlation; t = t-test value; df = degrees of freedom; p = frequentist probability value.
(DOCX)

S18 Table Occupational context correlates as moderators between focal correlates of WBM and composite WBM rate. ΔR^2^ = change in R^2^ between the model with just the main effects of the focal correlate of WBM and the occupational job context moderator and the model with the main effects plus the interaction effect between two predictor variables. F = the F ratio for the change in R^2^; p = frequentist probability value of the F ratio for the change in R^2^.
(DOCX)
