# Supplementary material for: An exploratory study of the correlates of weight-based mistreatment at work
Source: PLoS One. 2026 Aug 26;21(8):e0342144. doi: 10.1371/journal.pone.0342144 (PMC13514019; doi:10.1371/journal.pone.0342144)
Supplement: Supplementary file 1 [file pone.0342144.s001.docx]

**S1 Table*.* Forms of Weight-Based Mistreatment.**

| **Variable** | **Source** | **Example Item** |
| --- | --- | --- |
| Overt Aggression | Glomb (1) | Because of my weight, this person yelled or raised their voice. |
| Covert Aggression | Glomb (1) | Because of my weight, people at work have made me look bad. |
| Incivility | Einarsen, Hoel, and Notelaers (2) | *Work-related incivility*: Because of my weight, my opinions have been ignored.  *Person-related incivility:* Because of my weight, I have received persistent criticism of my errors and mistakes.  *Physically-intimidating incivility*: Because of my weight, I have been shouted at or was the target of spontaneous anger. |
| Microaggressions | Derivation of Sue and colleagues (3) | *Microassault:* To what extent have you heard people at work use crass or inappropriate words to describe your size?  *Microinvalidation:* To what extent are you made to feel your feelings and emotions as a fat person are unimportant?  *Microinsult:* To what extent have you experienced rudeness about your size in the workplace that does not include calling you mean-spirited names? |
| Benevolent | Herbo and Thompson (4) | Because of my weight, this person told me to go on a diet. |

The question stem for each item read: “In the prior six months, to what extent did the following individuals engage in these behaviors towards you specifically because of your weight?” Participants provided responses to each item using a 3-point Likert response scale with 1 = never, 2 = sometimes, and 3 = often. Furthermore, participants responded to each item with respect to the following perpetrators: boss, coworker, subordinate, client/customer, and other. We used the top three loading items from each construct to lessen respondent fatigue.
