# Supplementary material for: An exploratory study of the correlates of weight-based mistreatment at work
Source: PLoS One. 2026 Aug 26;21(8):e0342144. doi: 10.1371/journal.pone.0342144 (PMC13514019; doi:10.1371/journal.pone.0342144)
Supplement: Supplementary file 2 [file pone.0342144.s002.docx]

**S2 Table. Reliability Calculations for the Collapsed WBM Measures.**

| **WBM** | **ω_h_** | **ω_t_** | **α** |
| --- | --- | --- | --- |
| *Type of WBM* |  |  |  |
| Benevolent | 0.67 | 0.93 | 0.90 |
| Covert | 0.67 | 0.94 | 0.93 |
| Incivility-Personal | 0.74 | 0.92 | 0.90 |
| Incivility-Physical | 0.73 | 0.93 | 0.92 |
| Incivility-Work Related | 0.71 | 0.92 | 0.90 |
| Micro-assault | 0.57 | 0.88 | 0.83 |
| Micro-insult | 0.69 | 0.90 | 0.86 |
| Micro-invalidation | 0.68 | 0.90 | 0.87 |
| Overt | 0.72 | 0.96 | 0.96 |
| *Source of WBM* |  |  |  |
| Boss | 0.83 | 0.97 | 0.96 |
| Coworker | 0.78 | 0.94 | 0.93 |
| Customer | 0.84 | 0.96 | 0.96 |
| Subordinate | 0.81 | 0.97 | 0.96 |
| Other | 0.81 | 0.96 | 0.96 |

ω_h_ = omega-hierarchical; ω_t_ = omega-total; α = Cronbach’s alpha. We collapsed WBM by type (aggregated across sources) and source (aggregated across types) for ease of analysis and interpretation. See Revelle and Zinbarg (1) and Revelle (2) for more information about the omega coefficients and their advantages over Cronbach’s alpha.

References:

1. Revelle W, Zinbarg RW. Coefficients alpha, beta, omega, and the GLB: Comments on Sijtsma. Psychometrika [Internet]. 2009;74(145–154). Available from: https://www.personality-project.org/revelle/publications/rz09.pdf

2.	Revelle W. Classical test theory and the measurement of reliability [Internet]. 2020 Accessed. Available from: https://www.personality-project.org/r/book/#chapter7
