# Supplementary material for: An exploratory study of the correlates of weight-based mistreatment at work
Source: PLoS One. 2026 Aug 26;21(8):e0342144. doi: 10.1371/journal.pone.0342144 (PMC13514019; doi:10.1371/journal.pone.0342144)
Supplement: Supplementary file 3 [file pone.0342144.s003.docx]

**S3 Table. Ordinary Least-Squares Regression and Dominance Analysis for Benevolent WBM.**

| **Correlates** | **GD** | **RGD** | **B** | **SE** | **t** | **p** |
| --- | --- | --- | --- | --- | --- | --- |
| *Stereotypes* |  |  |  |  |  |  |
| Intercept |  |  | 1.895 | 0.140 | 13.517 | < 0.001 |
| Yourself Competent | 0.1% | 3.4% | -0.008 | 0.024 | -0.329 | 0.742 |
| Others Competent | 0.4% | 8.5% | -0.002 | 0.029 | -0.077 | 0.939 |
| Coworker Competent | 2.6% | 59.6% | -0.065 | 0.030 | -2.168 | 0.031 |
| Yourself Warm | 0.1% | 1.8% | 0.017 | 0.030 | 0.576 | 0.565 |
| Others Warm | 0.2% | 4.5% | -0.021 | 0.033 | -0.632 | 0.528 |
| Coworker Warm | 1.0% | 22.2% | -0.019 | 0.033 | -0.592 | 0.554 |
| Total R^2^ | 4.3% |  |  |  |  |  |
| Sample Size | 293 |  |  |  |  |  |
| Listwise Deletion | 6 |  |  |  |  |  |
| *Interpersonal Climate* |  |  |  |  |  |  |
| Intercept |  |  | 1.405 | 0.137 | 10.274 | < 0.001 |
| Climate Incl. Equitable | 0.2% | 2.0% | 0.013 | 0.031 | 0.436 | 0.663 |
| Climate Incl. Integration | 1.4% | 11.4% | -0.073 | 0.038 | -1.935 | 0.054 |
| Climate Incl. DM | 0.7% | 6.3% | 0.062 | 0.032 | 1.926 | 0.055 |
| Civility Climate | 2.3% | 19.5% | -0.047 | 0.030 | -1.560 | 0.120 |
| Leader-Member Exchange | 0.5% | 4.5% | -0.004 | 0.028 | -0.128 | 0.898 |
| Perceived Org. Support | 0.7% | 5.9% | 0.003 | 0.030 | 0.115 | 0.908 |
| Interpersonal Conflict | 6.0% | 50.5% | 0.115 | 0.029 | 3.921 | < 0.001 |
| Total R² | 12.0% |  |  |  |  |  |
| Sample Size | 299 |  |  |  |  |  |
| Listwise Deletion | 0 |  |  |  |  |  |
| *Weight Control Climate* |  |  |  |  |  |  |
| Intercept |  |  | 0.983 | 0.096 | 10.218 | < 0.001 |
| Tolerate Neg. Wgt. Talk | 6.8% | 47.5% | 0.080 | 0.021 | 3.832 | < 0.001 |
| Pressure for Thinness | 3.9% | 26.9% | 0.054 | 0.025 | 2.135 | 0.034 |
| Climate for Exercise | 0.8% | 5.5% | 0.042 | 0.038 | 1.093 | 0.275 |
| Wgt. Org. Support | 0.2% | 1.6% | -0.002 | 0.031 | -0.058 | 0.954 |
| Wgt. Social Support | 0.5% | 3.7% | -0.057 | 0.031 | -1.811 | 0.071 |
| Wgt. Diet Norms | 0.9% | 6.3% | 0.052 | 0.032 | 1.641 | 0.102 |
| Wgt. Belittle | 1.2% | 8.5% | 0.024 | 0.022 | 1.069 | 0.286 |
| Total R² | 14.4% |  |  |  |  |  |
| Sample Size | 299 |  |  |  |  |  |
| Listwise Deletion | 0 |  |  |  |  |  |
| *Human Resources Policies* |  |  |  |  |  |  |
| Intercept |  |  | 1.512 | 0.058 | 26.163 | < 0.001 |
| Offer Exercise Program | 0.2% | 9.4% | 0.037 | 0.073 | 0.505 | 0.614 |
| Offer Weight Loss | 0.1% | 3.8% | 0.017 | 0.072 | 0.232 | 0.817 |
| Offer Maintain Health | 0.3% | 16.2% | -0.047 | 0.064 | -0.733 | 0.464 |
| Comm. Exercise Program | 0.2% | 9.0% | 0.022 | 0.049 | 0.450 | 0.653 |
| Comm. Weight Loss | 0.2% | 12.6% | 0.035 | 0.048 | 0.731 | 0.466 |
| Comm. Maintain Health | 0.9% | 49.0% | -0.062 | 0.040 | -1.551 | 0.122 |
| Total R² | 1.8% |  |  |  |  |  |
| Sample Size | 249 |  |  |  |  |  |
| Listwise Deletion | 50 |  |  |  |  |  |
| *Social Status* |  |  |  |  |  |  |
| Intercept |  |  | 1.585 | 0.131 | 12.107 | < 0.001 |
| Education Breakdown | 0.9% | 9.9% | -0.035 | 0.014 | -2.468 | 0.014 |
| Weight Comparison | 1.4% | 14.8% | -0.047 | 0.014 | -3.241 | 0.001 |
| Gender Breakdown | 0.0% | 0.2% | 0.003 | 0.007 | 0.489 | 0.625 |
| Manager | 1.3% | 13.1% | 0.098 | 0.036 | 2.731 | 0.006 |
| Visible Disability | 1.4% | 14.8% | 0.038 | 0.020 | 1.909 | 0.057 |
| Ethnic Name | 2.3% | 24.3% | 0.045 | 0.018 | 2.478 | 0.013 |
| Body-Mass Index | 0.1% | 0.6% | 0.001 | 0.002 | 0.456 | 0.648 |
| Accent | 1.8% | 18.7% | 0.037 | 0.018 | 2.029 | 0.043 |
| Gender | 0.1% | 1.2% | -0.029 | 0.035 | -0.817 | 0.415 |
| Race | 0.2% | 2.5% | -0.037 | 0.041 | -0.907 | 0.365 |
| Total R² | 9.6% |  |  |  |  |  |
| Sample Size | 592 |  |  |  |  |  |
| Listwise Deletion | 166 |  |  |  |  |  |
| *Identity Management Strategies* |  |  |  |  |  |  |
| Intercept |  |  | 1.539 | 0.062 | 24.959 | < 0.001 |
| Redefinition Reassociate | 0.4% | 23.5% | 0.028 | 0.017 | 1.630 | 0.104 |
| Redefinition Regenerate | 0.1% | 7.0% | 0.011 | 0.022 | 0.512 | 0.609 |
| Switch Deemphasize | 0.2% | 15.1% | 0.044 | 0.025 | 1.797 | 0.073 |
| Switch Emphasize | 0.9% | 54.4% | -0.066 | 0.021 | -3.122 | 0.002 |
| Total R² | 1.6% |  |  |  |  |  |
| Sample Size | 757 |  |  |  |  |  |
| Listwise Deletion | 1 |  |  |  |  |  |
| *Objectification by the Self* |  |  |  |  |  |  |
| Intercept |  |  | 1.323 | 0.202 | 6.557 | < 0.001 |
| Anxiety | 2.3% | 18.2% | 0.041 | 0.018 | 2.245 | 0.025 |
| Guilt | 3.4% | 27.0% | 0.067 | 0.018 | 3.738 | < 0.001 |
| Shame | 1.9% | 14.6% | 0.063 | 0.024 | 2.681 | 0.008 |
| Depression | 1.2% | 9.4% | -0.019 | 0.024 | -0.778 | 0.437 |
| Core Self-Evaluations | 1.1% | 9.0% | -0.025 | 0.035 | -0.728 | 0.467 |
| Weight-Bias Internalization | 0.6% | 4.5% | -0.053 | 0.028 | -1.873 | 0.061 |
| Objectified Body Consc. | 1.4% | 11.2% | 0.039 | 0.016 | 2.399 | 0.017 |
| Intuitive Eating Trust | 0.1% | 0.9% | 0.001 | 0.014 | 0.087 | 0.931 |
| Body Dissatisfaction | 0.7% | 5.2% | -0.022 | 0.011 | -2.075 | 0.038 |
| Total R² | 12.7% |  |  |  |  |  |
| Sample Size | 735 |  |  |  |  |  |
| Listwise Deletion | 23 |  |  |  |  |  |
| *Objectification by Others* |  |  |  |  |  |  |
| Intercept |  |  | 1.035 | 0.094 | 11.059 | < 0.001 |
| Anti-fat Dislike | 3.7% | 27.2% | 0.058 | 0.033 | 1.747 | 0.082 |
| Anti-fat Fear | 2.8% | 20.4% | 0.025 | 0.040 | 0.632 | 0.528 |
| Anti-fat Willpower | 1.2% | 9.0% | -0.015 | 0.029 | -0.537 | 0.592 |
| Thin Ideal | 2.1% | 15.5% | 0.041 | 0.024 | 1.714 | 0.088 |
| Negative Body Talk Other | 3.5% | 25.4% | 0.060 | 0.033 | 1.857 | 0.064 |
| Negative Body Talk Self | 0.3% | 2.4% | -0.006 | 0.029 | -0.200 | 0.842 |
| Total R² | 13.8% |  |  |  |  |  |
| Sample Size | 299 |  |  |  |  |  |
| Listwise Deletion | 0 |  |  |  |  |  |
| *Job Context* |  |  |  |  |  |  |
| Intercept |  |  | 1.436 | 0.246 | 5.833 | < 0.001 |
| Job Task | 0.2% | 21.9% | -0.106 | 0.070 | -1.524 | 0.128 |
| Job Social | 0.6% | 75.4% | 0.124 | 0.052 | 2.376 | 0.018 |
| Job Physical | 0.0% | 2.8% | 0.025 | 0.042 | 0.604 | 0.546 |
| Total R² | 0.8% |  |  |  |  |  |
| Sample Size | 756 |  |  |  |  |  |
| Listwise Deletion | 2 |  |  |  |  |  |

Incl. = Inclusion; Org. = Organizational; Neg. = Negative; Wgt. = Weight; Comm. = Communications; Imp. = Importance; Phys. = Physical; Consc. = Consciousness; Gender: Male = 0 and Female = 1; Race: Non-White = 0 and White = 1; GD = General Dominance; RGD = Relative General Dominance; B = Unstandardized Regression Coefficient; SE = Standard Error; t = t-test value; p = frequentist probability value; p_bh_ = Benjamini-Hochberg correction to frequentist probability value. General dominance percentages sum to the total R² percentage; relative general dominance percentages sum to 100%. See Azen and Budescu (1) for more information on dominance analysis.

References:

1. Azen R, Budescu DV. The dominance analysis approach for comparing predictors in multiple regression. Psychol Methods. 2003;8(2):129–48.
