# Supplementary material for: An exploratory study of the correlates of weight-based mistreatment at work
Source: PLoS One. 2026 Aug 26;21(8):e0342144. doi: 10.1371/journal.pone.0342144 (PMC13514019; doi:10.1371/journal.pone.0342144)
Supplement: Supplementary file 4 [file pone.0342144.s004.docx]

**S4 Table. Ordinary Least-Squares Regression and Dominance Analysis for Covert WBM.**

| **Correlates** | **GD** | **RGD** | **B** | **SE** | **t** | **p** |
| --- | --- | --- | --- | --- | --- | --- |
| *Stereotypes* |  |  |  |  |  |  |
| Intercept |  |  | 1.897 | 0.124 | 15.308 | < 0.001 |
| Yourself Competent | 0.8% | 8.9% | -0.021 | 0.021 | -0.980 | 0.328 |
| Others Competent | 1.2% | 13.1% | -0.021 | 0.025 | -0.812 | 0.417 |
| Coworker Competent | 4.1% | 44.9% | -0.059 | 0.026 | -2.242 | 0.026 |
| Yourself Warm | 0.1% | 1.2% | 0.012 | 0.026 | 0.465 | 0.642 |
| Others Warm | 0.1% | 1.5% | 0.013 | 0.029 | 0.441 | 0.660 |
| Coworker Warm | 2.8% | 30.4% | -0.051 | 0.029 | -1.754 | 0.081 |
| Total R^2^ | 9.1% |  |  |  |  |  |
| Sample Size | 292 |  |  |  |  |  |
| Listwise Deletion | 7 |  |  |  |  |  |
| *Interpersonal Climate* |  |  |  |  |  |  |
| Intercept |  |  | 1.357 | 0.120 | 11.268 | < 0.001 |
| Climate Incl. Equitable | 1.1% | 6.9% | -0.019 | 0.027 | -0.711 | 0.477 |
| Climate Incl. Integration | 1.8% | 11.0% | -0.049 | 0.033 | -1.477 | 0.141 |
| Climate Incl. DM | 0.8% | 5.0% | 0.033 | 0.028 | 1.164 | 0.245 |
| Civility Climate | 1.3% | 8.3% | 0.003 | 0.026 | 0.104 | 0.918 |
| Leader-Member Exchange | 1.9% | 11.7% | -0.031 | 0.025 | -1.254 | 0.211 |
| Perceived Org. Support | 1.4% | 8.6% | -0.004 | 0.027 | -0.154 | 0.878 |
| Interpersonal Conflict | 7.8% | 48.5% | 0.119 | 0.026 | 4.599 | < 0.001 |
| Total R² | 16.0% |  |  |  |  |  |
| Sample Size | 298 |  |  |  |  |  |
| Listwise Deletion | 1 |  |  |  |  |  |
| *Weight Control Climate* |  |  |  |  |  |  |
| Intercept |  |  | 0.930 | 0.087 | 10.629 | < 0.001 |
| Tolerate Neg. Wgt. Talk | 6.6% | 50.2% | 0.069 | 0.019 | 3.635 | < 0.001 |
| Pressure for Thinness | 2.7% | 20.4% | 0.037 | 0.023 | 1.617 | 0.107 |
| Climate for Exercise | 0.2% | 1.3% | 0.006 | 0.035 | 0.173 | 0.863 |
| Wgt. Org. Support | 0.2% | 1.3% | 0.018 | 0.028 | 0.632 | 0.528 |
| Wgt. Social Support | 0.3% | 2.5% | -0.041 | 0.028 | -1.462 | 0.145 |
| Wgt. Diet Norms | 0.4% | 3.2% | 0.036 | 0.029 | 1.236 | 0.217 |
| Wgt. Belittle | 2.8% | 21.1% | 0.043 | 0.020 | 2.124 | 0.035 |
| Total R² | 13.1% |  |  |  |  |  |
| Sample Size | 298 |  |  |  |  |  |
| Listwise Deletion | 1 |  |  |  |  |  |
| *Human Resources Policies* |  |  |  |  |  |  |
| Intercept |  |  | 1.421 | 0.053 | 26.994 | < 0.001 |
| Offer Exercise Program | 0.1% | 3.9% | -0.019 | 0.066 | -0.290 | 0.772 |
| Offer Weight Loss | 0.2% | 6.7% | 0.044 | 0.066 | 0.660 | 0.510 |
| Offer Maintain Health | 0.6% | 23.8% | -0.062 | 0.058 | -1.065 | 0.288 |
| Comm. Exercise Program | 0.2% | 6.1% | 0.001 | 0.044 | 0.028 | 0.978 |
| Comm. Weight Loss | 0.8% | 33.1% | 0.071 | 0.044 | 1.608 | 0.109 |
| Comm. Maintain Health | 0.7% | 26.5% | -0.051 | 0.036 | -1.413 | 0.159 |
| Total R² | 2.5% |  |  |  |  |  |
| Sample Size | 249 |  |  |  |  |  |
| Listwise Deletion | 50 |  |  |  |  |  |
| *Social Status* |  |  |  |  |  |  |
| Intercept |  |  | 1.165 | 0.105 | 11.084 | < 0.001 |
| Education Breakdown | 0.7% | 5.3% | -0.022 | 0.011 | -1.974 | 0.049 |
| Weight Comparison | 0.8% | 6.2% | -0.028 | 0.012 | -2.458 | 0.014 |
| Gender Breakdown | 0.8% | 5.6% | 0.013 | 0.006 | 2.279 | 0.023 |
| Manager | 1.4% | 10.2% | 0.083 | 0.029 | 2.902 | 0.004 |
| Visible Disability | 3.4% | 24.9% | 0.057 | 0.016 | 3.540 | < 0.001 |
| Ethnic Name | 2.1% | 15.7% | 0.030 | 0.015 | 2.018 | 0.044 |
| Body-Mass Index | 0.3% | 2.0% | 0.002 | 0.002 | 1.241 | 0.215 |
| Accent | 3.2% | 23.6% | 0.046 | 0.015 | 3.172 | 0.002 |
| Gender | 0.8% | 6.1% | 0.061 | 0.028 | 2.129 | 0.034 |
| Race | 0.0% | 0.3% | < 0.001 | 0.033 | -0.014 | 0.989 |
| Total R² | 13.5% |  |  |  |  |  |
| Sample Size | 592 |  |  |  |  |  |
| Listwise Deletion | 166 |  |  |  |  |  |
| *Identity Management Strategies* |  |  |  |  |  |  |
| Intercept |  |  | 1.403 | 0.051 | 27.332 | < 0.001 |
| Redefinition Reassociate | 0.4% | 23.0% | 0.018 | 0.015 | 1.243 | 0.214 |
| Redefinition Regenerate | 0.5% | 32.0% | 0.033 | 0.018 | 1.828 | 0.068 |
| Switch Deemphasize | 0.1% | 5.4% | 0.015 | 0.021 | 0.729 | 0.466 |
| Switch Emphasize | 0.7% | 39.7% | -0.047 | 0.018 | -2.667 | 0.008 |
| Total R² | 1.7% |  |  |  |  |  |
| Sample Size | 757 |  |  |  |  |  |
| Listwise Deletion | 1 |  |  |  |  |  |
| *Objectification by the Self* |  |  |  |  |  |  |
| Intercept |  |  | 1.433 | 0.170 | 8.449 | < 0.001 |
| Anxiety | 2.8% | 19.1% | 0.038 | 0.015 | 2.479 | 0.013 |
| Guilt | 3.4% | 23.9% | 0.053 | 0.015 | 3.513 | < 0.001 |
| Shame | 0.8% | 5.5% | 0.011 | 0.020 | 0.539 | 0.590 |
| Depression | 1.5% | 10.2% | -0.004 | 0.020 | -0.214 | 0.830 |
| Core Self-Evaluations | 1.8% | 12.2% | -0.059 | 0.029 | -2.041 | 0.042 |
| Weight-Bias Internalization | 0.5% | 3.6% | -0.038 | 0.024 | -1.591 | 0.112 |
| Objectified Body Consc. | 1.8% | 12.6% | 0.037 | 0.014 | 2.755 | 0.006 |
| Intuitive Eating Trust | 0.3% | 2.1% | 0.010 | 0.012 | 0.821 | 0.412 |
| Body Dissatisfaction | 1.6% | 10.9% | -0.029 | 0.009 | -3.270 | 0.001 |
| Total R² | 14.4% |  |  |  |  |  |
| Sample Size | 735 |  |  |  |  |  |
| Listwise Deletion | 23 |  |  |  |  |  |
| *Objectification by Others* |  |  |  |  |  |  |
| Intercept |  |  | 1.055 | 0.085 | 12.406 | < 0.001 |
| Anti-fat Dislike | 4.3% | 35.2% | 0.068 | 0.030 | 2.252 | 0.025 |
| Anti-fat Fear | 3.6% | 29.1% | 0.068 | 0.036 | 1.875 | 0.062 |
| Anti-fat Willpower | 1.2% | 10.2% | -0.058 | 0.026 | -2.225 | 0.027 |
| Thin Ideal | 0.7% | 5.7% | 0.005 | 0.022 | 0.238 | 0.812 |
| Negative Body Talk Other | 2.0% | 16.4% | 0.025 | 0.030 | 0.853 | 0.394 |
| Negative Body Talk Self | 0.4% | 3.5% | 0.017 | 0.026 | 0.648 | 0.518 |
| Total R² | 12.2% |  |  |  |  |  |
| Sample Size | 298 |  |  |  |  |  |
| Listwise Deletion | 1 |  |  |  |  |  |
| *Job Context* |  |  |  |  |  |  |
| Intercept |  |  | 1.457 | 0.204 | 7.139 | < 0.001 |
| Job Task | 0.4% | 45.5% | -0.119 | 0.058 | -2.071 | 0.039 |
| Job Social | 0.5% | 52.6% | 0.095 | 0.044 | 2.171 | 0.030 |
| Job Physical | 0.0% | 1.9% | 0.019 | 0.035 | 0.555 | 0.579 |
| Total R² | 0.9% |  |  |  |  |  |
| Sample Size | 756 |  |  |  |  |  |
| Listwise Deletion | 2 |  |  |  |  |  |

Same notes as S3 Table.
