# Supplementary material for: An exploratory study of the correlates of weight-based mistreatment at work
Source: PLoS One. 2026 Aug 26;21(8):e0342144. doi: 10.1371/journal.pone.0342144 (PMC13514019; doi:10.1371/journal.pone.0342144)
Supplement: Supplementary file 5 [file pone.0342144.s005.docx]

**S5 Table. Ordinary Least-Squares Regression and Dominance Analysis for Personal Incivility WBM.**

| **Correlates** | **GD** | **RGD** | **B** | **SE** | **t** | **p** |
| --- | --- | --- | --- | --- | --- | --- |
| *Stereotypes* |  |  |  |  |  |  |
| Intercept |  |  | 1.757 | 0.130 | 13.467 | < 0.001 |
| Yourself Competent | 1.2% | 20.0% | -0.031 | 0.022 | -1.395 | 0.164 |
| Others Competent | 0.9% | 15.6% | -0.012 | 0.027 | -0.430 | 0.667 |
| Coworker Competent | 2.7% | 44.8% | -0.057 | 0.028 | -2.040 | 0.042 |
| Yourself Warm | 0.1% | 1.8% | 0.016 | 0.028 | 0.578 | 0.564 |
| Others Warm | 0.1% | 2.1% | -0.005 | 0.031 | -0.169 | 0.866 |
| Coworker Warm | 0.9% | 15.6% | -0.019 | 0.030 | -0.628 | 0.531 |
| Total R^2^ | 5.9% |  |  |  |  |  |
| Sample Size | 293 |  |  |  |  |  |
| Listwise Deletion | 6 |  |  |  |  |  |
| *Interpersonal Climate* |  |  |  |  |  |  |
| Intercept |  |  | 1.261 | 0.123 | 10.287 | < 0.001 |
| Climate Incl. Equitable | 0.8% | 4.3% | 0.003 | 0.027 | 0.096 | 0.924 |
| Climate Incl. Integration | 2.4% | 12.6% | -0.068 | 0.034 | -1.996 | 0.047 |
| Climate Incl. DM | 1.0% | 5.0% | 0.022 | 0.029 | 0.750 | 0.454 |
| Civility Climate | 1.7% | 8.9% | -0.002 | 0.027 | -0.079 | 0.937 |
| Leader-Member Exchange | 1.8% | 9.0% | -0.024 | 0.025 | -0.934 | 0.351 |
| Perceived Org. Support | 1.5% | 7.9% | -0.002 | 0.027 | -0.063 | 0.950 |
| Interpersonal Conflict | 10.2% | 52.3% | 0.143 | 0.026 | 5.435 | < 0.001 |
| Total R² | 19.5% |  |  |  |  |  |
| Sample Size | 299 |  |  |  |  |  |
| Listwise Deletion | 0 |  |  |  |  |  |
| *Weight Control Climate* |  |  |  |  |  |  |
| Intercept |  |  | 0.804 | 0.089 | 9.014 | < 0.001 |
| Tolerate Neg. Wgt. Talk | 8.0% | 49.2% | 0.081 | 0.019 | 4.158 | < 0.001 |
| Pressure for Thinness | 2.3% | 14.2% | 0.028 | 0.023 | 1.198 | 0.232 |
| Climate for Exercise | 0.2% | 1.4% | 0.023 | 0.035 | 0.648 | 0.517 |
| Wgt. Org. Support | 0.1% | 0.7% | 0.009 | 0.028 | 0.317 | 0.752 |
| Wgt. Social Support | 0.6% | 3.5% | -0.059 | 0.029 | -2.042 | 0.042 |
| Wgt. Diet Norms | 0.6% | 4.0% | 0.051 | 0.030 | 1.723 | 0.086 |
| Wgt. Belittle | 4.4% | 27.1% | 0.062 | 0.021 | 2.994 | 0.003 |
| Total R² | 16.4% |  |  |  |  |  |
| Sample Size | 299 |  |  |  |  |  |
| Listwise Deletion | 0 |  |  |  |  |  |
| *Human Resources Policies* |  |  |  |  |  |  |
| Intercept |  |  | 1.360 | 0.055 | 24.868 | < 0.001 |
| Offer Exercise Program | 0.2% | 6.9% | -0.027 | 0.069 | -0.393 | 0.694 |
| Offer Weight Loss | 0.6% | 24.6% | 0.097 | 0.069 | 1.420 | 0.157 |
| Offer Maintain Health | 0.9% | 41.6% | -0.089 | 0.061 | -1.469 | 0.143 |
| Comm. Exercise Program | 0.1% | 5.9% | 0.020 | 0.046 | 0.424 | 0.672 |
| Comm. Weight Loss | 0.1% | 6.5% | 0.022 | 0.046 | 0.485 | 0.628 |
| Comm. Maintain Health | 0.3% | 14.4% | -0.032 | 0.038 | -0.849 | 0.397 |
| Total R² | 2.3% |  |  |  |  |  |
| Sample Size | 249 |  |  |  |  |  |
| Listwise Deletion | 50 |  |  |  |  |  |
| *Social Status* |  |  |  |  |  |  |
| Intercept |  |  | 1.238 | 0.114 | 10.859 | < 0.001 |
| Education Breakdown | 1.8% | 10.3% | -0.040 | 0.012 | -3.261 | 0.001 |
| Weight Comparison | 1.1% | 6.3% | -0.038 | 0.013 | -3.058 | 0.002 |
| Gender Breakdown | 0.2% | 1.1% | 0.008 | 0.006 | 1.326 | 0.185 |
| Manager | 1.8% | 10.3% | 0.097 | 0.031 | 3.116 | 0.002 |
| Visible Disability | 5.9% | 34.5% | 0.088 | 0.018 | 5.035 | < 0.001 |
| Ethnic Name | 2.7% | 16.1% | 0.040 | 0.016 | 2.497 | 0.013 |
| Body-Mass Index | 0.0% | 0.2% | < 0.001 | 0.002 | 0.080 | 0.937 |
| Accent | 3.1% | 18.2% | 0.044 | 0.016 | 2.768 | 0.006 |
| Gender | 0.3% | 1.6% | 0.037 | 0.031 | 1.200 | 0.231 |
| Race | 0.2% | 1.4% | 0.044 | 0.036 | 1.235 | 0.217 |
| Total R² | 17.1% |  |  |  |  |  |
| Sample Size | 591 |  |  |  |  |  |
| Listwise Deletion | 167 |  |  |  |  |  |
| *Identity Management Strategies* |  |  |  |  |  |  |
| Intercept |  |  | 1.315 | 0.056 | 23.331 | < 0.001 |
| Redefinition Reassociate | 0.8% | 31.4% | 0.028 | 0.016 | 1.748 | 0.081 |
| Redefinition Regenerate | 1.1% | 40.8% | 0.048 | 0.020 | 2.386 | 0.017 |
| Switch Deemphasize | 0.1% | 4.6% | 0.017 | 0.023 | 0.745 | 0.456 |
| Switch Emphasize | 0.6% | 23.2% | -0.051 | 0.019 | -2.678 | 0.008 |
| Total R² | 2.6% |  |  |  |  |  |
| Sample Size | 756 |  |  |  |  |  |
| Listwise Deletion | 2 |  |  |  |  |  |
| *Objectification by the Self* |  |  |  |  |  |  |
| Intercept |  |  | 1.484 | 0.183 | 8.116 | < 0.001 |
| Anxiety | 4.3% | 23.9% | 0.065 | 0.017 | 3.927 | < 0.001 |
| Guilt | 4.1% | 22.7% | 0.063 | 0.016 | 3.907 | < 0.001 |
| Shame | 1.2% | 6.7% | 0.030 | 0.021 | 1.423 | 0.155 |
| Depression | 1.6% | 8.7% | -0.028 | 0.022 | -1.288 | 0.198 |
| Core Self-Evaluations | 2.4% | 13.3% | -0.089 | 0.031 | -2.833 | 0.005 |
| Weight-Bias Internalization | 0.9% | 5.1% | -0.070 | 0.026 | -2.725 | 0.007 |
| Objectified Body Consc. | 1.5% | 8.4% | 0.032 | 0.015 | 2.191 | 0.029 |
| Intuitive Eating Trust | 0.6% | 3.3% | 0.019 | 0.013 | 1.500 | 0.134 |
| Body Dissatisfaction | 1.4% | 7.9% | -0.028 | 0.010 | -2.942 | 0.003 |
| Total R² | 18.0% |  |  |  |  |  |
| Sample Size | 734 |  |  |  |  |  |
| Listwise Deletion | 24 |  |  |  |  |  |
| *Objectification by Others* |  |  |  |  |  |  |
| Intercept |  |  | 0.947 | 0.087 | 10.948 | < 0.001 |
| Anti-fat Dislike | 6.5% | 40.5% | 0.099 | 0.031 | 3.220 | 0.001 |
| Anti-fat Fear | 4.0% | 24.9% | 0.057 | 0.037 | 1.547 | 0.123 |
| Anti-fat Willpower | 1.4% | 8.8% | -0.059 | 0.027 | -2.218 | 0.027 |
| Thin Ideal | 1.5% | 9.5% | 0.024 | 0.022 | 1.091 | 0.276 |
| Negative Body Talk Other | 2.2% | 13.7% | 0.017 | 0.030 | 0.564 | 0.573 |
| Negative Body Talk Self | 0.4% | 2.6% | 0.011 | 0.026 | 0.427 | 0.670 |
| Total R² | 16.1% |  |  |  |  |  |
| Sample Size | 299 |  |  |  |  |  |
| Listwise Deletion | 0 |  |  |  |  |  |
| *Job Context* |  |  |  |  |  |  |
| Intercept |  |  | 1.518 | 0.227 | 6.693 | < 0.001 |
| Job Task | 0.3% | 64.5% | -0.115 | 0.064 | -1.784 | 0.075 |
| Job Social | 0.2% | 34.4% | 0.068 | 0.048 | 1.410 | 0.159 |
| Job Physical | 0.0% | 1.1% | 0.012 | 0.038 | 0.312 | 0.755 |
| Total R² | 0.5% |  |  |  |  |  |
| Sample Size | 755 |  |  |  |  |  |
| Listwise Deletion | 3 |  |  |  |  |  |

Same notes as S3 Table.
