# Supplementary material for: An exploratory study of the correlates of weight-based mistreatment at work
Source: PLoS One. 2026 Aug 26;21(8):e0342144. doi: 10.1371/journal.pone.0342144 (PMC13514019; doi:10.1371/journal.pone.0342144)
Supplement: Supplementary file 6 [file pone.0342144.s006.docx]

**S6 Table. Ordinary Least-Squares Regression and Dominance Analysis for Physical Incivility WBM.**

| **Correlates** | **GD** | **RGD** | **B** | **SE** | **t** | **p** |
| --- | --- | --- | --- | --- | --- | --- |
| *Stereotypes* |  |  |  |  |  |  |
| Intercept |  |  | 1.666 | 0.106 | 15.779 | < 0.001 |
| Yourself Competent | 0.9% | 10.9% | -0.018 | 0.018 | -1.014 | 0.311 |
| Others Competent | 0.9% | 11.1% | -0.007 | 0.022 | -0.307 | 0.759 |
| Coworker Competent | 3.5% | 44.9% | -0.051 | 0.023 | -2.262 | 0.024 |
| Yourself Warm | 0.2% | 3.2% | -0.001 | 0.022 | -0.061 | 0.951 |
| Others Warm | 0.3% | 3.2% | -0.004 | 0.025 | -0.149 | 0.882 |
| Coworker Warm | 2.1% | 26.7% | -0.032 | 0.025 | -1.292 | 0.197 |
| Total R^2^ | 7.9% |  |  |  |  |  |
| Sample Size | 293 |  |  |  |  |  |
| Listwise Deletion | 6 |  |  |  |  |  |
| *Interpersonal Climate* |  |  |  |  |  |  |
| Intercept |  |  | 1.080 | 0.100 | 10.840 | < 0.001 |
| Climate Incl. Equitable | 0.7% | 3.4% | -0.007 | 0.022 | -0.330 | 0.742 |
| Climate Incl. Integration | 1.7% | 7.9% | -0.048 | 0.028 | -1.752 | 0.081 |
| Climate Incl. DM | 0.7% | 3.4% | 0.032 | 0.023 | 1.381 | 0.168 |
| Civility Climate | 1.8% | 8.3% | -0.009 | 0.022 | -0.426 | 0.671 |
| Leader-Member Exchange | 3.0% | 14.2% | -0.050 | 0.021 | -2.415 | 0.016 |
| Perceived Org. Support | 1.0% | 4.8% | 0.039 | 0.022 | 1.767 | 0.078 |
| Interpersonal Conflict | 12.4% | 58.1% | 0.134 | 0.021 | 6.255 | < 0.001 |
| Total R² | 21.4% |  |  |  |  |  |
| Sample Size | 299 |  |  |  |  |  |
| Listwise Deletion | 0 |  |  |  |  |  |
| *Weight Control Climate* |  |  |  |  |  |  |
| Intercept |  |  | 0.888 | 0.076 | 11.621 | < 0.001 |
| Tolerate Neg. Wgt. Talk | 4.3% | 45.8% | 0.049 | 0.017 | 2.946 | 0.003 |
| Pressure for Thinness | 1.1% | 11.3% | 0.013 | 0.020 | 0.652 | 0.515 |
| Climate for Exercise | 0.2% | 2.6% | 0.028 | 0.030 | 0.925 | 0.356 |
| Wgt. Org. Support | 0.1% | 1.0% | -0.010 | 0.024 | -0.425 | 0.671 |
| Wgt. Social Support | 0.5% | 5.6% | -0.046 | 0.025 | -1.871 | 0.062 |
| Wgt. Diet Norms | 0.8% | 8.3% | 0.047 | 0.025 | 1.870 | 0.063 |
| Wgt. Belittle | 2.4% | 25.5% | 0.039 | 0.018 | 2.182 | 0.030 |
| Total R² | 9.4% |  |  |  |  |  |
| Sample Size | 299 |  |  |  |  |  |
| Listwise Deletion | 0 |  |  |  |  |  |
| *Human Resources Policies* |  |  |  |  |  |  |
| Intercept |  |  | 1.229 | 0.046 | 26.790 | < 0.001 |
| Offer Exercise Program | 0.1% | 5.9% | -0.033 | 0.058 | -0.578 | 0.564 |
| Offer Weight Loss | 0.4% | 19.1% | 0.066 | 0.057 | 1.144 | 0.254 |
| Offer Maintain Health | 0.2% | 7.4% | -0.022 | 0.051 | -0.436 | 0.663 |
| Comm. Exercise Program | 0.3% | 12.4% | -0.028 | 0.039 | -0.735 | 0.463 |
| Comm. Weight Loss | 0.5% | 26.2% | 0.056 | 0.038 | 1.476 | 0.141 |
| Comm. Maintain Health | 0.6% | 29.0% | -0.039 | 0.032 | -1.244 | 0.215 |
| Total R² | 2.1% |  |  |  |  |  |
| Sample Size | 249 |  |  |  |  |  |
| Listwise Deletion | 50 |  |  |  |  |  |
| *Social Status* |  |  |  |  |  |  |
| Intercept |  |  | 1.059 | 0.102 | 10.382 | < 0.001 |
| Education Breakdown | 2.2% | 11.2% | -0.041 | 0.011 | -3.684 | < 0.001 |
| Weight Comparison | 0.1% | 0.6% | -0.014 | 0.011 | -1.213 | 0.226 |
| Gender Breakdown | 0.2% | 0.8% | 0.008 | 0.006 | 1.365 | 0.173 |
| Manager | 1.1% | 5.6% | 0.066 | 0.028 | 2.362 | 0.019 |
| Visible Disability | 6.8% | 33.9% | 0.083 | 0.016 | 5.279 | < 0.001 |
| Ethnic Name | 4.3% | 21.6% | 0.046 | 0.014 | 3.258 | 0.001 |
| Body-Mass Index | 0.1% | 0.3% | 0.001 | 0.002 | 0.697 | 0.486 |
| Accent | 5.0% | 25.1% | 0.052 | 0.014 | 3.692 | < 0.001 |
| Gender | 0.1% | 0.3% | 0.011 | 0.028 | 0.404 | 0.686 |
| Race | 0.1% | 0.7% | 0.032 | 0.032 | 0.986 | 0.324 |
| Total R² | 19.9% |  |  |  |  |  |
| Sample Size | 591 |  |  |  |  |  |
| Listwise Deletion | 167 |  |  |  |  |  |
| *Identity Management Strategies* |  |  |  |  |  |  |
| Intercept |  |  | 1.178 | 0.052 | 22.823 | < 0.001 |
| Redefinition Reassociate | 1.5% | 36.6% | 0.036 | 0.015 | 2.469 | 0.014 |
| Redefinition Regenerate | 1.6% | 39.7% | 0.052 | 0.018 | 2.831 | 0.005 |
| Switch Deemphasize | 0.2% | 4.5% | 0.018 | 0.021 | 0.880 | 0.379 |
| Switch Emphasize | 0.8% | 19.3% | -0.055 | 0.018 | -3.125 | 0.002 |
| Total R² | 4.0% |  |  |  |  |  |
| Sample Size | 756 |  |  |  |  |  |
| Listwise Deletion | 2 |  |  |  |  |  |
| *Objectification by the Self* |  |  |  |  |  |  |
| Intercept |  |  | 1.596 | 0.170 | 9.364 | < 0.001 |
| Anxiety | 3.0% | 19.0% | 0.051 | 0.015 | 3.298 | 0.001 |
| Guilt | 3.7% | 22.9% | 0.064 | 0.015 | 4.227 | < 0.001 |
| Shame | 0.4% | 2.3% | 0.004 | 0.020 | 0.183 | 0.855 |
| Depression | 0.9% | 5.7% | -0.021 | 0.020 | -1.017 | 0.309 |
| Core Self-Evaluations | 1.5% | 9.3% | -0.080 | 0.029 | -2.729 | 0.007 |
| Weight-Bias Internalization | 1.7% | 10.5% | -0.094 | 0.024 | -3.913 | < 0.001 |
| Objectified Body Consc. | 1.4% | 8.5% | 0.031 | 0.014 | 2.296 | 0.022 |
| Intuitive Eating Trust | 0.6% | 3.6% | 0.010 | 0.012 | 0.797 | 0.426 |
| Body Dissatisfaction | 2.9% | 18.3% | -0.037 | 0.009 | -4.195 | < 0.001 |
| Total R² | 16.0% |  |  |  |  |  |
| Sample Size | 734 |  |  |  |  |  |
| Listwise Deletion | 24 |  |  |  |  |  |
| *Objectification by Others* |  |  |  |  |  |  |
| Intercept |  |  | 0.967 | 0.074 | 13.115 | < 0.001 |
| Anti-fat Dislike | 3.9% | 38.2% | 0.061 | 0.026 | 2.339 | 0.020 |
| Anti-fat Fear | 2.0% | 19.2% | 0.024 | 0.032 | 0.762 | 0.447 |
| Anti-fat Willpower | 1.0% | 9.8% | -0.046 | 0.023 | -2.021 | 0.044 |
| Thin Ideal | 0.6% | 5.7% | 0.009 | 0.019 | 0.460 | 0.646 |
| Negative Body Talk Other | 2.6% | 25.4% | 0.043 | 0.026 | 1.680 | 0.094 |
| Negative Body Talk Self | 0.2% | 1.7% | 0.003 | 0.023 | 0.122 | 0.903 |
| Total R² | 10.2% |  |  |  |  |  |
| Sample Size | 299 |  |  |  |  |  |
| Listwise Deletion | 0 |  |  |  |  |  |
| *Job Context* |  |  |  |  |  |  |
| Intercept |  |  | 1.341 | 0.209 | 6.423 | < 0.001 |
| Job Task | 0.3% | 50.4% | -0.098 | 0.059 | -1.651 | 0.099 |
| Job Social | 0.3% | 48.8% | 0.072 | 0.044 | 1.633 | 0.103 |
| Job Physical | 0.0% | 0.8% | 0.009 | 0.035 | 0.242 | 0.809 |
| Total R² | 0.5% |  |  |  |  |  |
| Sample Size | 755 |  |  |  |  |  |
| Listwise Deletion | 3 |  |  |  |  |  |

Same notes as S3 Table.
