# Supplementary material for: An exploratory study of the correlates of weight-based mistreatment at work
Source: PLoS One. 2026 Aug 26;21(8):e0342144. doi: 10.1371/journal.pone.0342144 (PMC13514019; doi:10.1371/journal.pone.0342144)
Supplement: Supplementary file 7 [file pone.0342144.s007.docx]

**S7 Table. Ordinary Least-Squares Regression and Dominance Analysis for Work Incivility WBM.**

| **Correlates** | **GD** | **RGD** | **B** | **SE** | **t** | **p** |
| --- | --- | --- | --- | --- | --- | --- |
| *Stereotypes* |  |  |  |  |  |  |
| Intercept |  |  | 1.849 | 0.134 | 13.814 | < 0.001 |
| Yourself Competent | 1.9% | 25.3% | -0.045 | 0.023 | -1.960 | 0.051 |
| Others Competent | 1.0% | 13.3% | -0.014 | 0.027 | -0.494 | 0.622 |
| Coworker Competent | 2.6% | 34.4% | -0.047 | 0.029 | -1.653 | 0.099 |
| Yourself Warm | 0.1% | 1.8% | 0.018 | 0.028 | 0.621 | 0.535 |
| Others Warm | 0.2% | 2.0% | 0.017 | 0.032 | 0.530 | 0.597 |
| Coworker Warm | 1.7% | 23.2% | -0.046 | 0.031 | -1.486 | 0.138 |
| Total R^2^ | 7.5% |  |  |  |  |  |
| Sample Size | 293 |  |  |  |  |  |
| Listwise Deletion | 6 |  |  |  |  |  |
| *Interpersonal Climate* |  |  |  |  |  |  |
| Intercept |  |  | 1.370 | 0.128 | 10.673 | < 0.001 |
| Climate Incl. Equitable | 1.4% | 8.6% | -0.026 | 0.029 | -0.890 | 0.374 |
| Climate Incl. Integration | 3.1% | 19.9% | -0.096 | 0.036 | -2.688 | 0.008 |
| Climate Incl. DM | 1.1% | 6.9% | 0.067 | 0.030 | 2.219 | 0.027 |
| Civility Climate | 0.9% | 5.8% | 0.020 | 0.028 | 0.698 | 0.486 |
| Leader-Member Exchange | 1.3% | 8.4% | -0.025 | 0.027 | -0.926 | 0.355 |
| Perceived Org. Support | 1.3% | 8.2% | -0.014 | 0.028 | -0.484 | 0.629 |
| Interpersonal Conflict | 6.6% | 42.1% | 0.119 | 0.028 | 4.327 | < 0.001 |
| Total R² | 15.7% |  |  |  |  |  |
| Sample Size | 299 |  |  |  |  |  |
| Listwise Deletion | 0 |  |  |  |  |  |
| *Weight Control Climate* |  |  |  |  |  |  |
| Intercept |  |  | 0.831 | 0.093 | 8.978 | < 0.001 |
| Tolerate Neg. Wgt. Talk | 6.1% | 43.5% | 0.071 | 0.020 | 3.509 | 0.001 |
| Pressure for Thinness | 2.5% | 17.5% | 0.034 | 0.024 | 1.385 | 0.167 |
| Climate for Exercise | 0.4% | 2.8% | 0.016 | 0.037 | 0.425 | 0.671 |
| Wgt. Org. Support | 0.3% | 2.0% | 0.011 | 0.029 | 0.372 | 0.710 |
| Wgt. Social Support | 0.4% | 2.9% | -0.044 | 0.030 | -1.472 | 0.142 |
| Wgt. Diet Norms | 1.4% | 9.7% | 0.065 | 0.031 | 2.130 | 0.034 |
| Wgt. Belittle | 3.0% | 21.6% | 0.053 | 0.021 | 2.448 | 0.015 |
| Total R² | 14.1% |  |  |  |  |  |
| Sample Size | 299 |  |  |  |  |  |
| Listwise Deletion | 0 |  |  |  |  |  |
| *Human Resources Policies* |  |  |  |  |  |  |
| Intercept |  |  | 1.391 | 0.055 | 25.264 | < 0.001 |
| Offer Exercise Program | 0.1% | 4.6% | 0.006 | 0.069 | 0.091 | 0.927 |
| Offer Weight Loss | 0.3% | 9.1% | 0.054 | 0.069 | 0.784 | 0.434 |
| Offer Maintain Health | 1.0% | 32.5% | -0.097 | 0.061 | -1.592 | 0.113 |
| Comm. Exercise Program | 0.4% | 14.0% | 0.028 | 0.046 | 0.598 | 0.551 |
| Comm. Weight Loss | 0.7% | 21.9% | 0.053 | 0.046 | 1.154 | 0.250 |
| Comm. Maintain Health | 0.6% | 17.9% | -0.048 | 0.038 | -1.261 | 0.209 |
| Total R² | 3.1% |  |  |  |  |  |
| Sample Size | 249 |  |  |  |  |  |
| Listwise Deletion | 50 |  |  |  |  |  |
| *Social Status* |  |  |  |  |  |  |
| Intercept |  |  | 1.216 | 0.114 | 10.674 | < 0.001 |
| Education Breakdown | 1.6% | 10.1% | -0.037 | 0.012 | -2.993 | 0.003 |
| Weight Comparison | 0.6% | 3.7% | -0.028 | 0.013 | -2.200 | 0.028 |
| Gender Breakdown | 0.1% | 0.7% | 0.006 | 0.006 | 0.951 | 0.342 |
| Manager | 1.1% | 7.2% | 0.081 | 0.031 | 2.618 | 0.009 |
| Visible Disability | 3.8% | 24.3% | 0.063 | 0.018 | 3.604 | < 0.001 |
| Ethnic Name | 3.6% | 22.5% | 0.049 | 0.016 | 3.066 | 0.002 |
| Body-Mass Index | 0.2% | 1.0% | 0.002 | 0.002 | 1.042 | 0.298 |
| Accent | 3.9% | 24.8% | 0.052 | 0.016 | 3.300 | 0.001 |
| Gender | 0.8% | 5.4% | 0.071 | 0.031 | 2.291 | 0.022 |
| Race | 0.1% | 0.4% | 0.007 | 0.036 | 0.190 | 0.849 |
| Total R² | 15.8% |  |  |  |  |  |
| Sample Size | 591 |  |  |  |  |  |
| Listwise Deletion | 167 |  |  |  |  |  |
| *Identity Management Strategies* |  |  |  |  |  |  |
| Intercept |  |  | 1.375 | 0.056 | 24.463 | < 0.001 |
| Redefinition Reassociate | 0.9% | 27.0% | 0.028 | 0.016 | 1.768 | 0.077 |
| Redefinition Regenerate | 1.6% | 45.0% | 0.062 | 0.020 | 3.107 | 0.002 |
| Switch Deemphasize | 0.1% | 3.6% | 0.010 | 0.023 | 0.433 | 0.665 |
| Switch Emphasize | 0.8% | 24.3% | -0.059 | 0.019 | -3.067 | 0.002 |
| Total R² | 3.5% |  |  |  |  |  |
| Sample Size | 756 |  |  |  |  |  |
| Listwise Deletion | 2 |  |  |  |  |  |
| *Objectification by the Self* |  |  |  |  |  |  |
| Intercept |  |  | 1.308 | 0.186 | 7.021 | < 0.001 |
| Anxiety | 2.9% | 18.4% | 0.051 | 0.017 | 3.052 | 0.002 |
| Guilt | 4.0% | 25.4% | 0.069 | 0.016 | 4.200 | < 0.001 |
| Shame | 0.9% | 5.5% | 0.024 | 0.022 | 1.086 | 0.278 |
| Depression | 1.1% | 6.8% | -0.036 | 0.022 | -1.632 | 0.103 |
| Core Self-Evaluations | 1.4% | 8.9% | -0.059 | 0.032 | -1.860 | 0.063 |
| Weight-Bias Internalization | 0.4% | 2.8% | -0.033 | 0.026 | -1.278 | 0.202 |
| Objectified Body Consc. | 2.6% | 16.6% | 0.053 | 0.015 | 3.531 | < 0.001 |
| Intuitive Eating Trust | 1.0% | 6.3% | 0.028 | 0.013 | 2.143 | 0.032 |
| Body Dissatisfaction | 1.5% | 9.3% | -0.029 | 0.010 | -2.933 | 0.003 |
| Total R² | 15.9% |  |  |  |  |  |
| Sample Size | 734 |  |  |  |  |  |
| Listwise Deletion | 24 |  |  |  |  |  |
| *Objectification by Others* |  |  |  |  |  |  |
| Intercept |  |  | 1.014 | 0.092 | 11.058 | < 0.001 |
| Anti-fat Dislike | 4.3% | 41.9% | 0.090 | 0.033 | 2.755 | 0.006 |
| Anti-fat Fear | 1.8% | 17.3% | 0.005 | 0.039 | 0.135 | 0.893 |
| Anti-fat Willpower | 0.7% | 7.1% | -0.028 | 0.028 | -0.979 | 0.328 |
| Thin Ideal | 1.3% | 12.9% | 0.028 | 0.023 | 1.212 | 0.226 |
| Negative Body Talk Other | 1.6% | 16.0% | 0.022 | 0.032 | 0.689 | 0.491 |
| Negative Body Talk Self | 0.5% | 4.8% | 0.019 | 0.028 | 0.679 | 0.498 |
| Total R² | 10.2% |  |  |  |  |  |
| Sample Size | 299 |  |  |  |  |  |
| Listwise Deletion | 0 |  |  |  |  |  |
| *Job Context* |  |  |  |  |  |  |
| Intercept |  |  | 1.644 | 0.227 | 7.252 | < 0.001 |
| Job Task | 0.3% | 88.6% | -0.100 | 0.064 | -1.548 | 0.122 |
| Job Social | 0.0% | 10.2% | 0.033 | 0.048 | 0.690 | 0.490 |
| Job Physical | 0.0% | 1.2% | 0.009 | 0.039 | 0.238 | 0.812 |
| Total R² | 0.3% |  |  |  |  |  |
| Sample Size | 755 |  |  |  |  |  |
| Listwise Deletion | 3 |  |  |  |  |  |

Same notes as S3 Table.
