# Supplementary material for: An exploratory study of the correlates of weight-based mistreatment at work
Source: PLoS One. 2026 Aug 26;21(8):e0342144. doi: 10.1371/journal.pone.0342144 (PMC13514019; doi:10.1371/journal.pone.0342144)
Supplement: Supplementary file 8 [file pone.0342144.s008.docx]

**S8 Table. Ordinary Least-Squares Regression and Dominance Analysis for Micro-Assault WBM.**

| **Correlates** | **GD** | **RGD** | **B** | **SE** | **t** | **p** |
| --- | --- | --- | --- | --- | --- | --- |
| *Stereotypes* |  |  |  |  |  |  |
| Intercept |  |  | 1.851 | 0.121 | 15.323 | < 0.001 |
| Yourself Competent | 0.2% | 3.4% | 0.004 | 0.021 | 0.179 | 0.858 |
| Others Competent | 1.6% | 26.2% | -0.036 | 0.025 | -1.470 | 0.143 |
| Coworker Competent | 2.7% | 44.3% | -0.049 | 0.026 | -1.912 | 0.057 |
| Yourself Warm | 0.1% | 2.2% | 0.017 | 0.026 | 0.660 | 0.510 |
| Others Warm | 0.2% | 3.1% | -0.004 | 0.029 | -0.142 | 0.887 |
| Coworker Warm | 1.3% | 20.8% | -0.025 | 0.028 | -0.872 | 0.384 |
| Total R^2^ | 6.1% |  |  |  |  |  |
| Sample Size | 293 |  |  |  |  |  |
| Listwise Deletion | 6 |  |  |  |  |  |
| *Interpersonal Climate* |  |  |  |  |  |  |
| Intercept |  |  | 1.349 | 0.115 | 11.678 | < 0.001 |
| Climate Incl. Equitable | 0.3% | 1.9% | -0.002 | 0.026 | -0.062 | 0.951 |
| Climate Incl. Integration | 0.5% | 3.3% | -0.008 | 0.032 | -0.261 | 0.794 |
| Climate Incl. DM | 0.4% | 2.5% | 0.018 | 0.027 | 0.663 | 0.508 |
| Civility Climate | 3.8% | 22.7% | -0.056 | 0.025 | -2.230 | 0.027 |
| Leader-Member Exchange | 0.9% | 5.6% | -0.003 | 0.024 | -0.135 | 0.893 |
| Perceived Org. Support | 1.1% | 6.5% | 0.008 | 0.026 | 0.330 | 0.742 |
| Interpersonal Conflict | 9.6% | 57.6% | 0.126 | 0.025 | 5.063 | < 0.001 |
| Total R² | 16.7% |  |  |  |  |  |
| Sample Size | 299 |  |  |  |  |  |
| Listwise Deletion | 0 |  |  |  |  |  |
| *Weight Control Climate* |  |  |  |  |  |  |
| Intercept |  |  | 1.033 | 0.083 | 12.519 | < 0.001 |
| Tolerate Neg. Wgt. Talk | 8.4% | 51.3% | 0.078 | 0.018 | 4.323 | < 0.001 |
| Pressure for Thinness | 3.5% | 21.4% | 0.047 | 0.022 | 2.162 | 0.031 |
| Climate for Exercise | 0.2% | 1.3% | -0.020 | 0.033 | -0.613 | 0.540 |
| Wgt. Org. Support | 0.5% | 2.8% | 0.037 | 0.026 | 1.412 | 0.159 |
| Wgt. Social Support | 0.7% | 4.1% | -0.057 | 0.027 | -2.114 | 0.035 |
| Wgt. Diet Norms | 0.7% | 4.4% | 0.047 | 0.027 | 1.728 | 0.085 |
| Wgt. Belittle | 2.4% | 14.6% | 0.034 | 0.019 | 1.788 | 0.075 |
| Total R² | 16.4% |  |  |  |  |  |
| Sample Size | 299 |  |  |  |  |  |
| Listwise Deletion | 0 |  |  |  |  |  |
| *Human Resources Policies* |  |  |  |  |  |  |
| Intercept |  |  | 1.486 | 0.050 | 29.492 | < 0.001 |
| Offer Exercise Program | 0.2% | 6.5% | -0.051 | 0.064 | -0.805 | 0.421 |
| Offer Weight Loss | 1.3% | 36.2% | 0.134 | 0.063 | 2.121 | 0.035 |
| Offer Maintain Health | 0.5% | 15.4% | -0.054 | 0.056 | -0.973 | 0.332 |
| Comm. Exercise Program | 0.1% | 3.5% | 0.009 | 0.043 | 0.215 | 0.830 |
| Comm. Weight Loss | 0.3% | 7.3% | 0.033 | 0.042 | 0.793 | 0.428 |
| Comm. Maintain Health | 1.1% | 31.0% | -0.060 | 0.035 | -1.732 | 0.084 |
| Total R² | 3.6% |  |  |  |  |  |
| Sample Size | 249 |  |  |  |  |  |
| Listwise Deletion | 50 |  |  |  |  |  |
| *Social Status* |  |  |  |  |  |  |
| Intercept |  |  | 1.321 | 0.114 | 11.622 | < 0.001 |
| Education Breakdown | 0.4% | 3.9% | -0.018 | 0.012 | -1.478 | 0.140 |
| Weight Comparison | 0.7% | 6.6% | -0.027 | 0.013 | -2.167 | 0.031 |
| Gender Breakdown | 0.0% | 0.2% | 0.003 | 0.006 | 0.496 | 0.620 |
| Manager | 1.6% | 16.1% | 0.094 | 0.031 | 3.019 | 0.003 |
| Visible Disability | 3.1% | 31.2% | 0.060 | 0.017 | 3.439 | 0.001 |
| Ethnic Name | 1.5% | 15.2% | 0.025 | 0.016 | 1.563 | 0.118 |
| Body-Mass Index | 0.3% | 3.3% | 0.003 | 0.002 | 1.470 | 0.142 |
| Accent | 2.3% | 22.5% | 0.040 | 0.016 | 2.553 | 0.011 |
| Gender | 0.1% | 0.6% | 0.018 | 0.031 | 0.592 | 0.554 |
| Race | 0.0% | 0.5% | -0.009 | 0.036 | -0.252 | 0.801 |
| Total R² | 10.1% |  |  |  |  |  |
| Sample Size | 593 |  |  |  |  |  |
| Listwise Deletion | 165 |  |  |  |  |  |
| *Identity Management Strategies* |  |  |  |  |  |  |
| Intercept |  |  | 1.527 | 0.054 | 28.345 | < 0.001 |
| Redefinition Reassociate | 1.1% | 41.5% | 0.042 | 0.015 | 2.788 | 0.005 |
| Redefinition Regenerate | 0.3% | 9.9% | 0.016 | 0.019 | 0.822 | 0.411 |
| Switch Deemphasize | 0.1% | 5.2% | 0.019 | 0.022 | 0.871 | 0.384 |
| Switch Emphasize | 1.2% | 43.5% | -0.061 | 0.018 | -3.324 | 0.001 |
| Total R² | 2.7% |  |  |  |  |  |
| Sample Size | 758 |  |  |  |  |  |
| Listwise Deletion | 0 |  |  |  |  |  |
| *Objectification by the Self* |  |  |  |  |  |  |
| Intercept |  |  | 1.530 | 0.178 | 8.574 | < 0.001 |
| Anxiety | 1.9% | 13.0% | 0.020 | 0.016 | 1.242 | 0.215 |
| Guilt | 3.8% | 26.4% | 0.062 | 0.016 | 3.916 | < 0.001 |
| Shame | 1.2% | 8.3% | 0.034 | 0.021 | 1.644 | 0.101 |
| Depression | 1.5% | 10.6% | 0.007 | 0.021 | 0.308 | 0.758 |
| Core Self-Evaluations | 1.6% | 11.4% | -0.054 | 0.031 | -1.774 | 0.077 |
| Weight-Bias Internalization | 0.7% | 4.9% | -0.060 | 0.025 | -2.373 | 0.018 |
| Objectified Body Consc. | 1.6% | 11.1% | 0.037 | 0.014 | 2.563 | 0.011 |
| Intuitive Eating Trust | 0.2% | 1.7% | 0.006 | 0.013 | 0.504 | 0.615 |
| Body Dissatisfaction | 1.8% | 12.7% | -0.033 | 0.009 | -3.578 | < 0.001 |
| Total R² | 14.3% |  |  |  |  |  |
| Sample Size | 736 |  |  |  |  |  |
| Listwise Deletion | 22 |  |  |  |  |  |
| *Objectification by Others* |  |  |  |  |  |  |
| Intercept |  |  | 1.031 | 0.081 | 12.693 | < 0.001 |
| Anti-fat Dislike | 3.4% | 24.5% | 0.047 | 0.029 | 1.616 | 0.107 |
| Anti-fat Fear | 2.3% | 16.8% | 0.010 | 0.035 | 0.281 | 0.779 |
| Anti-fat Willpower | 1.1% | 8.1% | -0.018 | 0.025 | -0.710 | 0.478 |
| Thin Ideal | 1.3% | 9.1% | 0.018 | 0.021 | 0.870 | 0.385 |
| Negative Body Talk Other | 4.9% | 35.5% | 0.077 | 0.028 | 2.721 | 0.007 |
| Negative Body Talk Self | 0.8% | 5.9% | 0.020 | 0.025 | 0.792 | 0.429 |
| Total R² | 13.8% |  |  |  |  |  |
| Sample Size | 299 |  |  |  |  |  |
| Listwise Deletion | 0 |  |  |  |  |  |
| *Job Context* |  |  |  |  |  |  |
| Intercept |  |  | 1.686 | 0.216 | 7.815 | < 0.001 |
| Job Task | 0.5% | 66.4% | -0.135 | 0.061 | -2.208 | 0.028 |
| Job Social | 0.3% | 32.5% | 0.078 | 0.046 | 1.693 | 0.091 |
| Job Physical | 0.0% | 1.0% | 0.013 | 0.037 | 0.363 | 0.717 |
| Total R² | 0.8% |  |  |  |  |  |
| Sample Size | 757 |  |  |  |  |  |
| Listwise Deletion | 1 |  |  |  |  |  |

Same notes as S3 Table.
