# Supplementary material for: An exploratory study of the correlates of weight-based mistreatment at work
Source: PLoS One. 2026 Aug 26;21(8):e0342144. doi: 10.1371/journal.pone.0342144 (PMC13514019; doi:10.1371/journal.pone.0342144)
Supplement: Supplementary file 9 [file pone.0342144.s009.docx]

**S9 Table. Ordinary Least-Squares Regression and Dominance Analysis for Micro-Insult WBM.**

| **Correlates** | **GD** | **RGD** | **B** | **SE** | **t** | **p** |
| --- | --- | --- | --- | --- | --- | --- |
| *Stereotypes* |  |  |  |  |  |  |
| Intercept |  |  | 1.914 | 0.153 | 12.545 | < 0.001 |
| Yourself Competent | 2.1% | 29.8% | -0.046 | 0.026 | -1.775 | 0.077 |
| Others Competent | 1.6% | 22.8% | -0.035 | 0.031 | -1.106 | 0.270 |
| Coworker Competent | 2.6% | 37.1% | -0.069 | 0.033 | -2.132 | 0.034 |
| Yourself Warm | 0.1% | 1.9% | 0.013 | 0.032 | 0.405 | 0.686 |
| Others Warm | 0.3% | 3.7% | 0.030 | 0.036 | 0.819 | 0.414 |
| Coworker Warm | 0.3% | 4.8% | 0.006 | 0.036 | 0.166 | 0.868 |
| Total R^2^ | 6.9% |  |  |  |  |  |
| Sample Size | 293 |  |  |  |  |  |
| Listwise Deletion | 6 |  |  |  |  |  |
| *Interpersonal Climate* |  |  |  |  |  |  |
| Intercept |  |  | 1.465 | 0.152 | 9.661 | < 0.001 |
| Climate Incl. Equitable | 0.6% | 6.3% | -0.017 | 0.034 | -0.515 | 0.607 |
| Climate Incl. Integration | 0.9% | 9.5% | -0.036 | 0.042 | -0.845 | 0.399 |
| Climate Incl. DM | 0.5% | 4.9% | 0.008 | 0.036 | 0.217 | 0.828 |
| Civility Climate | 1.2% | 12.9% | -0.027 | 0.033 | -0.819 | 0.414 |
| Leader-Member Exchange | 0.7% | 7.3% | -0.015 | 0.031 | -0.469 | 0.640 |
| Perceived Org. Support | 0.5% | 5.4% | 0.029 | 0.034 | 0.876 | 0.382 |
| Interpersonal Conflict | 5.1% | 53.6% | 0.118 | 0.033 | 3.626 | < 0.001 |
| Total R² | 9.4% |  |  |  |  |  |
| Sample Size | 299 |  |  |  |  |  |
| Listwise Deletion | 0 |  |  |  |  |  |
| *Weight Control Climate* |  |  |  |  |  |  |
| Intercept |  |  | 1.092 | 0.107 | 10.243 | < 0.001 |
| Tolerate Neg. Wgt. Talk | 7.5% | 62.6% | 0.097 | 0.023 | 4.178 | < 0.001 |
| Pressure for Thinness | 2.9% | 24.0% | 0.053 | 0.028 | 1.888 | 0.060 |
| Climate for Exercise | 0.2% | 1.9% | -0.042 | 0.042 | -0.990 | 0.323 |
| Wgt. Org. Support | 0.2% | 1.5% | 0.027 | 0.034 | 0.805 | 0.421 |
| Wgt. Social Support | 0.2% | 1.3% | -0.018 | 0.035 | -0.528 | 0.598 |
| Wgt. Diet Norms | 0.4% | 3.7% | 0.040 | 0.035 | 1.140 | 0.255 |
| Wgt. Belittle | 0.6% | 5.1% | 0.003 | 0.025 | 0.120 | 0.905 |
| Total R² | 12.0% |  |  |  |  |  |
| Sample Size | 299 |  |  |  |  |  |
| Listwise Deletion | 0 |  |  |  |  |  |
| *Human Resources Policies* |  |  |  |  |  |  |
| Intercept |  |  | 1.568 | 0.064 | 24.384 | < 0.001 |
| Offer Exercise Program | 0.1% | 21.5% | -0.050 | 0.081 | -0.611 | 0.542 |
| Offer Weight Loss | 0.0% | 5.8% | -0.003 | 0.081 | -0.043 | 0.966 |
| Offer Maintain Health | 0.0% | 6.4% | 0.023 | 0.071 | 0.317 | 0.751 |
| Comm. Exercise Program | 0.1% | 23.2% | 0.038 | 0.054 | 0.709 | 0.479 |
| Comm. Weight Loss | 0.0% | 6.5% | 0.008 | 0.054 | 0.148 | 0.882 |
| Comm. Maintain Health | 0.2% | 36.6% | -0.038 | 0.044 | -0.866 | 0.387 |
| Total R² | 0.6% |  |  |  |  |  |
| Sample Size | 249 |  |  |  |  |  |
| Listwise Deletion | 50 |  |  |  |  |  |
| *Social Status* |  |  |  |  |  |  |
| Intercept |  |  | 1.373 | 0.125 | 10.950 | < 0.001 |
| Education Breakdown | 0.2% | 3.0% | -0.015 | 0.014 | -1.091 | 0.276 |
| Weight Comparison | 1.3% | 19.0% | -0.039 | 0.014 | -2.803 | 0.005 |
| Gender Breakdown | 0.0% | 0.4% | -0.003 | 0.007 | -0.498 | 0.619 |
| Manager | 1.7% | 24.5% | 0.111 | 0.034 | 3.239 | 0.001 |
| Visible Disability | 0.8% | 11.0% | 0.027 | 0.019 | 1.389 | 0.165 |
| Ethnic Name | 0.7% | 10.2% | 0.028 | 0.017 | 1.583 | 0.114 |
| Body-Mass Index | 0.4% | 5.3% | 0.003 | 0.002 | 1.363 | 0.173 |
| Accent | 0.7% | 9.6% | 0.023 | 0.017 | 1.302 | 0.193 |
| Gender | 1.0% | 13.9% | 0.084 | 0.034 | 2.470 | 0.014 |
| Race | 0.2% | 2.9% | 0.040 | 0.039 | 1.029 | 0.304 |
| Total R² | 7.1% |  |  |  |  |  |
| Sample Size | 591 |  |  |  |  |  |
| Listwise Deletion | 167 |  |  |  |  |  |
| *Identity Management Strategies* |  |  |  |  |  |  |
| Intercept |  |  | 1.566 | 0.060 | 26.232 | < 0.001 |
| Redefinition Reassociate | 0.2% | 24.1% | 0.020 | 0.017 | 1.160 | 0.247 |
| Redefinition Regenerate | 0.1% | 7.7% | 0.010 | 0.021 | 0.468 | 0.640 |
| Switch Deemphasize | 0.1% | 8.0% | 0.019 | 0.024 | 0.784 | 0.433 |
| Switch Emphasize | 0.5% | 60.2% | -0.043 | 0.020 | -2.121 | 0.034 |
| Total R² | 0.8% |  |  |  |  |  |
| Sample Size | 756 |  |  |  |  |  |
| Listwise Deletion | 2 |  |  |  |  |  |
| *Objectification by the Self* |  |  |  |  |  |  |
| Intercept |  |  | 1.636 | 0.200 | 8.189 | < 0.001 |
| Anxiety | 2.0% | 17.7% | 0.037 | 0.018 | 2.075 | 0.038 |
| Guilt | 2.2% | 19.1% | 0.043 | 0.018 | 2.435 | 0.015 |
| Shame | 1.2% | 10.8% | 0.030 | 0.023 | 1.303 | 0.193 |
| Depression | 1.1% | 10.0% | -0.024 | 0.024 | -1.020 | 0.308 |
| Core Self-Evaluations | 2.2% | 18.8% | -0.085 | 0.034 | -2.477 | 0.013 |
| Weight-Bias Internalization | 0.5% | 4.8% | -0.025 | 0.028 | -0.879 | 0.380 |
| Objectified Body Consc. | 1.2% | 10.4% | 0.035 | 0.016 | 2.203 | 0.028 |
| Intuitive Eating Trust | 0.1% | 0.8% | -0.003 | 0.014 | -0.180 | 0.857 |
| Body Dissatisfaction | 0.9% | 7.5% | -0.027 | 0.010 | -2.570 | 0.010 |
| Total R² | 11.5% |  |  |  |  |  |
| Sample Size | 734 |  |  |  |  |  |
| Listwise Deletion | 24 |  |  |  |  |  |
| *Objectification by Others* |  |  |  |  |  |  |
| Intercept |  |  | 1.183 | 0.106 | 11.199 | < 0.001 |
| Anti-fat Dislike | 2.1% | 26.4% | 0.037 | 0.038 | 0.982 | 0.327 |
| Anti-fat Fear | 2.5% | 31.1% | 0.069 | 0.045 | 1.511 | 0.132 |
| Anti-fat Willpower | 0.7% | 9.1% | -0.034 | 0.032 | -1.035 | 0.302 |
| Thin Ideal | 0.6% | 8.0% | 0.014 | 0.027 | 0.535 | 0.593 |
| Negative Body Talk Other | 1.9% | 23.8% | 0.047 | 0.037 | 1.265 | 0.207 |
| Negative Body Talk Self | 0.1% | 1.6% | -0.012 | 0.032 | -0.368 | 0.713 |
| Total R² | 8.0% |  |  |  |  |  |
| Sample Size | 299 |  |  |  |  |  |
| Listwise Deletion | 0 |  |  |  |  |  |
| *Job Context* |  |  |  |  |  |  |
| Intercept |  |  | 1.682 | 0.236 | 7.121 | < 0.001 |
| Job Task | 0.6% | 56.6% | -0.169 | 0.067 | -2.525 | 0.012 |
| Job Social | 0.5% | 42.3% | 0.114 | 0.050 | 2.261 | 0.024 |
| Job Physical | 0.0% | 1.0% | 0.019 | 0.040 | 0.471 | 0.638 |
| Total R² | 1.1% |  |  |  |  |  |
| Sample Size | 755 |  |  |  |  |  |
| Listwise Deletion | 3 |  |  |  |  |  |

Same notes as S3 Table.
