# Supplementary material for: An exploratory study of the correlates of weight-based mistreatment at work
Source: PLoS One. 2026 Aug 26;21(8):e0342144. doi: 10.1371/journal.pone.0342144 (PMC13514019; doi:10.1371/journal.pone.0342144)
Supplement: Supplementary file 10 [file pone.0342144.s010.docx]

**S10 Table. Ordinary Least-Squares Regression and Dominance Analysis for Micro-Invalidation WBM.**

| **Correlates** | **GD** | **RGD** | **B** | **SE** | **t** | **p** |
| --- | --- | --- | --- | --- | --- | --- |
| *Stereotypes* |  |  |  |  |  |  |
| Intercept |  |  | 1.855 | 0.155 | 11.939 | < 0.001 |
| Yourself Competent | 1.9% | 28.9% | -0.045 | 0.026 | -1.709 | 0.089 |
| Others Competent | 1.4% | 22.4% | -0.035 | 0.032 | -1.090 | 0.276 |
| Coworker Competent | 2.3% | 36.3% | -0.065 | 0.033 | -1.965 | 0.050 |
| Yourself Warm | 0.2% | 2.4% | 0.017 | 0.033 | 0.508 | 0.612 |
| Others Warm | 0.3% | 4.5% | 0.032 | 0.037 | 0.878 | 0.381 |
| Coworker Warm | 0.3% | 5.4% | < 0.001 | 0.036 | -0.008 | 0.993 |
| Total R^2^ | 6.5% |  |  |  |  |  |
| Sample Size | 293 |  |  |  |  |  |
| Listwise Deletion | 6 |  |  |  |  |  |
| *Interpersonal Climate* |  |  |  |  |  |  |
| Intercept |  |  | 1.477 | 0.151 | 9.784 | < 0.001 |
| Climate Incl. Equitable | 0.9% | 7.0% | -0.011 | 0.034 | -0.324 | 0.746 |
| Climate Incl. Integration | 1.4% | 11.1% | -0.038 | 0.042 | -0.910 | 0.364 |
| Climate Incl. DM | 1.2% | 9.0% | -0.012 | 0.035 | -0.352 | 0.725 |
| Civility Climate | 1.0% | 7.7% | 0.007 | 0.033 | 0.211 | 0.833 |
| Leader-Member Exchange | 1.4% | 11.1% | -0.028 | 0.031 | -0.886 | 0.376 |
| Perceived Org. Support | 1.1% | 8.5% | 0.001 | 0.033 | 0.022 | 0.982 |
| Interpersonal Conflict | 5.9% | 45.6% | 0.128 | 0.032 | 3.940 | < 0.001 |
| Total R² | 12.9% |  |  |  |  |  |
| Sample Size | 299 |  |  |  |  |  |
| Listwise Deletion | 0 |  |  |  |  |  |
| *Weight Control Climate* |  |  |  |  |  |  |
| Intercept |  |  | 1.023 | 0.109 | 9.388 | < 0.001 |
| Tolerate Neg. Wgt. Talk | 6.8% | 62.2% | 0.092 | 0.024 | 3.873 | < 0.001 |
| Pressure for Thinness | 1.8% | 16.9% | 0.034 | 0.029 | 1.195 | 0.233 |
| Climate for Exercise | 0.1% | 1.0% | -0.023 | 0.043 | -0.523 | 0.601 |
| Wgt. Org. Support | 0.1% | 0.9% | 0.023 | 0.035 | 0.664 | 0.507 |
| Wgt. Social Support | 0.1% | 1.1% | -0.027 | 0.035 | -0.765 | 0.445 |
| Wgt. Diet Norms | 0.2% | 2.2% | 0.034 | 0.036 | 0.952 | 0.342 |
| Wgt. Belittle | 1.7% | 15.7% | 0.034 | 0.025 | 1.354 | 0.177 |
| Total R² | 10.9% |  |  |  |  |  |
| Sample Size | 299 |  |  |  |  |  |
| Listwise Deletion | 0 |  |  |  |  |  |
| *Human Resources Policies* |  |  |  |  |  |  |
| Intercept |  |  | 1.555 | 0.065 | 23.992 | < 0.001 |
| Offer Exercise Program | 0.2% | 11.0% | -0.054 | 0.082 | -0.661 | 0.509 |
| Offer Weight Loss | 0.4% | 19.7% | 0.100 | 0.081 | 1.234 | 0.219 |
| Offer Maintain Health | 0.5% | 25.0% | -0.057 | 0.072 | -0.797 | 0.426 |
| Comm. Exercise Program | 0.1% | 7.3% | 0.031 | 0.055 | 0.569 | 0.570 |
| Comm. Weight Loss | 0.1% | 5.3% | 0.014 | 0.054 | 0.256 | 0.798 |
| Comm. Maintain Health | 0.6% | 31.7% | -0.054 | 0.045 | -1.223 | 0.222 |
| Total R² | 2.0% |  |  |  |  |  |
| Sample Size | 249 |  |  |  |  |  |
| Listwise Deletion | 50 |  |  |  |  |  |
| *Social Status* |  |  |  |  |  |  |
| Intercept |  |  | 1.226 | 0.127 | 9.645 | < 0.001 |
| Education Breakdown | 0.8% | 7.3% | -0.029 | 0.014 | -2.108 | 0.035 |
| Weight Comparison | 1.3% | 11.1% | -0.038 | 0.014 | -2.728 | 0.007 |
| Gender Breakdown | 0.1% | 0.6% | 0.003 | 0.007 | 0.462 | 0.644 |
| Manager | 1.0% | 8.9% | 0.086 | 0.035 | 2.485 | 0.013 |
| Visible Disability | 2.3% | 20.7% | 0.057 | 0.020 | 2.901 | 0.004 |
| Ethnic Name | 1.4% | 12.7% | 0.039 | 0.018 | 2.207 | 0.028 |
| Body-Mass Index | 0.7% | 6.3% | 0.004 | 0.002 | 1.977 | 0.048 |
| Accent | 1.4% | 12.3% | 0.032 | 0.018 | 1.800 | 0.072 |
| Gender | 1.9% | 16.6% | 0.114 | 0.034 | 3.298 | 0.001 |
| Race | 0.4% | 3.4% | 0.062 | 0.040 | 1.560 | 0.119 |
| Total R² | 11.3% |  |  |  |  |  |
| Sample Size | 592 |  |  |  |  |  |
| Listwise Deletion | 166 |  |  |  |  |  |
| *Identity Management Strategies* |  |  |  |  |  |  |
| Intercept |  |  | 1.525 | 0.061 | 24.858 | < 0.001 |
| Redefinition Reassociate | 0.5% | 26.4% | 0.029 | 0.017 | 1.662 | 0.097 |
| Redefinition Regenerate | 0.5% | 24.1% | 0.038 | 0.022 | 1.725 | 0.085 |
| Switch Deemphasize | 0.1% | 5.2% | 0.016 | 0.025 | 0.642 | 0.521 |
| Switch Emphasize | 0.9% | 44.3% | -0.063 | 0.021 | -3.011 | 0.003 |
| Total R² | 2.1% |  |  |  |  |  |
| Sample Size | 757 |  |  |  |  |  |
| Listwise Deletion | 1 |  |  |  |  |  |
| *Objectification by the Self* |  |  |  |  |  |  |
| Intercept |  |  | 1.334 | 0.204 | 6.548 | < 0.001 |
| Anxiety | 2.5% | 17.6% | 0.042 | 0.018 | 2.303 | 0.022 |
| Guilt | 2.9% | 20.1% | 0.050 | 0.018 | 2.797 | 0.005 |
| Shame | 1.7% | 12.0% | 0.042 | 0.024 | 1.777 | 0.076 |
| Depression | 1.5% | 10.3% | -0.019 | 0.024 | -0.769 | 0.442 |
| Core Self-Evaluations | 2.0% | 14.2% | -0.065 | 0.035 | -1.876 | 0.061 |
| Weight-Bias Internalization | 0.7% | 4.9% | -0.008 | 0.029 | -0.277 | 0.782 |
| Objectified Body Consc. | 1.7% | 12.2% | 0.043 | 0.016 | 2.628 | 0.009 |
| Intuitive Eating Trust | 0.3% | 2.3% | 0.018 | 0.014 | 1.231 | 0.219 |
| Body Dissatisfaction | 0.9% | 6.3% | -0.028 | 0.011 | -2.588 | 0.010 |
| Total R² | 14.2% |  |  |  |  |  |
| Sample Size | 735 |  |  |  |  |  |
| Listwise Deletion | 23 |  |  |  |  |  |
| *Objectification by Others* |  |  |  |  |  |  |
| Intercept |  |  | 1.071 | 0.107 | 10.016 | < 0.001 |
| Anti-fat Dislike | 2.6% | 29.5% | 0.055 | 0.038 | 1.452 | 0.147 |
| Anti-fat Fear | 2.2% | 25.5% | 0.055 | 0.046 | 1.191 | 0.234 |
| Anti-fat Willpower | 0.7% | 8.5% | -0.037 | 0.033 | -1.129 | 0.260 |
| Thin Ideal | 0.3% | 3.8% | -0.012 | 0.027 | -0.448 | 0.655 |
| Negative Body Talk Other | 2.2% | 25.7% | 0.052 | 0.037 | 1.410 | 0.160 |
| Negative Body Talk Self | 0.6% | 7.0% | 0.032 | 0.033 | 0.992 | 0.322 |
| Total R² | 8.7% |  |  |  |  |  |
| Sample Size | 299 |  |  |  |  |  |
| Listwise Deletion | 0 |  |  |  |  |  |
| *Job Context* |  |  |  |  |  |  |
| Intercept |  |  | 1.861 | 0.245 | 7.608 | < 0.001 |
| Job Task | 0.8% | 86.5% | -0.176 | 0.069 | -2.545 | 0.011 |
| Job Social | 0.1% | 11.7% | 0.063 | 0.052 | 1.205 | 0.229 |
| Job Physical | 0.0% | 1.9% | 0.022 | 0.042 | 0.530 | 0.596 |
| Total R² | 0.9% |  |  |  |  |  |
| Sample Size | 756 |  |  |  |  |  |
| Listwise Deletion | 2 |  |  |  |  |  |

Same notes as S3 Table.
