# Supplementary material for: An exploratory study of the correlates of weight-based mistreatment at work
Source: PLoS One. 2026 Aug 26;21(8):e0342144. doi: 10.1371/journal.pone.0342144 (PMC13514019; doi:10.1371/journal.pone.0342144)
Supplement: Supplementary file 11 [file pone.0342144.s011.docx]

**S11 Table. Ordinary Least-Squares Regression and Dominance Analysis for Overt WBM.**

| **Correlates** | **GD** | **RGD** | **B** | **SE** | **t** | **p** |
| --- | --- | --- | --- | --- | --- | --- |
| *Stereotypes* |  |  |  |  |  |  |
| Intercept |  |  | 1.723 | 0.097 | 17.769 | < 0.001 |
| Yourself Competent | 1.4% | 15.3% | -0.023 | 0.017 | -1.415 | 0.158 |
| Others Competent | 1.5% | 16.3% | -0.016 | 0.020 | -0.807 | 0.420 |
| Coworker Competent | 3.1% | 32.9% | -0.035 | 0.021 | -1.701 | 0.090 |
| Yourself Warm | 0.2% | 2.4% | 0.006 | 0.020 | 0.298 | 0.766 |
| Others Warm | 0.3% | 3.1% | -0.001 | 0.023 | -0.030 | 0.976 |
| Coworker Warm | 2.8% | 30.0% | -0.043 | 0.023 | -1.913 | 0.057 |
| Total R^2^ | 9.4% |  |  |  |  |  |
| Sample Size | 292 |  |  |  |  |  |
| Listwise Deletion | 7 |  |  |  |  |  |
| *Interpersonal Climate* |  |  |  |  |  |  |
| Intercept |  |  | 1.244 | 0.095 | 13.137 | < 0.001 |
| Climate Incl. Equitable | 1.2% | 8.5% | -0.017 | 0.021 | -0.799 | 0.425 |
| Climate Incl. Integration | 1.8% | 12.3% | -0.035 | 0.026 | -1.327 | 0.185 |
| Climate Incl. DM | 0.9% | 6.0% | 0.010 | 0.022 | 0.465 | 0.642 |
| Civility Climate | 1.2% | 8.0% | 0.001 | 0.021 | 0.049 | 0.961 |
| Leader-Member Exchange | 1.2% | 8.6% | -0.013 | 0.020 | -0.678 | 0.498 |
| Perceived Org. Support | 1.1% | 7.5% | 0.002 | 0.021 | 0.115 | 0.909 |
| Interpersonal Conflict | 7.1% | 49.2% | 0.090 | 0.020 | 4.416 | < 0.001 |
| Total R² | 14.5% |  |  |  |  |  |
| Sample Size | 298 |  |  |  |  |  |
| Listwise Deletion | 1 |  |  |  |  |  |
| *Weight Control Climate* |  |  |  |  |  |  |
| Intercept |  |  | 0.979 | 0.070 | 14.075 | < 0.001 |
| Tolerate Neg. Wgt. Talk | 5.5% | 57.4% | 0.052 | 0.015 | 3.451 | 0.001 |
| Pressure for Thinness | 1.1% | 11.8% | 0.011 | 0.018 | 0.597 | 0.551 |
| Climate for Exercise | 0.1% | 1.5% | 0.017 | 0.028 | 0.615 | 0.539 |
| Wgt. Org. Support | 0.1% | 1.5% | -0.014 | 0.022 | -0.652 | 0.515 |
| Wgt. Social Support | 0.2% | 2.2% | -0.026 | 0.023 | -1.169 | 0.243 |
| Wgt. Diet Norms | 0.7% | 7.4% | 0.040 | 0.023 | 1.732 | 0.084 |
| Wgt. Belittle | 1.7% | 18.2% | 0.026 | 0.016 | 1.601 | 0.110 |
| Total R² | 9.5% |  |  |  |  |  |
| Sample Size | 298 |  |  |  |  |  |
| Listwise Deletion | 1 |  |  |  |  |  |
| *Human Resources Policies* |  |  |  |  |  |  |
| Intercept |  |  | 1.275 | 0.041 | 31.138 | < 0.001 |
| Offer Exercise Program | 0.0% | 3.7% | -0.010 | 0.052 | -0.201 | 0.841 |
| Offer Weight Loss | 0.2% | 22.5% | 0.046 | 0.051 | 0.891 | 0.374 |
| Offer Maintain Health | 0.2% | 20.6% | -0.030 | 0.045 | -0.657 | 0.512 |
| Comm. Exercise Program | 0.1% | 5.4% | 0.008 | 0.035 | 0.236 | 0.813 |
| Comm. Weight Loss | 0.1% | 10.6% | 0.018 | 0.034 | 0.537 | 0.592 |
| Comm. Maintain Health | 0.4% | 37.2% | -0.030 | 0.028 | -1.062 | 0.289 |
| Total R² | 1.1% |  |  |  |  |  |
| Sample Size | 249 |  |  |  |  |  |
| Listwise Deletion | 50 |  |  |  |  |  |
| *Social Status* |  |  |  |  |  |  |
| Intercept |  |  | 1.027 | 0.089 | 11.504 | < 0.001 |
| Education Breakdown | 1.0% | 4.4% | -0.023 | 0.010 | -2.361 | 0.019 |
| Weight Comparison | 0.2% | 1.0% | -0.015 | 0.010 | -1.498 | 0.135 |
| Gender Breakdown | 0.2% | 1.0% | 0.008 | 0.005 | 1.581 | 0.115 |
| Manager | 1.7% | 7.7% | 0.072 | 0.024 | 2.971 | 0.003 |
| Visible Disability | 7.8% | 35.8% | 0.079 | 0.014 | 5.787 | < 0.001 |
| Ethnic Name | 5.1% | 23.4% | 0.046 | 0.012 | 3.684 | < 0.001 |
| Body-Mass Index | 0.2% | 1.0% | 0.002 | 0.001 | 1.455 | 0.146 |
| Accent | 5.4% | 24.8% | 0.047 | 0.012 | 3.808 | < 0.001 |
| Gender | 0.1% | 0.3% | 0.013 | 0.024 | 0.540 | 0.589 |
| Race | 0.1% | 0.6% | 0.023 | 0.028 | 0.836 | 0.403 |
| Total R² | 21.8% |  |  |  |  |  |
| Sample Size | 592 |  |  |  |  |  |
| Listwise Deletion | 166 |  |  |  |  |  |
| *Identity Management Strategies* |  |  |  |  |  |  |
| Intercept |  |  | 1.278 | 0.046 | 27.835 | < 0.001 |
| Redefinition Reassociate | 1.3% | 35.0% | 0.031 | 0.013 | 2.395 | 0.017 |
| Redefinition Regenerate | 1.2% | 34.3% | 0.042 | 0.016 | 2.563 | 0.011 |
| Switch Deemphasize | 0.1% | 4.0% | 0.014 | 0.018 | 0.754 | 0.451 |
| Switch Emphasize | 1.0% | 26.7% | -0.052 | 0.016 | -3.322 | 0.001 |
| Total R² | 3.6% |  |  |  |  |  |
| Sample Size | 757 |  |  |  |  |  |
| Listwise Deletion | 1 |  |  |  |  |  |
| *Objectification by the Self* |  |  |  |  |  |  |
| Intercept |  |  | 1.293 | 0.153 | 8.436 | < 0.001 |
| Anxiety | 2.6% | 17.2% | 0.032 | 0.014 | 2.309 | 0.021 |
| Guilt | 3.1% | 20.4% | 0.044 | 0.014 | 3.249 | 0.001 |
| Shame | 0.4% | 2.9% | 0.004 | 0.018 | 0.200 | 0.841 |
| Depression | 1.8% | 11.8% | 0.026 | 0.018 | 1.445 | 0.149 |
| Core Self-Evaluations | 0.9% | 6.0% | -0.022 | 0.026 | -0.851 | 0.395 |
| Weight-Bias Internalization | 0.7% | 4.4% | -0.045 | 0.022 | -2.092 | 0.037 |
| Objectified Body Consc. | 1.2% | 7.9% | 0.023 | 0.012 | 1.898 | 0.058 |
| Intuitive Eating Trust | 0.7% | 4.4% | 0.015 | 0.011 | 1.365 | 0.173 |
| Body Dissatisfaction | 3.8% | 25.0% | -0.042 | 0.008 | -5.176 | < 0.001 |
| Total R² | 15.1% |  |  |  |  |  |
| Sample Size | 735 |  |  |  |  |  |
| Listwise Deletion | 23 |  |  |  |  |  |
| *Objectification by Others* |  |  |  |  |  |  |
| Intercept |  |  | 1.011 | 0.067 | 15.065 | < 0.001 |
| Anti-fat Dislike | 2.9% | 29.2% | 0.043 | 0.024 | 1.809 | 0.072 |
| Anti-fat Fear | 1.9% | 19.1% | 0.028 | 0.029 | 0.969 | 0.333 |
| Anti-fat Willpower | 1.1% | 10.5% | -0.045 | 0.021 | -2.187 | 0.030 |
| Thin Ideal | 0.5% | 4.6% | 0.001 | 0.017 | 0.054 | 0.957 |
| Negative Body Talk Other | 2.9% | 29.0% | 0.046 | 0.023 | 1.965 | 0.050 |
| Negative Body Talk Self | 0.7% | 7.4% | 0.023 | 0.020 | 1.118 | 0.265 |
| Total R² | 10.0% |  |  |  |  |  |
| Sample Size | 298 |  |  |  |  |  |
| Listwise Deletion | 1 |  |  |  |  |  |
| *Job Context* |  |  |  |  |  |  |
| Intercept |  |  | 1.510 | 0.185 | 8.178 | < 0.001 |
| Job Task | 0.3% | 48.4% | -0.077 | 0.052 | -1.481 | 0.139 |
| Job Social | 0.1% | 19.2% | 0.043 | 0.039 | 1.092 | 0.275 |
| Job Physical | 0.2% | 32.4% | -0.031 | 0.031 | -0.982 | 0.326 |
| Total R² | 0.6% |  |  |  |  |  |
| Sample Size | 756 |  |  |  |  |  |
| Listwise Deletion | 2 |  |  |  |  |  |

Same notes as S3 Table.
