# Supplementary material for: An exploratory study of the correlates of weight-based mistreatment at work
Source: PLoS One. 2026 Aug 26;21(8):e0342144. doi: 10.1371/journal.pone.0342144 (PMC13514019; doi:10.1371/journal.pone.0342144)
Supplement: Supplementary file 12 [file pone.0342144.s012.docx]

**S12 Table. Ordinary Least-Squares Regression and Dominance Analysis for WBM by Boss.**

| **Correlates** | **GD** | **RGD** | **B** | **SE** | **t** | **p** |
| --- | --- | --- | --- | --- | --- | --- |
| *Stereotypes* |  |  |  |  |  |  |
| Intercept |  |  | 1.823 | 0.153 | 11.909 | < 0.001 |
| Yourself Competent | 1.8% | 28.2% | -0.045 | 0.026 | -1.722 | 0.086 |
| Others Competent | 1.0% | 15.1% | -0.019 | 0.031 | -0.589 | 0.556 |
| Coworker Competent | 2.6% | 41.5% | -0.068 | 0.033 | -2.081 | 0.038 |
| Yourself Warm | 0.1% | 1.6% | < 0.001 | 0.032 | 0.006 | 0.996 |
| Others Warm | 0.2% | 3.4% | 0.030 | 0.036 | 0.834 | 0.405 |
| Coworker Warm | 0.6% | 10.2% | -0.013 | 0.036 | -0.369 | 0.713 |
| Total R^2^ | 6.4% |  |  |  |  |  |
| Sample Size | 293 |  |  |  |  |  |
| Listwise Deletion | 6 |  |  |  |  |  |
| *Interpersonal Climate* |  |  |  |  |  |  |
| Intercept |  |  | 1.579 | 0.140 | 11.300 | < 0.001 |
| Climate Incl. Equitable | 3.1% | 12.0% | -0.056 | 0.031 | -1.793 | 0.074 |
| Climate Incl. Integration | 2.8% | 10.7% | -0.052 | 0.039 | -1.333 | 0.184 |
| Climate Incl. DM | 1.7% | 6.6% | 0.035 | 0.033 | 1.055 | 0.292 |
| Civility Climate | 1.9% | 7.4% | 0.017 | 0.031 | 0.570 | 0.569 |
| Leader-Member Exchange | 5.8% | 22.5% | -0.094 | 0.029 | -3.237 | 0.001 |
| Perceived Org. Support | 2.4% | 9.3% | 0.001 | 0.031 | 0.021 | 0.984 |
| Interpersonal Conflict | 8.2% | 31.5% | 0.143 | 0.030 | 4.777 | < 0.001 |
| Total R² | 26.0% |  |  |  |  |  |
| Sample Size | 299 |  |  |  |  |  |
| Listwise Deletion | 0 |  |  |  |  |  |
| *Weight Control Climate* |  |  |  |  |  |  |
| Intercept |  |  | 0.679 | 0.103 | 6.579 | < 0.001 |
| Tolerate Neg. Wgt. Talk | 11.1% | 53.3% | 0.118 | 0.022 | 5.261 | < 0.001 |
| Pressure for Thinness | 1.7% | 8.0% | 0.009 | 0.027 | 0.346 | 0.730 |
| Climate for Exercise | 0.2% | 0.9% | 0.020 | 0.041 | 0.487 | 0.626 |
| Wgt. Org. Support | 0.2% | 0.8% | -0.018 | 0.033 | -0.537 | 0.592 |
| Wgt. Social Support | 0.4% | 1.9% | -0.057 | 0.033 | -1.691 | 0.092 |
| Wgt. Diet Norms | 2.1% | 10.2% | 0.108 | 0.034 | 3.156 | 0.002 |
| Wgt. Belittle | 5.2% | 24.8% | 0.079 | 0.024 | 3.319 | 0.001 |
| Total R² | 20.8% |  |  |  |  |  |
| Sample Size | 299 |  |  |  |  |  |
| Listwise Deletion | 0 |  |  |  |  |  |
| *Human Resources Policies* |  |  |  |  |  |  |
| Intercept |  |  | 1.415 | 0.065 | 21.817 | < 0.001 |
| Offer Exercise Program | 0.2% | 13.0% | -0.060 | 0.082 | -0.734 | 0.464 |
| Offer Weight Loss | 0.2% | 12.3% | 0.070 | 0.081 | 0.860 | 0.391 |
| Offer Maintain Health | 0.5% | 28.2% | -0.062 | 0.072 | -0.865 | 0.388 |
| Comm. Exercise Program | 0.4% | 26.6% | 0.057 | 0.055 | 1.041 | 0.299 |
| Comm. Weight Loss | 0.1% | 8.4% | 0.012 | 0.054 | 0.227 | 0.821 |
| Comm. Maintain Health | 0.2% | 11.6% | -0.035 | 0.045 | -0.776 | 0.438 |
| Total R² | 1.7% |  |  |  |  |  |
| Sample Size | 249 |  |  |  |  |  |
| Listwise Deletion | 50 |  |  |  |  |  |
| *Social Status* |  |  |  |  |  |  |
| Intercept |  |  | 1.280 | 0.131 | 9.756 | < 0.001 |
| Education Breakdown | 0.8% | 7.4% | -0.029 | 0.014 | -2.073 | 0.039 |
| Weight Comparison | 1.3% | 12.6% | -0.043 | 0.014 | -2.995 | 0.003 |
| Gender Breakdown | 0.0% | 0.2% | 0.003 | 0.007 | 0.403 | 0.687 |
| Manager | 0.9% | 9.2% | 0.077 | 0.036 | 2.161 | 0.031 |
| Visible Disability | 3.7% | 36.7% | 0.080 | 0.020 | 3.977 | < 0.001 |
| Ethnic Name | 1.4% | 13.5% | 0.031 | 0.018 | 1.672 | 0.095 |
| Body-Mass Index | 0.1% | 1.4% | 0.002 | 0.002 | 0.821 | 0.412 |
| Accent | 1.7% | 16.6% | 0.036 | 0.018 | 1.977 | 0.049 |
| Gender | 0.1% | 0.5% | 0.017 | 0.036 | 0.475 | 0.635 |
| Race | 0.2% | 1.8% | 0.043 | 0.041 | 1.055 | 0.292 |
| Total R² | 10.2% |  |  |  |  |  |
| Sample Size | 592 |  |  |  |  |  |
| Listwise Deletion | 166 |  |  |  |  |  |
| *Identity Management Strategies* |  |  |  |  |  |  |
| Intercept |  |  | 1.481 | 0.063 | 23.503 | < 0.001 |
| Redefinition Reassociate | 0.0% | 3.0% | 0.006 | 0.018 | 0.334 | 0.738 |
| Redefinition Regenerate | 0.2% | 13.8% | 0.032 | 0.022 | 1.425 | 0.155 |
| Switch Deemphasize | 0.2% | 10.3% | 0.033 | 0.025 | 1.298 | 0.195 |
| Switch Emphasize | 1.2% | 72.9% | -0.072 | 0.022 | -3.339 | 0.001 |
| Total R² | 1.6% |  |  |  |  |  |
| Sample Size | 757 |  |  |  |  |  |
| Listwise Deletion | 1 |  |  |  |  |  |
| *Objectification by the Self* |  |  |  |  |  |  |
| Intercept |  |  | 1.438 | 0.205 | 7.016 | < 0.001 |
| Anxiety | 4.0% | 23.5% | 0.060 | 0.019 | 3.226 | 0.001 |
| Guilt | 2.9% | 17.1% | 0.046 | 0.018 | 2.527 | 0.012 |
| Shame | 1.2% | 6.8% | 0.018 | 0.024 | 0.753 | 0.452 |
| Depression | 2.3% | 13.6% | 0.011 | 0.024 | 0.431 | 0.666 |
| Core Self-Evaluations | 2.5% | 14.3% | -0.083 | 0.035 | -2.366 | 0.018 |
| Weight-Bias Internalization | 0.8% | 4.5% | -0.062 | 0.029 | -2.157 | 0.031 |
| Objectified Body Consc. | 2.5% | 14.4% | 0.055 | 0.016 | 3.352 | 0.001 |
| Intuitive Eating Trust | 0.2% | 1.2% | 0.008 | 0.014 | 0.523 | 0.601 |
| Body Dissatisfaction | 0.8% | 4.6% | -0.025 | 0.011 | -2.317 | 0.021 |
| Total R² | 17.2% |  |  |  |  |  |
| Sample Size | 735 |  |  |  |  |  |
| Listwise Deletion | 23 |  |  |  |  |  |
| *Objectification by Others* |  |  |  |  |  |  |
| Intercept |  |  | 1.038 | 0.104 | 9.935 | < 0.001 |
| Anti-fat Dislike | 5.7% | 42.0% | 0.111 | 0.037 | 2.995 | 0.003 |
| Anti-fat Fear | 2.5% | 18.7% | 0.026 | 0.045 | 0.588 | 0.557 |
| Anti-fat Willpower | 1.0% | 7.5% | -0.050 | 0.032 | -1.577 | 0.116 |
| Thin Ideal | 1.3% | 9.4% | 0.036 | 0.027 | 1.349 | 0.178 |
| Negative Body Talk Other | 2.7% | 19.9% | 0.053 | 0.036 | 1.453 | 0.147 |
| Negative Body Talk Self | 0.3% | 2.5% | -0.039 | 0.032 | -1.235 | 0.218 |
| Total R² | 13.6% |  |  |  |  |  |
| Sample Size | 299 |  |  |  |  |  |
| Listwise Deletion | 0 |  |  |  |  |  |
| *Job Context* |  |  |  |  |  |  |
| Intercept |  |  | 1.722 | 0.251 | 6.869 | < 0.001 |
| Job Task | 0.5% | 86.1% | -0.143 | 0.071 | -2.020 | 0.044 |
| Job Social | 0.1% | 12.8% | 0.054 | 0.054 | 0.997 | 0.319 |
| Job Physical | 0.0% | 1.1% | 0.007 | 0.043 | 0.157 | 0.876 |
| Total R² | 0.6% |  |  |  |  |  |
| Sample Size | 756 |  |  |  |  |  |
| Listwise Deletion | 2 |  |  |  |  |  |

Same notes as S3 Table.
