# Supplementary material for: An exploratory study of the correlates of weight-based mistreatment at work
Source: PLoS One. 2026 Aug 26;21(8):e0342144. doi: 10.1371/journal.pone.0342144 (PMC13514019; doi:10.1371/journal.pone.0342144)
Supplement: Supplementary file 13 [file pone.0342144.s013.docx]

**S13 Table. Ordinary Least-Squares Regression and Dominance Analysis for WBM by Coworker.**

| **Correlates** | **GD** | **RGD** | **B** | **SE** | **t** | **p** |
| --- | --- | --- | --- | --- | --- | --- |
| *Stereotypes* |  |  |  |  |  |  |
| Intercept |  |  | 1.956 | 0.133 | 14.658 | < 0.001 |
| Yourself Competent | 1.4% | 22.8% | -0.036 | 0.023 | -1.594 | 0.112 |
| Others Competent | 0.9% | 14.7% | -0.014 | 0.027 | -0.520 | 0.603 |
| Coworker Competent | 2.9% | 48.0% | -0.065 | 0.028 | -2.294 | 0.023 |
| Yourself Warm | 0.2% | 3.6% | 0.024 | 0.028 | 0.847 | 0.398 |
| Others Warm | 0.1% | 2.1% | 0.011 | 0.032 | 0.336 | 0.737 |
| Coworker Warm | 0.5% | 8.7% | -0.005 | 0.031 | -0.167 | 0.868 |
| Total R^2^ | 6.0% |  |  |  |  |  |
| Sample Size | 293 |  |  |  |  |  |
| Listwise Deletion | 6 |  |  |  |  |  |
| *Interpersonal Climate* |  |  |  |  |  |  |
| Intercept |  |  | 1.586 | 0.128 | 12.423 | < 0.001 |
| Climate Incl. Equitable | 0.5% | 2.9% | 0.033 | 0.029 | 1.156 | 0.249 |
| Climate Incl. Integration | 2.0% | 11.1% | -0.071 | 0.035 | -1.990 | 0.048 |
| Climate Incl. DM | 0.7% | 3.8% | 0.030 | 0.030 | 1.006 | 0.315 |
| Civility Climate | 3.8% | 21.3% | -0.055 | 0.028 | -1.963 | 0.051 |
| Leader-Member Exchange | 1.0% | 5.3% | 0.006 | 0.026 | 0.236 | 0.814 |
| Perceived Org. Support | 1.8% | 10.2% | -0.016 | 0.028 | -0.560 | 0.576 |
| Interpersonal Conflict | 8.2% | 45.4% | 0.125 | 0.027 | 4.550 | < 0.001 |
| Total R² | 18.0% |  |  |  |  |  |
| Sample Size | 299 |  |  |  |  |  |
| Listwise Deletion | 0 |  |  |  |  |  |
| *Weight Control Climate* |  |  |  |  |  |  |
| Intercept |  |  | 1.086 | 0.093 | 11.676 | < 0.001 |
| Tolerate Neg. Wgt. Talk | 4.4% | 30.1% | 0.047 | 0.020 | 2.295 | 0.022 |
| Pressure for Thinness | 4.3% | 29.3% | 0.064 | 0.024 | 2.624 | 0.009 |
| Climate for Exercise | 0.2% | 1.4% | 0.006 | 0.037 | 0.161 | 0.872 |
| Wgt. Org. Support | 0.1% | 0.6% | -0.007 | 0.030 | -0.233 | 0.816 |
| Wgt. Social Support | 0.2% | 1.4% | -0.030 | 0.030 | -0.988 | 0.324 |
| Wgt. Diet Norms | 0.5% | 3.1% | 0.044 | 0.031 | 1.419 | 0.157 |
| Wgt. Belittle | 5.0% | 34.2% | 0.072 | 0.022 | 3.319 | 0.001 |
| Total R² | 14.7% |  |  |  |  |  |
| Sample Size | 299 |  |  |  |  |  |
| Listwise Deletion | 0 |  |  |  |  |  |
| *Human Resources Policies* |  |  |  |  |  |  |
| Intercept |  |  | 1.651 | 0.056 | 29.306 | < 0.001 |
| Offer Exercise Program | 0.6% | 34.7% | -0.102 | 0.071 | -1.432 | 0.154 |
| Offer Weight Loss | 0.4% | 25.5% | 0.094 | 0.071 | 1.330 | 0.185 |
| Offer Maintain Health | 0.1% | 6.9% | 0.002 | 0.063 | 0.035 | 0.972 |
| Comm. Exercise Program | 0.1% | 4.6% | 0.004 | 0.048 | 0.089 | 0.929 |
| Comm. Weight Loss | 0.1% | 7.6% | 0.028 | 0.047 | 0.597 | 0.551 |
| Comm. Maintain Health | 0.4% | 20.7% | -0.040 | 0.039 | -1.034 | 0.302 |
| Total R² | 1.8% |  |  |  |  |  |
| Sample Size | 249 |  |  |  |  |  |
| Listwise Deletion | 50 |  |  |  |  |  |
| *Social Status* |  |  |  |  |  |  |
| Intercept |  |  | 1.470 | 0.117 | 12.582 | < 0.001 |
| Education Breakdown | 0.3% | 3.7% | -0.016 | 0.013 | -1.290 | 0.198 |
| Weight Comparison | 0.6% | 7.2% | -0.026 | 0.013 | -1.988 | 0.047 |
| Gender Breakdown | 0.0% | 0.5% | -0.002 | 0.006 | -0.383 | 0.702 |
| Manager | 1.2% | 14.4% | 0.080 | 0.032 | 2.511 | 0.012 |
| Visible Disability | 1.2% | 15.2% | 0.028 | 0.018 | 1.575 | 0.116 |
| Ethnic Name | 2.7% | 32.8% | 0.049 | 0.016 | 3.032 | 0.003 |
| Body-Mass Index | 0.2% | 2.3% | 0.002 | 0.002 | 1.219 | 0.223 |
| Accent | 1.8% | 22.4% | 0.032 | 0.016 | 2.003 | 0.046 |
| Gender | 0.1% | 0.7% | 0.020 | 0.032 | 0.643 | 0.520 |
| Race | 0.1% | 0.7% | 0.018 | 0.037 | 0.500 | 0.617 |
| Total R² | 8.1% |  |  |  |  |  |
| Sample Size | 592 |  |  |  |  |  |
| Listwise Deletion | 166 |  |  |  |  |  |
| *Identity Management Strategies* |  |  |  |  |  |  |
| Intercept |  |  | 1.610 | 0.054 | 29.636 | < 0.001 |
| Redefinition Reassociate | 0.3% | 24.0% | 0.023 | 0.015 | 1.489 | 0.137 |
| Redefinition Regenerate | 0.1% | 7.0% | 0.007 | 0.019 | 0.377 | 0.706 |
| Switch Deemphasize | 0.3% | 21.5% | 0.042 | 0.022 | 1.924 | 0.055 |
| Switch Emphasize | 0.6% | 47.5% | -0.052 | 0.019 | -2.784 | 0.006 |
| Total R² | 1.4% |  |  |  |  |  |
| Sample Size | 757 |  |  |  |  |  |
| Listwise Deletion | 1 |  |  |  |  |  |
| *Objectification by the Self* |  |  |  |  |  |  |
| Intercept |  |  | 1.527 | 0.180 | 8.488 | < 0.001 |
| Anxiety | 2.5% | 19.1% | 0.039 | 0.016 | 2.420 | 0.016 |
| Guilt | 3.6% | 27.4% | 0.060 | 0.016 | 3.743 | < 0.001 |
| Shame | 1.6% | 12.0% | 0.040 | 0.021 | 1.922 | 0.055 |
| Depression | 1.3% | 9.9% | -0.022 | 0.021 | -1.005 | 0.315 |
| Core Self-Evaluations | 1.6% | 12.5% | -0.047 | 0.031 | -1.515 | 0.130 |
| Weight-Bias Internalization | 0.6% | 4.3% | -0.030 | 0.025 | -1.175 | 0.241 |
| Objectified Body Consc. | 1.0% | 8.0% | 0.026 | 0.014 | 1.819 | 0.069 |
| Intuitive Eating Trust | 0.1% | 1.1% | 0.005 | 0.013 | 0.418 | 0.676 |
| Body Dissatisfaction | 0.7% | 5.7% | -0.021 | 0.009 | -2.264 | 0.024 |
| Total R² | 13.1% |  |  |  |  |  |
| Sample Size | 735 |  |  |  |  |  |
| Listwise Deletion | 23 |  |  |  |  |  |
| *Objectification by Others* |  |  |  |  |  |  |
| Intercept |  |  | 1.190 | 0.088 | 13.495 | < 0.001 |
| Anti-fat Dislike | 6.5% | 35.3% | 0.082 | 0.031 | 2.611 | 0.010 |
| Anti-fat Fear | 4.4% | 23.8% | 0.049 | 0.038 | 1.288 | 0.199 |
| Anti-fat Willpower | 1.7% | 9.5% | -0.019 | 0.027 | -0.711 | 0.478 |
| Thin Ideal | 0.5% | 3.0% | -0.013 | 0.022 | -0.597 | 0.551 |
| Negative Body Talk Other | 5.0% | 27.1% | 0.070 | 0.031 | 2.296 | 0.022 |
| Negative Body Talk Self | 0.2% | 1.2% | -0.018 | 0.027 | -0.668 | 0.504 |
| Total R² | 18.3% |  |  |  |  |  |
| Sample Size | 299 |  |  |  |  |  |
| Listwise Deletion | 0 |  |  |  |  |  |
| *Job Context* |  |  |  |  |  |  |
| Intercept |  |  | 1.747 | 0.217 | 8.056 | < 0.001 |
| Job Task | 0.2% | 50.3% | -0.082 | 0.061 | -1.342 | 0.180 |
| Job Social | 0.0% | 4.9% | 0.026 | 0.046 | 0.554 | 0.580 |
| Job Physical | 0.2% | 44.8% | 0.046 | 0.037 | 1.249 | 0.212 |
| Total R² | 0.4% |  |  |  |  |  |
| Sample Size | 756 |  |  |  |  |  |
| Listwise Deletion | 2 |  |  |  |  |  |

Same notes as S3 Table.
