# Supplementary material for: An exploratory study of the correlates of weight-based mistreatment at work
Source: PLoS One. 2026 Aug 26;21(8):e0342144. doi: 10.1371/journal.pone.0342144 (PMC13514019; doi:10.1371/journal.pone.0342144)
Supplement: Supplementary file 14 [file pone.0342144.s014.docx]

**S14 Table. Ordinary Least-Squares Regression and Dominance Analysis for WBM by Customer.**

| **Correlates** | **GD** | **RGD** | **B** | **SE** | **t** | **p** |
| --- | --- | --- | --- | --- | --- | --- |
| *Stereotypes* |  |  |  |  |  |  |
| Intercept |  |  | 1.858 | 0.147 | 12.668 | < 0.001 |
| Yourself Competent | 0.8% | 12.4% | -0.030 | 0.025 | -1.183 | 0.238 |
| Others Competent | 0.6% | 8.7% | -0.003 | 0.030 | -0.108 | 0.914 |
| Coworker Competent | 2.9% | 45.4% | -0.061 | 0.031 | -1.942 | 0.053 |
| Yourself Warm | 0.1% | 1.4% | 0.010 | 0.031 | 0.335 | 0.738 |
| Others Warm | 0.1% | 1.4% | 0.001 | 0.035 | 0.035 | 0.972 |
| Coworker Warm | 2.0% | 30.7% | -0.049 | 0.034 | -1.429 | 0.154 |
| Total R^2^ | 6.4% |  |  |  |  |  |
| Sample Size | 292 |  |  |  |  |  |
| Listwise Deletion | 7 |  |  |  |  |  |
| *Interpersonal Climate* |  |  |  |  |  |  |
| Intercept |  |  | 1.242 | 0.148 | 8.419 | < 0.001 |
| Climate Incl. Equitable | 0.4% | 5.3% | 0.003 | 0.033 | 0.105 | 0.917 |
| Climate Incl. Integration | 1.2% | 15.4% | -0.049 | 0.041 | -1.186 | 0.236 |
| Climate Incl. DM | 0.8% | 10.1% | -0.017 | 0.035 | -0.480 | 0.632 |
| Civility Climate | 0.4% | 5.7% | 0.009 | 0.032 | 0.293 | 0.770 |
| Leader-Member Exchange | 0.4% | 5.8% | -0.007 | 0.031 | -0.237 | 0.813 |
| Perceived Org. Support | 0.4% | 5.2% | 0.015 | 0.033 | 0.445 | 0.657 |
| Interpersonal Conflict | 4.0% | 52.3% | 0.105 | 0.032 | 3.281 | 0.001 |
| Total R² | 7.6% |  |  |  |  |  |
| Sample Size | 298 |  |  |  |  |  |
| Listwise Deletion | 1 |  |  |  |  |  |
| *Weight Control Climate* |  |  |  |  |  |  |
| Intercept |  |  | 1.018 | 0.106 | 9.629 | < 0.001 |
| Tolerate Neg. Wgt. Talk | 4.2% | 62.8% | 0.071 | 0.023 | 3.094 | 0.002 |
| Pressure for Thinness | 1.4% | 20.4% | 0.029 | 0.028 | 1.045 | 0.297 |
| Climate for Exercise | 0.2% | 3.3% | 0.029 | 0.042 | 0.700 | 0.484 |
| Wgt. Org. Support | 0.1% | 1.3% | 0.015 | 0.034 | 0.447 | 0.655 |
| Wgt. Social Support | 0.3% | 4.3% | -0.044 | 0.034 | -1.283 | 0.201 |
| Wgt. Diet Norms | 0.0% | 0.4% | -0.001 | 0.035 | -0.025 | 0.980 |
| Wgt. Belittle | 0.5% | 7.5% | 0.010 | 0.025 | 0.424 | 0.672 |
| Total R² | 6.6% |  |  |  |  |  |
| Sample Size | 298 |  |  |  |  |  |
| Listwise Deletion | 1 |  |  |  |  |  |
| *Human Resources Policies* |  |  |  |  |  |  |
| Intercept |  |  | 1.363 | 0.062 | 21.908 | < 0.001 |
| Offer Exercise Program | 0.1% | 9.2% | -0.046 | 0.078 | -0.590 | 0.556 |
| Offer Weight Loss | 0.1% | 9.7% | 0.050 | 0.078 | 0.643 | 0.521 |
| Offer Maintain Health | 0.2% | 16.5% | -0.033 | 0.069 | -0.483 | 0.630 |
| Comm. Exercise Program | 0.1% | 8.1% | 0.017 | 0.052 | 0.325 | 0.745 |
| Comm. Weight Loss | 0.3% | 23.6% | 0.048 | 0.052 | 0.933 | 0.352 |
| Comm. Maintain Health | 0.4% | 32.8% | -0.051 | 0.043 | -1.185 | 0.237 |
| Total R² | 1.3% |  |  |  |  |  |
| Sample Size | 248 |  |  |  |  |  |
| Listwise Deletion | 51 |  |  |  |  |  |
| *Social Status* |  |  |  |  |  |  |
| Intercept |  |  | 1.024 | 0.123 | 8.331 | < 0.001 |
| Education Breakdown | 1.3% | 9.0% | -0.036 | 0.013 | -2.724 | 0.007 |
| Weight Comparison | 0.9% | 6.4% | -0.031 | 0.014 | -2.275 | 0.023 |
| Gender Breakdown | 1.8% | 12.9% | 0.022 | 0.007 | 3.280 | 0.001 |
| Manager | 1.4% | 9.8% | 0.104 | 0.034 | 3.105 | 0.002 |
| Visible Disability | 2.5% | 18.0% | 0.060 | 0.019 | 3.164 | 0.002 |
| Ethnic Name | 1.0% | 7.3% | 0.025 | 0.017 | 1.456 | 0.146 |
| Body-Mass Index | 1.0% | 7.1% | 0.005 | 0.002 | 2.331 | 0.020 |
| Accent | 1.8% | 12.6% | 0.040 | 0.017 | 2.362 | 0.018 |
| Gender | 2.3% | 16.7% | 0.116 | 0.033 | 3.467 | 0.001 |
| Race | 0.0% | 0.3% | 0.013 | 0.039 | 0.335 | 0.738 |
| Total R² | 13.9% |  |  |  |  |  |
| Sample Size | 589 |  |  |  |  |  |
| Listwise Deletion | 169 |  |  |  |  |  |
| *Identity Management Strategies* |  |  |  |  |  |  |
| Intercept |  |  | 1.386 | 0.059 | 23.312 | < 0.001 |
| Redefinition Reassociate | 0.9% | 39.1% | 0.037 | 0.017 | 2.211 | 0.027 |
| Redefinition Regenerate | 0.5% | 23.1% | 0.035 | 0.021 | 1.636 | 0.102 |
| Switch Deemphasize | 0.1% | 6.0% | -0.003 | 0.024 | -0.129 | 0.898 |
| Switch Emphasize | 0.7% | 31.8% | -0.051 | 0.020 | -2.488 | 0.013 |
| Total R² | 2.3% |  |  |  |  |  |
| Sample Size | 754 |  |  |  |  |  |
| Listwise Deletion | 4 |  |  |  |  |  |
| *Objectification by the Self* |  |  |  |  |  |  |
| Intercept |  |  | 1.379 | 0.200 | 6.895 | < 0.001 |
| Anxiety | 2.2% | 17.8% | 0.030 | 0.018 | 1.682 | 0.093 |
| Guilt | 3.2% | 25.4% | 0.059 | 0.018 | 3.316 | 0.001 |
| Shame | 1.1% | 8.9% | 0.031 | 0.023 | 1.324 | 0.186 |
| Depression | 1.8% | 14.6% | 0.018 | 0.024 | 0.755 | 0.450 |
| Core Self-Evaluations | 1.5% | 12.5% | -0.047 | 0.034 | -1.377 | 0.169 |
| Weight-Bias Internalization | 0.7% | 5.5% | -0.065 | 0.028 | -2.331 | 0.020 |
| Objectified Body Consc. | 1.0% | 8.2% | 0.030 | 0.016 | 1.848 | 0.065 |
| Intuitive Eating Trust | 0.1% | 0.7% | -0.006 | 0.014 | -0.430 | 0.667 |
| Body Dissatisfaction | 0.8% | 6.4% | -0.025 | 0.010 | -2.386 | 0.017 |
| Total R² | 12.4% |  |  |  |  |  |
| Sample Size | 732 |  |  |  |  |  |
| Listwise Deletion | 26 |  |  |  |  |  |
| *Objectification by Others* |  |  |  |  |  |  |
| Intercept |  |  | 0.924 | 0.102 | 9.077 | < 0.001 |
| Anti-fat Dislike | 0.8% | 9.8% | -0.004 | 0.036 | -0.109 | 0.913 |
| Anti-fat Fear | 3.1% | 35.5% | 0.103 | 0.044 | 2.374 | 0.018 |
| Anti-fat Willpower | 0.6% | 7.2% | -0.034 | 0.031 | -1.101 | 0.272 |
| Thin Ideal | 2.0% | 22.6% | 0.037 | 0.026 | 1.438 | 0.151 |
| Negative Body Talk Other | 0.6% | 6.7% | -0.011 | 0.035 | -0.300 | 0.764 |
| Negative Body Talk Self | 1.6% | 18.3% | 0.048 | 0.031 | 1.562 | 0.119 |
| Total R² | 8.7% |  |  |  |  |  |
| Sample Size | 298 |  |  |  |  |  |
| Listwise Deletion | 1 |  |  |  |  |  |
| *Job Context* |  |  |  |  |  |  |
| Intercept |  |  | 1.507 | 0.236 | 6.383 | < 0.001 |
| Job Task | 1.0% | 42.6% | -0.207 | 0.067 | -3.096 | 0.002 |
| Job Social | 1.2% | 53.3% | 0.173 | 0.050 | 3.425 | 0.001 |
| Job Physical | 0.1% | 4.2% | -0.015 | 0.040 | -0.373 | 0.709 |
| Total R² | 2.2% |  |  |  |  |  |
| Sample Size | 753 |  |  |  |  |  |
| Listwise Deletion | 5 |  |  |  |  |  |

Same notes as S3 Table.
