# Supplementary material for: An exploratory study of the correlates of weight-based mistreatment at work
Source: PLoS One. 2026 Aug 26;21(8):e0342144. doi: 10.1371/journal.pone.0342144 (PMC13514019; doi:10.1371/journal.pone.0342144)
Supplement: Supplementary file 15 [file pone.0342144.s015.docx]

**S15 Table. Ordinary Least-Squares Regression and Dominance Analysis for WBM by Subordinate.**

| **Correlates** | **GD** | **RGD** | **B** | **SE** | **t** | **p** |
| --- | --- | --- | --- | --- | --- | --- |
| *Stereotypes* |  |  |  |  |  |  |
| Intercept |  |  | 1.771 | 0.121 | 14.676 | < 0.001 |
| Yourself Competent | 0.7% | 7.7% | -0.016 | 0.021 | -0.775 | 0.439 |
| Others Competent | 1.9% | 21.9% | -0.031 | 0.025 | -1.267 | 0.206 |
| Coworker Competent | 1.9% | 21.4% | -0.021 | 0.026 | -0.814 | 0.416 |
| Yourself Warm | 0.3% | 3.2% | 0.028 | 0.026 | 1.109 | 0.268 |
| Others Warm | 0.7% | 8.4% | -0.024 | 0.029 | -0.826 | 0.409 |
| Coworker Warm | 3.3% | 37.4% | -0.068 | 0.028 | -2.417 | 0.016 |
| Total R^2^ | 8.8% |  |  |  |  |  |
| Sample Size | 292 |  |  |  |  |  |
| Listwise Deletion | 7 |  |  |  |  |  |
| *Interpersonal Climate* |  |  |  |  |  |  |
| Intercept |  |  | 1.032 | 0.122 | 8.491 | < 0.001 |
| Climate Incl. Equitable | 0.5% | 6.2% | -0.028 | 0.027 | -1.042 | 0.298 |
| Climate Incl. Integration | 0.3% | 3.1% | -0.010 | 0.034 | -0.299 | 0.765 |
| Climate Incl. DM | 0.2% | 2.5% | 0.024 | 0.029 | 0.837 | 0.403 |
| Civility Climate | 0.7% | 9.0% | -0.015 | 0.027 | -0.545 | 0.586 |
| Leader-Member Exchange | 0.2% | 2.3% | 0.008 | 0.025 | 0.323 | 0.747 |
| Perceived Org. Support | 0.3% | 3.2% | 0.011 | 0.027 | 0.423 | 0.673 |
| Interpersonal Conflict | 6.0% | 73.7% | 0.109 | 0.026 | 4.139 | < 0.001 |
| Total R² | 8.1% |  |  |  |  |  |
| Sample Size | 298 |  |  |  |  |  |
| Listwise Deletion | 1 |  |  |  |  |  |
| *Weight Control Climate* |  |  |  |  |  |  |
| Intercept |  |  | 0.949 | 0.087 | 10.935 | < 0.001 |
| Tolerate Neg. Wgt. Talk | 4.3% | 54.2% | 0.060 | 0.019 | 3.154 | 0.002 |
| Pressure for Thinness | 1.2% | 14.7% | 0.024 | 0.023 | 1.063 | 0.288 |
| Climate for Exercise | 0.1% | 1.7% | -0.018 | 0.034 | -0.521 | 0.603 |
| Wgt. Org. Support | 0.3% | 3.7% | 0.027 | 0.028 | 0.977 | 0.329 |
| Wgt. Social Support | 0.5% | 6.8% | -0.049 | 0.028 | -1.745 | 0.082 |
| Wgt. Diet Norms | 0.9% | 11.6% | 0.053 | 0.029 | 1.827 | 0.069 |
| Wgt. Belittle | 0.6% | 7.2% | 0.013 | 0.020 | 0.634 | 0.527 |
| Total R² | 7.9% |  |  |  |  |  |
| Sample Size | 298 |  |  |  |  |  |
| Listwise Deletion | 1 |  |  |  |  |  |
| *Human Resources Policies* |  |  |  |  |  |  |
| Intercept |  |  | 1.257 | 0.051 | 24.792 | < 0.001 |
| Offer Exercise Program | 0.1% | 5.4% | 0.021 | 0.064 | 0.331 | 0.741 |
| Offer Weight Loss | 0.5% | 21.8% | 0.081 | 0.063 | 1.274 | 0.204 |
| Offer Maintain Health | 0.9% | 37.5% | -0.091 | 0.056 | -1.621 | 0.106 |
| Comm. Exercise Program | 0.2% | 6.3% | -0.027 | 0.043 | -0.630 | 0.530 |
| Comm. Weight Loss | 0.3% | 11.7% | 0.041 | 0.042 | 0.984 | 0.326 |
| Comm. Maintain Health | 0.4% | 17.2% | -0.026 | 0.035 | -0.757 | 0.450 |
| Total R² | 2.4% |  |  |  |  |  |
| Sample Size | 248 |  |  |  |  |  |
| Listwise Deletion | 51 |  |  |  |  |  |
| *Social Status* |  |  |  |  |  |  |
| Intercept |  |  | 1.100 | 0.107 | 10.237 | < 0.001 |
| Education Breakdown | 1.3% | 6.9% | -0.033 | 0.012 | -2.869 | 0.004 |
| Weight Comparison | 0.2% | 0.9% | -0.017 | 0.012 | -1.447 | 0.149 |
| Gender Breakdown | 0.0% | 0.1% | 0.002 | 0.006 | 0.264 | 0.792 |
| Manager | 3.4% | 17.7% | 0.133 | 0.029 | 4.548 | < 0.001 |
| Visible Disability | 5.1% | 26.4% | 0.070 | 0.016 | 4.234 | < 0.001 |
| Ethnic Name | 4.7% | 24.4% | 0.054 | 0.015 | 3.626 | < 0.001 |
| Body-Mass Index | 0.1% | 0.4% | < 0.001 | 0.002 | 0.153 | 0.879 |
| Accent | 4.0% | 20.6% | 0.044 | 0.015 | 2.972 | 0.003 |
| Gender | 0.4% | 2.1% | 0.052 | 0.029 | 1.773 | 0.077 |
| Race | 0.1% | 0.4% | 0.007 | 0.034 | 0.203 | 0.839 |
| Total R² | 19.2% |  |  |  |  |  |
| Sample Size | 588 |  |  |  |  |  |
| Listwise Deletion | 170 |  |  |  |  |  |
| *Identity Management Strategies* |  |  |  |  |  |  |
| Intercept |  |  | 1.222 | 0.054 | 22.546 | < 0.001 |
| Redefinition Reassociate | 1.3% | 35.1% | 0.036 | 0.015 | 2.316 | 0.021 |
| Redefinition Regenerate | 1.4% | 38.1% | 0.053 | 0.019 | 2.722 | 0.007 |
| Switch Deemphasize | 0.1% | 3.7% | 0.013 | 0.022 | 0.605 | 0.545 |
| Switch Emphasize | 0.8% | 23.1% | -0.058 | 0.019 | -3.118 | 0.002 |
| Total R² | 3.7% |  |  |  |  |  |
| Sample Size | 753 |  |  |  |  |  |
| Listwise Deletion | 5 |  |  |  |  |  |
| *Objectification by the Self* |  |  |  |  |  |  |
| Intercept |  |  | 1.328 | 0.184 | 7.199 | < 0.001 |
| Anxiety | 1.4% | 12.0% | 0.033 | 0.017 | 1.974 | 0.049 |
| Guilt | 3.7% | 31.4% | 0.074 | 0.016 | 4.557 | < 0.001 |
| Shame | 0.4% | 3.0% | 0.016 | 0.022 | 0.738 | 0.461 |
| Depression | 0.6% | 4.7% | -0.039 | 0.022 | -1.798 | 0.073 |
| Core Self-Evaluations | 0.7% | 6.1% | -0.047 | 0.032 | -1.497 | 0.135 |
| Weight-Bias Internalization | 0.4% | 3.2% | -0.038 | 0.026 | -1.475 | 0.141 |
| Objectified Body Consc. | 1.3% | 11.3% | 0.036 | 0.015 | 2.414 | 0.016 |
| Intuitive Eating Trust | 0.7% | 5.7% | 0.017 | 0.013 | 1.301 | 0.194 |
| Body Dissatisfaction | 2.7% | 22.5% | -0.039 | 0.010 | -4.058 | < 0.001 |
| Total R² | 11.9% |  |  |  |  |  |
| Sample Size | 731 |  |  |  |  |  |
| Listwise Deletion | 27 |  |  |  |  |  |
| *Objectification by Others* |  |  |  |  |  |  |
| Intercept |  |  | 1.007 | 0.085 | 11.849 | < 0.001 |
| Anti-fat Dislike | 2.4% | 34.6% | 0.059 | 0.030 | 1.966 | 0.050 |
| Anti-fat Fear | 0.9% | 12.7% | 0.008 | 0.036 | 0.208 | 0.836 |
| Anti-fat Willpower | 0.9% | 13.5% | -0.054 | 0.026 | -2.095 | 0.037 |
| Thin Ideal | 0.4% | 5.5% | 0.008 | 0.022 | 0.393 | 0.694 |
| Negative Body Talk Other | 2.0% | 29.2% | 0.051 | 0.029 | 1.740 | 0.083 |
| Negative Body Talk Self | 0.3% | 4.5% | 0.017 | 0.026 | 0.652 | 0.515 |
| Total R² | 6.8% |  |  |  |  |  |
| Sample Size | 298 |  |  |  |  |  |
| Listwise Deletion | 1 |  |  |  |  |  |
| *Job Context* |  |  |  |  |  |  |
| Intercept |  |  | 1.343 | 0.218 | 6.159 | < 0.001 |
| Job Task | 0.2% | 26.3% | -0.089 | 0.062 | -1.444 | 0.149 |
| Job Social | 0.4% | 53.5% | 0.093 | 0.047 | 1.985 | 0.047 |
| Job Physical | 0.2% | 20.2% | -0.033 | 0.037 | -0.901 | 0.368 |
| Total R² | 0.8% |  |  |  |  |  |
| Sample Size | 752 |  |  |  |  |  |
| Listwise Deletion | 6 |  |  |  |  |  |

Same notes as S3 Table.
