# Supplementary material for: An exploratory study of the correlates of weight-based mistreatment at work
Source: PLoS One. 2026 Aug 26;21(8):e0342144. doi: 10.1371/journal.pone.0342144 (PMC13514019; doi:10.1371/journal.pone.0342144)
Supplement: Supplementary file 16 [file pone.0342144.s016.docx]

**S16 Table. Ordinary Least-Squares Regression and Dominance Analysis for WBM by Other.**

| **Correlates** | **GD** | **RGD** | **B** | **SE** | **t** | **p** |
| --- | --- | --- | --- | --- | --- | --- |
| *Stereotypes* |  |  |  |  |  |  |
| Intercept |  |  | 1.688 | 0.136 | 12.448 | < 0.001 |
| Yourself Competent | 0.2% | 5.4% | -0.003 | 0.023 | -0.150 | 0.881 |
| Others Competent | 0.6% | 15.5% | -0.020 | 0.028 | -0.715 | 0.475 |
| Coworker Competent | 1.9% | 47.4% | -0.046 | 0.029 | -1.581 | 0.115 |
| Yourself Warm | 0.1% | 3.0% | -0.009 | 0.029 | -0.325 | 0.745 |
| Others Warm | 0.1% | 2.2% | 0.012 | 0.032 | 0.364 | 0.716 |
| Coworker Warm | 1.0% | 26.5% | -0.027 | 0.032 | -0.847 | 0.397 |
| Total R^2^ | 3.9% |  |  |  |  |  |
| Sample Size | 293 |  |  |  |  |  |
| Listwise Deletion | 6 |  |  |  |  |  |
| *Interpersonal Climate* |  |  |  |  |  |  |
| Intercept |  |  | 1.158 | 0.133 | 8.698 | < 0.001 |
| Climate Incl. Equitable | 0.2% | 2.2% | -0.001 | 0.030 | -0.020 | 0.984 |
| Climate Incl. Integration | 1.1% | 14.7% | -0.072 | 0.037 | -1.959 | 0.051 |
| Climate Incl. DM | 0.7% | 9.2% | 0.061 | 0.031 | 1.955 | 0.052 |
| Civility Climate | 0.5% | 7.0% | -0.008 | 0.029 | -0.259 | 0.795 |
| Leader-Member Exchange | 0.5% | 5.9% | -0.021 | 0.028 | -0.766 | 0.444 |
| Perceived Org. Support | 0.3% | 3.2% | 0.023 | 0.030 | 0.766 | 0.444 |
| Interpersonal Conflict | 4.5% | 57.9% | 0.102 | 0.029 | 3.566 | < 0.001 |
| Total R² | 7.8% |  |  |  |  |  |
| Sample Size | 299 |  |  |  |  |  |
| Listwise Deletion | 0 |  |  |  |  |  |
| *Weight Control Climate* |  |  |  |  |  |  |
| Intercept |  |  | 0.974 | 0.096 | 10.176 | < 0.001 |
| Tolerate Neg. Wgt. Talk | 2.9% | 45.7% | 0.053 | 0.021 | 2.553 | 0.011 |
| Pressure for Thinness | 1.5% | 22.6% | 0.029 | 0.025 | 1.144 | 0.253 |
| Climate for Exercise | 0.5% | 7.4% | 0.021 | 0.038 | 0.566 | 0.572 |
| Wgt. Org. Support | 0.4% | 6.3% | 0.019 | 0.030 | 0.636 | 0.526 |
| Wgt. Social Support | 0.3% | 4.7% | -0.037 | 0.031 | -1.183 | 0.238 |
| Wgt. Diet Norms | 0.6% | 8.7% | 0.032 | 0.032 | 1.021 | 0.308 |
| Wgt. Belittle | 0.3% | 4.6% | 0.008 | 0.022 | 0.365 | 0.715 |
| Total R² | 6.5% |  |  |  |  |  |
| Sample Size | 299 |  |  |  |  |  |
| Listwise Deletion | 0 |  |  |  |  |  |
| *Human Resources Policies* |  |  |  |  |  |  |
| Intercept |  |  | 1.293 | 0.055 | 23.591 | < 0.001 |
| Offer Exercise Program | 0.8% | 22.7% | 0.090 | 0.069 | 1.302 | 0.194 |
| Offer Weight Loss | 0.2% | 5.0% | 0.006 | 0.069 | 0.094 | 0.925 |
| Offer Maintain Health | 0.5% | 13.7% | -0.069 | 0.061 | -1.136 | 0.257 |
| Comm. Exercise Program | 0.3% | 6.9% | 0.009 | 0.046 | 0.193 | 0.847 |
| Comm. Weight Loss | 0.6% | 16.9% | 0.057 | 0.046 | 1.248 | 0.213 |
| Comm. Maintain Health | 1.3% | 34.8% | -0.071 | 0.038 | -1.873 | 0.062 |
| Total R² | 3.6% |  |  |  |  |  |
| Sample Size | 249 |  |  |  |  |  |
| Listwise Deletion | 50 |  |  |  |  |  |
| *Social Status* |  |  |  |  |  |  |
| Intercept |  |  | 1.226 | 0.114 | 10.793 | < 0.001 |
| Education Breakdown | 1.7% | 11.6% | -0.039 | 0.012 | -3.128 | 0.002 |
| Weight Comparison | 0.2% | 1.7% | -0.019 | 0.013 | -1.551 | 0.122 |
| Gender Breakdown | 0.0% | 0.1% | 0.002 | 0.006 | 0.319 | 0.749 |
| Manager | 0.8% | 5.8% | 0.062 | 0.031 | 1.995 | 0.047 |
| Visible Disability | 4.4% | 30.6% | 0.070 | 0.017 | 4.032 | < 0.001 |
| Ethnic Name | 3.9% | 26.9% | 0.053 | 0.016 | 3.332 | 0.001 |
| Body-Mass Index | 0.0% | 0.3% | 0.001 | 0.002 | 0.456 | 0.649 |
| Accent | 3.2% | 22.3% | 0.040 | 0.016 | 2.562 | 0.011 |
| Gender | 0.0% | 0.1% | 0.004 | 0.031 | 0.139 | 0.889 |
| Race | 0.1% | 0.6% | 0.024 | 0.036 | 0.681 | 0.496 |
| Total R² | 14.5% |  |  |  |  |  |
| Sample Size | 590 |  |  |  |  |  |
| Listwise Deletion | 168 |  |  |  |  |  |
| *Identity Management Strategies* |  |  |  |  |  |  |
| Intercept |  |  | 1.248 | 0.055 | 22.516 | < 0.001 |
| Redefinition Reassociate | 1.7% | 43.9% | 0.042 | 0.016 | 2.691 | 0.007 |
| Redefinition Regenerate | 1.5% | 39.8% | 0.051 | 0.020 | 2.601 | 0.009 |
| Switch Deemphasize | 0.1% | 3.4% | 0.011 | 0.022 | 0.478 | 0.633 |
| Switch Emphasize | 0.5% | 12.9% | -0.047 | 0.019 | -2.481 | 0.013 |
| Total R² | 3.8% |  |  |  |  |  |
| Sample Size | 755 |  |  |  |  |  |
| Listwise Deletion | 3 |  |  |  |  |  |
| *Objectification by the Self* |  |  |  |  |  |  |
| Intercept |  |  | 1.400 | 0.188 | 7.455 | < 0.001 |
| Anxiety | 1.9% | 15.3% | 0.044 | 0.017 | 2.577 | 0.010 |
| Guilt | 2.2% | 17.2% | 0.047 | 0.017 | 2.829 | 0.005 |
| Shame | 0.4% | 2.9% | 0.017 | 0.022 | 0.784 | 0.433 |
| Depression | 0.6% | 4.6% | -0.016 | 0.022 | -0.721 | 0.471 |
| Core Self-Evaluations | 0.8% | 6.6% | -0.055 | 0.032 | -1.719 | 0.086 |
| Weight-Bias Internalization | 0.5% | 4.0% | -0.042 | 0.026 | -1.577 | 0.115 |
| Objectified Body Consc. | 1.0% | 7.7% | 0.025 | 0.015 | 1.670 | 0.095 |
| Intuitive Eating Trust | 1.7% | 13.1% | 0.037 | 0.013 | 2.781 | 0.006 |
| Body Dissatisfaction | 3.6% | 28.6% | -0.047 | 0.010 | -4.781 | < 0.001 |
| Total R² | 12.7% |  |  |  |  |  |
| Sample Size | 733 |  |  |  |  |  |
| Listwise Deletion | 25 |  |  |  |  |  |
| *Objectification by Others* |  |  |  |  |  |  |
| Intercept |  |  | 1.007 | 0.093 | 10.840 | < 0.001 |
| Anti-fat Dislike | 1.9% | 30.2% | 0.059 | 0.033 | 1.790 | 0.075 |
| Anti-fat Fear | 0.8% | 12.5% | 0.007 | 0.040 | 0.177 | 0.860 |
| Anti-fat Willpower | 0.5% | 8.8% | -0.044 | 0.028 | -1.532 | 0.127 |
| Thin Ideal | 0.4% | 5.9% | 0.003 | 0.024 | 0.147 | 0.884 |
| Negative Body Talk Other | 1.3% | 20.9% | 0.036 | 0.032 | 1.104 | 0.270 |
| Negative Body Talk Self | 1.3% | 21.7% | 0.051 | 0.028 | 1.804 | 0.072 |
| Total R² | 6.2% |  |  |  |  |  |
| Sample Size | 299 |  |  |  |  |  |
| Listwise Deletion | 0 |  |  |  |  |  |
| *Job Context* |  |  |  |  |  |  |
| Intercept |  |  | 1.277 | 0.224 | 5.703 | < 0.001 |
| Job Task | 0.0% | 13.8% | -0.043 | 0.063 | -0.678 | 0.498 |
| Job Social | 0.2% | 84.7% | 0.063 | 0.048 | 1.314 | 0.189 |
| Job Physical | 0.0% | 1.5% | 0.009 | 0.038 | 0.244 | 0.807 |
| Total R² | 0.2% |  |  |  |  |  |
| Sample Size | 754 |  |  |  |  |  |
| Listwise Deletion | 4 |  |  |  |  |  |

Same notes as S3 Table.
