# Supplementary material for: An exploratory study of the correlates of weight-based mistreatment at work
Source: PLoS One. 2026 Aug 26;21(8):e0342144. doi: 10.1371/journal.pone.0342144 (PMC13514019; doi:10.1371/journal.pone.0342144)
Supplement: Supplementary file 17 [file pone.0342144.s017.docx]

**S17 Table. Correlations Between All Antecedent Correlates.**

| **Variable 1** | **Variable 2** | **r** | **t** | **df** | **p** |
| --- | --- | --- | --- | --- | --- |
| Yourself Competent | Others Competent | 0.60 | 23.87 | 997 | < 0.001 |
| Yourself Competent | Coworker Competent | 0.24 | 4.84 | 379 | < 0.001 |
| Yourself Competent | Yourself Warm | 0.48 | 17.26 | 997 | < 0.001 |
| Yourself Competent | Others Warm | 0.35 | 11.66 | 998 | < 0.001 |
| Yourself Competent | Coworker Warm | 0.14 | 2.77 | 377 | 0.006 |
| Yourself Competent | Climate Incl. Equitable | 0.16 | 3.20 | 379 | 0.001 |
| Yourself Competent | Climate Incl. Integration | 0.15 | 3.02 | 379 | 0.003 |
| Yourself Competent | Climate Incl. DM | 0.18 | 3.59 | 379 | < 0.001 |
| Yourself Competent | Civility Climate | 0.12 | 2.44 | 379 | 0.015 |
| Yourself Competent | Leader-Member Exchange | 0.19 | 3.73 | 378 | < 0.001 |
| Yourself Competent | Perceived Org. Support | 0.12 | 2.29 | 379 | 0.023 |
| Yourself Competent | Interpersonal Conflict | -0.14 | -2.73 | 379 | 0.007 |
| Yourself Competent | Tolerate Neg. Wgt. Talk | -0.09 | -1.75 | 379 | 0.080 |
| Yourself Competent | Pressure for Thinness | 0.00 | -0.01 | 379 | 0.989 |
| Yourself Competent | Climate for Exercise | 0.10 | 1.93 | 379 | 0.054 |
| Yourself Competent | Wgt. Org. Support | 0.07 | 1.41 | 379 | 0.158 |
| Yourself Competent | Wgt. Social Support | 0.02 | 0.44 | 379 | 0.660 |
| Yourself Competent | Wgt. Diet Norms | 0.04 | 0.82 | 379 | 0.415 |
| Yourself Competent | Wgt. Belittle | -0.10 | -2.05 | 379 | 0.041 |
| Yourself Competent | Offer Exercise Program | 0.06 | 1.21 | 379 | 0.227 |
| Yourself Competent | Offer Weight Loss | 0.01 | 0.14 | 379 | 0.893 |
| Yourself Competent | Offer Maintain Health | 0.06 | 1.08 | 378 | 0.280 |
| Yourself Competent | Comm. Exercise Program | 0.04 | 0.68 | 330 | 0.497 |
| Yourself Competent | Comm. Weight Loss | 0.04 | 0.74 | 325 | 0.458 |
| Yourself Competent | Comm. Maintain Health | -0.01 | -0.23 | 334 | 0.822 |
| Yourself Competent | Education Breakdown | 0.06 | 1.86 | 993 | 0.064 |
| Yourself Competent | Weight Comparison | 0.06 | 2.02 | 991 | 0.044 |
| Yourself Competent | Gender Breakdown | -0.05 | -1.52 | 998 | 0.129 |
| Yourself Competent | Visible Disability | -0.08 | -2.65 | 996 | 0.008 |
| Yourself Competent | Ethnic Name | -0.04 | -1.23 | 998 | 0.218 |
| Yourself Competent | Body-Mass Index | -0.05 | -1.40 | 805 | 0.163 |
| Yourself Competent | Accent | -0.09 | -2.79 | 998 | 0.005 |
| Yourself Competent | Redefinition Reassociate | 0.27 | 8.74 | 1000 | < 0.001 |
| Yourself Competent | Redefinition Regenerate | 0.25 | 8.30 | 1000 | < 0.001 |
| Yourself Competent | Switch Deemphasize | 0.33 | 11.01 | 1000 | < 0.001 |
| Yourself Competent | Switch Emphasize | 0.40 | 14.00 | 1000 | < 0.001 |
| Yourself Competent | Anxiety | -0.34 | -11.42 | 1000 | < 0.001 |
| Yourself Competent | Guilt | -0.35 | -11.94 | 998 | < 0.001 |
| Yourself Competent | Shame | -0.31 | -10.21 | 1000 | < 0.001 |
| Yourself Competent | Depression | -0.38 | -12.99 | 999 | < 0.001 |
| Yourself Competent | Core Self-Evaluations | 0.51 | 18.75 | 1000 | < 0.001 |
| Yourself Competent | Weight-Bias Internalization | -0.32 | -10.65 | 1000 | < 0.001 |
| Yourself Competent | Objectified Body Consc. | -0.13 | -4.02 | 1000 | < 0.001 |
| Yourself Competent | Intuitive Eating Trust | 0.18 | 5.84 | 1000 | < 0.001 |
| Yourself Competent | Body Dissatisfaction | -0.10 | -3.23 | 979 | 0.001 |
| Yourself Competent | Anti-fat Dislike | -0.09 | -1.71 | 379 | 0.087 |
| Yourself Competent | Anti-fat Fear | -0.06 | -1.16 | 379 | 0.245 |
| Yourself Competent | Anti-fat Willpower | -0.02 | -0.47 | 379 | 0.642 |
| Yourself Competent | Thin Ideal | -0.08 | -1.48 | 378 | 0.140 |
| Yourself Competent | Negative Body Talk Other | -0.12 | -2.44 | 379 | 0.015 |
| Yourself Competent | Negative Body Talk Self | 0.06 | 1.18 | 379 | 0.238 |
| Yourself Competent | Job Task | 0.05 | 1.54 | 999 | 0.125 |
| Yourself Competent | Job Social | 0.00 | 0.06 | 999 | 0.951 |
| Yourself Competent | Job Physical | -0.01 | -0.32 | 999 | 0.749 |
| Others Competent | Coworker Competent | 0.38 | 7.97 | 379 | < 0.001 |
| Others Competent | Yourself Warm | 0.41 | 14.27 | 999 | < 0.001 |
| Others Competent | Others Warm | 0.54 | 20.07 | 1000 | < 0.001 |
| Others Competent | Coworker Warm | 0.28 | 5.68 | 377 | < 0.001 |
| Others Competent | Climate Incl. Equitable | 0.16 | 3.22 | 379 | 0.001 |
| Others Competent | Climate Incl. Integration | 0.21 | 4.26 | 379 | < 0.001 |
| Others Competent | Climate Incl. DM | 0.23 | 4.63 | 379 | < 0.001 |
| Others Competent | Civility Climate | 0.24 | 4.83 | 379 | < 0.001 |
| Others Competent | Leader-Member Exchange | 0.28 | 5.71 | 378 | < 0.001 |
| Others Competent | Perceived Org. Support | 0.25 | 5.00 | 379 | < 0.001 |
| Others Competent | Interpersonal Conflict | -0.20 | -3.90 | 379 | < 0.001 |
| Others Competent | Tolerate Neg. Wgt. Talk | -0.16 | -3.16 | 379 | 0.002 |
| Others Competent | Pressure for Thinness | -0.02 | -0.31 | 379 | 0.757 |
| Others Competent | Climate for Exercise | 0.09 | 1.84 | 379 | 0.066 |
| Others Competent | Wgt. Org. Support | 0.08 | 1.63 | 379 | 0.103 |
| Others Competent | Wgt. Social Support | 0.05 | 1.01 | 379 | 0.314 |
| Others Competent | Wgt. Diet Norms | -0.01 | -0.17 | 379 | 0.865 |
| Others Competent | Wgt. Belittle | -0.10 | -1.92 | 379 | 0.055 |
| Others Competent | Offer Exercise Program | 0.02 | 0.46 | 379 | 0.646 |
| Others Competent | Offer Weight Loss | 0.00 | -0.07 | 379 | 0.941 |
| Others Competent | Offer Maintain Health | 0.03 | 0.59 | 378 | 0.559 |
| Others Competent | Comm. Exercise Program | 0.08 | 1.38 | 330 | 0.170 |
| Others Competent | Comm. Weight Loss | 0.05 | 0.95 | 325 | 0.343 |
| Others Competent | Comm. Maintain Health | 0.04 | 0.75 | 334 | 0.454 |
| Others Competent | Education Breakdown | 0.08 | 2.46 | 995 | 0.014 |
| Others Competent | Weight Comparison | -0.01 | -0.35 | 993 | 0.726 |
| Others Competent | Gender Breakdown | 0.02 | 0.63 | 1000 | 0.530 |
| Others Competent | Visible Disability | -0.12 | -3.68 | 998 | < 0.001 |
| Others Competent | Ethnic Name | -0.07 | -2.27 | 1000 | 0.023 |
| Others Competent | Body-Mass Index | 0.02 | 0.66 | 808 | 0.511 |
| Others Competent | Accent | -0.09 | -2.79 | 1000 | 0.005 |
| Others Competent | Redefinition Reassociate | 0.20 | 6.58 | 1002 | < 0.001 |
| Others Competent | Redefinition Regenerate | 0.19 | 6.13 | 1002 | < 0.001 |
| Others Competent | Switch Deemphasize | 0.33 | 11.19 | 1002 | < 0.001 |
| Others Competent | Switch Emphasize | 0.41 | 14.24 | 1002 | < 0.001 |
| Others Competent | Anxiety | -0.27 | -8.77 | 1002 | < 0.001 |
| Others Competent | Guilt | -0.29 | -9.50 | 1001 | < 0.001 |
| Others Competent | Shame | -0.22 | -7.03 | 1002 | < 0.001 |
| Others Competent | Depression | -0.30 | -10.13 | 1001 | < 0.001 |
| Others Competent | Core Self-Evaluations | 0.44 | 15.73 | 1002 | < 0.001 |
| Others Competent | Weight-Bias Internalization | -0.23 | -7.42 | 1002 | < 0.001 |
| Others Competent | Objectified Body Consc. | -0.15 | -4.97 | 1002 | < 0.001 |
| Others Competent | Intuitive Eating Trust | 0.12 | 3.70 | 1002 | < 0.001 |
| Others Competent | Body Dissatisfaction | 0.01 | 0.20 | 981 | 0.842 |
| Others Competent | Anti-fat Dislike | -0.13 | -2.45 | 379 | 0.015 |
| Others Competent | Anti-fat Fear | -0.08 | -1.59 | 379 | 0.113 |
| Others Competent | Anti-fat Willpower | -0.02 | -0.45 | 379 | 0.655 |
| Others Competent | Thin Ideal | -0.08 | -1.53 | 378 | 0.128 |
| Others Competent | Negative Body Talk Other | -0.19 | -3.83 | 379 | < 0.001 |
| Others Competent | Negative Body Talk Self | 0.07 | 1.28 | 379 | 0.202 |
| Others Competent | Job Task | 0.01 | 0.27 | 1001 | 0.787 |
| Others Competent | Job Social | -0.01 | -0.46 | 1001 | 0.645 |
| Others Competent | Job Physical | -0.01 | -0.26 | 1001 | 0.794 |
| Coworker Competent | Yourself Warm | 0.15 | 2.98 | 380 | 0.003 |
| Coworker Competent | Others Warm | 0.17 | 3.44 | 381 | 0.001 |
| Coworker Competent | Coworker Warm | 0.58 | 13.82 | 379 | < 0.001 |
| Coworker Competent | Climate Incl. Equitable | 0.22 | 4.40 | 381 | < 0.001 |
| Coworker Competent | Climate Incl. Integration | 0.29 | 6.02 | 381 | < 0.001 |
| Coworker Competent | Climate Incl. DM | 0.24 | 4.87 | 381 | < 0.001 |
| Coworker Competent | Civility Climate | 0.28 | 5.72 | 381 | < 0.001 |
| Coworker Competent | Leader-Member Exchange | 0.28 | 5.75 | 380 | < 0.001 |
| Coworker Competent | Perceived Org. Support | 0.24 | 4.75 | 381 | < 0.001 |
| Coworker Competent | Interpersonal Conflict | -0.29 | -5.83 | 381 | < 0.001 |
| Coworker Competent | Tolerate Neg. Wgt. Talk | -0.14 | -2.66 | 381 | 0.008 |
| Coworker Competent | Pressure for Thinness | -0.08 | -1.62 | 381 | 0.105 |
| Coworker Competent | Climate for Exercise | -0.02 | -0.31 | 381 | 0.755 |
| Coworker Competent | Wgt. Org. Support | 0.00 | 0.04 | 381 | 0.971 |
| Coworker Competent | Wgt. Social Support | -0.03 | -0.52 | 381 | 0.602 |
| Coworker Competent | Wgt. Diet Norms | -0.11 | -2.24 | 381 | 0.026 |
| Coworker Competent | Wgt. Belittle | -0.21 | -4.14 | 381 | < 0.001 |
| Coworker Competent | Offer Exercise Program | -0.03 | -0.67 | 381 | 0.502 |
| Coworker Competent | Offer Weight Loss | -0.02 | -0.33 | 381 | 0.741 |
| Coworker Competent | Offer Maintain Health | 0.00 | -0.01 | 380 | 0.993 |
| Coworker Competent | Comm. Exercise Program | -0.03 | -0.57 | 332 | 0.569 |
| Coworker Competent | Comm. Weight Loss | -0.07 | -1.27 | 327 | 0.204 |
| Coworker Competent | Comm. Maintain Health | 0.02 | 0.36 | 336 | 0.716 |
| Coworker Competent | Education Breakdown | 0.06 | 1.12 | 380 | 0.262 |
| Coworker Competent | Weight Comparison | -0.07 | -1.33 | 377 | 0.184 |
| Coworker Competent | Gender Breakdown | 0.06 | 1.09 | 381 | 0.278 |
| Coworker Competent | Visible Disability | -0.17 | -3.27 | 378 | 0.001 |
| Coworker Competent | Ethnic Name | -0.07 | -1.31 | 381 | 0.190 |
| Coworker Competent | Body-Mass Index | 0.02 | 0.29 | 314 | 0.774 |
| Coworker Competent | Accent | -0.08 | -1.66 | 381 | 0.098 |
| Coworker Competent | Redefinition Reassociate | 0.08 | 1.49 | 381 | 0.138 |
| Coworker Competent | Redefinition Regenerate | 0.05 | 0.91 | 381 | 0.362 |
| Coworker Competent | Switch Deemphasize | 0.09 | 1.76 | 381 | 0.080 |
| Coworker Competent | Switch Emphasize | 0.17 | 3.33 | 381 | 0.001 |
| Coworker Competent | Anxiety | -0.14 | -2.78 | 381 | 0.006 |
| Coworker Competent | Guilt | -0.17 | -3.46 | 380 | 0.001 |
| Coworker Competent | Shame | -0.07 | -1.37 | 381 | 0.170 |
| Coworker Competent | Depression | -0.16 | -3.25 | 381 | 0.001 |
| Coworker Competent | Core Self-Evaluations | 0.17 | 3.34 | 381 | 0.001 |
| Coworker Competent | Weight-Bias Internalization | -0.07 | -1.39 | 381 | 0.166 |
| Coworker Competent | Objectified Body Consc. | -0.02 | -0.34 | 381 | 0.732 |
| Coworker Competent | Intuitive Eating Trust | 0.08 | 1.52 | 381 | 0.129 |
| Coworker Competent | Body Dissatisfaction | 0.01 | 0.12 | 377 | 0.904 |
| Coworker Competent | Anti-fat Dislike | -0.26 | -5.19 | 381 | < 0.001 |
| Coworker Competent | Anti-fat Fear | -0.19 | -3.76 | 381 | < 0.001 |
| Coworker Competent | Anti-fat Willpower | -0.09 | -1.81 | 381 | 0.072 |
| Coworker Competent | Thin Ideal | -0.03 | -0.66 | 380 | 0.510 |
| Coworker Competent | Negative Body Talk Other | -0.25 | -5.05 | 381 | < 0.001 |
| Coworker Competent | Negative Body Talk Self | 0.11 | 2.17 | 381 | 0.031 |
| Coworker Competent | Job Task | 0.03 | 0.66 | 381 | 0.508 |
| Coworker Competent | Job Social | 0.07 | 1.44 | 381 | 0.150 |
| Coworker Competent | Job Physical | 0.03 | 0.64 | 381 | 0.524 |
| Yourself Warm | Others Warm | 0.63 | 25.70 | 1001 | < 0.001 |
| Yourself Warm | Coworker Warm | 0.25 | 5.09 | 378 | < 0.001 |
| Yourself Warm | Climate Incl. Equitable | 0.07 | 1.35 | 380 | 0.176 |
| Yourself Warm | Climate Incl. Integration | 0.12 | 2.26 | 380 | 0.024 |
| Yourself Warm | Climate Incl. DM | 0.10 | 2.05 | 380 | 0.041 |
| Yourself Warm | Civility Climate | 0.06 | 1.19 | 380 | 0.235 |
| Yourself Warm | Leader-Member Exchange | 0.18 | 3.53 | 379 | < 0.001 |
| Yourself Warm | Perceived Org. Support | 0.06 | 1.20 | 380 | 0.231 |
| Yourself Warm | Interpersonal Conflict | -0.18 | -3.53 | 380 | < 0.001 |
| Yourself Warm | Tolerate Neg. Wgt. Talk | 0.00 | 0.03 | 380 | 0.973 |
| Yourself Warm | Pressure for Thinness | 0.06 | 1.12 | 380 | 0.262 |
| Yourself Warm | Climate for Exercise | 0.08 | 1.63 | 380 | 0.103 |
| Yourself Warm | Wgt. Org. Support | 0.05 | 0.95 | 380 | 0.344 |
| Yourself Warm | Wgt. Social Support | 0.11 | 2.12 | 380 | 0.035 |
| Yourself Warm | Wgt. Diet Norms | 0.04 | 0.75 | 380 | 0.455 |
| Yourself Warm | Wgt. Belittle | -0.03 | -0.67 | 380 | 0.503 |
| Yourself Warm | Offer Exercise Program | 0.03 | 0.49 | 380 | 0.622 |
| Yourself Warm | Offer Weight Loss | 0.00 | -0.09 | 380 | 0.928 |
| Yourself Warm | Offer Maintain Health | -0.01 | -0.26 | 379 | 0.793 |
| Yourself Warm | Comm. Exercise Program | 0.02 | 0.34 | 331 | 0.735 |
| Yourself Warm | Comm. Weight Loss | 0.01 | 0.11 | 326 | 0.915 |
| Yourself Warm | Comm. Maintain Health | -0.05 | -0.92 | 335 | 0.359 |
| Yourself Warm | Education Breakdown | -0.02 | -0.62 | 995 | 0.535 |
| Yourself Warm | Weight Comparison | 0.01 | 0.26 | 993 | 0.791 |
| Yourself Warm | Gender Breakdown | 0.02 | 0.56 | 1000 | 0.573 |
| Yourself Warm | Visible Disability | -0.13 | -3.98 | 998 | < 0.001 |
| Yourself Warm | Ethnic Name | -0.05 | -1.57 | 1000 | 0.116 |
| Yourself Warm | Body-Mass Index | 0.06 | 1.74 | 807 | 0.082 |
| Yourself Warm | Accent | -0.09 | -2.80 | 1000 | 0.005 |
| Yourself Warm | Redefinition Reassociate | 0.12 | 3.73 | 1002 | < 0.001 |
| Yourself Warm | Redefinition Regenerate | 0.21 | 6.65 | 1002 | < 0.001 |
| Yourself Warm | Switch Deemphasize | 0.30 | 9.88 | 1002 | < 0.001 |
| Yourself Warm | Switch Emphasize | 0.39 | 13.54 | 1002 | < 0.001 |
| Yourself Warm | Anxiety | -0.20 | -6.29 | 1002 | < 0.001 |
| Yourself Warm | Guilt | -0.25 | -8.04 | 1000 | < 0.001 |
| Yourself Warm | Shame | -0.11 | -3.62 | 1002 | < 0.001 |
| Yourself Warm | Depression | -0.23 | -7.58 | 1001 | < 0.001 |
| Yourself Warm | Core Self-Evaluations | 0.33 | 10.97 | 1002 | < 0.001 |
| Yourself Warm | Weight-Bias Internalization | -0.15 | -4.64 | 1002 | < 0.001 |
| Yourself Warm | Objectified Body Consc. | -0.07 | -2.18 | 1002 | 0.029 |
| Yourself Warm | Intuitive Eating Trust | 0.14 | 4.33 | 1002 | < 0.001 |
| Yourself Warm | Body Dissatisfaction | -0.04 | -1.16 | 981 | 0.248 |
| Yourself Warm | Anti-fat Dislike | -0.02 | -0.41 | 380 | 0.680 |
| Yourself Warm | Anti-fat Fear | 0.00 | -0.09 | 380 | 0.932 |
| Yourself Warm | Anti-fat Willpower | 0.05 | 1.06 | 380 | 0.291 |
| Yourself Warm | Thin Ideal | 0.01 | 0.16 | 379 | 0.873 |
| Yourself Warm | Negative Body Talk Other | -0.07 | -1.39 | 380 | 0.166 |
| Yourself Warm | Negative Body Talk Self | 0.04 | 0.84 | 380 | 0.399 |
| Yourself Warm | Job Task | -0.02 | -0.64 | 1001 | 0.524 |
| Yourself Warm | Job Social | 0.01 | 0.31 | 1001 | 0.754 |
| Yourself Warm | Job Physical | 0.00 | -0.05 | 1001 | 0.961 |
| Others Warm | Coworker Warm | 0.30 | 6.02 | 379 | < 0.001 |
| Others Warm | Climate Incl. Equitable | 0.05 | 0.96 | 381 | 0.339 |
| Others Warm | Climate Incl. Integration | 0.13 | 2.53 | 381 | 0.012 |
| Others Warm | Climate Incl. DM | 0.11 | 2.14 | 381 | 0.033 |
| Others Warm | Civility Climate | 0.09 | 1.72 | 381 | 0.085 |
| Others Warm | Leader-Member Exchange | 0.18 | 3.57 | 380 | < 0.001 |
| Others Warm | Perceived Org. Support | 0.10 | 1.92 | 381 | 0.055 |
| Others Warm | Interpersonal Conflict | -0.12 | -2.29 | 381 | 0.022 |
| Others Warm | Tolerate Neg. Wgt. Talk | -0.02 | -0.44 | 381 | 0.661 |
| Others Warm | Pressure for Thinness | 0.02 | 0.42 | 381 | 0.672 |
| Others Warm | Climate for Exercise | 0.03 | 0.52 | 381 | 0.606 |
| Others Warm | Wgt. Org. Support | 0.00 | 0.08 | 381 | 0.933 |
| Others Warm | Wgt. Social Support | 0.05 | 0.91 | 381 | 0.363 |
| Others Warm | Wgt. Diet Norms | 0.00 | 0.05 | 381 | 0.958 |
| Others Warm | Wgt. Belittle | -0.04 | -0.85 | 381 | 0.393 |
| Others Warm | Offer Exercise Program | -0.04 | -0.74 | 381 | 0.459 |
| Others Warm | Offer Weight Loss | -0.08 | -1.64 | 381 | 0.102 |
| Others Warm | Offer Maintain Health | -0.06 | -1.20 | 380 | 0.231 |
| Others Warm | Comm. Exercise Program | 0.01 | 0.12 | 332 | 0.907 |
| Others Warm | Comm. Weight Loss | -0.04 | -0.66 | 327 | 0.508 |
| Others Warm | Comm. Maintain Health | -0.05 | -0.84 | 336 | 0.402 |
| Others Warm | Education Breakdown | 0.04 | 1.17 | 996 | 0.244 |
| Others Warm | Weight Comparison | 0.00 | 0.02 | 994 | 0.988 |
| Others Warm | Gender Breakdown | 0.03 | 1.02 | 1001 | 0.310 |
| Others Warm | Visible Disability | -0.14 | -4.32 | 999 | < 0.001 |
| Others Warm | Ethnic Name | -0.07 | -2.32 | 1001 | 0.020 |
| Others Warm | Body-Mass Index | 0.12 | 3.53 | 808 | < 0.001 |
| Others Warm | Accent | -0.09 | -2.91 | 1001 | 0.004 |
| Others Warm | Redefinition Reassociate | 0.12 | 3.75 | 1003 | < 0.001 |
| Others Warm | Redefinition Regenerate | 0.19 | 6.08 | 1003 | < 0.001 |
| Others Warm | Switch Deemphasize | 0.31 | 10.15 | 1003 | < 0.001 |
| Others Warm | Switch Emphasize | 0.38 | 13.21 | 1003 | < 0.001 |
| Others Warm | Anxiety | -0.17 | -5.59 | 1003 | < 0.001 |
| Others Warm | Guilt | -0.21 | -6.81 | 1001 | < 0.001 |
| Others Warm | Shame | -0.08 | -2.44 | 1003 | 0.015 |
| Others Warm | Depression | -0.21 | -6.91 | 1002 | < 0.001 |
| Others Warm | Core Self-Evaluations | 0.28 | 9.09 | 1003 | < 0.001 |
| Others Warm | Weight-Bias Internalization | -0.09 | -2.89 | 1003 | 0.004 |
| Others Warm | Objectified Body Consc. | -0.05 | -1.58 | 1003 | 0.115 |
| Others Warm | Intuitive Eating Trust | 0.10 | 3.10 | 1003 | 0.002 |
| Others Warm | Body Dissatisfaction | 0.03 | 0.90 | 982 | 0.367 |
| Others Warm | Anti-fat Dislike | -0.10 | -1.90 | 381 | 0.058 |
| Others Warm | Anti-fat Fear | -0.04 | -0.77 | 381 | 0.440 |
| Others Warm | Anti-fat Willpower | 0.04 | 0.71 | 381 | 0.480 |
| Others Warm | Thin Ideal | 0.03 | 0.53 | 380 | 0.594 |
| Others Warm | Negative Body Talk Other | -0.10 | -1.99 | 381 | 0.047 |
| Others Warm | Negative Body Talk Self | 0.08 | 1.66 | 381 | 0.098 |
| Others Warm | Job Task | -0.03 | -1.06 | 1002 | 0.289 |
| Others Warm | Job Social | 0.03 | 0.81 | 1002 | 0.419 |
| Others Warm | Job Physical | 0.00 | 0.00 | 1002 | 0.998 |
| Coworker Warm | Climate Incl. Equitable | 0.18 | 3.63 | 379 | < 0.001 |
| Coworker Warm | Climate Incl. Integration | 0.24 | 4.75 | 379 | < 0.001 |
| Coworker Warm | Climate Incl. DM | 0.19 | 3.76 | 379 | < 0.001 |
| Coworker Warm | Civility Climate | 0.23 | 4.70 | 379 | < 0.001 |
| Coworker Warm | Leader-Member Exchange | 0.21 | 4.19 | 378 | < 0.001 |
| Coworker Warm | Perceived Org. Support | 0.24 | 4.77 | 379 | < 0.001 |
| Coworker Warm | Interpersonal Conflict | -0.27 | -5.49 | 379 | < 0.001 |
| Coworker Warm | Tolerate Neg. Wgt. Talk | -0.07 | -1.31 | 379 | 0.190 |
| Coworker Warm | Pressure for Thinness | 0.06 | 1.17 | 379 | 0.244 |
| Coworker Warm | Climate for Exercise | 0.05 | 0.98 | 379 | 0.326 |
| Coworker Warm | Wgt. Org. Support | 0.05 | 1.01 | 379 | 0.314 |
| Coworker Warm | Wgt. Social Support | 0.04 | 0.84 | 379 | 0.400 |
| Coworker Warm | Wgt. Diet Norms | -0.06 | -1.13 | 379 | 0.260 |
| Coworker Warm | Wgt. Belittle | -0.11 | -2.15 | 379 | 0.032 |
| Coworker Warm | Offer Exercise Program | -0.03 | -0.49 | 379 | 0.626 |
| Coworker Warm | Offer Weight Loss | -0.01 | -0.22 | 379 | 0.829 |
| Coworker Warm | Offer Maintain Health | 0.03 | 0.56 | 378 | 0.576 |
| Coworker Warm | Comm. Exercise Program | 0.02 | 0.44 | 330 | 0.662 |
| Coworker Warm | Comm. Weight Loss | 0.00 | -0.06 | 325 | 0.951 |
| Coworker Warm | Comm. Maintain Health | 0.08 | 1.39 | 334 | 0.167 |
| Coworker Warm | Education Breakdown | -0.02 | -0.47 | 378 | 0.640 |
| Coworker Warm | Weight Comparison | -0.04 | -0.84 | 375 | 0.400 |
| Coworker Warm | Gender Breakdown | 0.04 | 0.73 | 379 | 0.466 |
| Coworker Warm | Visible Disability | -0.13 | -2.47 | 376 | 0.014 |
| Coworker Warm | Ethnic Name | -0.06 | -1.21 | 379 | 0.227 |
| Coworker Warm | Body-Mass Index | 0.06 | 1.04 | 313 | 0.299 |
| Coworker Warm | Accent | -0.05 | -0.89 | 379 | 0.376 |
| Coworker Warm | Redefinition Reassociate | 0.03 | 0.50 | 379 | 0.616 |
| Coworker Warm | Redefinition Regenerate | 0.02 | 0.43 | 379 | 0.671 |
| Coworker Warm | Switch Deemphasize | 0.11 | 2.11 | 379 | 0.036 |
| Coworker Warm | Switch Emphasize | 0.18 | 3.55 | 379 | < 0.001 |
| Coworker Warm | Anxiety | -0.06 | -1.15 | 379 | 0.249 |
| Coworker Warm | Guilt | -0.09 | -1.76 | 378 | 0.079 |
| Coworker Warm | Shame | -0.03 | -0.64 | 379 | 0.521 |
| Coworker Warm | Depression | -0.07 | -1.32 | 379 | 0.189 |
| Coworker Warm | Core Self-Evaluations | 0.13 | 2.47 | 379 | 0.014 |
| Coworker Warm | Weight-Bias Internalization | 0.01 | 0.23 | 379 | 0.818 |
| Coworker Warm | Objectified Body Consc. | -0.06 | -1.10 | 379 | 0.273 |
| Coworker Warm | Intuitive Eating Trust | -0.02 | -0.39 | 379 | 0.699 |
| Coworker Warm | Body Dissatisfaction | 0.03 | 0.53 | 375 | 0.593 |
| Coworker Warm | Anti-fat Dislike | -0.18 | -3.49 | 379 | 0.001 |
| Coworker Warm | Anti-fat Fear | -0.10 | -1.87 | 379 | 0.062 |
| Coworker Warm | Anti-fat Willpower | -0.06 | -1.08 | 379 | 0.279 |
| Coworker Warm | Thin Ideal | 0.02 | 0.30 | 378 | 0.762 |
| Coworker Warm | Negative Body Talk Other | -0.18 | -3.61 | 379 | < 0.001 |
| Coworker Warm | Negative Body Talk Self | 0.09 | 1.78 | 379 | 0.075 |
| Coworker Warm | Job Task | 0.06 | 1.13 | 379 | 0.258 |
| Coworker Warm | Job Social | 0.05 | 0.96 | 379 | 0.339 |
| Coworker Warm | Job Physical | 0.05 | 0.88 | 379 | 0.381 |
| Climate Incl. Equitable | Climate Incl. Integration | 0.72 | 20.43 | 381 | < 0.001 |
| Climate Incl. Equitable | Climate Incl. DM | 0.69 | 18.84 | 381 | < 0.001 |
| Climate Incl. Equitable | Civility Climate | 0.46 | 10.24 | 381 | < 0.001 |
| Climate Incl. Equitable | Leader-Member Exchange | 0.53 | 12.08 | 380 | < 0.001 |
| Climate Incl. Equitable | Perceived Org. Support | 0.56 | 13.07 | 381 | < 0.001 |
| Climate Incl. Equitable | Interpersonal Conflict | -0.34 | -7.01 | 381 | < 0.001 |
| Climate Incl. Equitable | Tolerate Neg. Wgt. Talk | -0.32 | -6.64 | 381 | < 0.001 |
| Climate Incl. Equitable | Pressure for Thinness | -0.08 | -1.58 | 381 | 0.114 |
| Climate Incl. Equitable | Climate for Exercise | 0.24 | 4.77 | 381 | < 0.001 |
| Climate Incl. Equitable | Wgt. Org. Support | 0.29 | 5.96 | 381 | < 0.001 |
| Climate Incl. Equitable | Wgt. Social Support | 0.13 | 2.50 | 381 | 0.013 |
| Climate Incl. Equitable | Wgt. Diet Norms | 0.18 | 3.66 | 381 | < 0.001 |
| Climate Incl. Equitable | Wgt. Belittle | -0.30 | -6.12 | 381 | < 0.001 |
| Climate Incl. Equitable | Offer Exercise Program | 0.19 | 3.70 | 381 | < 0.001 |
| Climate Incl. Equitable | Offer Weight Loss | 0.18 | 3.51 | 381 | 0.001 |
| Climate Incl. Equitable | Offer Maintain Health | 0.22 | 4.40 | 380 | < 0.001 |
| Climate Incl. Equitable | Comm. Exercise Program | 0.25 | 4.66 | 332 | < 0.001 |
| Climate Incl. Equitable | Comm. Weight Loss | 0.25 | 4.57 | 327 | < 0.001 |
| Climate Incl. Equitable | Comm. Maintain Health | 0.26 | 4.88 | 336 | < 0.001 |
| Climate Incl. Equitable | Education Breakdown | -0.09 | -1.79 | 380 | 0.074 |
| Climate Incl. Equitable | Weight Comparison | 0.01 | 0.28 | 377 | 0.782 |
| Climate Incl. Equitable | Gender Breakdown | -0.08 | -1.49 | 381 | 0.138 |
| Climate Incl. Equitable | Visible Disability | -0.07 | -1.40 | 378 | 0.161 |
| Climate Incl. Equitable | Ethnic Name | -0.09 | -1.70 | 381 | 0.090 |
| Climate Incl. Equitable | Body-Mass Index | -0.04 | -0.79 | 314 | 0.431 |
| Climate Incl. Equitable | Accent | -0.14 | -2.69 | 381 | 0.007 |
| Climate Incl. Equitable | Redefinition Reassociate | 0.17 | 3.40 | 381 | 0.001 |
| Climate Incl. Equitable | Redefinition Regenerate | 0.09 | 1.69 | 381 | 0.093 |
| Climate Incl. Equitable | Switch Deemphasize | 0.09 | 1.80 | 381 | 0.072 |
| Climate Incl. Equitable | Switch Emphasize | 0.15 | 3.04 | 381 | 0.003 |
| Climate Incl. Equitable | Anxiety | -0.22 | -4.44 | 381 | < 0.001 |
| Climate Incl. Equitable | Guilt | -0.20 | -4.01 | 380 | < 0.001 |
| Climate Incl. Equitable | Shame | -0.17 | -3.45 | 381 | 0.001 |
| Climate Incl. Equitable | Depression | -0.25 | -5.12 | 381 | < 0.001 |
| Climate Incl. Equitable | Core Self-Evaluations | 0.31 | 6.31 | 381 | < 0.001 |
| Climate Incl. Equitable | Weight-Bias Internalization | -0.12 | -2.33 | 381 | 0.020 |
| Climate Incl. Equitable | Objectified Body Consc. | -0.08 | -1.53 | 381 | 0.126 |
| Climate Incl. Equitable | Intuitive Eating Trust | 0.12 | 2.40 | 381 | 0.017 |
| Climate Incl. Equitable | Body Dissatisfaction | -0.01 | -0.27 | 377 | 0.784 |
| Climate Incl. Equitable | Anti-fat Dislike | -0.29 | -5.99 | 381 | < 0.001 |
| Climate Incl. Equitable | Anti-fat Fear | -0.25 | -4.94 | 381 | < 0.001 |
| Climate Incl. Equitable | Anti-fat Willpower | -0.24 | -4.92 | 381 | < 0.001 |
| Climate Incl. Equitable | Thin Ideal | -0.15 | -2.88 | 380 | 0.004 |
| Climate Incl. Equitable | Negative Body Talk Other | -0.32 | -6.50 | 381 | < 0.001 |
| Climate Incl. Equitable | Negative Body Talk Self | -0.11 | -2.07 | 381 | 0.039 |
| Climate Incl. Equitable | Job Task | 0.01 | 0.29 | 381 | 0.770 |
| Climate Incl. Equitable | Job Social | -0.05 | -0.91 | 381 | 0.365 |
| Climate Incl. Equitable | Job Physical | 0.05 | 0.99 | 381 | 0.323 |
| Climate Incl. Integration | Climate Incl. DM | 0.78 | 24.60 | 381 | < 0.001 |
| Climate Incl. Integration | Civility Climate | 0.59 | 14.19 | 381 | < 0.001 |
| Climate Incl. Integration | Leader-Member Exchange | 0.58 | 13.93 | 380 | < 0.001 |
| Climate Incl. Integration | Perceived Org. Support | 0.64 | 16.28 | 381 | < 0.001 |
| Climate Incl. Integration | Interpersonal Conflict | -0.41 | -8.72 | 381 | < 0.001 |
| Climate Incl. Integration | Tolerate Neg. Wgt. Talk | -0.39 | -8.19 | 381 | < 0.001 |
| Climate Incl. Integration | Pressure for Thinness | -0.15 | -2.88 | 381 | 0.004 |
| Climate Incl. Integration | Climate for Exercise | 0.24 | 4.83 | 381 | < 0.001 |
| Climate Incl. Integration | Wgt. Org. Support | 0.28 | 5.79 | 381 | < 0.001 |
| Climate Incl. Integration | Wgt. Social Support | 0.14 | 2.73 | 381 | 0.007 |
| Climate Incl. Integration | Wgt. Diet Norms | 0.16 | 3.08 | 381 | 0.002 |
| Climate Incl. Integration | Wgt. Belittle | -0.42 | -9.03 | 381 | < 0.001 |
| Climate Incl. Integration | Offer Exercise Program | 0.19 | 3.69 | 381 | < 0.001 |
| Climate Incl. Integration | Offer Weight Loss | 0.16 | 3.07 | 381 | 0.002 |
| Climate Incl. Integration | Offer Maintain Health | 0.18 | 3.64 | 380 | < 0.001 |
| Climate Incl. Integration | Comm. Exercise Program | 0.24 | 4.44 | 332 | < 0.001 |
| Climate Incl. Integration | Comm. Weight Loss | 0.27 | 5.04 | 327 | < 0.001 |
| Climate Incl. Integration | Comm. Maintain Health | 0.22 | 4.18 | 336 | < 0.001 |
| Climate Incl. Integration | Education Breakdown | -0.04 | -0.69 | 380 | 0.491 |
| Climate Incl. Integration | Weight Comparison | 0.10 | 1.94 | 377 | 0.054 |
| Climate Incl. Integration | Gender Breakdown | -0.03 | -0.53 | 381 | 0.595 |
| Climate Incl. Integration | Visible Disability | -0.13 | -2.48 | 378 | 0.014 |
| Climate Incl. Integration | Ethnic Name | -0.02 | -0.40 | 381 | 0.688 |
| Climate Incl. Integration | Body-Mass Index | -0.10 | -1.70 | 314 | 0.090 |
| Climate Incl. Integration | Accent | -0.06 | -1.12 | 381 | 0.261 |
| Climate Incl. Integration | Redefinition Reassociate | 0.17 | 3.34 | 381 | 0.001 |
| Climate Incl. Integration | Redefinition Regenerate | 0.16 | 3.17 | 381 | 0.002 |
| Climate Incl. Integration | Switch Deemphasize | 0.13 | 2.52 | 381 | 0.012 |
| Climate Incl. Integration | Switch Emphasize | 0.19 | 3.80 | 381 | < 0.001 |
| Climate Incl. Integration | Anxiety | -0.32 | -6.55 | 381 | < 0.001 |
| Climate Incl. Integration | Guilt | -0.24 | -4.79 | 380 | < 0.001 |
| Climate Incl. Integration | Shame | -0.23 | -4.61 | 381 | < 0.001 |
| Climate Incl. Integration | Depression | -0.33 | -6.83 | 381 | < 0.001 |
| Climate Incl. Integration | Core Self-Evaluations | 0.36 | 7.61 | 381 | < 0.001 |
| Climate Incl. Integration | Weight-Bias Internalization | -0.19 | -3.83 | 381 | < 0.001 |
| Climate Incl. Integration | Objectified Body Consc. | -0.06 | -1.23 | 381 | 0.220 |
| Climate Incl. Integration | Intuitive Eating Trust | 0.18 | 3.52 | 381 | < 0.001 |
| Climate Incl. Integration | Body Dissatisfaction | -0.10 | -1.88 | 377 | 0.061 |
| Climate Incl. Integration | Anti-fat Dislike | -0.45 | -9.94 | 381 | < 0.001 |
| Climate Incl. Integration | Anti-fat Fear | -0.39 | -8.30 | 381 | < 0.001 |
| Climate Incl. Integration | Anti-fat Willpower | -0.38 | -8.02 | 381 | < 0.001 |
| Climate Incl. Integration | Thin Ideal | -0.20 | -4.07 | 380 | < 0.001 |
| Climate Incl. Integration | Negative Body Talk Other | -0.46 | -10.03 | 381 | < 0.001 |
| Climate Incl. Integration | Negative Body Talk Self | -0.09 | -1.73 | 381 | 0.085 |
| Climate Incl. Integration | Job Task | -0.05 | -1.03 | 381 | 0.303 |
| Climate Incl. Integration | Job Social | -0.08 | -1.59 | 381 | 0.112 |
| Climate Incl. Integration | Job Physical | 0.01 | 0.22 | 381 | 0.829 |
| Climate Incl. DM | Civility Climate | 0.55 | 12.88 | 381 | < 0.001 |
| Climate Incl. DM | Leader-Member Exchange | 0.61 | 15.16 | 380 | < 0.001 |
| Climate Incl. DM | Perceived Org. Support | 0.65 | 16.67 | 381 | < 0.001 |
| Climate Incl. DM | Interpersonal Conflict | -0.39 | -8.37 | 381 | < 0.001 |
| Climate Incl. DM | Tolerate Neg. Wgt. Talk | -0.39 | -8.22 | 381 | < 0.001 |
| Climate Incl. DM | Pressure for Thinness | -0.06 | -1.19 | 381 | 0.236 |
| Climate Incl. DM | Climate for Exercise | 0.29 | 5.98 | 381 | < 0.001 |
| Climate Incl. DM | Wgt. Org. Support | 0.37 | 7.83 | 381 | < 0.001 |
| Climate Incl. DM | Wgt. Social Support | 0.23 | 4.66 | 381 | < 0.001 |
| Climate Incl. DM | Wgt. Diet Norms | 0.23 | 4.57 | 381 | < 0.001 |
| Climate Incl. DM | Wgt. Belittle | -0.33 | -6.93 | 381 | < 0.001 |
| Climate Incl. DM | Offer Exercise Program | 0.27 | 5.47 | 381 | < 0.001 |
| Climate Incl. DM | Offer Weight Loss | 0.23 | 4.53 | 381 | < 0.001 |
| Climate Incl. DM | Offer Maintain Health | 0.26 | 5.19 | 380 | < 0.001 |
| Climate Incl. DM | Comm. Exercise Program | 0.33 | 6.31 | 332 | < 0.001 |
| Climate Incl. DM | Comm. Weight Loss | 0.34 | 6.60 | 327 | < 0.001 |
| Climate Incl. DM | Comm. Maintain Health | 0.31 | 6.06 | 336 | < 0.001 |
| Climate Incl. DM | Education Breakdown | -0.08 | -1.49 | 380 | 0.136 |
| Climate Incl. DM | Weight Comparison | 0.13 | 2.60 | 377 | 0.010 |
| Climate Incl. DM | Gender Breakdown | -0.08 | -1.61 | 381 | 0.108 |
| Climate Incl. DM | Visible Disability | -0.08 | -1.49 | 378 | 0.138 |
| Climate Incl. DM | Ethnic Name | 0.03 | 0.51 | 381 | 0.607 |
| Climate Incl. DM | Body-Mass Index | -0.14 | -2.43 | 314 | 0.016 |
| Climate Incl. DM | Accent | -0.02 | -0.37 | 381 | 0.712 |
| Climate Incl. DM | Redefinition Reassociate | 0.18 | 3.59 | 381 | < 0.001 |
| Climate Incl. DM | Redefinition Regenerate | 0.12 | 2.34 | 381 | 0.020 |
| Climate Incl. DM | Switch Deemphasize | 0.10 | 1.97 | 381 | 0.049 |
| Climate Incl. DM | Switch Emphasize | 0.19 | 3.76 | 381 | < 0.001 |
| Climate Incl. DM | Anxiety | -0.29 | -5.98 | 381 | < 0.001 |
| Climate Incl. DM | Guilt | -0.19 | -3.82 | 380 | < 0.001 |
| Climate Incl. DM | Shame | -0.23 | -4.70 | 381 | < 0.001 |
| Climate Incl. DM | Depression | -0.32 | -6.58 | 381 | < 0.001 |
| Climate Incl. DM | Core Self-Evaluations | 0.36 | 7.60 | 381 | < 0.001 |
| Climate Incl. DM | Weight-Bias Internalization | -0.18 | -3.58 | 381 | < 0.001 |
| Climate Incl. DM | Objectified Body Consc. | -0.02 | -0.44 | 381 | 0.661 |
| Climate Incl. DM | Intuitive Eating Trust | 0.18 | 3.47 | 381 | 0.001 |
| Climate Incl. DM | Body Dissatisfaction | -0.08 | -1.47 | 377 | 0.143 |
| Climate Incl. DM | Anti-fat Dislike | -0.39 | -8.31 | 381 | < 0.001 |
| Climate Incl. DM | Anti-fat Fear | -0.34 | -7.08 | 381 | < 0.001 |
| Climate Incl. DM | Anti-fat Willpower | -0.33 | -6.88 | 381 | < 0.001 |
| Climate Incl. DM | Thin Ideal | -0.16 | -3.11 | 380 | 0.002 |
| Climate Incl. DM | Negative Body Talk Other | -0.38 | -8.11 | 381 | < 0.001 |
| Climate Incl. DM | Negative Body Talk Self | -0.11 | -2.08 | 381 | 0.038 |
| Climate Incl. DM | Job Task | -0.01 | -0.11 | 381 | 0.915 |
| Climate Incl. DM | Job Social | -0.03 | -0.53 | 381 | 0.598 |
| Climate Incl. DM | Job Physical | 0.00 | -0.05 | 381 | 0.961 |
| Civility Climate | Leader-Member Exchange | 0.60 | 14.60 | 380 | < 0.001 |
| Civility Climate | Perceived Org. Support | 0.69 | 18.70 | 381 | < 0.001 |
| Civility Climate | Interpersonal Conflict | -0.52 | -11.83 | 381 | < 0.001 |
| Civility Climate | Tolerate Neg. Wgt. Talk | -0.54 | -12.49 | 381 | < 0.001 |
| Civility Climate | Pressure for Thinness | -0.29 | -5.83 | 381 | < 0.001 |
| Civility Climate | Climate for Exercise | 0.19 | 3.80 | 381 | < 0.001 |
| Civility Climate | Wgt. Org. Support | 0.27 | 5.53 | 381 | < 0.001 |
| Civility Climate | Wgt. Social Support | 0.12 | 2.31 | 381 | 0.022 |
| Civility Climate | Wgt. Diet Norms | 0.16 | 3.18 | 381 | 0.002 |
| Civility Climate | Wgt. Belittle | -0.46 | -10.19 | 381 | < 0.001 |
| Civility Climate | Offer Exercise Program | 0.16 | 3.08 | 381 | 0.002 |
| Civility Climate | Offer Weight Loss | 0.12 | 2.46 | 381 | 0.015 |
| Civility Climate | Offer Maintain Health | 0.19 | 3.75 | 380 | < 0.001 |
| Civility Climate | Comm. Exercise Program | 0.24 | 4.54 | 332 | < 0.001 |
| Civility Climate | Comm. Weight Loss | 0.20 | 3.72 | 327 | < 0.001 |
| Civility Climate | Comm. Maintain Health | 0.20 | 3.72 | 336 | < 0.001 |
| Civility Climate | Education Breakdown | -0.07 | -1.39 | 380 | 0.165 |
| Civility Climate | Weight Comparison | 0.09 | 1.71 | 377 | 0.088 |
| Civility Climate | Gender Breakdown | 0.04 | 0.76 | 381 | 0.449 |
| Civility Climate | Visible Disability | -0.08 | -1.64 | 378 | 0.102 |
| Civility Climate | Ethnic Name | -0.06 | -1.14 | 381 | 0.254 |
| Civility Climate | Body-Mass Index | -0.13 | -2.29 | 314 | 0.023 |
| Civility Climate | Accent | -0.08 | -1.49 | 381 | 0.137 |
| Civility Climate | Redefinition Reassociate | 0.16 | 3.23 | 381 | 0.001 |
| Civility Climate | Redefinition Regenerate | 0.15 | 2.97 | 381 | 0.003 |
| Civility Climate | Switch Deemphasize | 0.11 | 2.11 | 381 | 0.035 |
| Civility Climate | Switch Emphasize | 0.16 | 3.25 | 381 | 0.001 |
| Civility Climate | Anxiety | -0.23 | -4.69 | 381 | < 0.001 |
| Civility Climate | Guilt | -0.24 | -4.73 | 380 | < 0.001 |
| Civility Climate | Shame | -0.24 | -4.92 | 381 | < 0.001 |
| Civility Climate | Depression | -0.28 | -5.78 | 381 | < 0.001 |
| Civility Climate | Core Self-Evaluations | 0.33 | 6.94 | 381 | < 0.001 |
| Civility Climate | Weight-Bias Internalization | -0.19 | -3.75 | 381 | < 0.001 |
| Civility Climate | Objectified Body Consc. | -0.05 | -0.90 | 381 | 0.368 |
| Civility Climate | Intuitive Eating Trust | 0.07 | 1.44 | 381 | 0.150 |
| Civility Climate | Body Dissatisfaction | -0.12 | -2.29 | 377 | 0.022 |
| Civility Climate | Anti-fat Dislike | -0.60 | -14.69 | 381 | < 0.001 |
| Civility Climate | Anti-fat Fear | -0.51 | -11.59 | 381 | < 0.001 |
| Civility Climate | Anti-fat Willpower | -0.46 | -10.05 | 381 | < 0.001 |
| Civility Climate | Thin Ideal | -0.26 | -5.15 | 380 | < 0.001 |
| Civility Climate | Negative Body Talk Other | -0.64 | -16.44 | 381 | < 0.001 |
| Civility Climate | Negative Body Talk Self | -0.19 | -3.72 | 381 | < 0.001 |
| Civility Climate | Job Task | 0.00 | 0.07 | 381 | 0.945 |
| Civility Climate | Job Social | -0.02 | -0.35 | 381 | 0.729 |
| Civility Climate | Job Physical | -0.06 | -1.11 | 381 | 0.266 |
| Leader-Member Exchange | Perceived Org. Support | 0.68 | 18.24 | 380 | < 0.001 |
| Leader-Member Exchange | Interpersonal Conflict | -0.44 | -9.49 | 380 | < 0.001 |
| Leader-Member Exchange | Tolerate Neg. Wgt. Talk | -0.41 | -8.78 | 380 | < 0.001 |
| Leader-Member Exchange | Pressure for Thinness | -0.09 | -1.86 | 380 | 0.064 |
| Leader-Member Exchange | Climate for Exercise | 0.27 | 5.44 | 380 | < 0.001 |
| Leader-Member Exchange | Wgt. Org. Support | 0.28 | 5.69 | 380 | < 0.001 |
| Leader-Member Exchange | Wgt. Social Support | 0.21 | 4.10 | 380 | < 0.001 |
| Leader-Member Exchange | Wgt. Diet Norms | 0.19 | 3.73 | 380 | < 0.001 |
| Leader-Member Exchange | Wgt. Belittle | -0.36 | -7.48 | 380 | < 0.001 |
| Leader-Member Exchange | Offer Exercise Program | 0.23 | 4.54 | 380 | < 0.001 |
| Leader-Member Exchange | Offer Weight Loss | 0.17 | 3.46 | 380 | 0.001 |
| Leader-Member Exchange | Offer Maintain Health | 0.23 | 4.58 | 379 | < 0.001 |
| Leader-Member Exchange | Comm. Exercise Program | 0.26 | 4.90 | 331 | < 0.001 |
| Leader-Member Exchange | Comm. Weight Loss | 0.24 | 4.55 | 326 | < 0.001 |
| Leader-Member Exchange | Comm. Maintain Health | 0.24 | 4.60 | 335 | < 0.001 |
| Leader-Member Exchange | Education Breakdown | -0.08 | -1.59 | 379 | 0.113 |
| Leader-Member Exchange | Weight Comparison | 0.14 | 2.66 | 376 | 0.008 |
| Leader-Member Exchange | Gender Breakdown | 0.02 | 0.37 | 380 | 0.713 |
| Leader-Member Exchange | Visible Disability | -0.10 | -1.87 | 377 | 0.062 |
| Leader-Member Exchange | Ethnic Name | -0.06 | -1.18 | 380 | 0.240 |
| Leader-Member Exchange | Body-Mass Index | -0.10 | -1.81 | 313 | 0.071 |
| Leader-Member Exchange | Accent | -0.04 | -0.71 | 380 | 0.480 |
| Leader-Member Exchange | Redefinition Reassociate | 0.17 | 3.45 | 380 | 0.001 |
| Leader-Member Exchange | Redefinition Regenerate | 0.12 | 2.42 | 380 | 0.016 |
| Leader-Member Exchange | Switch Deemphasize | 0.12 | 2.33 | 380 | 0.020 |
| Leader-Member Exchange | Switch Emphasize | 0.18 | 3.55 | 380 | < 0.001 |
| Leader-Member Exchange | Anxiety | -0.27 | -5.51 | 380 | < 0.001 |
| Leader-Member Exchange | Guilt | -0.24 | -4.74 | 379 | < 0.001 |
| Leader-Member Exchange | Shame | -0.25 | -4.99 | 380 | < 0.001 |
| Leader-Member Exchange | Depression | -0.31 | -6.46 | 380 | < 0.001 |
| Leader-Member Exchange | Core Self-Evaluations | 0.37 | 7.77 | 380 | < 0.001 |
| Leader-Member Exchange | Weight-Bias Internalization | -0.17 | -3.31 | 380 | 0.001 |
| Leader-Member Exchange | Objectified Body Consc. | -0.03 | -0.56 | 380 | 0.574 |
| Leader-Member Exchange | Intuitive Eating Trust | 0.09 | 1.80 | 380 | 0.073 |
| Leader-Member Exchange | Body Dissatisfaction | -0.10 | -1.86 | 376 | 0.063 |
| Leader-Member Exchange | Anti-fat Dislike | -0.42 | -8.96 | 380 | < 0.001 |
| Leader-Member Exchange | Anti-fat Fear | -0.34 | -7.16 | 380 | < 0.001 |
| Leader-Member Exchange | Anti-fat Willpower | -0.28 | -5.62 | 380 | < 0.001 |
| Leader-Member Exchange | Thin Ideal | -0.11 | -2.18 | 379 | 0.030 |
| Leader-Member Exchange | Negative Body Talk Other | -0.43 | -9.39 | 380 | < 0.001 |
| Leader-Member Exchange | Negative Body Talk Self | -0.06 | -1.16 | 380 | 0.247 |
| Leader-Member Exchange | Job Task | 0.04 | 0.71 | 380 | 0.478 |
| Leader-Member Exchange | Job Social | 0.06 | 1.14 | 380 | 0.256 |
| Leader-Member Exchange | Job Physical | -0.10 | -1.90 | 380 | 0.058 |
| Perceived Org. Support | Interpersonal Conflict | -0.49 | -11.09 | 381 | < 0.001 |
| Perceived Org. Support | Tolerate Neg. Wgt. Talk | -0.50 | -11.21 | 381 | < 0.001 |
| Perceived Org. Support | Pressure for Thinness | -0.15 | -2.93 | 381 | 0.004 |
| Perceived Org. Support | Climate for Exercise | 0.28 | 5.76 | 381 | < 0.001 |
| Perceived Org. Support | Wgt. Org. Support | 0.35 | 7.24 | 381 | < 0.001 |
| Perceived Org. Support | Wgt. Social Support | 0.17 | 3.38 | 381 | 0.001 |
| Perceived Org. Support | Wgt. Diet Norms | 0.22 | 4.38 | 381 | < 0.001 |
| Perceived Org. Support | Wgt. Belittle | -0.42 | -8.95 | 381 | < 0.001 |
| Perceived Org. Support | Offer Exercise Program | 0.22 | 4.47 | 381 | < 0.001 |
| Perceived Org. Support | Offer Weight Loss | 0.18 | 3.48 | 381 | 0.001 |
| Perceived Org. Support | Offer Maintain Health | 0.27 | 5.53 | 380 | < 0.001 |
| Perceived Org. Support | Comm. Exercise Program | 0.27 | 5.02 | 332 | < 0.001 |
| Perceived Org. Support | Comm. Weight Loss | 0.27 | 4.99 | 327 | < 0.001 |
| Perceived Org. Support | Comm. Maintain Health | 0.29 | 5.53 | 336 | < 0.001 |
| Perceived Org. Support | Education Breakdown | -0.04 | -0.82 | 380 | 0.412 |
| Perceived Org. Support | Weight Comparison | 0.18 | 3.49 | 377 | 0.001 |
| Perceived Org. Support | Gender Breakdown | 0.02 | 0.44 | 381 | 0.661 |
| Perceived Org. Support | Visible Disability | -0.07 | -1.31 | 378 | 0.192 |
| Perceived Org. Support | Ethnic Name | -0.07 | -1.35 | 381 | 0.178 |
| Perceived Org. Support | Body-Mass Index | -0.15 | -2.64 | 314 | 0.009 |
| Perceived Org. Support | Accent | -0.07 | -1.41 | 381 | 0.161 |
| Perceived Org. Support | Redefinition Reassociate | 0.19 | 3.75 | 381 | < 0.001 |
| Perceived Org. Support | Redefinition Regenerate | 0.14 | 2.80 | 381 | 0.005 |
| Perceived Org. Support | Switch Deemphasize | 0.14 | 2.81 | 381 | 0.005 |
| Perceived Org. Support | Switch Emphasize | 0.22 | 4.46 | 381 | < 0.001 |
| Perceived Org. Support | Anxiety | -0.25 | -4.99 | 381 | < 0.001 |
| Perceived Org. Support | Guilt | -0.21 | -4.20 | 380 | < 0.001 |
| Perceived Org. Support | Shame | -0.26 | -5.16 | 381 | < 0.001 |
| Perceived Org. Support | Depression | -0.28 | -5.78 | 381 | < 0.001 |
| Perceived Org. Support | Core Self-Evaluations | 0.37 | 7.87 | 381 | < 0.001 |
| Perceived Org. Support | Weight-Bias Internalization | -0.17 | -3.29 | 381 | 0.001 |
| Perceived Org. Support | Objectified Body Consc. | -0.01 | -0.22 | 381 | 0.824 |
| Perceived Org. Support | Intuitive Eating Trust | 0.11 | 2.23 | 381 | 0.026 |
| Perceived Org. Support | Body Dissatisfaction | -0.13 | -2.47 | 377 | 0.014 |
| Perceived Org. Support | Anti-fat Dislike | -0.50 | -11.27 | 381 | < 0.001 |
| Perceived Org. Support | Anti-fat Fear | -0.40 | -8.56 | 381 | < 0.001 |
| Perceived Org. Support | Anti-fat Willpower | -0.36 | -7.54 | 381 | < 0.001 |
| Perceived Org. Support | Thin Ideal | -0.17 | -3.33 | 380 | 0.001 |
| Perceived Org. Support | Negative Body Talk Other | -0.52 | -11.98 | 381 | < 0.001 |
| Perceived Org. Support | Negative Body Talk Self | -0.11 | -2.08 | 381 | 0.038 |
| Perceived Org. Support | Job Task | 0.00 | -0.02 | 381 | 0.987 |
| Perceived Org. Support | Job Social | 0.00 | 0.02 | 381 | 0.983 |
| Perceived Org. Support | Job Physical | -0.06 | -1.17 | 381 | 0.244 |
| Interpersonal Conflict | Tolerate Neg. Wgt. Talk | 0.45 | 9.78 | 381 | < 0.001 |
| Interpersonal Conflict | Pressure for Thinness | 0.30 | 6.06 | 381 | < 0.001 |
| Interpersonal Conflict | Climate for Exercise | -0.02 | -0.43 | 381 | 0.670 |
| Interpersonal Conflict | Wgt. Org. Support | -0.07 | -1.34 | 381 | 0.180 |
| Interpersonal Conflict | Wgt. Social Support | 0.01 | 0.22 | 381 | 0.829 |
| Interpersonal Conflict | Wgt. Diet Norms | 0.00 | -0.02 | 381 | 0.982 |
| Interpersonal Conflict | Wgt. Belittle | 0.39 | 8.32 | 381 | < 0.001 |
| Interpersonal Conflict | Offer Exercise Program | -0.01 | -0.25 | 381 | 0.804 |
| Interpersonal Conflict | Offer Weight Loss | 0.00 | -0.09 | 381 | 0.929 |
| Interpersonal Conflict | Offer Maintain Health | -0.05 | -1.00 | 380 | 0.317 |
| Interpersonal Conflict | Comm. Exercise Program | -0.06 | -1.02 | 332 | 0.310 |
| Interpersonal Conflict | Comm. Weight Loss | -0.03 | -0.55 | 327 | 0.584 |
| Interpersonal Conflict | Comm. Maintain Health | -0.06 | -1.08 | 336 | 0.283 |
| Interpersonal Conflict | Education Breakdown | -0.01 | -0.27 | 380 | 0.789 |
| Interpersonal Conflict | Weight Comparison | -0.02 | -0.46 | 377 | 0.648 |
| Interpersonal Conflict | Gender Breakdown | -0.06 | -1.10 | 381 | 0.272 |
| Interpersonal Conflict | Visible Disability | 0.18 | 3.65 | 378 | < 0.001 |
| Interpersonal Conflict | Ethnic Name | 0.10 | 1.92 | 381 | 0.056 |
| Interpersonal Conflict | Body-Mass Index | 0.00 | -0.05 | 314 | 0.963 |
| Interpersonal Conflict | Accent | 0.09 | 1.81 | 381 | 0.071 |
| Interpersonal Conflict | Redefinition Reassociate | -0.02 | -0.32 | 381 | 0.751 |
| Interpersonal Conflict | Redefinition Regenerate | -0.02 | -0.35 | 381 | 0.727 |
| Interpersonal Conflict | Switch Deemphasize | -0.02 | -0.32 | 381 | 0.748 |
| Interpersonal Conflict | Switch Emphasize | -0.08 | -1.49 | 381 | 0.136 |
| Interpersonal Conflict | Anxiety | 0.26 | 5.16 | 381 | < 0.001 |
| Interpersonal Conflict | Guilt | 0.25 | 5.09 | 380 | < 0.001 |
| Interpersonal Conflict | Shame | 0.19 | 3.82 | 381 | < 0.001 |
| Interpersonal Conflict | Depression | 0.22 | 4.42 | 381 | < 0.001 |
| Interpersonal Conflict | Core Self-Evaluations | -0.27 | -5.42 | 381 | < 0.001 |
| Interpersonal Conflict | Weight-Bias Internalization | 0.13 | 2.62 | 381 | 0.009 |
| Interpersonal Conflict | Objectified Body Consc. | 0.10 | 1.90 | 381 | 0.058 |
| Interpersonal Conflict | Intuitive Eating Trust | -0.03 | -0.58 | 381 | 0.564 |
| Interpersonal Conflict | Body Dissatisfaction | 0.05 | 0.89 | 377 | 0.376 |
| Interpersonal Conflict | Anti-fat Dislike | 0.55 | 13.00 | 381 | < 0.001 |
| Interpersonal Conflict | Anti-fat Fear | 0.47 | 10.53 | 381 | < 0.001 |
| Interpersonal Conflict | Anti-fat Willpower | 0.36 | 7.46 | 381 | < 0.001 |
| Interpersonal Conflict | Thin Ideal | 0.29 | 5.94 | 380 | < 0.001 |
| Interpersonal Conflict | Negative Body Talk Other | 0.53 | 12.31 | 381 | < 0.001 |
| Interpersonal Conflict | Negative Body Talk Self | 0.23 | 4.57 | 381 | < 0.001 |
| Interpersonal Conflict | Job Task | -0.02 | -0.34 | 381 | 0.737 |
| Interpersonal Conflict | Job Social | 0.03 | 0.63 | 381 | 0.526 |
| Interpersonal Conflict | Job Physical | 0.07 | 1.33 | 381 | 0.186 |
| Tolerate Neg. Wgt. Talk | Pressure for Thinness | 0.46 | 10.15 | 381 | < 0.001 |
| Tolerate Neg. Wgt. Talk | Climate for Exercise | -0.02 | -0.30 | 381 | 0.766 |
| Tolerate Neg. Wgt. Talk | Wgt. Org. Support | -0.09 | -1.83 | 381 | 0.068 |
| Tolerate Neg. Wgt. Talk | Wgt. Social Support | 0.04 | 0.85 | 381 | 0.395 |
| Tolerate Neg. Wgt. Talk | Wgt. Diet Norms | -0.01 | -0.21 | 381 | 0.837 |
| Tolerate Neg. Wgt. Talk | Wgt. Belittle | 0.41 | 8.87 | 381 | < 0.001 |
| Tolerate Neg. Wgt. Talk | Offer Exercise Program | -0.02 | -0.43 | 381 | 0.668 |
| Tolerate Neg. Wgt. Talk | Offer Weight Loss | -0.01 | -0.22 | 381 | 0.827 |
| Tolerate Neg. Wgt. Talk | Offer Maintain Health | -0.07 | -1.37 | 380 | 0.170 |
| Tolerate Neg. Wgt. Talk | Comm. Exercise Program | -0.06 | -1.15 | 332 | 0.249 |
| Tolerate Neg. Wgt. Talk | Comm. Weight Loss | -0.06 | -1.09 | 327 | 0.279 |
| Tolerate Neg. Wgt. Talk | Comm. Maintain Health | -0.09 | -1.67 | 336 | 0.096 |
| Tolerate Neg. Wgt. Talk | Education Breakdown | 0.01 | 0.16 | 380 | 0.874 |
| Tolerate Neg. Wgt. Talk | Weight Comparison | -0.15 | -2.99 | 377 | 0.003 |
| Tolerate Neg. Wgt. Talk | Gender Breakdown | 0.00 | -0.02 | 381 | 0.988 |
| Tolerate Neg. Wgt. Talk | Visible Disability | 0.05 | 0.89 | 378 | 0.374 |
| Tolerate Neg. Wgt. Talk | Ethnic Name | 0.08 | 1.63 | 381 | 0.104 |
| Tolerate Neg. Wgt. Talk | Body-Mass Index | 0.10 | 1.74 | 314 | 0.084 |
| Tolerate Neg. Wgt. Talk | Accent | 0.10 | 1.95 | 381 | 0.052 |
| Tolerate Neg. Wgt. Talk | Redefinition Reassociate | -0.18 | -3.53 | 381 | < 0.001 |
| Tolerate Neg. Wgt. Talk | Redefinition Regenerate | -0.14 | -2.85 | 381 | 0.005 |
| Tolerate Neg. Wgt. Talk | Switch Deemphasize | -0.06 | -1.22 | 381 | 0.221 |
| Tolerate Neg. Wgt. Talk | Switch Emphasize | -0.16 | -3.09 | 381 | 0.002 |
| Tolerate Neg. Wgt. Talk | Anxiety | 0.28 | 5.66 | 381 | < 0.001 |
| Tolerate Neg. Wgt. Talk | Guilt | 0.22 | 4.39 | 380 | < 0.001 |
| Tolerate Neg. Wgt. Talk | Shame | 0.38 | 8.10 | 381 | < 0.001 |
| Tolerate Neg. Wgt. Talk | Depression | 0.31 | 6.37 | 381 | < 0.001 |
| Tolerate Neg. Wgt. Talk | Core Self-Evaluations | -0.35 | -7.37 | 381 | < 0.001 |
| Tolerate Neg. Wgt. Talk | Weight-Bias Internalization | 0.32 | 6.69 | 381 | < 0.001 |
| Tolerate Neg. Wgt. Talk | Objectified Body Consc. | 0.15 | 3.05 | 381 | 0.002 |
| Tolerate Neg. Wgt. Talk | Intuitive Eating Trust | -0.16 | -3.06 | 381 | 0.002 |
| Tolerate Neg. Wgt. Talk | Body Dissatisfaction | 0.20 | 3.87 | 377 | < 0.001 |
| Tolerate Neg. Wgt. Talk | Anti-fat Dislike | 0.62 | 15.40 | 381 | < 0.001 |
| Tolerate Neg. Wgt. Talk | Anti-fat Fear | 0.61 | 14.97 | 381 | < 0.001 |
| Tolerate Neg. Wgt. Talk | Anti-fat Willpower | 0.57 | 13.52 | 381 | < 0.001 |
| Tolerate Neg. Wgt. Talk | Thin Ideal | 0.43 | 9.34 | 380 | < 0.001 |
| Tolerate Neg. Wgt. Talk | Negative Body Talk Other | 0.60 | 14.71 | 381 | < 0.001 |
| Tolerate Neg. Wgt. Talk | Negative Body Talk Self | 0.25 | 5.05 | 381 | < 0.001 |
| Tolerate Neg. Wgt. Talk | Job Task | 0.00 | 0.09 | 381 | 0.925 |
| Tolerate Neg. Wgt. Talk | Job Social | 0.03 | 0.53 | 381 | 0.596 |
| Tolerate Neg. Wgt. Talk | Job Physical | 0.00 | 0.07 | 381 | 0.942 |
| Pressure for Thinness | Climate for Exercise | 0.42 | 9.09 | 381 | < 0.001 |
| Pressure for Thinness | Wgt. Org. Support | 0.28 | 5.67 | 381 | < 0.001 |
| Pressure for Thinness | Wgt. Social Support | 0.38 | 8.11 | 381 | < 0.001 |
| Pressure for Thinness | Wgt. Diet Norms | 0.27 | 5.55 | 381 | < 0.001 |
| Pressure for Thinness | Wgt. Belittle | 0.24 | 4.78 | 381 | < 0.001 |
| Pressure for Thinness | Offer Exercise Program | 0.21 | 4.22 | 381 | < 0.001 |
| Pressure for Thinness | Offer Weight Loss | 0.21 | 4.15 | 381 | < 0.001 |
| Pressure for Thinness | Offer Maintain Health | 0.20 | 4.05 | 380 | < 0.001 |
| Pressure for Thinness | Comm. Exercise Program | 0.20 | 3.77 | 332 | < 0.001 |
| Pressure for Thinness | Comm. Weight Loss | 0.25 | 4.72 | 327 | < 0.001 |
| Pressure for Thinness | Comm. Maintain Health | 0.18 | 3.28 | 336 | 0.001 |
| Pressure for Thinness | Education Breakdown | -0.05 | -0.95 | 380 | 0.344 |
| Pressure for Thinness | Weight Comparison | -0.07 | -1.43 | 377 | 0.153 |
| Pressure for Thinness | Gender Breakdown | 0.03 | 0.64 | 381 | 0.520 |
| Pressure for Thinness | Visible Disability | 0.01 | 0.25 | 378 | 0.805 |
| Pressure for Thinness | Ethnic Name | 0.02 | 0.44 | 381 | 0.661 |
| Pressure for Thinness | Body-Mass Index | 0.04 | 0.66 | 314 | 0.510 |
| Pressure for Thinness | Accent | 0.11 | 2.24 | 381 | 0.026 |
| Pressure for Thinness | Redefinition Reassociate | -0.02 | -0.33 | 381 | 0.740 |
| Pressure for Thinness | Redefinition Regenerate | -0.02 | -0.33 | 381 | 0.743 |
| Pressure for Thinness | Switch Deemphasize | -0.03 | -0.68 | 381 | 0.496 |
| Pressure for Thinness | Switch Emphasize | -0.06 | -1.14 | 381 | 0.254 |
| Pressure for Thinness | Anxiety | 0.08 | 1.48 | 381 | 0.141 |
| Pressure for Thinness | Guilt | 0.11 | 2.18 | 380 | 0.030 |
| Pressure for Thinness | Shame | 0.22 | 4.38 | 381 | < 0.001 |
| Pressure for Thinness | Depression | 0.07 | 1.46 | 381 | 0.144 |
| Pressure for Thinness | Core Self-Evaluations | -0.11 | -2.11 | 381 | 0.036 |
| Pressure for Thinness | Weight-Bias Internalization | 0.19 | 3.80 | 381 | < 0.001 |
| Pressure for Thinness | Objectified Body Consc. | 0.14 | 2.85 | 381 | 0.005 |
| Pressure for Thinness | Intuitive Eating Trust | -0.09 | -1.81 | 381 | 0.071 |
| Pressure for Thinness | Body Dissatisfaction | 0.09 | 1.78 | 377 | 0.076 |
| Pressure for Thinness | Anti-fat Dislike | 0.49 | 10.86 | 381 | < 0.001 |
| Pressure for Thinness | Anti-fat Fear | 0.55 | 12.99 | 381 | < 0.001 |
| Pressure for Thinness | Anti-fat Willpower | 0.49 | 10.89 | 381 | < 0.001 |
| Pressure for Thinness | Thin Ideal | 0.47 | 10.51 | 380 | < 0.001 |
| Pressure for Thinness | Negative Body Talk Other | 0.53 | 12.05 | 381 | < 0.001 |
| Pressure for Thinness | Negative Body Talk Self | 0.37 | 7.87 | 381 | < 0.001 |
| Pressure for Thinness | Job Task | 0.07 | 1.46 | 381 | 0.144 |
| Pressure for Thinness | Job Social | 0.12 | 2.44 | 381 | 0.015 |
| Pressure for Thinness | Job Physical | -0.01 | -0.11 | 381 | 0.916 |
| Climate for Exercise | Wgt. Org. Support | 0.74 | 21.62 | 381 | < 0.001 |
| Climate for Exercise | Wgt. Social Support | 0.73 | 20.83 | 381 | < 0.001 |
| Climate for Exercise | Wgt. Diet Norms | 0.65 | 16.72 | 381 | < 0.001 |
| Climate for Exercise | Wgt. Belittle | -0.12 | -2.41 | 381 | 0.016 |
| Climate for Exercise | Offer Exercise Program | 0.61 | 15.15 | 381 | < 0.001 |
| Climate for Exercise | Offer Weight Loss | 0.58 | 13.84 | 381 | < 0.001 |
| Climate for Exercise | Offer Maintain Health | 0.58 | 13.92 | 380 | < 0.001 |
| Climate for Exercise | Comm. Exercise Program | 0.64 | 15.21 | 332 | < 0.001 |
| Climate for Exercise | Comm. Weight Loss | 0.67 | 16.30 | 327 | < 0.001 |
| Climate for Exercise | Comm. Maintain Health | 0.59 | 13.40 | 336 | < 0.001 |
| Climate for Exercise | Education Breakdown | -0.17 | -3.31 | 380 | 0.001 |
| Climate for Exercise | Weight Comparison | 0.01 | 0.22 | 377 | 0.829 |
| Climate for Exercise | Gender Breakdown | -0.01 | -0.20 | 381 | 0.845 |
| Climate for Exercise | Visible Disability | 0.05 | 1.03 | 378 | 0.305 |
| Climate for Exercise | Ethnic Name | 0.05 | 1.00 | 381 | 0.318 |
| Climate for Exercise | Body-Mass Index | -0.08 | -1.44 | 314 | 0.152 |
| Climate for Exercise | Accent | 0.08 | 1.61 | 381 | 0.108 |
| Climate for Exercise | Redefinition Reassociate | 0.21 | 4.24 | 381 | < 0.001 |
| Climate for Exercise | Redefinition Regenerate | 0.22 | 4.35 | 381 | < 0.001 |
| Climate for Exercise | Switch Deemphasize | 0.11 | 2.20 | 381 | 0.029 |
| Climate for Exercise | Switch Emphasize | 0.09 | 1.79 | 381 | 0.074 |
| Climate for Exercise | Anxiety | -0.07 | -1.32 | 381 | 0.188 |
| Climate for Exercise | Guilt | -0.07 | -1.42 | 380 | 0.158 |
| Climate for Exercise | Shame | -0.05 | -1.02 | 381 | 0.309 |
| Climate for Exercise | Depression | -0.12 | -2.27 | 381 | 0.023 |
| Climate for Exercise | Core Self-Evaluations | 0.15 | 3.05 | 381 | 0.002 |
| Climate for Exercise | Weight-Bias Internalization | -0.08 | -1.64 | 381 | 0.103 |
| Climate for Exercise | Objectified Body Consc. | 0.07 | 1.37 | 381 | 0.170 |
| Climate for Exercise | Intuitive Eating Trust | 0.14 | 2.81 | 381 | 0.005 |
| Climate for Exercise | Body Dissatisfaction | -0.08 | -1.50 | 377 | 0.134 |
| Climate for Exercise | Anti-fat Dislike | 0.03 | 0.55 | 381 | 0.582 |
| Climate for Exercise | Anti-fat Fear | 0.06 | 1.19 | 381 | 0.233 |
| Climate for Exercise | Anti-fat Willpower | 0.01 | 0.22 | 381 | 0.825 |
| Climate for Exercise | Thin Ideal | 0.07 | 1.33 | 380 | 0.184 |
| Climate for Exercise | Negative Body Talk Other | -0.02 | -0.47 | 381 | 0.640 |
| Climate for Exercise | Negative Body Talk Self | 0.05 | 0.92 | 381 | 0.356 |
| Climate for Exercise | Job Task | 0.08 | 1.63 | 381 | 0.105 |
| Climate for Exercise | Job Social | 0.11 | 2.18 | 381 | 0.030 |
| Climate for Exercise | Job Physical | 0.06 | 1.24 | 381 | 0.217 |
| Wgt. Org. Support | Wgt. Social Support | 0.65 | 16.86 | 381 | < 0.001 |
| Wgt. Org. Support | Wgt. Diet Norms | 0.63 | 15.98 | 381 | < 0.001 |
| Wgt. Org. Support | Wgt. Belittle | -0.16 | -3.25 | 381 | 0.001 |
| Wgt. Org. Support | Offer Exercise Program | 0.57 | 13.68 | 381 | < 0.001 |
| Wgt. Org. Support | Offer Weight Loss | 0.57 | 13.69 | 381 | < 0.001 |
| Wgt. Org. Support | Offer Maintain Health | 0.56 | 13.03 | 380 | < 0.001 |
| Wgt. Org. Support | Comm. Exercise Program | 0.65 | 15.65 | 332 | < 0.001 |
| Wgt. Org. Support | Comm. Weight Loss | 0.67 | 16.14 | 327 | < 0.001 |
| Wgt. Org. Support | Comm. Maintain Health | 0.60 | 13.89 | 336 | < 0.001 |
| Wgt. Org. Support | Education Breakdown | -0.17 | -3.35 | 380 | 0.001 |
| Wgt. Org. Support | Weight Comparison | 0.09 | 1.66 | 377 | 0.098 |
| Wgt. Org. Support | Gender Breakdown | 0.00 | 0.02 | 381 | 0.981 |
| Wgt. Org. Support | Visible Disability | 0.04 | 0.76 | 378 | 0.450 |
| Wgt. Org. Support | Ethnic Name | 0.03 | 0.61 | 381 | 0.545 |
| Wgt. Org. Support | Body-Mass Index | -0.13 | -2.30 | 314 | 0.022 |
| Wgt. Org. Support | Accent | 0.04 | 0.72 | 381 | 0.471 |
| Wgt. Org. Support | Redefinition Reassociate | 0.17 | 3.45 | 381 | 0.001 |
| Wgt. Org. Support | Redefinition Regenerate | 0.18 | 3.49 | 381 | 0.001 |
| Wgt. Org. Support | Switch Deemphasize | 0.04 | 0.85 | 381 | 0.396 |
| Wgt. Org. Support | Switch Emphasize | 0.03 | 0.62 | 381 | 0.538 |
| Wgt. Org. Support | Anxiety | -0.05 | -1.02 | 381 | 0.309 |
| Wgt. Org. Support | Guilt | -0.01 | -0.21 | 380 | 0.834 |
| Wgt. Org. Support | Shame | -0.07 | -1.29 | 381 | 0.199 |
| Wgt. Org. Support | Depression | -0.09 | -1.69 | 381 | 0.092 |
| Wgt. Org. Support | Core Self-Evaluations | 0.17 | 3.38 | 381 | 0.001 |
| Wgt. Org. Support | Weight-Bias Internalization | -0.08 | -1.61 | 381 | 0.108 |
| Wgt. Org. Support | Objectified Body Consc. | 0.07 | 1.46 | 381 | 0.145 |
| Wgt. Org. Support | Intuitive Eating Trust | 0.11 | 2.19 | 381 | 0.029 |
| Wgt. Org. Support | Body Dissatisfaction | -0.08 | -1.51 | 377 | 0.131 |
| Wgt. Org. Support | Anti-fat Dislike | -0.06 | -1.21 | 381 | 0.228 |
| Wgt. Org. Support | Anti-fat Fear | -0.02 | -0.47 | 381 | 0.638 |
| Wgt. Org. Support | Anti-fat Willpower | -0.09 | -1.69 | 381 | 0.092 |
| Wgt. Org. Support | Thin Ideal | -0.03 | -0.59 | 380 | 0.555 |
| Wgt. Org. Support | Negative Body Talk Other | -0.09 | -1.74 | 381 | 0.083 |
| Wgt. Org. Support | Negative Body Talk Self | -0.04 | -0.73 | 381 | 0.469 |
| Wgt. Org. Support | Job Task | 0.12 | 2.38 | 381 | 0.018 |
| Wgt. Org. Support | Job Social | 0.05 | 0.90 | 381 | 0.368 |
| Wgt. Org. Support | Job Physical | 0.06 | 1.14 | 381 | 0.255 |
| Wgt. Social Support | Wgt. Diet Norms | 0.67 | 17.38 | 381 | < 0.001 |
| Wgt. Social Support | Wgt. Belittle | -0.05 | -1.07 | 381 | 0.284 |
| Wgt. Social Support | Offer Exercise Program | 0.47 | 10.27 | 381 | < 0.001 |
| Wgt. Social Support | Offer Weight Loss | 0.43 | 9.37 | 381 | < 0.001 |
| Wgt. Social Support | Offer Maintain Health | 0.43 | 9.28 | 380 | < 0.001 |
| Wgt. Social Support | Comm. Exercise Program | 0.45 | 9.20 | 332 | < 0.001 |
| Wgt. Social Support | Comm. Weight Loss | 0.48 | 9.79 | 327 | < 0.001 |
| Wgt. Social Support | Comm. Maintain Health | 0.42 | 8.43 | 336 | < 0.001 |
| Wgt. Social Support | Education Breakdown | -0.16 | -3.25 | 380 | 0.001 |
| Wgt. Social Support | Weight Comparison | 0.01 | 0.18 | 377 | 0.857 |
| Wgt. Social Support | Gender Breakdown | -0.02 | -0.38 | 381 | 0.705 |
| Wgt. Social Support | Visible Disability | 0.00 | -0.05 | 378 | 0.961 |
| Wgt. Social Support | Ethnic Name | -0.03 | -0.60 | 381 | 0.549 |
| Wgt. Social Support | Body-Mass Index | -0.08 | -1.35 | 314 | 0.179 |
| Wgt. Social Support | Accent | 0.05 | 0.92 | 381 | 0.359 |
| Wgt. Social Support | Redefinition Reassociate | 0.11 | 2.07 | 381 | 0.040 |
| Wgt. Social Support | Redefinition Regenerate | 0.10 | 1.96 | 381 | 0.051 |
| Wgt. Social Support | Switch Deemphasize | 0.06 | 1.25 | 381 | 0.210 |
| Wgt. Social Support | Switch Emphasize | 0.04 | 0.75 | 381 | 0.457 |
| Wgt. Social Support | Anxiety | -0.03 | -0.66 | 381 | 0.512 |
| Wgt. Social Support | Guilt | 0.01 | 0.25 | 380 | 0.805 |
| Wgt. Social Support | Shame | 0.04 | 0.72 | 381 | 0.469 |
| Wgt. Social Support | Depression | -0.04 | -0.72 | 381 | 0.474 |
| Wgt. Social Support | Core Self-Evaluations | 0.06 | 1.11 | 381 | 0.268 |
| Wgt. Social Support | Weight-Bias Internalization | 0.01 | 0.24 | 381 | 0.809 |
| Wgt. Social Support | Objectified Body Consc. | 0.10 | 1.99 | 381 | 0.047 |
| Wgt. Social Support | Intuitive Eating Trust | 0.05 | 1.02 | 381 | 0.309 |
| Wgt. Social Support | Body Dissatisfaction | -0.04 | -0.77 | 377 | 0.444 |
| Wgt. Social Support | Anti-fat Dislike | 0.04 | 0.87 | 381 | 0.385 |
| Wgt. Social Support | Anti-fat Fear | 0.10 | 1.99 | 381 | 0.047 |
| Wgt. Social Support | Anti-fat Willpower | 0.08 | 1.53 | 381 | 0.127 |
| Wgt. Social Support | Thin Ideal | 0.07 | 1.37 | 380 | 0.173 |
| Wgt. Social Support | Negative Body Talk Other | 0.01 | 0.26 | 381 | 0.794 |
| Wgt. Social Support | Negative Body Talk Self | 0.03 | 0.64 | 381 | 0.521 |
| Wgt. Social Support | Job Task | 0.07 | 1.42 | 381 | 0.158 |
| Wgt. Social Support | Job Social | 0.09 | 1.76 | 381 | 0.078 |
| Wgt. Social Support | Job Physical | 0.01 | 0.14 | 381 | 0.886 |
| Wgt. Diet Norms | Wgt. Belittle | -0.16 | -3.19 | 381 | 0.002 |
| Wgt. Diet Norms | Offer Exercise Program | 0.41 | 8.79 | 381 | < 0.001 |
| Wgt. Diet Norms | Offer Weight Loss | 0.42 | 9.12 | 381 | < 0.001 |
| Wgt. Diet Norms | Offer Maintain Health | 0.38 | 8.12 | 380 | < 0.001 |
| Wgt. Diet Norms | Comm. Exercise Program | 0.48 | 9.97 | 332 | < 0.001 |
| Wgt. Diet Norms | Comm. Weight Loss | 0.47 | 9.76 | 327 | < 0.001 |
| Wgt. Diet Norms | Comm. Maintain Health | 0.43 | 8.66 | 336 | < 0.001 |
| Wgt. Diet Norms | Education Breakdown | -0.20 | -3.94 | 380 | < 0.001 |
| Wgt. Diet Norms | Weight Comparison | 0.09 | 1.81 | 377 | 0.072 |
| Wgt. Diet Norms | Gender Breakdown | -0.03 | -0.58 | 381 | 0.559 |
| Wgt. Diet Norms | Visible Disability | 0.03 | 0.60 | 378 | 0.550 |
| Wgt. Diet Norms | Ethnic Name | 0.00 | 0.07 | 381 | 0.942 |
| Wgt. Diet Norms | Body-Mass Index | -0.11 | -1.94 | 314 | 0.053 |
| Wgt. Diet Norms | Accent | 0.07 | 1.31 | 381 | 0.191 |
| Wgt. Diet Norms | Redefinition Reassociate | 0.17 | 3.44 | 381 | 0.001 |
| Wgt. Diet Norms | Redefinition Regenerate | 0.14 | 2.66 | 381 | 0.008 |
| Wgt. Diet Norms | Switch Deemphasize | 0.02 | 0.45 | 381 | 0.650 |
| Wgt. Diet Norms | Switch Emphasize | 0.02 | 0.38 | 381 | 0.704 |
| Wgt. Diet Norms | Anxiety | -0.03 | -0.58 | 381 | 0.559 |
| Wgt. Diet Norms | Guilt | -0.04 | -0.75 | 380 | 0.454 |
| Wgt. Diet Norms | Shame | -0.10 | -1.98 | 381 | 0.048 |
| Wgt. Diet Norms | Depression | -0.08 | -1.64 | 381 | 0.101 |
| Wgt. Diet Norms | Core Self-Evaluations | 0.15 | 2.97 | 381 | 0.003 |
| Wgt. Diet Norms | Weight-Bias Internalization | -0.11 | -2.14 | 381 | 0.033 |
| Wgt. Diet Norms | Objectified Body Consc. | 0.04 | 0.84 | 381 | 0.400 |
| Wgt. Diet Norms | Intuitive Eating Trust | 0.15 | 2.94 | 381 | 0.004 |
| Wgt. Diet Norms | Body Dissatisfaction | -0.04 | -0.80 | 377 | 0.425 |
| Wgt. Diet Norms | Anti-fat Dislike | 0.01 | 0.21 | 381 | 0.832 |
| Wgt. Diet Norms | Anti-fat Fear | -0.03 | -0.59 | 381 | 0.555 |
| Wgt. Diet Norms | Anti-fat Willpower | -0.07 | -1.46 | 381 | 0.144 |
| Wgt. Diet Norms | Thin Ideal | -0.01 | -0.27 | 380 | 0.784 |
| Wgt. Diet Norms | Negative Body Talk Other | -0.03 | -0.67 | 381 | 0.501 |
| Wgt. Diet Norms | Negative Body Talk Self | -0.11 | -2.22 | 381 | 0.027 |
| Wgt. Diet Norms | Job Task | 0.05 | 0.91 | 381 | 0.361 |
| Wgt. Diet Norms | Job Social | 0.04 | 0.71 | 381 | 0.475 |
| Wgt. Diet Norms | Job Physical | -0.04 | -0.76 | 381 | 0.448 |
| Wgt. Belittle | Offer Exercise Program | -0.10 | -2.02 | 381 | 0.045 |
| Wgt. Belittle | Offer Weight Loss | -0.09 | -1.79 | 381 | 0.074 |
| Wgt. Belittle | Offer Maintain Health | -0.14 | -2.80 | 380 | 0.005 |
| Wgt. Belittle | Comm. Exercise Program | -0.10 | -1.86 | 332 | 0.063 |
| Wgt. Belittle | Comm. Weight Loss | -0.11 | -1.98 | 327 | 0.048 |
| Wgt. Belittle | Comm. Maintain Health | -0.13 | -2.43 | 336 | 0.015 |
| Wgt. Belittle | Education Breakdown | -0.02 | -0.33 | 380 | 0.742 |
| Wgt. Belittle | Weight Comparison | 0.01 | 0.27 | 377 | 0.785 |
| Wgt. Belittle | Gender Breakdown | 0.00 | 0.05 | 381 | 0.957 |
| Wgt. Belittle | Visible Disability | 0.06 | 1.23 | 378 | 0.218 |
| Wgt. Belittle | Ethnic Name | 0.09 | 1.71 | 381 | 0.088 |
| Wgt. Belittle | Body-Mass Index | -0.01 | -0.10 | 314 | 0.917 |
| Wgt. Belittle | Accent | 0.06 | 1.21 | 381 | 0.228 |
| Wgt. Belittle | Redefinition Reassociate | -0.15 | -3.06 | 381 | 0.002 |
| Wgt. Belittle | Redefinition Regenerate | -0.06 | -1.12 | 381 | 0.264 |
| Wgt. Belittle | Switch Deemphasize | -0.01 | -0.25 | 381 | 0.799 |
| Wgt. Belittle | Switch Emphasize | -0.09 | -1.74 | 381 | 0.082 |
| Wgt. Belittle | Anxiety | 0.29 | 5.89 | 381 | < 0.001 |
| Wgt. Belittle | Guilt | 0.26 | 5.22 | 380 | < 0.001 |
| Wgt. Belittle | Shame | 0.30 | 6.21 | 381 | < 0.001 |
| Wgt. Belittle | Depression | 0.27 | 5.45 | 381 | < 0.001 |
| Wgt. Belittle | Core Self-Evaluations | -0.28 | -5.68 | 381 | < 0.001 |
| Wgt. Belittle | Weight-Bias Internalization | 0.21 | 4.10 | 381 | < 0.001 |
| Wgt. Belittle | Objectified Body Consc. | 0.10 | 1.87 | 381 | 0.062 |
| Wgt. Belittle | Intuitive Eating Trust | -0.04 | -0.79 | 381 | 0.432 |
| Wgt. Belittle | Body Dissatisfaction | 0.14 | 2.75 | 377 | 0.006 |
| Wgt. Belittle | Anti-fat Dislike | 0.50 | 11.39 | 381 | < 0.001 |
| Wgt. Belittle | Anti-fat Fear | 0.40 | 8.46 | 381 | < 0.001 |
| Wgt. Belittle | Anti-fat Willpower | 0.31 | 6.28 | 381 | < 0.001 |
| Wgt. Belittle | Thin Ideal | 0.30 | 6.19 | 380 | < 0.001 |
| Wgt. Belittle | Negative Body Talk Other | 0.46 | 10.12 | 381 | < 0.001 |
| Wgt. Belittle | Negative Body Talk Self | 0.17 | 3.33 | 381 | 0.001 |
| Wgt. Belittle | Job Task | -0.04 | -0.76 | 381 | 0.445 |
| Wgt. Belittle | Job Social | 0.06 | 1.12 | 381 | 0.263 |
| Wgt. Belittle | Job Physical | 0.00 | -0.04 | 381 | 0.967 |
| Offer Exercise Program | Offer Weight Loss | 0.87 | 33.66 | 381 | < 0.001 |
| Offer Exercise Program | Offer Maintain Health | 0.85 | 30.95 | 380 | < 0.001 |
| Offer Exercise Program | Comm. Exercise Program | 0.79 | 23.53 | 332 | < 0.001 |
| Offer Exercise Program | Comm. Weight Loss | 0.80 | 23.80 | 327 | < 0.001 |
| Offer Exercise Program | Comm. Maintain Health | 0.71 | 18.63 | 336 | < 0.001 |
| Offer Exercise Program | Education Breakdown | -0.13 | -2.65 | 380 | 0.008 |
| Offer Exercise Program | Weight Comparison | 0.08 | 1.64 | 377 | 0.102 |
| Offer Exercise Program | Gender Breakdown | -0.02 | -0.33 | 381 | 0.744 |
| Offer Exercise Program | Visible Disability | 0.11 | 2.17 | 378 | 0.030 |
| Offer Exercise Program | Ethnic Name | 0.09 | 1.68 | 381 | 0.094 |
| Offer Exercise Program | Body-Mass Index | -0.19 | -3.41 | 314 | 0.001 |
| Offer Exercise Program | Accent | 0.09 | 1.75 | 381 | 0.080 |
| Offer Exercise Program | Redefinition Reassociate | 0.21 | 4.14 | 381 | < 0.001 |
| Offer Exercise Program | Redefinition Regenerate | 0.14 | 2.83 | 381 | 0.005 |
| Offer Exercise Program | Switch Deemphasize | 0.05 | 1.03 | 381 | 0.302 |
| Offer Exercise Program | Switch Emphasize | 0.04 | 0.72 | 381 | 0.473 |
| Offer Exercise Program | Anxiety | -0.07 | -1.44 | 381 | 0.150 |
| Offer Exercise Program | Guilt | -0.06 | -1.18 | 380 | 0.238 |
| Offer Exercise Program | Shame | -0.08 | -1.53 | 381 | 0.127 |
| Offer Exercise Program | Depression | -0.12 | -2.39 | 381 | 0.017 |
| Offer Exercise Program | Core Self-Evaluations | 0.14 | 2.82 | 381 | 0.005 |
| Offer Exercise Program | Weight-Bias Internalization | -0.13 | -2.51 | 381 | 0.013 |
| Offer Exercise Program | Objectified Body Consc. | -0.03 | -0.55 | 381 | 0.580 |
| Offer Exercise Program | Intuitive Eating Trust | 0.13 | 2.49 | 381 | 0.013 |
| Offer Exercise Program | Body Dissatisfaction | -0.11 | -2.16 | 377 | 0.031 |
| Offer Exercise Program | Anti-fat Dislike | -0.01 | -0.12 | 381 | 0.905 |
| Offer Exercise Program | Anti-fat Fear | -0.03 | -0.66 | 381 | 0.510 |
| Offer Exercise Program | Anti-fat Willpower | -0.08 | -1.53 | 381 | 0.128 |
| Offer Exercise Program | Thin Ideal | 0.05 | 0.95 | 380 | 0.343 |
| Offer Exercise Program | Negative Body Talk Other | -0.05 | -0.90 | 381 | 0.367 |
| Offer Exercise Program | Negative Body Talk Self | 0.03 | 0.57 | 381 | 0.569 |
| Offer Exercise Program | Job Task | 0.12 | 2.31 | 381 | 0.021 |
| Offer Exercise Program | Job Social | 0.11 | 2.18 | 381 | 0.030 |
| Offer Exercise Program | Job Physical | -0.07 | -1.36 | 381 | 0.175 |
| Offer Weight Loss | Offer Maintain Health | 0.82 | 28.29 | 380 | < 0.001 |
| Offer Weight Loss | Comm. Exercise Program | 0.76 | 21.08 | 332 | < 0.001 |
| Offer Weight Loss | Comm. Weight Loss | 0.80 | 24.13 | 327 | < 0.001 |
| Offer Weight Loss | Comm. Maintain Health | 0.73 | 19.51 | 336 | < 0.001 |
| Offer Weight Loss | Education Breakdown | -0.17 | -3.36 | 380 | 0.001 |
| Offer Weight Loss | Weight Comparison | 0.04 | 0.75 | 377 | 0.455 |
| Offer Weight Loss | Gender Breakdown | 0.01 | 0.16 | 381 | 0.872 |
| Offer Weight Loss | Visible Disability | 0.04 | 0.84 | 378 | 0.402 |
| Offer Weight Loss | Ethnic Name | 0.06 | 1.10 | 381 | 0.271 |
| Offer Weight Loss | Body-Mass Index | -0.18 | -3.28 | 314 | 0.001 |
| Offer Weight Loss | Accent | 0.10 | 1.96 | 381 | 0.050 |
| Offer Weight Loss | Redefinition Reassociate | 0.19 | 3.84 | 381 | < 0.001 |
| Offer Weight Loss | Redefinition Regenerate | 0.14 | 2.80 | 381 | 0.005 |
| Offer Weight Loss | Switch Deemphasize | 0.04 | 0.87 | 381 | 0.384 |
| Offer Weight Loss | Switch Emphasize | 0.03 | 0.57 | 381 | 0.572 |
| Offer Weight Loss | Anxiety | -0.03 | -0.58 | 381 | 0.565 |
| Offer Weight Loss | Guilt | -0.03 | -0.53 | 380 | 0.593 |
| Offer Weight Loss | Shame | -0.09 | -1.85 | 381 | 0.065 |
| Offer Weight Loss | Depression | -0.06 | -1.27 | 381 | 0.205 |
| Offer Weight Loss | Core Self-Evaluations | 0.13 | 2.61 | 381 | 0.009 |
| Offer Weight Loss | Weight-Bias Internalization | -0.11 | -2.22 | 381 | 0.027 |
| Offer Weight Loss | Objectified Body Consc. | -0.05 | -1.06 | 381 | 0.291 |
| Offer Weight Loss | Intuitive Eating Trust | 0.10 | 2.05 | 381 | 0.041 |
| Offer Weight Loss | Body Dissatisfaction | -0.08 | -1.62 | 377 | 0.106 |
| Offer Weight Loss | Anti-fat Dislike | 0.02 | 0.39 | 381 | 0.694 |
| Offer Weight Loss | Anti-fat Fear | 0.00 | 0.00 | 381 | 0.999 |
| Offer Weight Loss | Anti-fat Willpower | -0.05 | -0.95 | 381 | 0.345 |
| Offer Weight Loss | Thin Ideal | 0.06 | 1.11 | 380 | 0.268 |
| Offer Weight Loss | Negative Body Talk Other | 0.00 | -0.02 | 381 | 0.980 |
| Offer Weight Loss | Negative Body Talk Self | 0.04 | 0.73 | 381 | 0.467 |
| Offer Weight Loss | Job Task | 0.14 | 2.79 | 381 | 0.006 |
| Offer Weight Loss | Job Social | 0.09 | 1.84 | 381 | 0.067 |
| Offer Weight Loss | Job Physical | -0.04 | -0.71 | 381 | 0.480 |
| Offer Maintain Health | Comm. Exercise Program | 0.75 | 20.92 | 331 | < 0.001 |
| Offer Maintain Health | Comm. Weight Loss | 0.78 | 22.43 | 326 | < 0.001 |
| Offer Maintain Health | Comm. Maintain Health | 0.79 | 23.27 | 335 | < 0.001 |
| Offer Maintain Health | Education Breakdown | -0.13 | -2.61 | 379 | 0.009 |
| Offer Maintain Health | Weight Comparison | 0.09 | 1.66 | 376 | 0.098 |
| Offer Maintain Health | Gender Breakdown | -0.01 | -0.15 | 380 | 0.884 |
| Offer Maintain Health | Visible Disability | 0.10 | 2.00 | 377 | 0.046 |
| Offer Maintain Health | Ethnic Name | 0.08 | 1.56 | 380 | 0.119 |
| Offer Maintain Health | Body-Mass Index | -0.20 | -3.52 | 313 | < 0.001 |
| Offer Maintain Health | Accent | 0.08 | 1.57 | 380 | 0.118 |
| Offer Maintain Health | Redefinition Reassociate | 0.19 | 3.82 | 380 | < 0.001 |
| Offer Maintain Health | Redefinition Regenerate | 0.11 | 2.15 | 380 | 0.032 |
| Offer Maintain Health | Switch Deemphasize | 0.03 | 0.62 | 380 | 0.538 |
| Offer Maintain Health | Switch Emphasize | 0.07 | 1.45 | 380 | 0.148 |
| Offer Maintain Health | Anxiety | -0.03 | -0.58 | 380 | 0.565 |
| Offer Maintain Health | Guilt | -0.05 | -1.07 | 379 | 0.286 |
| Offer Maintain Health | Shame | -0.08 | -1.50 | 380 | 0.135 |
| Offer Maintain Health | Depression | -0.08 | -1.55 | 380 | 0.121 |
| Offer Maintain Health | Core Self-Evaluations | 0.13 | 2.57 | 380 | 0.011 |
| Offer Maintain Health | Weight-Bias Internalization | -0.10 | -1.98 | 380 | 0.048 |
| Offer Maintain Health | Objectified Body Consc. | -0.04 | -0.69 | 380 | 0.488 |
| Offer Maintain Health | Intuitive Eating Trust | 0.10 | 1.88 | 380 | 0.061 |
| Offer Maintain Health | Body Dissatisfaction | -0.13 | -2.49 | 376 | 0.013 |
| Offer Maintain Health | Anti-fat Dislike | -0.05 | -1.04 | 380 | 0.299 |
| Offer Maintain Health | Anti-fat Fear | -0.04 | -0.75 | 380 | 0.453 |
| Offer Maintain Health | Anti-fat Willpower | -0.06 | -1.26 | 380 | 0.208 |
| Offer Maintain Health | Thin Ideal | 0.09 | 1.70 | 379 | 0.090 |
| Offer Maintain Health | Negative Body Talk Other | -0.08 | -1.63 | 380 | 0.105 |
| Offer Maintain Health | Negative Body Talk Self | 0.02 | 0.39 | 380 | 0.694 |
| Offer Maintain Health | Job Task | 0.12 | 2.43 | 380 | 0.016 |
| Offer Maintain Health | Job Social | 0.11 | 2.18 | 380 | 0.030 |
| Offer Maintain Health | Job Physical | -0.05 | -1.02 | 380 | 0.310 |
| Comm. Exercise Program | Comm. Weight Loss | 0.92 | 43.81 | 324 | < 0.001 |
| Comm. Exercise Program | Comm. Maintain Health | 0.88 | 34.09 | 328 | < 0.001 |
| Comm. Exercise Program | Education Breakdown | -0.18 | -3.36 | 331 | 0.001 |
| Comm. Exercise Program | Weight Comparison | 0.09 | 1.72 | 329 | 0.086 |
| Comm. Exercise Program | Gender Breakdown | -0.02 | -0.38 | 332 | 0.706 |
| Comm. Exercise Program | Visible Disability | 0.06 | 1.05 | 329 | 0.292 |
| Comm. Exercise Program | Ethnic Name | 0.13 | 2.30 | 332 | 0.022 |
| Comm. Exercise Program | Body-Mass Index | -0.19 | -3.28 | 274 | 0.001 |
| Comm. Exercise Program | Accent | 0.13 | 2.39 | 332 | 0.018 |
| Comm. Exercise Program | Redefinition Reassociate | 0.14 | 2.67 | 332 | 0.008 |
| Comm. Exercise Program | Redefinition Regenerate | 0.12 | 2.28 | 332 | 0.023 |
| Comm. Exercise Program | Switch Deemphasize | 0.03 | 0.53 | 332 | 0.600 |
| Comm. Exercise Program | Switch Emphasize | 0.07 | 1.31 | 332 | 0.192 |
| Comm. Exercise Program | Anxiety | -0.08 | -1.51 | 332 | 0.132 |
| Comm. Exercise Program | Guilt | -0.09 | -1.63 | 331 | 0.104 |
| Comm. Exercise Program | Shame | -0.11 | -2.04 | 332 | 0.042 |
| Comm. Exercise Program | Depression | -0.11 | -2.03 | 332 | 0.043 |
| Comm. Exercise Program | Core Self-Evaluations | 0.19 | 3.50 | 332 | 0.001 |
| Comm. Exercise Program | Weight-Bias Internalization | -0.13 | -2.35 | 332 | 0.019 |
| Comm. Exercise Program | Objectified Body Consc. | -0.03 | -0.58 | 332 | 0.562 |
| Comm. Exercise Program | Intuitive Eating Trust | 0.14 | 2.48 | 332 | 0.014 |
| Comm. Exercise Program | Body Dissatisfaction | -0.05 | -0.91 | 329 | 0.364 |
| Comm. Exercise Program | Anti-fat Dislike | -0.04 | -0.70 | 332 | 0.483 |
| Comm. Exercise Program | Anti-fat Fear | -0.06 | -1.07 | 332 | 0.285 |
| Comm. Exercise Program | Anti-fat Willpower | -0.07 | -1.30 | 332 | 0.194 |
| Comm. Exercise Program | Thin Ideal | 0.02 | 0.41 | 331 | 0.681 |
| Comm. Exercise Program | Negative Body Talk Other | -0.05 | -0.89 | 332 | 0.376 |
| Comm. Exercise Program | Negative Body Talk Self | -0.02 | -0.31 | 332 | 0.756 |
| Comm. Exercise Program | Job Task | 0.12 | 2.23 | 332 | 0.027 |
| Comm. Exercise Program | Job Social | 0.03 | 0.57 | 332 | 0.568 |
| Comm. Exercise Program | Job Physical | -0.01 | -0.20 | 332 | 0.843 |
| Comm. Weight Loss | Comm. Maintain Health | 0.90 | 36.31 | 324 | < 0.001 |
| Comm. Weight Loss | Education Breakdown | -0.17 | -3.05 | 326 | 0.003 |
| Comm. Weight Loss | Weight Comparison | 0.09 | 1.63 | 324 | 0.104 |
| Comm. Weight Loss | Gender Breakdown | -0.03 | -0.62 | 327 | 0.535 |
| Comm. Weight Loss | Visible Disability | 0.08 | 1.39 | 324 | 0.167 |
| Comm. Weight Loss | Ethnic Name | 0.09 | 1.68 | 327 | 0.095 |
| Comm. Weight Loss | Body-Mass Index | -0.17 | -2.75 | 269 | 0.006 |
| Comm. Weight Loss | Accent | 0.16 | 2.92 | 327 | 0.004 |
| Comm. Weight Loss | Redefinition Reassociate | 0.18 | 3.28 | 327 | 0.001 |
| Comm. Weight Loss | Redefinition Regenerate | 0.16 | 2.97 | 327 | 0.003 |
| Comm. Weight Loss | Switch Deemphasize | 0.04 | 0.76 | 327 | 0.450 |
| Comm. Weight Loss | Switch Emphasize | 0.07 | 1.25 | 327 | 0.211 |
| Comm. Weight Loss | Anxiety | -0.08 | -1.51 | 327 | 0.132 |
| Comm. Weight Loss | Guilt | -0.08 | -1.46 | 326 | 0.144 |
| Comm. Weight Loss | Shame | -0.13 | -2.38 | 327 | 0.018 |
| Comm. Weight Loss | Depression | -0.12 | -2.13 | 327 | 0.034 |
| Comm. Weight Loss | Core Self-Evaluations | 0.20 | 3.70 | 327 | < 0.001 |
| Comm. Weight Loss | Weight-Bias Internalization | -0.16 | -2.84 | 327 | 0.005 |
| Comm. Weight Loss | Objectified Body Consc. | -0.04 | -0.68 | 327 | 0.497 |
| Comm. Weight Loss | Intuitive Eating Trust | 0.18 | 3.38 | 327 | 0.001 |
| Comm. Weight Loss | Body Dissatisfaction | -0.05 | -0.85 | 323 | 0.399 |
| Comm. Weight Loss | Anti-fat Dislike | -0.02 | -0.45 | 327 | 0.656 |
| Comm. Weight Loss | Anti-fat Fear | -0.05 | -0.99 | 327 | 0.325 |
| Comm. Weight Loss | Anti-fat Willpower | -0.08 | -1.43 | 327 | 0.152 |
| Comm. Weight Loss | Thin Ideal | 0.06 | 1.10 | 326 | 0.272 |
| Comm. Weight Loss | Negative Body Talk Other | -0.04 | -0.78 | 327 | 0.434 |
| Comm. Weight Loss | Negative Body Talk Self | 0.00 | -0.05 | 327 | 0.962 |
| Comm. Weight Loss | Job Task | 0.13 | 2.32 | 327 | 0.021 |
| Comm. Weight Loss | Job Social | 0.05 | 0.99 | 327 | 0.321 |
| Comm. Weight Loss | Job Physical | 0.00 | -0.09 | 327 | 0.930 |
| Comm. Maintain Health | Education Breakdown | -0.15 | -2.75 | 335 | 0.006 |
| Comm. Maintain Health | Weight Comparison | 0.06 | 1.08 | 333 | 0.279 |
| Comm. Maintain Health | Gender Breakdown | 0.00 | 0.02 | 336 | 0.984 |
| Comm. Maintain Health | Visible Disability | 0.06 | 1.08 | 333 | 0.281 |
| Comm. Maintain Health | Ethnic Name | 0.08 | 1.39 | 336 | 0.165 |
| Comm. Maintain Health | Body-Mass Index | -0.19 | -3.19 | 279 | 0.002 |
| Comm. Maintain Health | Accent | 0.15 | 2.78 | 336 | 0.006 |
| Comm. Maintain Health | Redefinition Reassociate | 0.14 | 2.61 | 336 | 0.010 |
| Comm. Maintain Health | Redefinition Regenerate | 0.10 | 1.83 | 336 | 0.068 |
| Comm. Maintain Health | Switch Deemphasize | 0.02 | 0.30 | 336 | 0.768 |
| Comm. Maintain Health | Switch Emphasize | 0.07 | 1.20 | 336 | 0.232 |
| Comm. Maintain Health | Anxiety | -0.05 | -0.94 | 336 | 0.348 |
| Comm. Maintain Health | Guilt | -0.08 | -1.42 | 335 | 0.158 |
| Comm. Maintain Health | Shame | -0.11 | -1.95 | 336 | 0.052 |
| Comm. Maintain Health | Depression | -0.08 | -1.43 | 336 | 0.153 |
| Comm. Maintain Health | Core Self-Evaluations | 0.16 | 3.05 | 336 | 0.002 |
| Comm. Maintain Health | Weight-Bias Internalization | -0.14 | -2.65 | 336 | 0.009 |
| Comm. Maintain Health | Objectified Body Consc. | -0.04 | -0.65 | 336 | 0.513 |
| Comm. Maintain Health | Intuitive Eating Trust | 0.15 | 2.75 | 336 | 0.006 |
| Comm. Maintain Health | Body Dissatisfaction | -0.06 | -1.03 | 332 | 0.304 |
| Comm. Maintain Health | Anti-fat Dislike | -0.06 | -1.03 | 336 | 0.304 |
| Comm. Maintain Health | Anti-fat Fear | -0.09 | -1.71 | 336 | 0.089 |
| Comm. Maintain Health | Anti-fat Willpower | -0.09 | -1.75 | 336 | 0.082 |
| Comm. Maintain Health | Thin Ideal | 0.05 | 0.94 | 335 | 0.347 |
| Comm. Maintain Health | Negative Body Talk Other | -0.07 | -1.30 | 336 | 0.194 |
| Comm. Maintain Health | Negative Body Talk Self | 0.02 | 0.30 | 336 | 0.765 |
| Comm. Maintain Health | Job Task | 0.11 | 2.01 | 336 | 0.045 |
| Comm. Maintain Health | Job Social | 0.05 | 0.87 | 336 | 0.382 |
| Comm. Maintain Health | Job Physical | -0.02 | -0.43 | 336 | 0.667 |
| Education Breakdown | Weight Comparison | -0.03 | -0.92 | 991 | 0.356 |
| Education Breakdown | Gender Breakdown | 0.03 | 1.11 | 998 | 0.269 |
| Education Breakdown | Visible Disability | -0.09 | -2.99 | 996 | 0.003 |
| Education Breakdown | Ethnic Name | -0.11 | -3.36 | 998 | 0.001 |
| Education Breakdown | Body-Mass Index | 0.05 | 1.33 | 808 | 0.185 |
| Education Breakdown | Accent | -0.12 | -3.81 | 998 | < 0.001 |
| Education Breakdown | Redefinition Reassociate | -0.01 | -0.32 | 999 | 0.748 |
| Education Breakdown | Redefinition Regenerate | -0.08 | -2.43 | 999 | 0.015 |
| Education Breakdown | Switch Deemphasize | -0.01 | -0.43 | 999 | 0.667 |
| Education Breakdown | Switch Emphasize | 0.01 | 0.40 | 999 | 0.688 |
| Education Breakdown | Anxiety | -0.08 | -2.57 | 999 | 0.010 |
| Education Breakdown | Guilt | -0.10 | -3.28 | 996 | 0.001 |
| Education Breakdown | Shame | -0.01 | -0.43 | 998 | 0.669 |
| Education Breakdown | Depression | -0.06 | -1.92 | 997 | 0.056 |
| Education Breakdown | Core Self-Evaluations | 0.06 | 1.96 | 998 | 0.050 |
| Education Breakdown | Weight-Bias Internalization | -0.01 | -0.25 | 999 | 0.802 |
| Education Breakdown | Objectified Body Consc. | -0.09 | -3.01 | 999 | 0.003 |
| Education Breakdown | Intuitive Eating Trust | -0.08 | -2.51 | 999 | 0.012 |
| Education Breakdown | Body Dissatisfaction | 0.08 | 2.44 | 979 | 0.015 |
| Education Breakdown | Anti-fat Dislike | -0.07 | -1.33 | 380 | 0.185 |
| Education Breakdown | Anti-fat Fear | -0.08 | -1.57 | 380 | 0.117 |
| Education Breakdown | Anti-fat Willpower | 0.01 | 0.19 | 380 | 0.849 |
| Education Breakdown | Thin Ideal | -0.10 | -1.87 | 379 | 0.062 |
| Education Breakdown | Negative Body Talk Other | 0.01 | 0.23 | 380 | 0.815 |
| Education Breakdown | Negative Body Talk Self | -0.04 | -0.85 | 380 | 0.395 |
| Education Breakdown | Job Task | 0.03 | 1.10 | 999 | 0.273 |
| Education Breakdown | Job Social | 0.02 | 0.78 | 999 | 0.433 |
| Education Breakdown | Job Physical | 0.12 | 3.72 | 999 | < 0.001 |
| Weight Comparison | Gender Breakdown | -0.03 | -1.03 | 996 | 0.304 |
| Weight Comparison | Visible Disability | 0.06 | 1.97 | 995 | 0.049 |
| Weight Comparison | Ethnic Name | 0.06 | 1.93 | 996 | 0.054 |
| Weight Comparison | Body-Mass Index | -0.30 | -8.88 | 804 | < 0.001 |
| Weight Comparison | Accent | 0.10 | 3.21 | 996 | 0.001 |
| Weight Comparison | Redefinition Reassociate | 0.09 | 2.80 | 997 | 0.005 |
| Weight Comparison | Redefinition Regenerate | 0.15 | 4.85 | 997 | < 0.001 |
| Weight Comparison | Switch Deemphasize | 0.04 | 1.16 | 997 | 0.247 |
| Weight Comparison | Switch Emphasize | 0.09 | 2.99 | 997 | 0.003 |
| Weight Comparison | Anxiety | -0.11 | -3.50 | 997 | < 0.001 |
| Weight Comparison | Guilt | -0.11 | -3.42 | 994 | 0.001 |
| Weight Comparison | Shame | -0.20 | -6.35 | 996 | < 0.001 |
| Weight Comparison | Depression | -0.16 | -5.15 | 995 | < 0.001 |
| Weight Comparison | Core Self-Evaluations | 0.15 | 4.95 | 996 | < 0.001 |
| Weight Comparison | Weight-Bias Internalization | -0.22 | -6.99 | 997 | < 0.001 |
| Weight Comparison | Objectified Body Consc. | -0.05 | -1.66 | 997 | 0.096 |
| Weight Comparison | Intuitive Eating Trust | 0.16 | 5.26 | 997 | < 0.001 |
| Weight Comparison | Body Dissatisfaction | -0.24 | -7.70 | 977 | < 0.001 |
| Weight Comparison | Anti-fat Dislike | -0.13 | -2.45 | 377 | 0.015 |
| Weight Comparison | Anti-fat Fear | -0.17 | -3.38 | 377 | 0.001 |
| Weight Comparison | Anti-fat Willpower | -0.14 | -2.70 | 377 | 0.007 |
| Weight Comparison | Thin Ideal | -0.04 | -0.80 | 376 | 0.425 |
| Weight Comparison | Negative Body Talk Other | -0.07 | -1.38 | 377 | 0.168 |
| Weight Comparison | Negative Body Talk Self | 0.03 | 0.60 | 377 | 0.549 |
| Weight Comparison | Job Task | 0.07 | 2.15 | 997 | 0.032 |
| Weight Comparison | Job Social | 0.03 | 1.00 | 997 | 0.316 |
| Weight Comparison | Job Physical | 0.02 | 0.54 | 997 | 0.590 |
| Gender Breakdown | Visible Disability | -0.10 | -3.06 | 1001 | 0.002 |
| Gender Breakdown | Ethnic Name | -0.06 | -2.04 | 1003 | 0.041 |
| Gender Breakdown | Body-Mass Index | 0.07 | 1.94 | 810 | 0.053 |
| Gender Breakdown | Accent | -0.07 | -2.33 | 1003 | 0.020 |
| Gender Breakdown | Redefinition Reassociate | -0.05 | -1.72 | 1004 | 0.086 |
| Gender Breakdown | Redefinition Regenerate | -0.05 | -1.67 | 1004 | 0.095 |
| Gender Breakdown | Switch Deemphasize | -0.03 | -0.88 | 1004 | 0.378 |
| Gender Breakdown | Switch Emphasize | -0.03 | -0.92 | 1004 | 0.357 |
| Gender Breakdown | Anxiety | 0.01 | 0.23 | 1004 | 0.816 |
| Gender Breakdown | Guilt | 0.05 | 1.70 | 1001 | 0.090 |
| Gender Breakdown | Shame | 0.03 | 1.02 | 1003 | 0.307 |
| Gender Breakdown | Depression | 0.05 | 1.73 | 1002 | 0.083 |
| Gender Breakdown | Core Self-Evaluations | -0.06 | -1.83 | 1003 | 0.068 |
| Gender Breakdown | Weight-Bias Internalization | 0.06 | 1.76 | 1004 | 0.078 |
| Gender Breakdown | Objectified Body Consc. | 0.02 | 0.68 | 1004 | 0.495 |
| Gender Breakdown | Intuitive Eating Trust | -0.09 | -2.92 | 1004 | 0.004 |
| Gender Breakdown | Body Dissatisfaction | 0.07 | 2.20 | 984 | 0.028 |
| Gender Breakdown | Anti-fat Dislike | -0.08 | -1.50 | 381 | 0.134 |
| Gender Breakdown | Anti-fat Fear | 0.04 | 0.69 | 381 | 0.491 |
| Gender Breakdown | Anti-fat Willpower | 0.04 | 0.70 | 381 | 0.484 |
| Gender Breakdown | Thin Ideal | 0.10 | 1.93 | 380 | 0.054 |
| Gender Breakdown | Negative Body Talk Other | -0.01 | -0.18 | 381 | 0.854 |
| Gender Breakdown | Negative Body Talk Self | 0.06 | 1.22 | 381 | 0.222 |
| Gender Breakdown | Job Task | -0.13 | -4.31 | 1004 | < 0.001 |
| Gender Breakdown | Job Social | 0.06 | 1.84 | 1004 | 0.066 |
| Gender Breakdown | Job Physical | 0.06 | 1.97 | 1004 | 0.049 |
| Visible Disability | Ethnic Name | 0.36 | 12.12 | 1001 | < 0.001 |
| Visible Disability | Body-Mass Index | -0.02 | -0.56 | 809 | 0.579 |
| Visible Disability | Accent | 0.35 | 11.91 | 1001 | < 0.001 |
| Visible Disability | Redefinition Reassociate | 0.10 | 3.25 | 1002 | 0.001 |
| Visible Disability | Redefinition Regenerate | 0.15 | 4.89 | 1002 | < 0.001 |
| Visible Disability | Switch Deemphasize | 0.02 | 0.53 | 1002 | 0.596 |
| Visible Disability | Switch Emphasize | -0.03 | -0.81 | 1002 | 0.416 |
| Visible Disability | Anxiety | 0.17 | 5.46 | 1002 | < 0.001 |
| Visible Disability | Guilt | 0.21 | 6.67 | 999 | < 0.001 |
| Visible Disability | Shame | 0.07 | 2.28 | 1001 | 0.023 |
| Visible Disability | Depression | 0.12 | 3.97 | 1000 | < 0.001 |
| Visible Disability | Core Self-Evaluations | -0.10 | -3.24 | 1001 | 0.001 |
| Visible Disability | Weight-Bias Internalization | -0.02 | -0.63 | 1002 | 0.526 |
| Visible Disability | Objectified Body Consc. | 0.13 | 4.00 | 1002 | < 0.001 |
| Visible Disability | Intuitive Eating Trust | 0.15 | 4.69 | 1002 | < 0.001 |
| Visible Disability | Body Dissatisfaction | -0.19 | -6.10 | 982 | < 0.001 |
| Visible Disability | Anti-fat Dislike | 0.07 | 1.34 | 378 | 0.181 |
| Visible Disability | Anti-fat Fear | 0.04 | 0.73 | 378 | 0.469 |
| Visible Disability | Anti-fat Willpower | -0.05 | -1.05 | 378 | 0.295 |
| Visible Disability | Thin Ideal | 0.02 | 0.46 | 377 | 0.648 |
| Visible Disability | Negative Body Talk Other | 0.04 | 0.72 | 378 | 0.475 |
| Visible Disability | Negative Body Talk Self | 0.04 | 0.84 | 378 | 0.403 |
| Visible Disability | Job Task | -0.04 | -1.14 | 1002 | 0.256 |
| Visible Disability | Job Social | -0.01 | -0.22 | 1002 | 0.826 |
| Visible Disability | Job Physical | -0.05 | -1.61 | 1002 | 0.108 |
| Ethnic Name | Body-Mass Index | -0.14 | -3.91 | 810 | < 0.001 |
| Ethnic Name | Accent | 0.51 | 18.79 | 1003 | < 0.001 |
| Ethnic Name | Redefinition Reassociate | 0.11 | 3.42 | 1004 | 0.001 |
| Ethnic Name | Redefinition Regenerate | 0.15 | 4.87 | 1004 | < 0.001 |
| Ethnic Name | Switch Deemphasize | 0.03 | 1.05 | 1004 | 0.293 |
| Ethnic Name | Switch Emphasize | 0.07 | 2.15 | 1004 | 0.031 |
| Ethnic Name | Anxiety | 0.07 | 2.13 | 1004 | 0.033 |
| Ethnic Name | Guilt | 0.10 | 3.23 | 1001 | 0.001 |
| Ethnic Name | Shame | 0.00 | 0.12 | 1003 | 0.904 |
| Ethnic Name | Depression | 0.01 | 0.45 | 1002 | 0.651 |
| Ethnic Name | Core Self-Evaluations | -0.01 | -0.40 | 1003 | 0.691 |
| Ethnic Name | Weight-Bias Internalization | -0.05 | -1.60 | 1004 | 0.110 |
| Ethnic Name | Objectified Body Consc. | 0.10 | 3.09 | 1004 | 0.002 |
| Ethnic Name | Intuitive Eating Trust | 0.17 | 5.50 | 1004 | < 0.001 |
| Ethnic Name | Body Dissatisfaction | -0.16 | -5.06 | 984 | < 0.001 |
| Ethnic Name | Anti-fat Dislike | 0.11 | 2.10 | 381 | 0.037 |
| Ethnic Name | Anti-fat Fear | 0.10 | 2.04 | 381 | 0.043 |
| Ethnic Name | Anti-fat Willpower | 0.01 | 0.26 | 381 | 0.792 |
| Ethnic Name | Thin Ideal | 0.06 | 1.18 | 380 | 0.240 |
| Ethnic Name | Negative Body Talk Other | 0.13 | 2.49 | 381 | 0.013 |
| Ethnic Name | Negative Body Talk Self | -0.03 | -0.49 | 381 | 0.623 |
| Ethnic Name | Job Task | -0.03 | -1.02 | 1004 | 0.307 |
| Ethnic Name | Job Social | -0.02 | -0.48 | 1004 | 0.630 |
| Ethnic Name | Job Physical | -0.05 | -1.71 | 1004 | 0.087 |
| Body-Mass Index | Accent | -0.11 | -3.09 | 810 | 0.002 |
| Body-Mass Index | Redefinition Reassociate | 0.00 | -0.14 | 811 | 0.888 |
| Body-Mass Index | Redefinition Regenerate | -0.03 | -0.84 | 811 | 0.403 |
| Body-Mass Index | Switch Deemphasize | 0.00 | -0.03 | 811 | 0.972 |
| Body-Mass Index | Switch Emphasize | -0.05 | -1.48 | 811 | 0.141 |
| Body-Mass Index | Anxiety | 0.02 | 0.61 | 811 | 0.540 |
| Body-Mass Index | Guilt | 0.07 | 1.93 | 809 | 0.054 |
| Body-Mass Index | Shame | 0.12 | 3.58 | 810 | < 0.001 |
| Body-Mass Index | Depression | 0.08 | 2.21 | 809 | 0.027 |
| Body-Mass Index | Core Self-Evaluations | -0.10 | -2.83 | 810 | 0.005 |
| Body-Mass Index | Weight-Bias Internalization | 0.14 | 3.94 | 811 | < 0.001 |
| Body-Mass Index | Objectified Body Consc. | 0.03 | 0.89 | 811 | 0.374 |
| Body-Mass Index | Intuitive Eating Trust | -0.10 | -2.89 | 811 | 0.004 |
| Body-Mass Index | Body Dissatisfaction | 0.27 | 7.80 | 793 | < 0.001 |
| Body-Mass Index | Anti-fat Dislike | 0.08 | 1.43 | 314 | 0.154 |
| Body-Mass Index | Anti-fat Fear | 0.11 | 1.88 | 314 | 0.061 |
| Body-Mass Index | Anti-fat Willpower | 0.07 | 1.17 | 314 | 0.242 |
| Body-Mass Index | Thin Ideal | 0.08 | 1.50 | 313 | 0.134 |
| Body-Mass Index | Negative Body Talk Other | 0.06 | 1.02 | 314 | 0.309 |
| Body-Mass Index | Negative Body Talk Self | 0.06 | 1.11 | 314 | 0.268 |
| Body-Mass Index | Job Task | -0.01 | -0.27 | 811 | 0.786 |
| Body-Mass Index | Job Social | 0.00 | 0.05 | 811 | 0.960 |
| Body-Mass Index | Job Physical | 0.02 | 0.44 | 811 | 0.658 |
| Accent | Redefinition Reassociate | 0.15 | 4.82 | 1004 | < 0.001 |
| Accent | Redefinition Regenerate | 0.17 | 5.35 | 1004 | < 0.001 |
| Accent | Switch Deemphasize | 0.05 | 1.63 | 1004 | 0.103 |
| Accent | Switch Emphasize | 0.05 | 1.46 | 1004 | 0.144 |
| Accent | Anxiety | 0.07 | 2.20 | 1004 | 0.028 |
| Accent | Guilt | 0.08 | 2.47 | 1001 | 0.014 |
| Accent | Shame | -0.03 | -0.83 | 1003 | 0.409 |
| Accent | Depression | 0.01 | 0.22 | 1002 | 0.824 |
| Accent | Core Self-Evaluations | -0.01 | -0.33 | 1003 | 0.745 |
| Accent | Weight-Bias Internalization | -0.09 | -2.90 | 1004 | 0.004 |
| Accent | Objectified Body Consc. | 0.12 | 3.85 | 1004 | < 0.001 |
| Accent | Intuitive Eating Trust | 0.17 | 5.33 | 1004 | < 0.001 |
| Accent | Body Dissatisfaction | -0.18 | -5.69 | 984 | < 0.001 |
| Accent | Anti-fat Dislike | 0.07 | 1.31 | 381 | 0.191 |
| Accent | Anti-fat Fear | 0.04 | 0.81 | 381 | 0.416 |
| Accent | Anti-fat Willpower | 0.01 | 0.14 | 381 | 0.892 |
| Accent | Thin Ideal | 0.15 | 2.86 | 380 | 0.004 |
| Accent | Negative Body Talk Other | 0.10 | 1.95 | 381 | 0.052 |
| Accent | Negative Body Talk Self | 0.02 | 0.44 | 381 | 0.659 |
| Accent | Job Task | -0.03 | -0.82 | 1004 | 0.415 |
| Accent | Job Social | 0.03 | 0.81 | 1004 | 0.421 |
| Accent | Job Physical | -0.07 | -2.37 | 1004 | 0.018 |
| Redefinition Reassociate | Redefinition Regenerate | 0.59 | 23.34 | 1006 | < 0.001 |
| Redefinition Reassociate | Switch Deemphasize | 0.34 | 11.49 | 1006 | < 0.001 |
| Redefinition Reassociate | Switch Emphasize | 0.36 | 12.12 | 1006 | < 0.001 |
| Redefinition Reassociate | Anxiety | -0.22 | -7.07 | 1006 | < 0.001 |
| Redefinition Reassociate | Guilt | -0.22 | -7.19 | 1003 | < 0.001 |
| Redefinition Reassociate | Shame | -0.34 | -11.47 | 1005 | < 0.001 |
| Redefinition Reassociate | Depression | -0.35 | -11.93 | 1004 | < 0.001 |
| Redefinition Reassociate | Core Self-Evaluations | 0.41 | 14.19 | 1005 | < 0.001 |
| Redefinition Reassociate | Weight-Bias Internalization | -0.47 | -17.07 | 1006 | < 0.001 |
| Redefinition Reassociate | Objectified Body Consc. | 0.00 | 0.16 | 1006 | 0.876 |
| Redefinition Reassociate | Intuitive Eating Trust | 0.43 | 15.22 | 1006 | < 0.001 |
| Redefinition Reassociate | Body Dissatisfaction | -0.26 | -8.53 | 985 | < 0.001 |
| Redefinition Reassociate | Anti-fat Dislike | -0.16 | -3.07 | 381 | 0.002 |
| Redefinition Reassociate | Anti-fat Fear | -0.15 | -2.93 | 381 | 0.004 |
| Redefinition Reassociate | Anti-fat Willpower | -0.16 | -3.19 | 381 | 0.002 |
| Redefinition Reassociate | Thin Ideal | -0.08 | -1.50 | 380 | 0.135 |
| Redefinition Reassociate | Negative Body Talk Other | -0.15 | -2.94 | 381 | 0.004 |
| Redefinition Reassociate | Negative Body Talk Self | -0.02 | -0.45 | 381 | 0.650 |
| Redefinition Reassociate | Job Task | 0.05 | 1.47 | 1005 | 0.143 |
| Redefinition Reassociate | Job Social | 0.04 | 1.14 | 1005 | 0.256 |
| Redefinition Reassociate | Job Physical | 0.03 | 0.90 | 1005 | 0.368 |
| Redefinition Regenerate | Switch Deemphasize | 0.53 | 19.76 | 1006 | < 0.001 |
| Redefinition Regenerate | Switch Emphasize | 0.51 | 19.01 | 1006 | < 0.001 |
| Redefinition Regenerate | Anxiety | -0.18 | -5.68 | 1006 | < 0.001 |
| Redefinition Regenerate | Guilt | -0.22 | -7.26 | 1003 | < 0.001 |
| Redefinition Regenerate | Shame | -0.32 | -10.61 | 1005 | < 0.001 |
| Redefinition Regenerate | Depression | -0.34 | -11.30 | 1004 | < 0.001 |
| Redefinition Regenerate | Core Self-Evaluations | 0.38 | 13.03 | 1005 | < 0.001 |
| Redefinition Regenerate | Weight-Bias Internalization | -0.51 | -18.71 | 1006 | < 0.001 |
| Redefinition Regenerate | Objectified Body Consc. | 0.03 | 0.85 | 1006 | 0.394 |
| Redefinition Regenerate | Intuitive Eating Trust | 0.52 | 19.20 | 1006 | < 0.001 |
| Redefinition Regenerate | Body Dissatisfaction | -0.28 | -8.99 | 985 | < 0.001 |
| Redefinition Regenerate | Anti-fat Dislike | -0.07 | -1.33 | 381 | 0.186 |
| Redefinition Regenerate | Anti-fat Fear | -0.11 | -2.11 | 381 | 0.036 |
| Redefinition Regenerate | Anti-fat Willpower | -0.18 | -3.50 | 381 | 0.001 |
| Redefinition Regenerate | Thin Ideal | 0.00 | -0.06 | 380 | 0.950 |
| Redefinition Regenerate | Negative Body Talk Other | -0.12 | -2.45 | 381 | 0.015 |
| Redefinition Regenerate | Negative Body Talk Self | 0.02 | 0.39 | 381 | 0.697 |
| Redefinition Regenerate | Job Task | -0.01 | -0.18 | 1005 | 0.856 |
| Redefinition Regenerate | Job Social | 0.00 | -0.05 | 1005 | 0.959 |
| Redefinition Regenerate | Job Physical | -0.02 | -0.58 | 1005 | 0.564 |
| Switch Deemphasize | Switch Emphasize | 0.71 | 31.91 | 1006 | < 0.001 |
| Switch Deemphasize | Anxiety | -0.18 | -5.68 | 1006 | < 0.001 |
| Switch Deemphasize | Guilt | -0.24 | -7.67 | 1003 | < 0.001 |
| Switch Deemphasize | Shame | -0.21 | -6.82 | 1005 | < 0.001 |
| Switch Deemphasize | Depression | -0.29 | -9.59 | 1004 | < 0.001 |
| Switch Deemphasize | Core Self-Evaluations | 0.36 | 12.40 | 1005 | < 0.001 |
| Switch Deemphasize | Weight-Bias Internalization | -0.32 | -10.53 | 1006 | < 0.001 |
| Switch Deemphasize | Objectified Body Consc. | -0.03 | -0.87 | 1006 | 0.387 |
| Switch Deemphasize | Intuitive Eating Trust | 0.33 | 10.93 | 1006 | < 0.001 |
| Switch Deemphasize | Body Dissatisfaction | -0.16 | -5.04 | 985 | < 0.001 |
| Switch Deemphasize | Anti-fat Dislike | -0.06 | -1.14 | 381 | 0.257 |
| Switch Deemphasize | Anti-fat Fear | -0.04 | -0.72 | 381 | 0.471 |
| Switch Deemphasize | Anti-fat Willpower | -0.03 | -0.64 | 381 | 0.523 |
| Switch Deemphasize | Thin Ideal | -0.07 | -1.44 | 380 | 0.152 |
| Switch Deemphasize | Negative Body Talk Other | -0.12 | -2.38 | 381 | 0.018 |
| Switch Deemphasize | Negative Body Talk Self | -0.03 | -0.53 | 381 | 0.596 |
| Switch Deemphasize | Job Task | 0.02 | 0.51 | 1005 | 0.612 |
| Switch Deemphasize | Job Social | 0.00 | -0.11 | 1005 | 0.912 |
| Switch Deemphasize | Job Physical | -0.02 | -0.56 | 1005 | 0.577 |
| Switch Emphasize | Anxiety | -0.30 | -9.96 | 1006 | < 0.001 |
| Switch Emphasize | Guilt | -0.35 | -11.68 | 1003 | < 0.001 |
| Switch Emphasize | Shame | -0.34 | -11.39 | 1005 | < 0.001 |
| Switch Emphasize | Depression | -0.42 | -14.71 | 1004 | < 0.001 |
| Switch Emphasize | Core Self-Evaluations | 0.51 | 18.64 | 1005 | < 0.001 |
| Switch Emphasize | Weight-Bias Internalization | -0.41 | -14.33 | 1006 | < 0.001 |
| Switch Emphasize | Objectified Body Consc. | -0.09 | -2.84 | 1006 | 0.005 |
| Switch Emphasize | Intuitive Eating Trust | 0.36 | 12.13 | 1006 | < 0.001 |
| Switch Emphasize | Body Dissatisfaction | -0.19 | -6.16 | 985 | < 0.001 |
| Switch Emphasize | Anti-fat Dislike | -0.12 | -2.33 | 381 | 0.020 |
| Switch Emphasize | Anti-fat Fear | -0.07 | -1.38 | 381 | 0.168 |
| Switch Emphasize | Anti-fat Willpower | -0.05 | -0.94 | 381 | 0.347 |
| Switch Emphasize | Thin Ideal | -0.09 | -1.75 | 380 | 0.080 |
| Switch Emphasize | Negative Body Talk Other | -0.15 | -2.90 | 381 | 0.004 |
| Switch Emphasize | Negative Body Talk Self | -0.02 | -0.45 | 381 | 0.653 |
| Switch Emphasize | Job Task | 0.03 | 0.96 | 1005 | 0.339 |
| Switch Emphasize | Job Social | 0.03 | 0.89 | 1005 | 0.372 |
| Switch Emphasize | Job Physical | 0.00 | -0.05 | 1005 | 0.960 |
| Anxiety | Guilt | 0.61 | 24.42 | 1003 | < 0.001 |
| Anxiety | Shame | 0.56 | 21.39 | 1005 | < 0.001 |
| Anxiety | Depression | 0.76 | 36.49 | 1004 | < 0.001 |
| Anxiety | Core Self-Evaluations | -0.64 | -26.72 | 1005 | < 0.001 |
| Anxiety | Weight-Bias Internalization | 0.49 | 17.82 | 1006 | < 0.001 |
| Anxiety | Objectified Body Consc. | 0.33 | 11.25 | 1006 | < 0.001 |
| Anxiety | Intuitive Eating Trust | -0.22 | -7.25 | 1006 | < 0.001 |
| Anxiety | Body Dissatisfaction | 0.14 | 4.32 | 985 | < 0.001 |
| Anxiety | Anti-fat Dislike | 0.26 | 5.35 | 381 | < 0.001 |
| Anxiety | Anti-fat Fear | 0.24 | 4.80 | 381 | < 0.001 |
| Anxiety | Anti-fat Willpower | 0.14 | 2.76 | 381 | 0.006 |
| Anxiety | Thin Ideal | 0.20 | 4.02 | 380 | < 0.001 |
| Anxiety | Negative Body Talk Other | 0.19 | 3.80 | 381 | < 0.001 |
| Anxiety | Negative Body Talk Self | 0.06 | 1.18 | 381 | 0.238 |
| Anxiety | Job Task | -0.06 | -1.75 | 1005 | 0.080 |
| Anxiety | Job Social | 0.00 | -0.04 | 1005 | 0.969 |
| Anxiety | Job Physical | 0.04 | 1.23 | 1005 | 0.218 |
| Guilt | Shame | 0.57 | 21.81 | 1003 | < 0.001 |
| Guilt | Depression | 0.68 | 29.06 | 1002 | < 0.001 |
| Guilt | Core Self-Evaluations | -0.61 | -24.21 | 1003 | < 0.001 |
| Guilt | Weight-Bias Internalization | 0.53 | 19.75 | 1003 | < 0.001 |
| Guilt | Objectified Body Consc. | 0.30 | 9.86 | 1003 | < 0.001 |
| Guilt | Intuitive Eating Trust | -0.18 | -5.90 | 1003 | < 0.001 |
| Guilt | Body Dissatisfaction | 0.11 | 3.48 | 982 | 0.001 |
| Guilt | Anti-fat Dislike | 0.25 | 5.02 | 380 | < 0.001 |
| Guilt | Anti-fat Fear | 0.25 | 4.97 | 380 | < 0.001 |
| Guilt | Anti-fat Willpower | 0.13 | 2.52 | 380 | 0.012 |
| Guilt | Thin Ideal | 0.22 | 4.33 | 379 | < 0.001 |
| Guilt | Negative Body Talk Other | 0.27 | 5.37 | 380 | < 0.001 |
| Guilt | Negative Body Talk Self | 0.12 | 2.39 | 380 | 0.017 |
| Guilt | Job Task | -0.04 | -1.40 | 1002 | 0.161 |
| Guilt | Job Social | 0.00 | 0.05 | 1002 | 0.959 |
| Guilt | Job Physical | 0.03 | 0.95 | 1002 | 0.343 |
| Shame | Depression | 0.68 | 29.78 | 1004 | < 0.001 |
| Shame | Core Self-Evaluations | -0.72 | -32.91 | 1005 | < 0.001 |
| Shame | Weight-Bias Internalization | 0.70 | 31.20 | 1005 | < 0.001 |
| Shame | Objectified Body Consc. | 0.29 | 9.71 | 1005 | < 0.001 |
| Shame | Intuitive Eating Trust | -0.31 | -10.48 | 1005 | < 0.001 |
| Shame | Body Dissatisfaction | 0.25 | 8.10 | 984 | < 0.001 |
| Shame | Anti-fat Dislike | 0.32 | 6.60 | 381 | < 0.001 |
| Shame | Anti-fat Fear | 0.36 | 7.50 | 381 | < 0.001 |
| Shame | Anti-fat Willpower | 0.33 | 6.76 | 381 | < 0.001 |
| Shame | Thin Ideal | 0.28 | 5.62 | 380 | < 0.001 |
| Shame | Negative Body Talk Other | 0.26 | 5.23 | 381 | < 0.001 |
| Shame | Negative Body Talk Self | 0.15 | 2.94 | 381 | 0.004 |
| Shame | Job Task | -0.04 | -1.19 | 1004 | 0.235 |
| Shame | Job Social | 0.00 | 0.10 | 1004 | 0.922 |
| Shame | Job Physical | 0.05 | 1.55 | 1004 | 0.121 |
| Depression | Core Self-Evaluations | -0.77 | -38.12 | 1004 | < 0.001 |
| Depression | Weight-Bias Internalization | 0.66 | 27.49 | 1004 | < 0.001 |
| Depression | Objectified Body Consc. | 0.33 | 11.10 | 1004 | < 0.001 |
| Depression | Intuitive Eating Trust | -0.32 | -10.82 | 1004 | < 0.001 |
| Depression | Body Dissatisfaction | 0.23 | 7.39 | 983 | < 0.001 |
| Depression | Anti-fat Dislike | 0.27 | 5.49 | 381 | < 0.001 |
| Depression | Anti-fat Fear | 0.26 | 5.30 | 381 | < 0.001 |
| Depression | Anti-fat Willpower | 0.20 | 3.97 | 381 | < 0.001 |
| Depression | Thin Ideal | 0.21 | 4.18 | 380 | < 0.001 |
| Depression | Negative Body Talk Other | 0.23 | 4.66 | 381 | < 0.001 |
| Depression | Negative Body Talk Self | 0.06 | 1.22 | 381 | 0.225 |
| Depression | Job Task | -0.04 | -1.18 | 1003 | 0.239 |
| Depression | Job Social | 0.02 | 0.61 | 1003 | 0.541 |
| Depression | Job Physical | 0.06 | 1.84 | 1003 | 0.067 |
| Core Self-Evaluations | Weight-Bias Internalization | -0.68 | -29.42 | 1005 | < 0.001 |
| Core Self-Evaluations | Objectified Body Consc. | -0.28 | -9.39 | 1005 | < 0.001 |
| Core Self-Evaluations | Intuitive Eating Trust | 0.35 | 11.72 | 1005 | < 0.001 |
| Core Self-Evaluations | Body Dissatisfaction | -0.22 | -7.23 | 984 | < 0.001 |
| Core Self-Evaluations | Anti-fat Dislike | -0.29 | -5.97 | 381 | < 0.001 |
| Core Self-Evaluations | Anti-fat Fear | -0.29 | -5.90 | 381 | < 0.001 |
| Core Self-Evaluations | Anti-fat Willpower | -0.22 | -4.47 | 381 | < 0.001 |
| Core Self-Evaluations | Thin Ideal | -0.26 | -5.17 | 380 | < 0.001 |
| Core Self-Evaluations | Negative Body Talk Other | -0.29 | -5.99 | 381 | < 0.001 |
| Core Self-Evaluations | Negative Body Talk Self | -0.09 | -1.81 | 381 | 0.071 |
| Core Self-Evaluations | Job Task | 0.08 | 2.57 | 1004 | 0.010 |
| Core Self-Evaluations | Job Social | 0.00 | 0.08 | 1004 | 0.937 |
| Core Self-Evaluations | Job Physical | -0.04 | -1.21 | 1004 | 0.228 |
| Weight-Bias Internalization | Objectified Body Consc. | 0.23 | 7.39 | 1006 | < 0.001 |
| Weight-Bias Internalization | Intuitive Eating Trust | -0.46 | -16.31 | 1006 | < 0.001 |
| Weight-Bias Internalization | Body Dissatisfaction | 0.33 | 10.81 | 985 | < 0.001 |
| Weight-Bias Internalization | Anti-fat Dislike | 0.26 | 5.32 | 381 | < 0.001 |
| Weight-Bias Internalization | Anti-fat Fear | 0.33 | 6.81 | 381 | < 0.001 |
| Weight-Bias Internalization | Anti-fat Willpower | 0.32 | 6.69 | 381 | < 0.001 |
| Weight-Bias Internalization | Thin Ideal | 0.28 | 5.79 | 380 | < 0.001 |
| Weight-Bias Internalization | Negative Body Talk Other | 0.26 | 5.28 | 381 | < 0.001 |
| Weight-Bias Internalization | Negative Body Talk Self | 0.14 | 2.82 | 381 | 0.005 |
| Weight-Bias Internalization | Job Task | -0.04 | -1.42 | 1005 | 0.155 |
| Weight-Bias Internalization | Job Social | 0.02 | 0.76 | 1005 | 0.448 |
| Weight-Bias Internalization | Job Physical | 0.02 | 0.50 | 1005 | 0.619 |
| Objectified Body Consc. | Intuitive Eating Trust | 0.05 | 1.51 | 1006 | 0.131 |
| Objectified Body Consc. | Body Dissatisfaction | 0.00 | -0.13 | 985 | 0.899 |
| Objectified Body Consc. | Anti-fat Dislike | 0.13 | 2.59 | 381 | 0.010 |
| Objectified Body Consc. | Anti-fat Fear | 0.10 | 1.91 | 381 | 0.057 |
| Objectified Body Consc. | Anti-fat Willpower | 0.04 | 0.74 | 381 | 0.460 |
| Objectified Body Consc. | Thin Ideal | 0.13 | 2.50 | 380 | 0.013 |
| Objectified Body Consc. | Negative Body Talk Other | 0.06 | 1.12 | 381 | 0.262 |
| Objectified Body Consc. | Negative Body Talk Self | 0.09 | 1.80 | 381 | 0.072 |
| Objectified Body Consc. | Job Task | -0.06 | -1.85 | 1005 | 0.065 |
| Objectified Body Consc. | Job Social | 0.00 | -0.03 | 1005 | 0.973 |
| Objectified Body Consc. | Job Physical | 0.04 | 1.39 | 1005 | 0.166 |
| Intuitive Eating Trust | Body Dissatisfaction | -0.31 | -10.29 | 985 | < 0.001 |
| Intuitive Eating Trust | Anti-fat Dislike | -0.07 | -1.44 | 381 | 0.150 |
| Intuitive Eating Trust | Anti-fat Fear | -0.15 | -2.94 | 381 | 0.003 |
| Intuitive Eating Trust | Anti-fat Willpower | -0.22 | -4.50 | 381 | < 0.001 |
| Intuitive Eating Trust | Thin Ideal | -0.13 | -2.61 | 380 | 0.009 |
| Intuitive Eating Trust | Negative Body Talk Other | -0.11 | -2.17 | 381 | 0.031 |
| Intuitive Eating Trust | Negative Body Talk Self | -0.03 | -0.66 | 381 | 0.512 |
| Intuitive Eating Trust | Job Task | 0.01 | 0.18 | 1005 | 0.859 |
| Intuitive Eating Trust | Job Social | 0.02 | 0.78 | 1005 | 0.436 |
| Intuitive Eating Trust | Job Physical | -0.03 | -0.86 | 1005 | 0.389 |
| Body Dissatisfaction | Anti-fat Dislike | 0.15 | 2.90 | 377 | 0.004 |
| Body Dissatisfaction | Anti-fat Fear | 0.16 | 3.22 | 377 | 0.001 |
| Body Dissatisfaction | Anti-fat Willpower | 0.12 | 2.32 | 377 | 0.021 |
| Body Dissatisfaction | Thin Ideal | 0.19 | 3.77 | 376 | < 0.001 |
| Body Dissatisfaction | Negative Body Talk Other | 0.20 | 3.98 | 377 | < 0.001 |
| Body Dissatisfaction | Negative Body Talk Self | -0.01 | -0.17 | 377 | 0.862 |
| Body Dissatisfaction | Job Task | -0.05 | -1.72 | 985 | 0.086 |
| Body Dissatisfaction | Job Social | -0.06 | -1.73 | 985 | 0.084 |
| Body Dissatisfaction | Job Physical | 0.02 | 0.75 | 985 | 0.454 |
| Anti-fat Dislike | Anti-fat Fear | 0.78 | 24.39 | 381 | < 0.001 |
| Anti-fat Dislike | Anti-fat Willpower | 0.62 | 15.51 | 381 | < 0.001 |
| Anti-fat Dislike | Thin Ideal | 0.44 | 9.44 | 380 | < 0.001 |
| Anti-fat Dislike | Negative Body Talk Other | 0.72 | 20.28 | 381 | < 0.001 |
| Anti-fat Dislike | Negative Body Talk Self | 0.25 | 5.07 | 381 | < 0.001 |
| Anti-fat Dislike | Job Task | 0.02 | 0.40 | 381 | 0.690 |
| Anti-fat Dislike | Job Social | 0.02 | 0.41 | 381 | 0.680 |
| Anti-fat Dislike | Job Physical | 0.03 | 0.51 | 381 | 0.612 |
| Anti-fat Fear | Anti-fat Willpower | 0.76 | 22.62 | 381 | < 0.001 |
| Anti-fat Fear | Thin Ideal | 0.55 | 12.95 | 380 | < 0.001 |
| Anti-fat Fear | Negative Body Talk Other | 0.68 | 18.12 | 381 | < 0.001 |
| Anti-fat Fear | Negative Body Talk Self | 0.35 | 7.21 | 381 | < 0.001 |
| Anti-fat Fear | Job Task | 0.07 | 1.45 | 381 | 0.148 |
| Anti-fat Fear | Job Social | 0.09 | 1.83 | 381 | 0.068 |
| Anti-fat Fear | Job Physical | 0.04 | 0.73 | 381 | 0.465 |
| Anti-fat Willpower | Thin Ideal | 0.46 | 10.12 | 380 | < 0.001 |
| Anti-fat Willpower | Negative Body Talk Other | 0.60 | 14.69 | 381 | < 0.001 |
| Anti-fat Willpower | Negative Body Talk Self | 0.30 | 6.03 | 381 | < 0.001 |
| Anti-fat Willpower | Job Task | 0.08 | 1.47 | 381 | 0.142 |
| Anti-fat Willpower | Job Social | 0.04 | 0.71 | 381 | 0.478 |
| Anti-fat Willpower | Job Physical | 0.05 | 0.92 | 381 | 0.359 |
| Thin Ideal | Negative Body Talk Other | 0.44 | 9.44 | 380 | < 0.001 |
| Thin Ideal | Negative Body Talk Self | 0.43 | 9.22 | 380 | < 0.001 |
| Thin Ideal | Job Task | 0.01 | 0.23 | 380 | 0.819 |
| Thin Ideal | Job Social | 0.14 | 2.72 | 380 | 0.007 |
| Thin Ideal | Job Physical | -0.06 | -1.17 | 380 | 0.242 |
| Negative Body Talk Other | Negative Body Talk Self | 0.35 | 7.41 | 381 | < 0.001 |
| Negative Body Talk Other | Job Task | 0.04 | 0.71 | 381 | 0.477 |
| Negative Body Talk Other | Job Social | 0.03 | 0.58 | 381 | 0.564 |
| Negative Body Talk Other | Job Physical | 0.08 | 1.65 | 381 | 0.100 |
| Negative Body Talk Self | Job Task | -0.03 | -0.61 | 381 | 0.544 |
| Negative Body Talk Self | Job Social | 0.05 | 0.88 | 381 | 0.377 |
| Negative Body Talk Self | Job Physical | 0.03 | 0.49 | 381 | 0.623 |
| Job Task | Job Social | 0.33 | 11.25 | 1005 | < 0.001 |
| Job Task | Job Physical | 0.20 | 6.43 | 1005 | < 0.001 |
| Job Social | Job Physical | 0.01 | 0.44 | 1005 | 0.659 |

Incl. = Inclusion; Org. = Organizational; Neg. = Negative; Wgt. = Weight; Comm. = Communications; Imp. = Importance; Phys. = Physical; Consc. = Consciousness; Gender: Male = 0 and Female = 1; Race: Non-White = 0 and White = 1; r = Pearson product-moment correlation; t = t-test value; df = degrees of freedom; p = frequentist probability value.
