# Supplementary material for: An exploratory study of the correlates of weight-based mistreatment at work
Source: PLoS One. 2026 Aug 26;21(8):e0342144. doi: 10.1371/journal.pone.0342144 (PMC13514019; doi:10.1371/journal.pone.0342144)
Supplement: Supplementary file 18 [file pone.0342144.s018.docx]

**S18 Table. Occupational Context Correlates as Moderators Between Focal Correlates of WBM and Composite WBM Rate.**

| **Focal Correlate of WBM** | **Occupational Context Moderator** | ***ΔR^2^*** | ***F*(1)** | ***p*** |
| --- | --- | --- | --- | --- |
| *Stereotypes* |  |  |  |  |
| Yourself Competent | Job Task | 0.004 | 3.258 | 0.071 |
| Yourself Competent | Job Social | 0.003 | 2.307 | 0.129 |
| Yourself Competent | Job Physical | 0.001 | 0.791 | 0.374 |
| Others Competent | Job Task | < 0.001 | 0.054 | 0.816 |
| Others Competent | Job Social | < 0.001 | 0.069 | 0.793 |
| Others Competent | Job Physical | 0.001 | 0.786 | 0.376 |
| Coworker Competent | Job Task | 0.005 | 1.585 | 0.209 |
| Coworker Competent | Job Social | 0.003 | 0.834 | 0.362 |
| Coworker Competent | Job Physical | 0.003 | 0.837 | 0.361 |
| Yourself Warm | Job Task | < 0.001 | 0.170 | 0.680 |
| Yourself Warm | Job Social | < 0.001 | 0.089 | 0.765 |
| Yourself Warm | Job Physical | < 0.001 | 0.014 | 0.905 |
| Others Warm | Job Task | 0.002 | 1.625 | 0.203 |
| Others Warm | Job Social | < 0.001 | 0.306 | 0.580 |
| Others Warm | Job Physical | 0.004 | 3.270 | 0.071 |
| Coworker Warm | Job Task | 0.001 | 0.263 | 0.608 |
| Coworker Warm | Job Social | 0.001 | 0.394 | 0.531 |
| Coworker Warm | Job Physical | 0.011 | 3.545 | 0.061 |
| *Interpersonal Climate* |  |  |  |  |
| Climate Incl. Equitable | Job Task | 0.001 | 0.194 | 0.660 |
| Climate Incl. Equitable | Job Social | < 0.001 | 0.084 | 0.772 |
| Climate Incl. Equitable | Job Physical | 0.001 | 0.290 | 0.591 |
| Climate Incl. Integration | Job Task | 0.001 | 0.317 | 0.574 |
| Climate Incl. Integration | Job Social | < 0.001 | 0.062 | 0.803 |
| Climate Incl. Integration | Job Physical | 0.005 | 1.479 | 0.225 |
| Climate Incl. DM | Job Task | 0.001 | 0.345 | 0.557 |
| Climate Incl. DM | Job Social | < 0.001 | 0.007 | 0.932 |
| Climate Incl. DM | Job Physical | < 0.001 | 0.124 | 0.725 |
| Civility Climate | Job Task | 0.001 | 0.367 | 0.545 |
| Civility Climate | Job Social | 0.001 | 0.445 | 0.505 |
| Civility Climate | Job Physical | 0.004 | 1.373 | 0.242 |
| Leader-Member Exchange | Job Task | 0.001 | 0.161 | 0.689 |
| Leader-Member Exchange | Job Social | 0.001 | 0.281 | 0.596 |
| Leader-Member Exchange | Job Physical | 0.004 | 1.320 | 0.251 |
| Perceived Org. Support | Job Task | < 0.001 | 0.092 | 0.762 |
| Perceived Org. Support | Job Social | < 0.001 | 0.050 | 0.824 |
| Perceived Org. Support | Job Physical | < 0.001 | 0.045 | 0.831 |
| Interpersonal Conflict | Job Task | 0.001 | 0.273 | 0.602 |
| Interpersonal Conflict | Job Social | 0.007 | 2.578 | 0.109 |
| Interpersonal Conflict | Job Physical | < 0.001 | 0.009 | 0.924 |
| *Weight Control Climate* |  |  |  |  |
| Tolerate Neg. Wgt. Talk | Job Task | 0.003 | 0.921 | 0.338 |
| Tolerate Neg. Wgt. Talk | Job Social | < 0.001 | 0.148 | 0.700 |
| Tolerate Neg. Wgt. Talk | Job Physical | < 0.001 | 0.040 | 0.842 |
| Pressure for Thinness | Job Task | 0.015 | 4.744 | 0.030 |
| Pressure for Thinness | Job Social | 0.006 | 1.775 | 0.184 |
| Pressure for Thinness | Job Physical | 0.001 | 0.189 | 0.664 |
| Climate for Exercise | Job Task | 0.003 | 0.845 | 0.359 |
| Climate for Exercise | Job Social | 0.009 | 2.573 | 0.110 |
| Climate for Exercise | Job Physical | 0.001 | 0.409 | 0.523 |
| Wgt. Org. Support | Job Task | 0.002 | 0.507 | 0.477 |
| Wgt. Org. Support | Job Social | 0.004 | 1.270 | 0.261 |
| Wgt. Org. Support | Job Physical | 0.003 | 0.754 | 0.386 |
| Wgt. Social Support | Job Task | 0.005 | 1.435 | 0.232 |
| Wgt. Social Support | Job Social | 0.007 | 2.226 | 0.137 |
| Wgt. Social Support | Job Physical | 0.003 | 0.879 | 0.349 |
| Wgt. Diet Norms | Job Task | < 0.001 | 0.030 | 0.862 |
| Wgt. Diet Norms | Job Social | < 0.001 | 0.041 | 0.840 |
| Wgt. Diet Norms | Job Physical | < 0.001 | 0.022 | 0.881 |
| Wgt. Belittle | Job Task | 0.012 | 3.852 | 0.051 |
| Wgt. Belittle | Job Social | < 0.001 | 0.136 | 0.713 |
| Wgt. Belittle | Job Physical | < 0.001 | 0.031 | 0.860 |
| *Human Resources Policies* |  |  |  |  |
| Offer Exercise Program | Job Task | < 0.001 | 0.095 | 0.758 |
| Offer Exercise Program | Job Social | 0.005 | 1.353 | 0.246 |
| Offer Exercise Program | Job Physical | 0.006 | 1.691 | 0.194 |
| Offer Weight Loss | Job Task | < 0.001 | < 0.001 | 0.999 |
| Offer Weight Loss | Job Social | 0.003 | 0.917 | 0.339 |
| Offer Weight Loss | Job Physical | 0.002 | 0.722 | 0.396 |
| Offer Maintain Health | Job Task | 0.004 | 1.145 | 0.286 |
| Offer Maintain Health | Job Social | < 0.001 | 0.012 | 0.914 |
| Offer Maintain Health | Job Physical | 0.001 | 0.401 | 0.527 |
| Comm. Exercise Program | Job Task | 0.001 | 0.200 | 0.655 |
| Comm. Exercise Program | Job Social | 0.010 | 2.634 | 0.106 |
| Comm. Exercise Program | Job Physical | 0.002 | 0.487 | 0.486 |
| Comm. Weight Loss | Job Task | 0.002 | 0.423 | 0.516 |
| Comm. Weight Loss | Job Social | 0.005 | 1.389 | 0.240 |
| Comm. Weight Loss | Job Physical | 0.001 | 0.260 | 0.610 |
| Comm. Maintain Health | Job Task | 0.001 | 0.298 | 0.586 |
| Comm. Maintain Health | Job Social | 0.002 | 0.628 | 0.429 |
| Comm. Maintain Health | Job Physical | 0.003 | 0.761 | 0.384 |
| *Social Status* |  |  |  |  |
| Education Breakdown | Job Task | 0.001 | 0.533 | 0.466 |
| Education Breakdown | Job Social | 0.003 | 2.657 | 0.104 |
| Education Breakdown | Job Physical | 0.001 | 0.609 | 0.435 |
| Weight Comparison | Job Task | 0.002 | 1.532 | 0.216 |
| Weight Comparison | Job Social | < 0.001 | 0.218 | 0.640 |
| Weight Comparison | Job Physical | < 0.001 | 0.045 | 0.832 |
| Gender Breakdown | Job Task | 0.003 | 2.228 | 0.136 |
| Gender Breakdown | Job Social | < 0.001 | 0.044 | 0.834 |
| Gender Breakdown | Job Physical | < 0.001 | 0.045 | 0.832 |
| Manager | Job Task | 0.001 | 0.916 | 0.339 |
| Manager | Job Social | 0.004 | 2.898 | 0.089 |
| Manager | Job Physical | 0.001 | 0.972 | 0.325 |
| Visible Disability | Job Task | 0.001 | 0.851 | 0.357 |
| Visible Disability | Job Social | < 0.001 | 0.372 | 0.542 |
| Visible Disability | Job Physical | 0.005 | 4.049 | 0.045 |
| Ethnic Name | Job Task | < 0.001 | 0.032 | 0.859 |
| Ethnic Name | Job Social | 0.002 | 1.246 | 0.265 |
| Ethnic Name | Job Physical | < 0.001 | 0.043 | 0.836 |
| Body-Mass Index | Job Task | 0.001 | 0.351 | 0.554 |
| Body-Mass Index | Job Social | 0.004 | 2.182 | 0.140 |
| Body-Mass Index | Job Physical | 0.004 | 2.651 | 0.104 |
| Accent | Job Task | 0.002 | 1.466 | 0.226 |
| Accent | Job Social | < 0.001 | 0.083 | 0.774 |
| Accent | Job Physical | 0.003 | 2.781 | 0.096 |
| *Identity Management Strategies* |  |  |  |  |
| Redefinition Reassociate | Job Task | 0.002 | 1.598 | 0.207 |
| Redefinition Reassociate | Job Social | < 0.001 | 0.051 | 0.821 |
| Redefinition Reassociate | Job Physical | 0.005 | 3.730 | 0.054 |
| Redefinition Regenerate | Job Task | < 0.001 | 0.141 | 0.707 |
| Redefinition Regenerate | Job Social | 0.001 | 0.899 | 0.343 |
| Redefinition Regenerate | Job Physical | < 0.001 | 0.337 | 0.562 |
| Switch Deemphasize | Job Task | < 0.001 | 0.299 | 0.585 |
| Switch Deemphasize | Job Social | < 0.001 | 0.001 | 0.975 |
| Switch Deemphasize | Job Physical | 0.004 | 2.690 | 0.101 |
| Switch Emphasize | Job Task | < 0.001 | 0.013 | 0.908 |
| Switch Emphasize | Job Social | 0.005 | 3.860 | 0.050 |
| Switch Emphasize | Job Physical | < 0.001 | 0.038 | 0.845 |
| *Objectification by the Self* |  |  |  |  |
| Anxiety | Job Task | < 0.001 | 0.284 | 0.595 |
| Anxiety | Job Social | 0.001 | 0.672 | 0.413 |
| Anxiety | Job Physical | < 0.001 | 0.007 | 0.933 |
| Guilt | Job Task | 0.001 | 0.498 | 0.481 |
| Guilt | Job Social | < 0.001 | 0.303 | 0.582 |
| Guilt | Job Physical | < 0.001 | 0.016 | 0.899 |
| Shame | Job Task | < 0.001 | 0.015 | 0.903 |
| Shame | Job Social | < 0.001 | 0.025 | 0.875 |
| Shame | Job Physical | < 0.001 | < 0.001 | 0.994 |
| Depression | Job Task | 0.001 | 0.741 | 0.390 |
| Depression | Job Social | < 0.001 | 0.129 | 0.719 |
| Depression | Job Physical | 0.001 | 0.415 | 0.520 |
| Core Self-Evaluations | Job Task | < 0.001 | 0.195 | 0.659 |
| Core Self-Evaluations | Job Social | 0.001 | 0.877 | 0.349 |
| Core Self-Evaluations | Job Physical | < 0.001 | 0.403 | 0.526 |
| Weight-Bias Internalization | Job Task | 0.001 | 0.672 | 0.413 |
| Weight-Bias Internalization | Job Social | 0.001 | 0.930 | 0.335 |
| Weight-Bias Internalization | Job Physical | < 0.001 | 0.022 | 0.883 |
| Objectified Body Consc. | Job Task | < 0.001 | 0.086 | 0.770 |
| Objectified Body Consc. | Job Social | 0.003 | 2.752 | 0.098 |
| Objectified Body Consc. | Job Physical | < 0.001 | 0.251 | 0.617 |
| Intuitive Eating Trust | Job Task | 0.004 | 3.369 | 0.067 |
| Intuitive Eating Trust | Job Social | 0.001 | 1.006 | 0.316 |
| Intuitive Eating Trust | Job Physical | 0.003 | 2.292 | 0.130 |
| Body Dissatisfaction | Job Task | < 0.001 | 0.030 | 0.862 |
| Body Dissatisfaction | Job Social | 0.004 | 2.678 | 0.102 |
| Body Dissatisfaction | Job Physical | 0.022 | 16.727 | < 0.001 |
| *Objectification by Others* |  |  |  |  |
| Anti-fat Dislike | Job Task | 0.001 | 0.323 | 0.571 |
| Anti-fat Dislike | Job Social | 0.004 | 1.322 | 0.251 |
| Anti-fat Dislike | Job Physical | 0.003 | 1.130 | 0.289 |
| Anti-fat Fear | Job Task | 0.007 | 2.466 | 0.117 |
| Anti-fat Fear | Job Social | < 0.001 | 0.011 | 0.915 |
| Anti-fat Fear | Job Physical | 0.002 | 0.756 | 0.385 |
| Anti-fat Willpower | Job Task | 0.001 | 0.191 | 0.662 |
| Anti-fat Willpower | Job Social | 0.001 | 0.206 | 0.650 |
| Anti-fat Willpower | Job Physical | 0.005 | 1.487 | 0.224 |
| Thin Ideal | Job Task | 0.005 | 1.652 | 0.200 |
| Thin Ideal | Job Social | 0.004 | 1.386 | 0.240 |
| Thin Ideal | Job Physical | 0.002 | 0.735 | 0.392 |
| Negative Body Talk Other | Job Task | 0.003 | 1.043 | 0.308 |
| Negative Body Talk Other | Job Social | < 0.001 | 0.062 | 0.803 |
| Negative Body Talk Other | Job Physical | 0.007 | 2.402 | 0.122 |
| Negative Body Talk Self | Job Task | 0.005 | 1.510 | 0.220 |
| Negative Body Talk Self | Job Social | 0.006 | 1.948 | 0.164 |
| Negative Body Talk Self | Job Physical | 0.003 | 0.773 | 0.380 |

ΔR^2^ = change in R^2^ between the model with just the main effects of the focal correlate of WBM and the occupational job context moderator and the model with the main effects plus the interaction effect between two predictor variables. F = the F ratio for the change in R^2^; p = frequentist probability value of the F ratio for the change in R^2^.
